# Retinal microvascular density and inner thickness in Alzheimer’s disease and mild cognitive impairment

**DOI:** 10.3389/fnagi.2025.1477008

**Published:** 2025-02-28

**Authors:** Yehia Ibrahim, Antonella Macerollo, Rodolfo Sardone, Yaochun Shen, Vito Romano, Yalin Zheng

**Affiliations:** ^1^Department of Eye and Vision Sciences, University of Liverpool, Liverpool, United Kingdom; ^2^Department of Pharmacology and Therapeutics, Institute of Systems, Molecular and Integrative Biology, University of Liverpool, Liverpool, United Kingdom; ^3^Department of Neurology, The Walton Centre NHS Foundation Trust, Liverpool, United Kingdom; ^4^Statistics and Epidemiology Unit, Local Healthcare Authority of Taranto, Taranto, Italy; ^5^Department of Electrical Engineering and Electronics, University of Liverpool, Liverpool, United Kingdom; ^6^Department of Medical and Surgical Specialties, Radiological Sciences, and Public Health, University of Brescia, Brescia, Italy; ^7^Liverpool Centre for Cardiovascular Science, University of Liverpool and Liverpool Heart and Chest Hospital, Liverpool, United Kingdom

**Keywords:** Alzheimer’s disease, dementia, mild cognitive impairment, neurodegenerative disorders, optical coherence tomography, optical coherence tomography angiography, retinal biomarkers

## Abstract

**Background:**

Alzheimer’s disease (AD) is a major healthcare challenge, with existing diagnostics being costly/infeasible. This study explores retinal biomarkers from optical coherence tomography (OCT) and OCT angiography (OCTA) as a cost-effective and non-invasive solution to differentiate AD, mild cognitive impairment (MCI), and healthy controls (HCs).

**Methods:**

Participants from the CALLIOPE Research Program were classified as “Dem” (AD and early AD), “MCI,” and “HCs” using neuropsychological tests and clinical diagnosis by a neurologist. OCT/OCTA examinations were conducted using the RTVue XR 100 Avanti SD-OCT system (VISIONIX), with retinal parameters extracted. Statistical analysis included normality and homogeneity of variance (HOV) tests to select ANOVA methods. Post-hoc analyses utilized Mann–Whitney *U*, Dunnett, or Tukey-HSD tests based on parameters’ normality and HOV. Correlations with age were assessed via Pearson or Spearman tests. A generalized linear model (GLM) using Tweedie regression modeled the relationship between OCT/OCTA parameters and MMSE scores, correcting for age. Another ordinal logistic GLM (OL-GLM) modeled OCT/OCTA parameters against classes, adjusting for multiple confounders.

**Results:**

We analyzed 357 participants: 44 Dem, 139 MCI, and 174 HCs. Significant microvascular density (VD) reductions around the fovea were linked with MCI and Dem compared to HCs. Age-related analysis associated thickness parameters with HCs’ old age. Our OL-GLM demonstrated significant thickness/volume reductions in Inner_Retina and Full_Retina layers. Foveal avascular zone (FAZ) area and perimeter were initially not correlated with cognitive decline; however, OL-GLM significantly associated FAZ perimeter enlargement with Dem and MCI groups. Significant average and inferior peripapillary RNFL thinning were linked to Dem and MCI groups.

**Conclusion:**

This is the first study to examine VD changes in G grid sections among Dem, MCI, and HCs. We found a significant association between various VD parameters and cognitive decline. Most macular thickness/volume changes did not correlate with cognitive decline initially; however, our OL-GLM succeeded, highlighting the importance of the confounders’ corrections. Our analysis excluded individual retinal layer parameters due to limitations; however, the literature suggests their value. Our study confirmed existing biomarkers’ efficacy and uncovered novel retinal parameters for cognitive decline, requiring further validation.

## 1 Introduction

Dementia can be identified as a recognizable pattern of symptoms, including behavioral changes, impairments in daily activities, language and cognitive function disruptions, and memory deficiencies (Dening and Sandilyan, 2014). Structural and chemical alterations lead to neuronal loss and a reduction in brain volume for patients with dementia syndromes (Dening and Sandilyan, 2014). Alzheimer’s disease (AD) is a cause of dementia and is believed to be responsible for a majority of dementia cases (Qiu et al., 2009). An abnormal accumulation of amyloid “plaques,” an insoluble fibrous protein, and twisted fibers known as “neurofibrillary tangles” are the neuropathological changes of AD (Dening and Thomas, 2013). Mild cognitive impairment (MCI) refers to a level of cognitive decline that, while noticeable, does not significantly disrupt basic everyday activities (Gauthier et al., 2006).

There are currently over 50 million people worldwide living with dementia, a number that is expected to rise by 2030 to over 74 million people (Alzheimer’s Disease International, 2019; Prince et al., 2015). Traditional methods to identify dementia disorders include cerebrospinal fluid (CSF) analysis, brain imaging, genetic testing, and blood tests (NIA-Scientists, 2022). While these methods are valuable in assessing cognitive functions, they are significantly expensive and, thus, impractical to serve as screening methods for MCI or early-stage AD (Florek et al., 2018; Mounsey and Zeitler, 2018). Therefore, there is an unfulfilled need to discover cost-effective biomarkers to perform dementia screening.

The human retina is the only inner organ that can be directly observed non-invasively, offering a window to diagnose and manage ocular and systemic pathologies. The examination of the retina has been considered a potential biomarker for various neurodegenerative conditions in recent years (Snyder et al., 2021). Non-invasive imaging technologies include optical coherence tomography (OCT) and OCT angiography (OCTA), which are usually utilized to capture structural and vascular alterations in the retina, respectively (De Carlo et al., 2015). The retinal biomarkers used to diagnose MCI and AD can be classified into structural, vascular, and electrophysiological categories (Ge et al., 2021). Structural alterations include notable thinning of specific individual retinal layers or a few combined layers. For instance, peripapillary retinal nerve fiber layer thickness reduction was associated with MCI (Tao et al., 2019; Montorio et al., 2022) and with AD patients (Tao et al., 2019; Lemmens et al., 2020; Wang et al., 2022) compared against HCs. However, other studies (Poroy and Yücel, 2018; Almeida et al., 2019) suggested that these thickness changes were not significantly different in AD and MCI patients compared to HCs. Additionally, AD patients were linked with thinning in the macular retinal nerve fiber layer, ganglion cell layer, inner plexiform layer, and outer nuclear layers (Garcia-Martin et al., 2016). Moreover, AD patients were correlated with thinning in full retinal layer thickness (Jáñez-Escalada et al., 2019; Marziani et al., 2013).

Vascular biomarkers, another subset of retinal biomarkers, include microvascular density (VD) and foveal avascular zone (FAZ) changes. Recent studies have associated MCI with VD reduction in superficial layers (Chua et al., 2020; Bulut et al., 2018; Zhang et al., 2019), while AD patients were linked to VD decline in only specific regions of the superficial layers (Yan et al., 2021; Wu et al., 2020). Notably, inconsistencies in the literature are evident in two aspects: the choice of retinal layers analyzed and the regions reported to exhibit significant VD reductions (Chua et al., 2020; Bulut et al., 2018; Zhang et al., 2019; Yan et al., 2021; Wu et al., 2020). Similarly, related to vascular biomarkers, cognitively impaired patients had a significant FAZ enlargement compared to healthy controls (Bulut et al., 2018; Wu et al., 2020; O’Bryhim et al., 2021; Shin et al., 2021); however, FAZ changes were not significant in other studies (Zhang et al., 2019; Peng et al., 2021; Robbins et al., 2022; Biscetti et al., 2021; Yang et al., 2022). Existing biomarkers have limitations that could lead to conflicting results. Therefore, to understand the role of retinal biomarkers in neurodegenerative conditions, this study will explore structural and vascular retinal parameters to evaluate their efficacy in distinguishing AD and MCI from HCs. Robust statistical and post-hoc analyses of OCT/OCTA parameters will be used to identify relevant biomarkers, while discrepancies between our findings and the literature will be addressed through age-related analysis and generalized linear models adjusted for multiple confounding factors. Additionally, a novel VD-related biomarker will be proposed for future exploration.

## 2 Materials and methods

### 2.1 Building the cohort dataset

The dataset was collected in the CALLIOPE Research Program “Open Data Initiative for Dementia,” Italy. The study was conducted in adherence to the Declaration of Helsinki (Williams, 2008), complying with all ethical and legal requirements. The regional local ethical committee IRCCS “Giovanni Paolo XIII” approved all the phases (retrospective and prospective data) of this study. The data management and anonymization were compliant with the European General Data Protection Regulation. RS was responsible for the ethics and data management and protection.

The inclusion criteria required participants to be aged ≥70 years, to provide written informed consent (or consent from their legal guardians), and to complete neuropsychological assessments. Given the limited availability of patients without eye pathologies, we also included participants with maculopathy, including age-related macular degeneration (AMD), diabetic retinopathy (DR), and glaucoma. The cognitive assessments were based on the Mini-Mental State Examination (MMSE) score (Folstein et al., 1983), the Frontal Assessment Battery (FAB) (Appollonio et al., 2005), and the Apathy Evaluation Scale (Apathy Scale) (Guercio et al., 2015). Patients were excluded if their OCTA image quality was below a certain threshold, which was determined by an algorithm detailed in section 2.3.

The collected demographic details include gender, age, and eye pathology (if any). The participants were assessed by a neurologist according to their MMSE and FAB scores following the Diagnostic and Statistical Manual of Mental Disorders, Fifth Edition (DSM-5) criteria for dementia (Regier et al., 2013). The Apathy Scale (Guercio et al., 2015) was considered as additional supporting evaluation criteria for the neurologist.

The neurologist classified participants into three groups: (1) HCs: maximum scores in both assessments or no more than −2 points of selective deficit; (2) MCI: cognitive impairment was confirmed by deficient scores in one or more cognitive domains of MMSE or FAB (performance between −1 and −2 standard deviations); (3) AD and early AD (eAD) denoted as “Dem”: deficit scores in all cognitive domains (performances −2 standard deviations). The scale used to rate the dementia grade was the Clinical Dementia Rating (CDR) (Morris, 1993).

### 2.2 OCT/OCTA image acquisition

**Figure 1:**
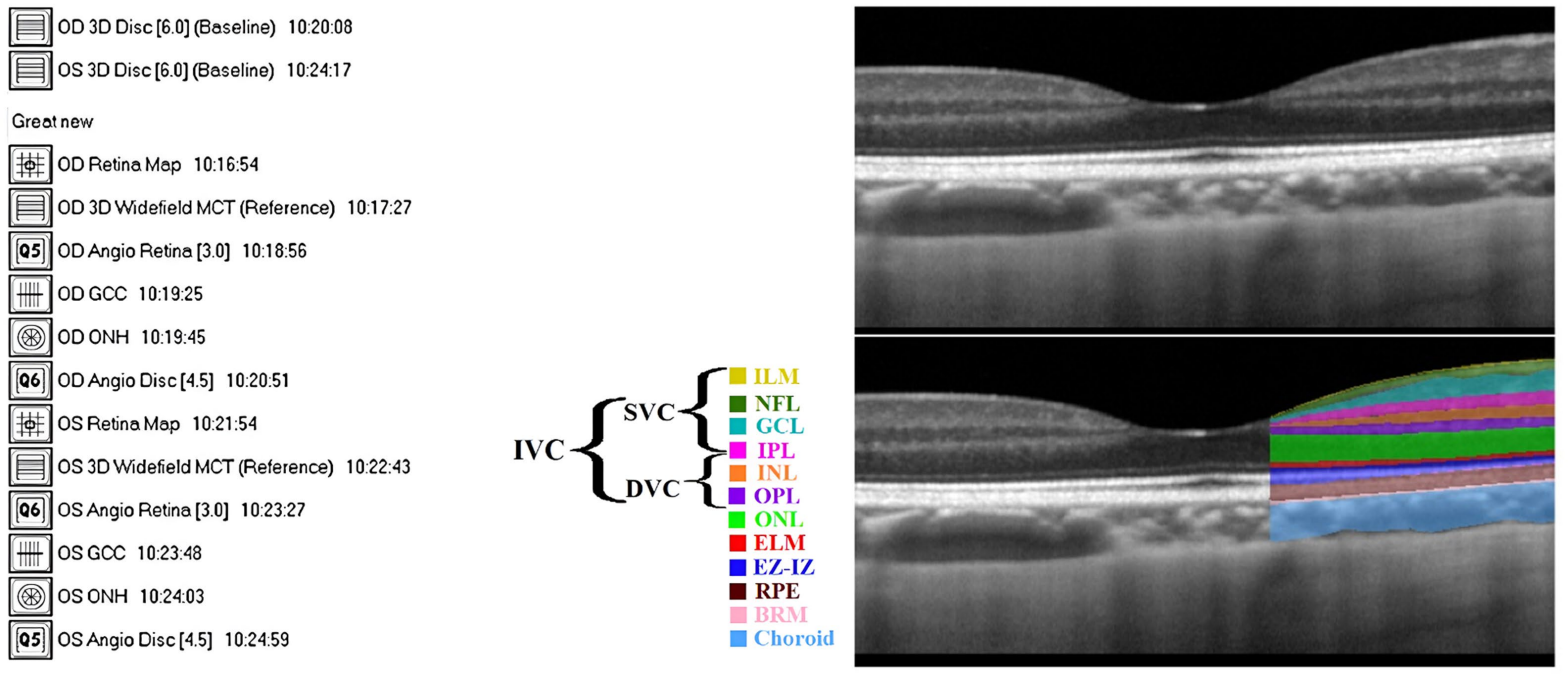
**(A)** Complete 14 scans provided by Avanti SD-OCT. **(B)** The definition of retinal layers in a spectral domain optical coherence tomography B-scan image: Top: unlabeled, Bottom: labeled (adapted from Ibrahim et al., 2023). Defined layers: inner limiting membrane (ILM), nerve fiber layer (NFL), ganglion cell layer (GCL), inner plexiform layer (IPL), inner nuclear layer (INL), outer plexiform layer (OPL), outer nuclear layer (ONL), external limiting membrane (ELM), ellipsoid zone and interdigitation zone (EZ-IZ), retinal pigment epithelium (RPE), and Bruch’s membrane (BRM).

All participants underwent OCTA examination by the RTVue XR 100 Avanti spectral domain OCT (SD-OCT) system (VISIONIX, formerly known as Optovue, Inc.), where OCT segmentation was performed using the built-in AngioVue module (version 2014.2.0.13). Patients’ eyes were not dilated prior to imaging. Figure 1A shows various scan types provided by Avanti SD-OCT, where scan patterns could be mainly grouped into raster scans around the fovea and radial scans around the optic disc.

A scan area of 3 × 3 mm^2^ centered on the fovea with an image resolution of 304 × 304 pixels was exported and analyzed. The nerve head map 4-mm diameter (NHM4) RTVue protocol was used to obtain optic disc imaging and parameters (Mesiwala et al., 2012). The NHM4 protocol consists of 12 radial scans 3.4 mm in length (452 A-scans each) and six concentric ring scans ranging from 2.5 to 4.0 mm (587 to 775 A-scans each), all centered around the optic disc contour line automated by the 3D protocol (Mesiwala et al., 2012). The areas between the A-scans were interpolated, and various parameters were automatically generated to describe the optic disc (Mesiwala et al., 2012).

An example of an SD-OCT b-scan is shown in Figure 1B (top). OCT can almost resolve all the retinal cellular layers, as demonstrated in Figure 1B (bottom). The included layers from inner to outer were the inner limiting membrane (ILM), nerve fiber layer (NFL), ganglion cell layer (GCL), inner plexiform layer (IPL), inner nuclear layer (INL), outer plexiform layer (OPL), outer nuclear layer (ONL), external limiting membrane (ELM), ellipsoid zone and interdigitation zone (EZ-IZ), retinal pigment epithelium (RPE), and Bruch’s membrane (BRM).

**Figure 2:**
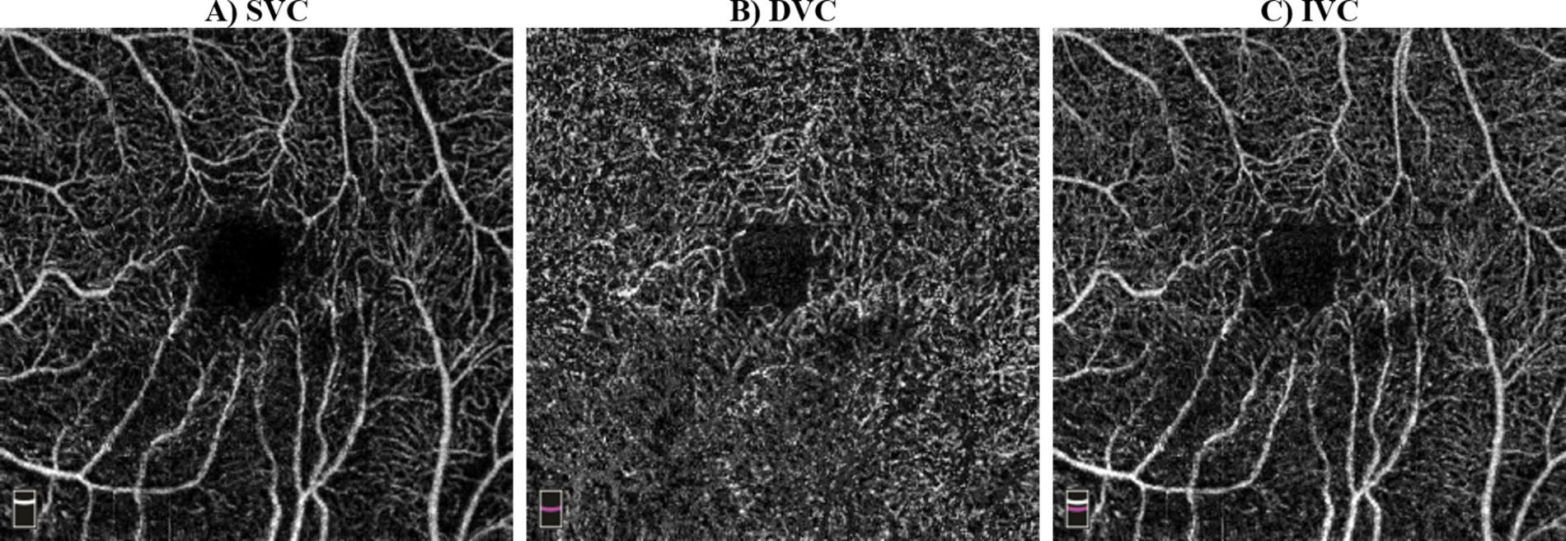
Examples of optical coherence tomography angiography (OCTA) scans: **(A)** superficial vascular complex (SVC) from inner limiting membrane (ILM) to 10 μm above the inner plexiform layer (IPL), **(B)** deep vascular complex (DVC) from 10 μm above IPL to 10 μm below outer plexiform layer (OPL), **(C)** inner vascular complex (IVC) from ILM to 10 μm below OPL.

The OCTA device captured 70,000 A-scans per second, with an axial resolution of 5 μm (Hashmani et al., 2018), and utilized a light source with an 840 nm wavelength. The RTVue software was used to extract OCTA projection maps (En-Face OCTA) of the microvasculature into superficial vascular complex (SVC), deep vascular complex (DVC), and inner vascular complex or inner retina (IVC). The SVC was defined from the ILM to 10 μm above the IPL, while the DVC was defined from 10 μm above the IPL to 10 μm below the OPL (Chen et al., 2020; Zhuang et al., 2020). Finally, the IVC was considered to include both SVC and DVC (Xie et al., 2023), in our case, from ILM to 10 μm below OPL (Hanumunthadu et al., 2021). Figure 2 included SVC, DVC, and IVC examples for an HC’s eye. The FAZ was automatically segmented by the software that accompanies the OCT device after projecting IVC layers. The FAZ area (FAZ_Area), perimeter (FAZ_Perim), acircularity index (AcirIndx), foveal area density (FD_300_Area_Density), and foveal length density (FD_300_Length_Density) were extracted automatically by the software. Notably, FD_300 indicates density at a distance of 300 μm from the FAZ (Çolak et al., 2021) and ignores the FAZ yet evaluates around it in IVC layers (Çolak et al., 2021).

### 2.3 Scan exclusions

The quality of the OCTA scans was inconsistent due to artifacts and noise. The signal strength index (SSI), which is a numeric grade between 0 and 100 indicating the scan quality (Yu et al., 2019), was initially used to exclude lower quality scans, specifically, SSI <40 (Ali et al., 2020; Zhang et al., 2015; Tang et al., 2017; Tanga et al., 2015). If both eyes’ OCTA images met the quality criteria, one eye was randomly chosen for analysis. Otherwise, only the eye with sufficiently high-quality OCTA images was selected for analysis.

### 2.4 Retinal biomarkers

The OCT/OCTA scans were grouped into optic disc (3D disc, angio disc, optic nerve head) and fovea-centered (ganglion cell complex, angio retina, retina map) categories. This study analyzed only retinal parameters automatically extracted by commercial software, grouped into ganglion cell complex (GCC), optic nerve head (ONH), Macula_3mm, Retina3DFlowDensity, and Retina Map.

#### 2.4.1 ONH related parameters

**Figure 3:**
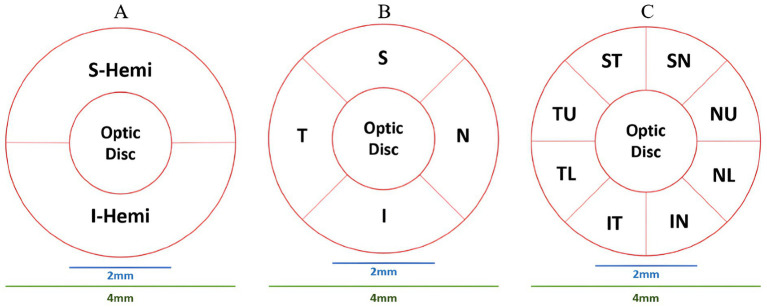
ONH partitioning methods. **(A)** Hemisphere S (S-Hemi) and hemisphere I (I-Hemi). **(B)** Superior (S), inferior (I), temporal (T), nasal (N) quadrants. **(C)** Supertemporal (ST), superonasal (SN), inferotemporal (IT), inferonasal (IN), nasal upper (NU), nasal lower (NL), temporal upper (TU), and temporal lower (TL).

ONH-based parameters included optic disc analysis and peripapillary retinal nerve fiber layer (pRNFL) thickness. Optic disc analysis included optic disc area/volume, cup area/volume, rim area/volume, and cup-to-disc (area, horizontal, and vertical) ratios. The pRNFL thickness was measured in hemisphere S (S-Hemi) and hemisphere I (I-Hemi) in Figure 3A and the average pRNFL thickness (González-García et al., 2009). Additionally, other pRNFL regions included quadrants-based Superior (S), Inferior (I), Temporal (T), Nasal (N), in Figure 3B, as well as supertemporal (ST), superonasal (SN), inferotemporal (IT), inferonasal (IN), nasal upper (NU), nasal lower (NL), temporal upper (TU), and temporal lower (TL) in Figure 3C.

#### 2.4.2 GCC related parameters

**Figure 4:**
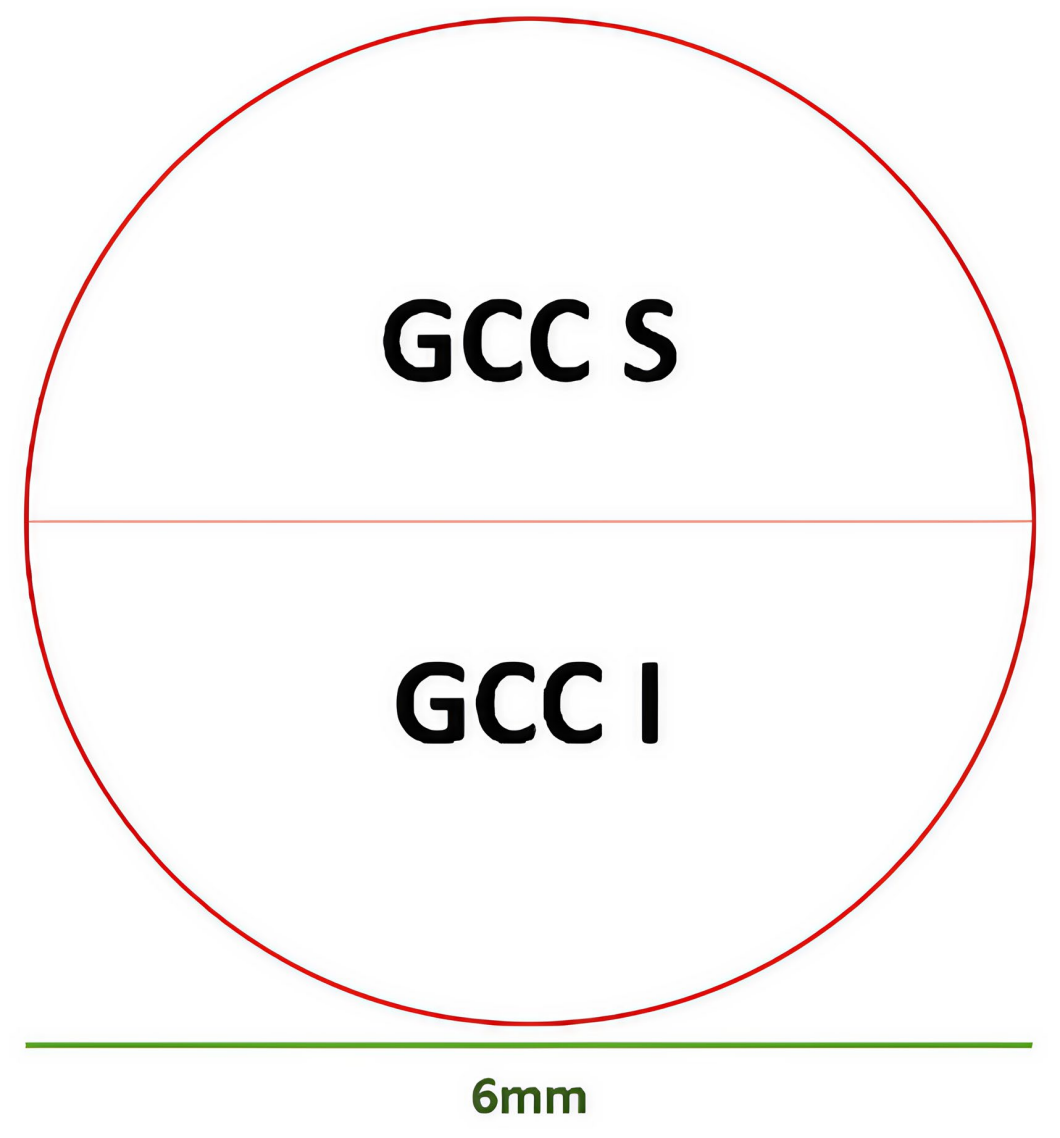
GCC partitioning methods. Superior (S) and inferior (I) partitions.

GCC-based parameters evaluated retinal thickness (μm) in Inner_Retina [ILM to 10 μm below OPL (Hanumunthadu et al., 2021)], Full_Retina [ILM to RPE/BRM complex (Hanumunthadu et al., 2021)], and Outer_Retina [10 μm below OPL to RPE/BRM complex (Venkatesh et al., 2019; Ye et al., 2020)]. GCC-parameters, following Figure 4, included average, S, I, and S-I thicknesses for Inner_Retina, Full_Retina, and Outer_Retina layers definitions (Hanumunthadu et al., 2021; Venkatesh et al., 2019; Ye et al., 2020; Rao et al., 2010). RTVue software also provided global loss volume (GLV), focal loss volume (FLV), and root mean square (RMS) (Rao et al., 2010).

#### 2.4.3 Retina map-related parameters

**Figure 5:**
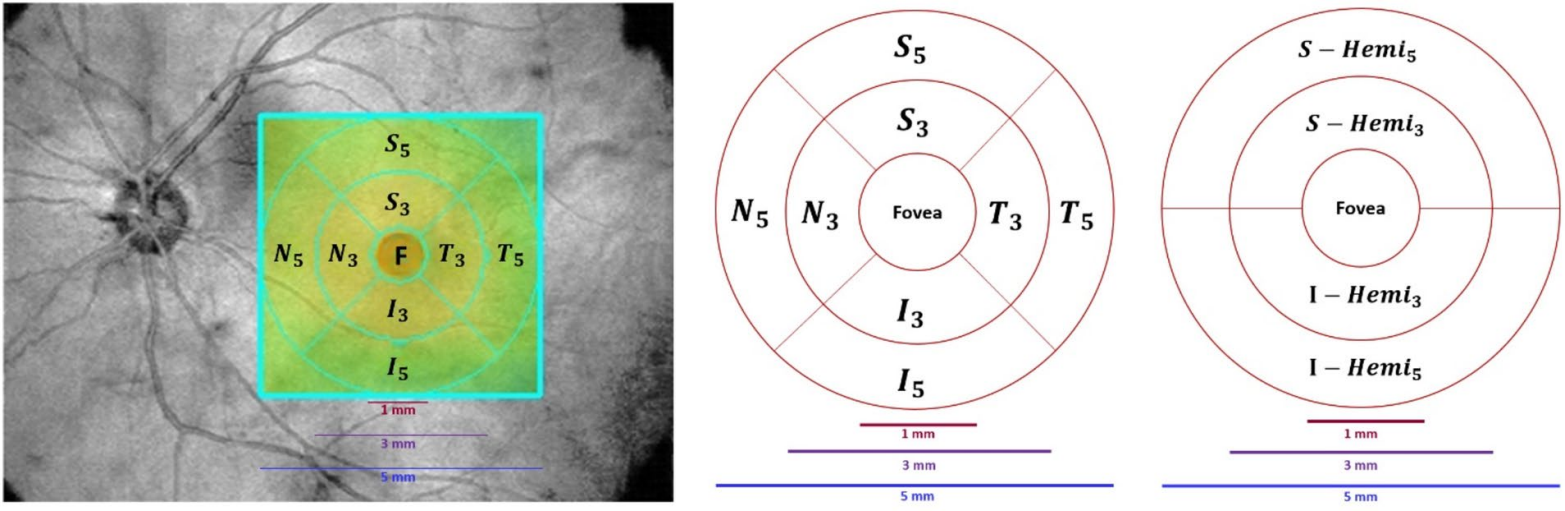
Retina map partition method. Left/Middle: Fovea (F), superior (S), inferior (I), temporal (T), nasal (N) quadrants in the inner ring (3 mm) and outer ring (5 mm). Right: Superior and inferior hemispheres in the inner ring (3 mm) and outer ring (5 mm).

The retina map measured retinal OCT thickness (μm) and volume (mm^3^) around the fovea using 1 mm, 3 mm, and 5 mm circles. In Figure 5, foveal thickness/volume was denoted by Fovea, while quadrants of (S_3_, I_3_, T_3_, N_3_) and (S_5_, I_5_, T_5_, N_5_) corresponded to parafovea and perifovea, respectively. Also, in Figure 5, hemispheres S_Hemi_3_ and I_Hemi_3_ represented 3 mm rings (Para), while S_Hemi_5_ and I_Hemi_5_ indicated 5 mm rings (Peri). Additionally, parafovea was the combined S_Hemi_3_ and I_Hemi_3_, while perifovea was the combined S_Hemi_5_ and I_Hemi_5_. The retina map (μm/mm^3^) was calculated in Inner_Retina and Full_Retina layers, similarly to GCC parameters, as well as in RPE thickness (Elevation).

#### 2.4.4 Retina3DFlowDensity related parameters

**Figure 6:**
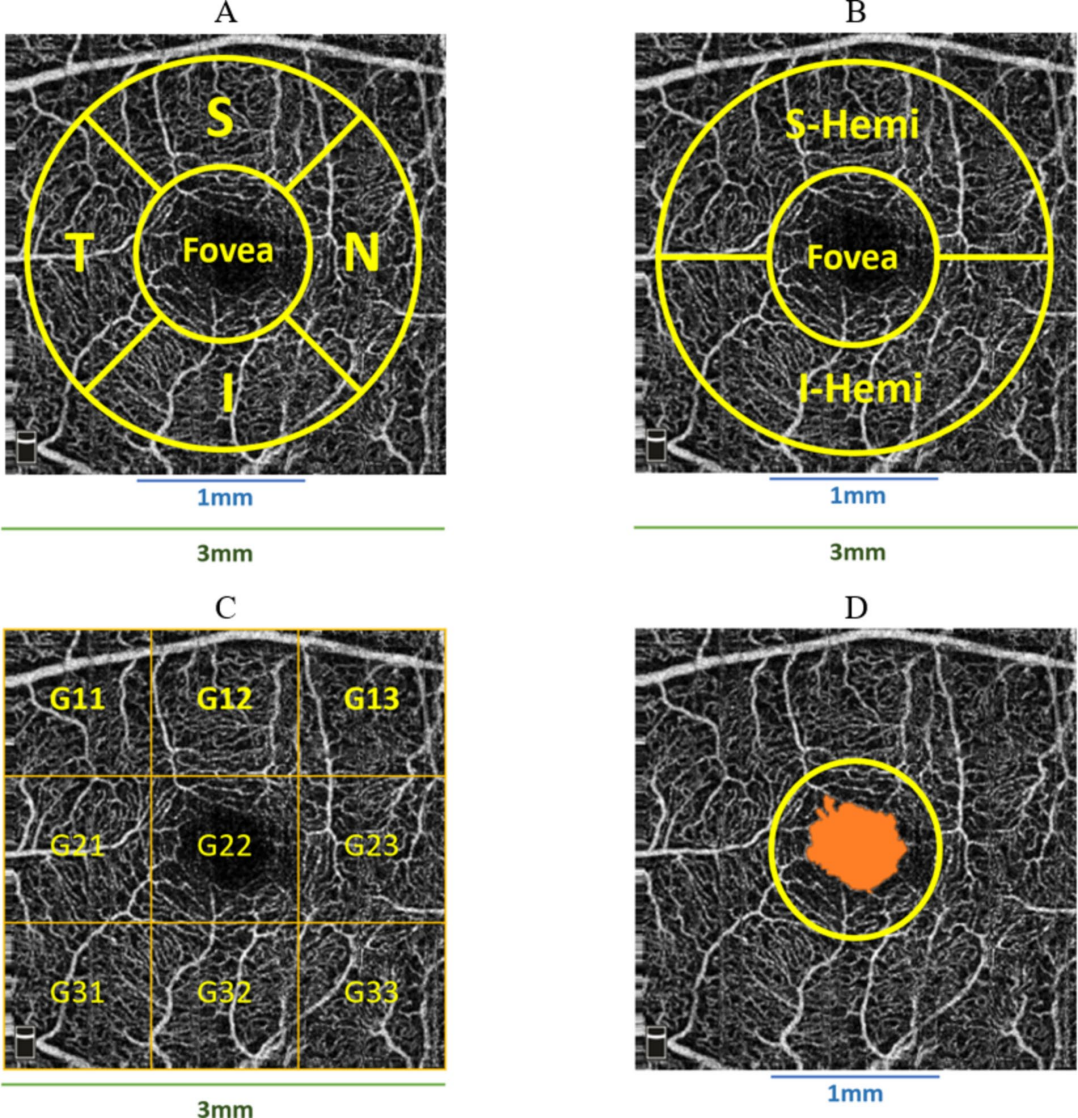
Various partitioning methods adopted by Optovue software around the fovea, **(A)** superior (S), inferior (I), temporal (T), nasal (N) quadrants; **(B)** superior (S-Hemi) and inferior (I-Hemi) hemispheres; **(C)** 3 × 3 grid sections; **(D)** foveal density 300 *μm* (FD_300) around fovea used to compute FD_300_Area_Density and FD_300_Length_Density.

Retina3DFlowDensity investigated retinal VD changes using 3 mm quadrants and hemispheres (S and I) partitioning. The analysis also included Whole_Image, differing from Early Treatment of Diabetic Retinopathy Study (ETDRS) by including the fovea region (Savastano et al., 2021). Whole_Image was further split into S_Hemi_3_ and I_Hemi_3_. Additionally, VD changes in the 3 × 3 grid G and foveal density (FD_300) were also examined as part of Retina3DFlowDensity parameters, as illustrated in Figures 6A–D. FD_300 zone was used to compute FD_300_Area_Density and FD_300_Length_Density. Other Retina3DFlowDensity parameters include Fovea, FAZ_Area, FAZ_Perim (Mo et al., 2016), and AcirIndx (Tam et al., 2011). Importantly, VD was computed for various retinal layers (SVC, DVC, IVC) defined in Figure 2.

#### 2.4.5 Macula_3mm related parameters

Macula_3mm parameters evaluated retinal thickness/volume changes in various quadrants and layers (ILM to IPL, ILM to RPE, ILM to BRM, and RPE to BRM). Following Figures 6A,B, thickness/volume analyses for distinct layers definitions included Center_1 (1 mm foveal ring), quadrants (S, I, T, N) in the 3 mm ring excluding 1 mm (1minus3), S and I hemispheres in 1minus3, and combined hemispheres (All_1minus3). Additionally, following Figure 4, thickness/volume analyses for distinct layers definitions involved S and I hemispheres field, where “field” indicated the inclusiveness of Center_1 region.

### 2.5 Overall statistical analysis

#### 2.5.1 Data imputation and selection

The R *mice* package (multivariate imputation by chained equations) was used to deal with missing values (van Buuren and Groothuis-Oudshoorn, 2011). A maximum threshold value of 50 percent was adopted as the limit for data eligibility for multiple imputations (Mishra and Khare, 2014; Mera-Gaona et al., 2021), with the number of iterations and multiple imputations set at 5. Interestingly, the number of missing values was ≤16% for certain parameters. The multiple imputation method was set as “default” using predictive mean matching for numeric data, logistic regression imputation for binary data with a factor of 2 levels, polytomous regression imputation for unordered categorical data (factor >2 levels), and a proportional odds model for (ordered, factor >2 levels) (Mishra and Khare, 2014). Once the En-Face Angio scans were selected for all classes, then these Angio scans were used to select the corresponding OCT/OCTA extracted parameters’ values. Basically, each Angio scan involves the patient’s ID and visit date/time, which were used to extract machine parameters associated with such conditions.

#### 2.5.2 Statistical analysis

The Shapiro–Wilk test was used for normality (Shapiro and Wilk, 1965). Non-normal parameters were tested with Kruskal–Wallis (Kruskal, 1952) to find significance among AD, MCI, and HCs. Normal parameters underwent Bartlett’s test for homogeneity of variance (HOV) (Bartlett, 1937). If Bartlett’s *p*-value >0.05, one-way ANOVA was used, assuming equal variances (Bartlett, 1937). Both Bartlett and Shapiro–Wilk ensured HOV and normality, respectively, as conjoined conditions for the ANOVA test. If Bartlett’s *p*-value ≤0.05, indicating unequal variances, Welch’s ANOVA was used (Delacre et al., 2019).

#### 2.5.3 Post-hoc analysis

After finding certain parameters to be significant (*p*_value_ <0.05), further examination should be carried out to investigate differences between the groups (Allen, 2017). Post-hoc analysis was used to determine which two groups correlate most with significant parameters (Allen, 2017). The Mann–Whitney *U* test was used for non-normal parameters across group combinations (Dem vs. MCI, Dem vs. HCs, MCI vs. HCs) (Sainani, 2012; Field, 2017). For normally distributed parameters, Dunnett’s test was used for failed HOV (Ruxton and Beauchamp, 2008; Hoffman et al., 2008), while the Tukey-HSD test was used for satisfied HOV (Abdi and Williams, 2010). The Mann–Whitney test results were adjusted for multiple comparisons, consistent with the adjustments made for Dunnet and Tukey’s tests.

#### 2.5.4 Age-related analysis

To explore correlations between parameters and age, statistical analysis was performed using the HCs group. Pearson correlation was used for parameters with normal and linear distributions (Sedgwick, 2012), while the Spearman non-parametric test was used for non-linear and non-normal parameters. Shapiro–Wilk and Kolmogorov–Smirnov were used to test for normality (Shapiro and Wilk, 1965) and linearity (Hong-Zhi and Bing, 1991), respectively. Parameters with Pearson or Spearman of *p*_value_ ≤0.05 were considered significantly correlated with age. This method was repeated for all the parameters.

#### 2.5.5 Generalized linear model

##### 2.5.5.1 Target variable: MMSE

A generalized linear model (GLM) based on Tweedie regression (TW-GLM) was implemented, following Moss (2020) and IBM-Corp (2021), to model the relationship between OCT/OCTA parameters and MMSE score. Tweedie regression was chosen for its ability to handle varying variances (Bonat and Kokonendji, 2017). Then, the MMSE score served as the dependent variable, gender as a categorical factor, and the studied parameters as covariates. A Type-III analysis of a 95% confidence interval was performed (Moss, 2020) because this method is widely applied in general cases and does not involve prior assumptions about the order of predictors (IBM-Corp, 2024). Age was included as an offset variable to account for scaling effects without estimating its independent contribution (IBM-Corp, 2023). The analysis was corrected for age effects on predictors (Field, 2017). The detailed results of OL-GLM can be found in section 1 of Supplementary material D.

##### 2.5.5.2 Target variable: classes

The original class variable was converted into numerical equivalent values “Class_Num” such that HCs, MCI, and Dem groups were given 0, 1, 2, respectively. Therefore, we implemented another ordinal logistic GLM (OL-GLM), following Moss (2020) and IBM-Corp (2021), to model OCT/OCTA parameters against Class_Num. A Type-III analysis of a 95% confidence interval was performed (Moss, 2020). Age was included as an offset variable to account for scaling effects without estimating its independent contribution (IBM-Corp, 2023), while gender and eye pathologies were added as OL-GLM confounding factors (IBM-Corp, 2021; IBM-Corp, 2023). The analysis was indeed corrected for age, gender, and eye pathology effects on predictors (Field, 2017).

While developing OL-GLM, we categorized the parameters hierarchically into primary types: ONH, GCC, Macula_3mm, Retina3DFlowDensity, and Retina Map. The parameters related to the retina map were further divided, to avoid intercorrelated parameters, into three subcategories: (a) Inner Retina Thickness parameters, (b) Inner Retina Volume parameters, and (c) Full Retina Thickness/Volume and RPE parameters. The detailed results of OL-GLM can be found in section 2 of Supplementary material D.

#### 2.5.6 Sample size calculation

Sample size calculation for 90% power was based on the effect size (Δ or Cohen’s *d*) from the systematic review and meta-analysis conducted by Thomson et al. (2015), which revealed a weighted mean difference (WMD) of 12.44 μm in pRNFL thickness between AD patients and HCs. We can proceed with a sample size calculation described by Equation 1:


(1) $$n={\left(\frac{Z_{\alpha /2}+Z_{\beta }\;}{\Delta /\sigma }\right)}^{2}$$

where *Z*_*α*/2_ = 1.96 for a two-tailed test at a 5% significance level (*α* = 0.05), Δ = 12.44 μm (effect size), σ = 15 μm (assumed standard deviation). Additionally, to calculate the sample size with 90% power instead of 80%, the *Z*-value for 90% power (*Z_β_*) should be 1.28 instead of 0.84 (Whitley and Ball, 2002; Flahault et al., 2005). Substituting the previous values into Equation 1, we get *n* = 15.28. Therefore, approximately 16 subjects per group were required to achieve 90% power to detect a weighted mean difference (WMD) of 12.44 μm in pRNFL thickness, assuming a standard deviation of 15 μm.

## 3 Results

Seven hundred and twenty seven OCT/OCTA scans were acquired from participants’ both eyes, including 120 (6 + 114), 304, and 303 scans from Dem (eAD + AD), MCI, and HCs, respectively. After excluding lower-quality scans and selecting one eye per participant, 357 participants’ scans remained for the analysis: Dem (eAD + AD), MCI, and HCs of 44 (3 + 41), 139, and 174, respectively. The demographic information of the cohort neuropsychological evaluation results is all shown in Table 1.

**Table 1:** Demographic information and clinical characteristics of the cohort analyzed in this study.

Number of included participants’ eyes (one eye per participant)	Dem (*N* = 44)	MCI (*N* = 139)	HCs (*N* = 174)	*p*-value
Number of male/female participants	15/29	77/62	59/115	**<0.001** ^a^
Eye pathology occurrence %	59.1%	48.2%	43.1%	0.156^a^
Eye pathology ( ${\mathrm{Maculopathy}}_{{\mathrm{includes}}_{AMD}}$ )	23	57	60	0.083^a^
Eye pathology (DR)	0	3	3	0.623^a^
Eye pathology (glaucoma)	2	7	12	0.724^a^
No eye pathology	19	72	99	0.241^a^
Age ( mean±std ) years	82.2 ± 6.2	80.7 ± 5.9	79.1 ± 5.9	**<0.01** ^b^
MMSE ( mean±std )	19.4 ± 4.5	25.3 ± 3.2	28.1 ± 2.0	**<0.001** ^b^
FAB ( mean±std )	8.0 ± 2.8	11.0 ± 3.3	14.6 ± 2.9	**<0.001** ^b^
Apathy Scale ( mean±std )	8.7 ± 7.4	4.1 ± 4.4	3.2 ± 4.1	**<0.001** ^b^

The participants’ cognitive levels were evaluated mainly based on the Mini-Mental State Examination (MMSE) score (Folstein et al., 1983), Frontal Assessment Battery (FAB) (Appollonio et al., 2005), and the apathy evaluation scale (Apathy Scale) (Guercio et al., 2015). The examined eye pathologies: (1) maculopathy, including age-related macular degeneration (AMD), (2) diabetic retinopathy (DR), and (3) glaucoma. Statistically significant parameters were highlighted in bold.

a The *p*-value was obtained by the chi-square test because these variables were categorical.

b The *p*-value was obtained by Kruskal–Wallis because these parameters were not normally distributed.

The detailed findings of initial statistical analysis, post-hoc analysis, age-related analysis, and GLM results (TW-GLM and OL-GLM) can be found in Supplementary material A–D, respectively. We shall only describe the main results in the next section.

### 3.1 Statistical analysis results of OCT/OCTA parameters across Dem, MCI, and HCs

#### 3.1.1 ONH, GCC, and Macula_3mm

CupVolume and pRNFL thickness in the SN1 sector showed statistical significance (*p* = 0.048 each). ONH CupVolume decreased, while pRNFL thickness increased in the Dem and MCI groups compared to HCs. Other ONH, GCC, and Macula_3mm parameters were not significant (*p*-value >0.05) when comparing the three groups.

#### 3.1.2 Retina map

The Inner_Retina layers in the retina map showed significant thinning in parafovea, S_Hemi_3_, N_3_, perifovea, I_Hemi_5_ sections for Dem and MCI, with no significant changes in other parafovea and perifovea quadrants. Moreover, significant Inner_Retina volume reduction was observed in parafovea, S_Hemi_3_, I_Hemi_3_, S_3_, I_3_, T_3_, perifovea, S_Hemi_5_, I_Hemi_5_, S_5_, and N_5_ partitions for Dem and MCI groups; however, other quadrants’ volume changes were not significant.

**Figure 7:**
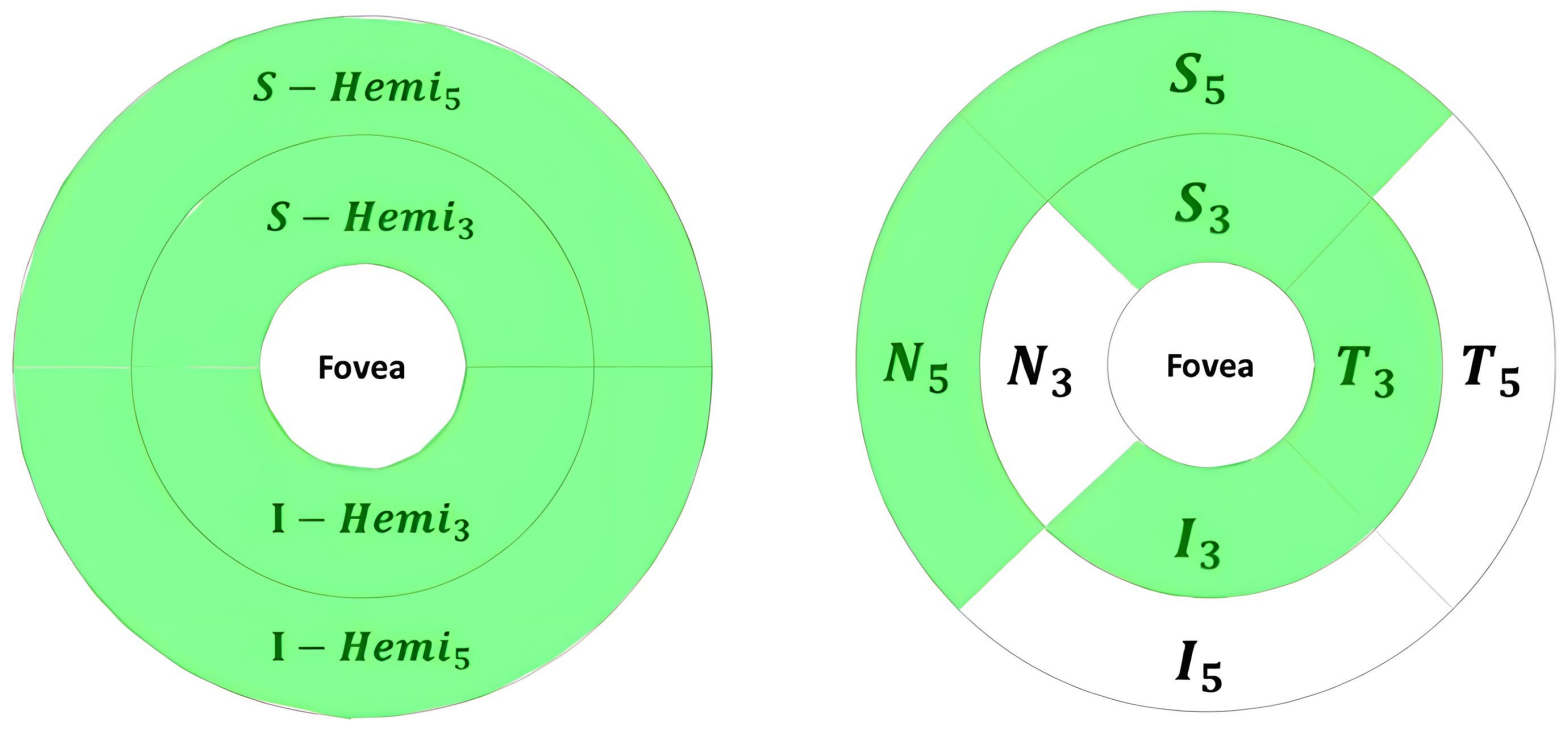
Significant volume changes of retina map parameters in the inner retina for Dem and MCI compared independently against HCs, highlighted in green.

Only T_3_ quadrant of Full_Retina showed a significant volume reduction in Dem and MCI groups compared to HCs, with negligible volume/thickness parameters in other Full_Retina quadrants. The RPE thickness (Elevation) was significantly thinner for the Dem and MCI groups only in I_3_ quadrant, with negligible changes in other quadrants. Figure 7 illustrates an example of significant parameters in the inner retina, with the green-shaded areas indicating statistical significance (*p* < 0.05), computed following methods outlined in section 2.5.2 across the three groups.

#### 3.1.3 Retina3DFlowDensity

The significant parameters included SVC VD decline in Whole_Image, Whole Image in S_Hemi_3_ and I_Hemi_3_ for Dem and MCI groups. Significant Retina3DFlowDensity parameters within the 3 mm ETDRS grid included SVC VD decrease for Dem and MCI patients in parafovea/inner ring across all quadrants (S_3_, I_3_, T_3_, N_3_), parafovea S_Hemi_3_ and I_Hemi_3_ parts, and the full SVC parafovea. Furthermore, the SVC VD of Whole 3 mm ETDRS was significantly reduced for the Dem and MCI groups. However, SVC_L1_Fovea, representing Fovea VD, was insignificant in group comparisons.

**Figure 8:**
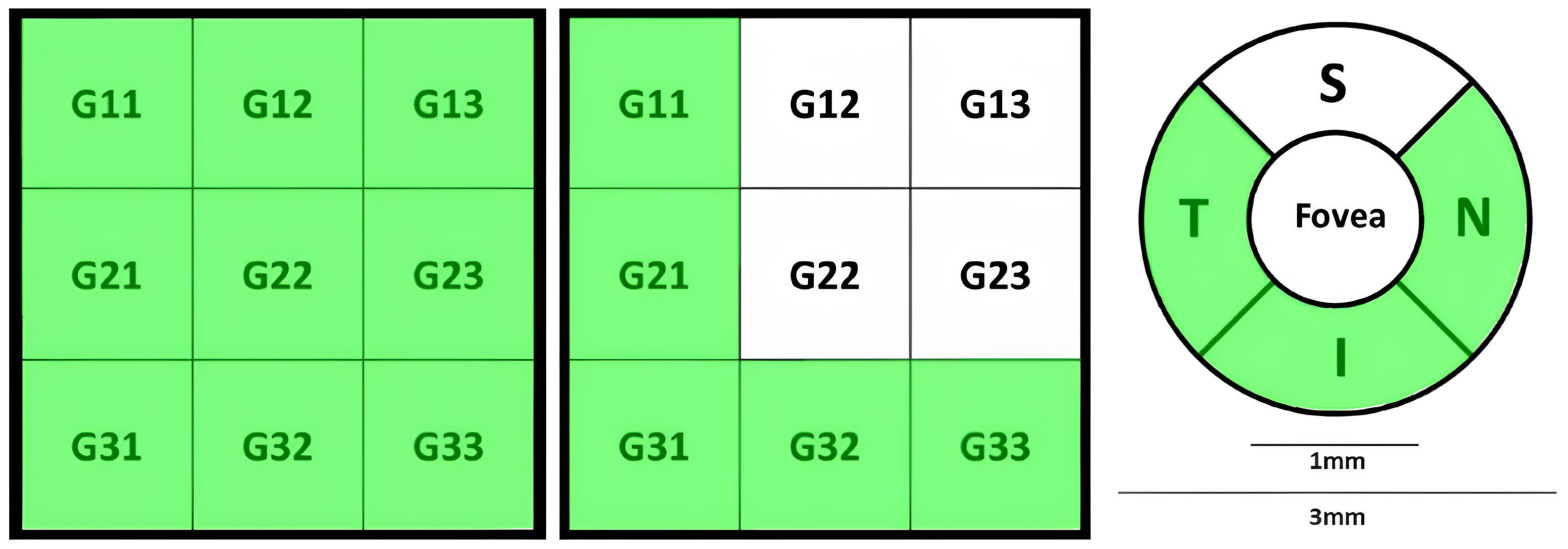
Retina3DFlowDensity significant parameters for Dem and MCI compared to HCs highlighted in green, from left to right: SVC 3 × 3 grid sections, DVC 3 × 3 grid sections, quadrants-based in DVC.

The other significant parameters of Retina3DFlowDensity following the 3 × 3 grid were SVC VD diminution of G11 throughout G33 parts for Dem and MCI patients. Notably, most significant parameters in SVC layers were also significant in DVC layers; however, the DVC VD of Fovea, parafovea S_Hemi_3_, and S_3_ failed to reach statistical significance. Other negligible Retina3DFlowDensity parameters in DVC following the 3 × 3 grid were G12, G13, G22, and G23. Both FD_300_Area_Density and FD_300_Length_Density were significantly lowered for Dem and MCI patients against HCs, while variations in FAZ_Area, FAZ_Perim, and AcirIndx lacked statistical significance. Figure 8 illustrates significant parameters with green shades indicating statistical significance (*p* < 0.05)

### 3.2 Post-hoc results

#### 3.2.1 Post-hoc after significant parameters

##### 3.2.1.1 All three comparisons—significant parameters

The analysis of parameters showed a significant SVC VD deterioration in G12, G21, G31, and G33 sections based on a 3 × 3 grid from Retina3DFlowDensity analysis for all group comparisons. Other significant parameters included lower SVC VD in parafovea hemispheres S and I, full parafovea, S_3_, I_3_, N_3_, and whole 3 mm ETDRS. Moreover, whole image analysis revealed a significant SVC VD decline in Whole_Image and its split into hemispheres S and I. Additionally, Length_Density of FD_300 was significantly lower in all comparisons.

##### 3.2.1.2 Dem vs. HCs & Dem vs. MCI comparisons—significant parameters

For Dem vs. HCs and Dem vs. MCI comparisons, significant Retina3DFlowDensity parameters included only DVC VD decline at G31, G32, and G33 sections, I_3_ quadrant, Whole 3 mm ETDRS, Whole_Image in hemispheres S and I, Whole_Image, and SVC VD decline at G13 section. In conjunction with the retina map, significant correlations in Dem vs. HCs and Dem vs. MCI comparisons included volumetric reductions in S_Hemi_3_, I_Hemi_3_, parafovea, T_3_, S_Hemi_5_, and perifovea parts of Inner_Retina layers.

##### 3.2.1.3 Dem vs. HCs & MCI vs. HCs comparisons—significant parameters

Additional Retina3DFlowDensity parameters were significant only in specific comparisons (Dem vs. HCs & MCI vs. HCs), including SVC VD decrease at G11, G23, and G32 sections, and T_3_ quadrant.

##### 3.2.1.4 Only certain comparisons—significant parameters

Other significant Retina3DFlowDensity parameters for the Dem vs. HCs differentiation task included DVC VD reduction at T_3_ and N_3_ quadrants, I_Hemi_3_, parafovea, G11, G21 sections, and FD_300 Area_Density. Notable retina map parameters discriminating Dem vs. HCs of Inner_Retina layers included volumetric shrinkage in $S_{3}$ , $I_{3}$ , $I\_{\mathrm{Hemi}}_{5}$ , $S_{5}$ , $N_{5}$ parts, as well as thickness thinning in $S\_{\mathrm{Hemi}}_{3}$ , $I\_{\mathrm{Hemi}}_{5}$ , parafovea, $N_{3}$ , and perifovea parts. Additionally, only $I_{3}$ quadrant of the RPE thickness (Elevation) parameter was significantly smaller for the Dem group. Compared to HCs, ONH parameters showed significant pRNFL thickening in the SN1 sector for MCI and substantial volumetric reduction in ONH Cup Volume for Dem.

#### 3.2.2 Post-hoc of non-significant parameters

Despite the initial lack of significance, certain parameters may show notable significance in specific comparisons. Post-hoc analysis revealed significant GCC parameters, including average thickness reduction at Full_Retina in S for MCI vs. HCs, and thickness decrease at Inner_Retina in S-I quadrant for Dem vs. MCI. Other significant ONH parameters included an increase of rim area and optic disc volume for Dem vs. HCs.

Contrary to initial results, certain Macula_3mm parameters showed statistical significance, including volume reductions ( ${\mathrm{mm}}^{3}$ ) from ILM to RPE layers at $S\_{\mathrm{Hemi}}_{3}$ and N parts, both under 1minus3 definition, when comparing Dem vs. HCs. Significant Retina3DFlowDensity parameters emerged from post-hoc analysis, including DVC VD reduction at $S\_{\mathrm{Hemi}}_{3}$ , G13, and G23 sections for Dem vs. HCs.

The post-hoc analysis also identified significant retina map parameters such as volumetric reduction in $S\_{\mathrm{Hemi}}_{3}$ , $S\_{\mathrm{Hemi}}_{5}$ , and $T_{5}$ parts of Full_Retina layers, and shrinkage in $I_{5}$ and $T_{5}$ quadrants of Inner_Retina for Dem vs. HCs. Moreover, other retina map parameters, including notable thickness reduction of Inner_Retina layers in $I\_{\mathrm{Hemi}}_{3}$ , $S_{3}$ , $I_{3}$ , $S\_{\mathrm{Hemi}}_{5}$ , and $N_{5}$ parts for Dem vs. HCs. Lastly, only $N_{5}$ section of RPE thickness (Elevation) was significantly reduced in Dem vs. MCI.

### 3.3 Age-related analysis results

Data from 174 HCs were analyzed with demographic age from 56 to 94 years, and mean±standard deviation of 79±5.9 years. None of the studied parameters met linearity and normality conditions for Pearson parametric testing (Sedgwick, 2012), and hence, Spearman non-parametric testing was used. Notably, age was majorly correlated with macular thickness/volume and VD-based parameters, with no correlations with FAZ area changes. To avoid a lengthy discussion, we will discuss only a few age-related parameters that we believe will help in clarifying a few discrepancies between our results and those in the literature.

### 3.4 GLM results

#### 3.4.1 Target variable: MMSE

Age was heavily correlated with OCT/OCTA parameters, and hence, it was used as an offset variable to correct age effects on predictors (Field, 2017). The TW-GLM identified many parameters correlated with the MMSE score, and we shall highlight key associations only.

In terms of GCC-based parameters, a lower MMSE score was correlated with inferior thinning in $\left(\mathrm{Inner,Full,Outer}\right)\_\mathrm{Retina}$ , and superior thinning in $\left(\mathrm{Inner,Full}\right)\_\mathrm{Retina}$ . Other significant associations with lower MMSE included average S-I thickness in $\left(\mathrm{Full,Outer}\right)\_\mathrm{Retina}$ and FLV, GLV, RMS, and average Inner_Retina thickness.

ONH-based parameters were correlated with MMSE decline, except for vertical cup-to-disc ratio as well as rim and disc volumes. The pRNFL thickness parameters were correlated with MMSE, except for these sections: T, S, NL, TL, ST2, NL1, NL2, IN2, IN1, IT2, and TL1.

Macula_3mm parameters correlated with MMSE decline, with a few exceptions described in Supplementary material D. VD, changes around the fovea were significantly associated with MMSE decline, except for FAZ area changes that will be discussed further.

Retina map age-corrected parameters that were correlated with MMSE decline included Inner_Retina thickness thinning ( $T_{3}$ , $T_{5}$ , $I_{5}$ ), Inner_Retina volume reductions ( $N_{3}$ , $I_{5}$ ), Full_Retina thickness decrease (ParaFovea, PeriFovea, $S\_{\mathrm{Hemi}}_{3}$ , $T_{3}$ , $S_{3}$ , $T_{5}$ , $N_{5}$ , $I_{5}$ ), and Full_Retina volume reduction ( $S\_{\mathrm{Hemi}}_{3}$ , $I\_{\mathrm{Hemi}}_{3}$ , $S_{3}$ , $I_{3}$ , $T_{3}$ , $N_{3}$ ). These findings are interesting since the initial statistical results correlated only Full_Retina volume decrease with cognitive decline among the groups.

#### 3.4.2 Target variable: classes

The OL-GLM demonstrated no correlation of GCC-related parameters against class variables, which aligned with our initial statistical analysis results.

Interestingly, our OL-GLM showed a significant correlation between class variables against many of ONH-based parameters, which is surprising since our initial analysis only found an association between class variable against CupVolume and SN1 pRNFL thickness. These inconsistencies will be discussed in section 4.3.

Parameters based on Macula_3mm were correlated with class variables. Some of these parameters following ILM to IPL layer definition (Tao et al., 2019; Zhang et al., 2019; Yang et al., 2022) include significant thickness reduction in the S hemisphere (1minus3 defined in section 2.4.5) and in the whole field (without partitions). Additional Macula_3mm parameters (ILM to IPL layers) include volumetric reduction of N and I-Hemi sections (1minus3) and the S and I hemispheres field (defined in Figure 4). On the contrary, the class variables studied by OL-GLM was associated with other Macula_3mm parameters (ILM to BRM layer definition) including a significant thickness thinning in Center_1 , T and I (1minus3 definition), S and I hemispheres (1minus3), S and I hemispheres field (defined in Figure 4) sections, as well as in the whole field (without partitions). Moreover, following ILM to BRM layer definitions, the class variables analyzed by OL-GLM was also correlated with a notable volumetric shrinkage in Center_1 , I (1minus3), S-Hemi (1minus3), S-Hemi field (defined in Figure 4), as well as in the whole field (without partitions).

In terms of parameters related to retina map with Full_Retina layers definition, the OL-GLM linked class variables with significant thickness/volume reduction in fovea , $I\_{\mathrm{Hemi}}_{3}$ , $I_{3}$ , and whole perifovea sections. Moreover, the class variable was also associated with a notable thinning in whole parafovea, $S\_{\mathrm{Hemi}}_{5}$ , $I\_{\mathrm{Hemi}}_{5}$ , $S_{5}$ , and $T_{5}$ sections. Additionally, the OL-GLM associated class variables with a prominent volumetric decrease in $S_{3}$ , $T_{3}$ , $N_{3}$ , and $N_{5}$ sections. On the other hand, the OL-GLM correlated class variables with retina map parameters in Inner_Retina layers definition. Specifically, a significant thinning in fovea , parafovea, $S\_{\mathrm{Hemi}}_{3}$ , $N_{3}$ , $T_{3}$ , and $I_{3}$ sections were linked with class variables. Moreover, class variables was associated with prominent Inner_Retina volumetric reduction observed in fovea , parafovea, $S\_{\mathrm{Hemi}}_{3}$ , $I\_{\mathrm{Hemi}}_{3}$ , $N_{3},T_{3}$ , perifovea, $S\_{\mathrm{Hemi}}_{5}$ , $I\_{\mathrm{Hemi}}_{5}$ , $S_{5}$ , $I_{5}$ , $T_{5}$ , and $N_{5}$ partitions.

Following RPE thickness (Elevation) assessment, the OL-GLM related class variables with significant thinning in $I_{3}$ section, which is in line with our initial analysis. However, OL-GLM also found an association between class variables with notable thinning in $T_{3}$ , $T_{5}$ , $S_{5}$ , and $I_{5}$ sections.

Next, the class variables by OL-GLM was linked to most parameters based on Retina3DflowDensity. This indicates the importance of VD changes in SVC/DVC layers even after adjusting for confounding factors like age, gender, and eye pathologies. It is fair to say that the OL-GLM results are more reliable than the initial statistical results due to the necessary adjustments that were applied.

Moving our attention to Retina3DflowDensity parameters, the OL-GLM correlated these parameters with class variables. Some of these correlations agreed with our initial statistical analysis; however, OL-GLM discovered more parameters. A detailed description of the discrepancies between both methods (initial statistical analysis against OL-GLM) can be found in discussion section 4.1.3.

## 4 Discussion

Retinal biomarkers were analyzed to find their relationship with three groups (Dem, MCI, HCs) and with MMSE score. This section will compare our findings against similar studies in the literature, grouping parameters to facilitate a clear discussion.

### 4.1 Microvascular density changes against the literature

#### 4.1.1 General VD alterations

Research conducted by Chua et al. (2020), Bulut et al. (2018), Sadda et al. (2019), and Salobrar-Garcia et al. (2020) studied VD alterations in various layers’ definitions; however, only Bulut et al. (2018) used a similar SVC layer definition to ours. Our findings demonstrated substantial VD decline of Whole_Image and parafovea in SVC layers for Dem vs. HCs, aligned with Bulut et al. (2018). However, Bulut et al. (2018) also found a significant fovea VD decrease in SVC layers for Alzheimer’s type dementia; unlike our initial and post-hoc analyses. Bulut et al. (2018) excluded patients with macular pathologies, whereas our cohort included them due to old-aged participants, with the majority of them having eye pathologies.

Although our study followed definitions of SVC/DVC layers, it was worth discussing other works in the literature that adopted slightly different definitions of retinal layers. Importantly, Chua et al. (2020) showed a significant VD loss in superficial capillary plexus (SCP) deep capillary plexus (DCP) layers for AD against HCs. Notably, SCP and DCP layers were defined by Chua et al. (2020) and Zhang et al. (2019) from ILM to IPL and INL to OPL, respectively. The other study by Zhang et al. (2019) revealed significant VD reduction in SCP layers at parafovea, matching our results. Despite different SCP layer definitions by Chua et al. (2020) and Zhang et al. (2019), the findings emphasize parafovea VD changes in superficial layers and their correlation with cognitive decline.

#### 4.1.2 VD changes in certain sectors

Our initial findings agreed with Yan et al. (2021) that significant VD reductions in SVC (parafovea, $I\_{\mathrm{Hemi}}_{3}$ , $T_{3}$ , $N_{3}$ ) and in DVC ( $I\_{\mathrm{Hemi}}_{3}$ , $T_{3}$ ) were associated with AD compared to HCs. Our results further highlighted a notable VD decrease in SVC layers, specifically in $S\_{\mathrm{Hemi}}_{3}$ , $S_{3}$ , and $I_{3}$ . Additionally, a prominent VD decline was observed in DVC layers within parafovea, $N_{3}$ , and $I_{3}$ . On the contrary, these significant changes in SVC and DVC layers were not significant according to Yan et al. (2021). Intriguingly, both studies (our and Yan et al., 2021) agreed on minor VD changes in fovea at SVC/DVC and in S at DVC between AD and HCs. These results doubt the reliability of VD reductions around the fovea. However, VD changes in specific SVC/DVC sections were reliably correlated with AD.

Wu et al. (2020) explored VD variations in SCP (3 μm below ILM to 15 μm below IPL) and DCP (from 15–70 μm below IPL till OPL) layers, using similar Avanti SD-OCT. Our findings match Wu et al. (2020), showing VD decrease in superficial layers (only $S_{3}$ ), and in deep layers (parafovea, $I_{3},T_{3}$ , $N_{3}$ ) linked to AD compared to HCs. Wu et al. (2020) associated VD decline in $S_{3}$ of superficial layers with AD; however, we observed VD decline in superficial layers (parafovea, $S\_{\mathrm{Hemi}}_{3}$ , $I\_{\mathrm{Hemi}}_{3}$ , $I_{3}$ , $T_{3}$ , and $N_{3}$ ) and $I\_{\mathrm{Hemi}}_{3}$ of deep layers. Wu et al. (2020) also found VD decline in $S_{3}$ of deep layers linked to AD, contradicting our results.

Our research found noteworthy VD decline in all superficial layers’ sections, except for fovea , for MCI against HCs; however, Wu et al. (2020) did not report similar significant changes. Additionally, both studies (ours and Wu et al., 2020) observed VD decline in $I_{3}$ and $T_{3}$ sections in deep layers for MCI, unlike HCs. Moreover, both studies showed some inconsistencies: (a) prominent deep layers VD decrease in $S_{3}$ was linked with MCI in Wu et al. (2020) but not observed in our results; (b) significant deep layers VD decrease in $N_{3}$ was correlated with MCI in our analysis but not significantly in Wu’s et al. (2020) study. These inconsistencies may arise from different layer definitions.

Yang et al. (2022) demonstrated a significant VD decrease in parafovea, $S_{3}$ , $I_{3}$ , $T_{3}$ , $N_{3}$ sections in SCP/DCP layers for MCI compared with HCs, which is consistent with our findings in superficial layers only.

We found negligible VD changes in all deep layers’ sections between MCI and HCs. Interestingly, the findings of Yang et al. (2022) and our study reported considerable VD decline in FD_300_Length_Density for MCI. Our initial findings of subtle changes in FAZ_Area, FAZ_Perim, and AcirIndx for MCI vs. HCs were also consistent with Yang et al. (2022). Other inconsistencies comparing MCI against HCs, our study showed minor VD changes in FD_300_Length_Density in our study but notable by Yang et al. (2022). Notably, SCP and DCP layers were defined by Yang et al. (2022) from ILM to IPL and from IPL to OPL, respectively. Despite different SCP and DCP layers definitions by Yang et al. (2022) and our SVC and DVC layers definitions, VD changes in superficial layers were similar.

#### 4.1.3 VD changes by GLM targeting class variables

The class variable studied by OL-GLM was correlated with Retina3DflowDensity parameters. Importantly, a few of these parameters were significant in our initial statistical analysis; however, they were not significant by OL-GLM. These parameters were VD changes in SVC (parafovea, $S\_{\mathrm{Hemi}}_{3}$ , $I\_{\mathrm{Hemi}}_{3}$ ) and in DVC ( Whole_ETDRS ). We believe that these parameters were heavily influenced by confounding factors.

Interestingly, the VD of SVC and DVC following the 3 × 3 grid in all sections were significantly correlated with class variables by OL-GLM, unlike our initial statistical analysis that failed to link cognitive decline with DVC VD decrease in Fovea, parafovea $S\_{\mathrm{Hemi}}_{3}$ , and $S_{3}$ sections. This indicates that the OL-GLM model, adjusted for confounders, confirmed the association of SVC/DVC VD decline in many sections and captured insights that were missed by traditional statistical analysis methods.

Similarly, the class variables studied by OL-GLM were correlated with notable VD decline in SVC quadrants ( $S_{3},I_{3},T_{3},N_{3}$ ), in DVC quadrants ( $S_{3},I_{3},T_{3},N_{3}$ ), as well as a significant decrease in FD_300_Area_Density and FD_300_Length_Density. The same parameters were correlated with MCI by Yang et al. (2022). On the contrary, another research by Yan et al. (2021) confirmed VD decline for the AD group against HCs only in $T_{3}$ and $N_{3}$ sections of SVC and in $T_{3}$ section of DVC. This indicates that the reliability of SVC/DVC VD in some sections is questionable for the AD group and more consistent for the MCI group.

### 4.2 Macular thickness/volume changes against the literature

The initial analysis showed negligible changes in macular thickness parameters. Post-hoc analysis correlated Dem with significant retinal volume reduction for ILM to RPE layers only in the S-Hemi and N quadrants against HCs. However, there is not enough research has discussed such parameters in the literature. On the contrary, our initial findings of negligible changes in macular thickness/volume contradicted the literature. For instance, Garcia-Martin et al. (2016) found thinning in macular RNFL (mRNFL), GCL, IPL, and ONL layers in AD vs. HCs. Similarly, Jáñez-Escalada et al. (2019) indicated a prominent thinning in mRNFL, GCL, IPL, INL layers, and full retinal layers thickness (FRT) (ILM to RPE/BRM) linked with AD. This discrepancy may be due to the use of different devices: Jáñez-Escalada et al. (2019) utilized Topcon spectral domain OCT, whereas we used Avanti SD-OCT. Additionally, age-related analysis linked thickness parameters (ILM to BRM and RPE to BRM) with old age for HCs, impacting parameters’ efficacy for detecting cognitive decline. Software limitations also prevented the analysis of individual layers against Dem and MCI groups.

Parameters based on Macula_3mm were correlated with class variables studied by OL-GLM; however, the initial statistical analysis showed no significant correlation with classes. This indicates that Macula_3mm parameters were heavily affected by many confounding factors (age, gender, and eye pathologies), and using these parameters must be considered cautiously.

Many retina map parameters, especially with Full_Retina layers definition, were correlated with class variables using OL-GLM. These results slightly contradict the initial statistical analysis that correlated classes with only volumetric shrinkage in $T_{3}$ section. Nevertheless, our results were somewhat consistent with Marziani et al. (2013) such that both studies demonstrated a correlation between AD and a prominent thinning in retina map parameters defined by Full_Retina layers, especially in fovea , $I_{3}$ , $T_{5}$ , and $S_{5}$ sections. However, Marziani et al. (2013) also associated AD with notable thinning in other sections of retina map parameters defined by Full_Retina layers. Specifically, a prominent thinning in $S_{3},T_{3},N_{3},I_{5},N_{5}$ sections were correlated with AD. We suspect this discrepancy was caused by slightly different tasks, such that our OL-GLM investigated three classes, while Marziani et al. (2013) investigated only AD against HCs. Notably, initial statistical analysis and OL-GLM results correlated multiple retina map parameters with class variables. However, unlike OL-GLM, the initial statistical analysis in Inner_Retina had minor thickness changes in fovea , $T_{3}$ , $I_{3}$ sections, as well as negligible volumetric alterations in fovea , $N_{3}$ , $I_{5}$ , $T_{5}$ sections. This indicates that the OL-GLM, being adjusted for multiple confounding factors (age, gender, and eye pathologies), managed to unravel more significant retina map parameters associated with cognitive decline.

### 4.3 Optic disc analysis and peripapillary RNFL thickness changes against the literature

Our initial and post-hoc analyses showed subtle changes in pRNFL thickness (Avg, S, I) between groups, contradicting Tao et al. (2019), Montorio et al. (2022), and Wang et al. (2022) but aligning with Poroy and Yücel (2018) and Almeida et al. (2019). AD in Poroy and Yücel (2018) and MCI in Almeida’s et al. (2019) research had negligible pRNFL (Avg, S, I) thickness changes compared to HCs. In contrast, Tao et al. (2019) found significant pRNFL (Avg, S, I) thinning in AD and MCI compared to HCs, with no differences between AD and MCI groups. Additionally, Wang et al. (2022) noticed a prominent pRNFL (Avg, S, I) thinning in AD against HCs, while the MCI group in Montorio et al. (2022) study also showed significant pRNFL (Avg, S, I) thinning compared to HCs.

Contrary to our initial analysis findings, Yan et al. (2021) reported significant pRNFL thinning (Avg, S-Hemi, I-Hemi, SN, nasal superior (NS), nasal inferior (NI), IN) for AD against HCs. Notably, the NS and NI sections by Yan et al. (2021) aligned with our NU and NL sections in Figure 3C Our initial analysis found significant changes in pRNFL thickness only in the SN1 sector, particularly for MCI, compared to HCs. This new correlation, SN1 pRNFL thickness thickening for MCI, still requires further research.

These major discrepancies between our results and findings in the literature could be explained by OL-GLM results. When applying OL-GLM against class variables, the average and inferior pRNFL thicknesses were significantly reduced against class variables, which was confirmed by Tao et al. (2019), Montorio et al. (2022), Lemmens et al. (2020), and Wang et al. (2022). However, Tao et al. (2019), Montorio et al. (2022), and Wang et al. (2022) also found a significant superior pRNFL thinning, which contradicts our analysis. Upon further investigation, the studied groups by Tao et al. (2019) were aged 71.40 ± 7.82, 71.67 ± 8.04, and 68.91 ± 5.88 for AD, MCI, and HCs, respectively, while the groups by Montorio et al. (2022) were aged 73 ± 6.6 and 72.66 ± 7.05 for MCI and HCs, respectively. Moreover, the groups by Wang et al. (2022) were examined by Cirrus HD-OCT, dissimilar to our Avanti SD-OCT device, and were aged 63.03 ± 9.06 and 61.55 ± 8.92 for AD and HCs, respectively. These groups were relatively younger than ours, with 82.2 ± 6.2, 80.7 ± 5.9, and 79.1 ± 5.9 for Dem, MCI, and HCs, respectively.

Building on previous discrepancies, our OL-GLM also associated significant thickness thinning in average, I-Hemi, NU/NS, and NL/NI pRNFL sections against class variables, which aligned with Yan et al. (2021) findings. However, Yan et al. (2021) also found other significant thickness thinning in S-Hemi, SN, and IN pRNFL sections, which contradicted our analysis. On further review, the groups by Yan et al. (2021), although examined by similar Avanti SD-OCT, were aged 63.89 ± 9.574 and 60.28 ± 7.096 for AD and HCs, respectively. In addition to the groups by Yan et al. (2021) being relatively younger than ours (82.2 ± 6.2, 80.7 ± 5.9, 79.1 ± 5.9 for Dem, MCI, and HCs), Yan et al. (2021) also excluded participants with eye diseases. However, due to our cohort’s being old and having a higher occurrence of eye pathologies, we decided to include these patients and correct these confounders in OL-GLM.

Therefore, the reliability of pRNFL parameters could be affected by using dissimilar OCT machines, comparing cohorts with different age groups, or including eye pathology.

In terms of ONH parameters based on optic disc analysis, the OL-GLM also correlated ONH CupVolume shrinkage with the class variable, similar to our initial statistical analysis. However, the OL-GLM also correlated class variables with other ONH alterations. Specifically, the optic disc area declined for MCI and increased for Dem, and the cup area/volume and cup-to-disc (area, horizontal, and vertical) ratios decreased for both groups. Conversely, the rim area/volume enlarged for Dem and MCI groups against HCs. We believed OL-GLM was able to uncover these correlations due to being corrected for age, gender, and eye pathologies.

In contrast to the literature, Tsai et al. (1991) reported an increased cup-to-disc ratio, cup volume, and decreased disc rim area for the AD group against HCs. Our results agreed with Tsai et al. (1991) only for ONH CupVolume reduction and were inconsistent with the cup-to-disc ratio and rim area change.

Additionally, Kromer et al. (2014) and Danesh-Meyer et al. (2006) demonstrated an increased cup-to-disc ratio for AD patients against HCs. Danesh-Meyer et al. (2006) also reported a reduced rim area/volume and an increased vertical cup-to-disc ratio. These results contradicted our findings and could be explained due to differently used devices. Specifically, Kromer et al. (2014) and Danesh-Meyer et al. (2006) used Heidelberg SD-OCT and laser imaging methodology dissimilar to our Avanti SD-OCT.

### 4.4 GCC changes against the literature

Tao et al. (2019) and Montorio et al. (2022) showed significant GCC (Avg, S, I) thinning in MCI versus HCs, with Tao et al. (2019) also noting GCC (average, S, I) thickness reduction for AD and not with MCI compared with HCs. Our study found no significant GCC differences between groups and no correlation between GCC parameters and class variables by OL-GLM adjusted for age, gender, and eye pathologies. This might have occurred due to dissimilar studied layers such that Tao et al. (2019) and Montorio et al. (2022) studied ILM to IPL layers, while we examined Inner_Retina, Full_Retina, and Outer_Retina layers. Future research may target examining GCC superficial layers.

### 4.5 FAZ changes against the literature

IVC FAZ area changes were not significant in initial statistical and post-hoc analyses. Our results matched Zhang et al. (2019) and Peng et al. (2021); however, they contradicted Wu et al. (2020) and O’Bryhim et al. (2021). Significant FAZ enlargement by Wu et al. (2020) for AD and MCI against HCs, while O’Bryhim et al. (2021) for amyloid positive, supported by CSF, against amyloid negative. The exclusion of high myopia patients by Wu et al. (2020) might explain this discrepancy, whereas our cohort potentially included myopic patients with infeasible corrections due to inaccessibility to axial length information (Sampson et al., 2017). Additionally, there were only nine amyloid-positive patients in O’Bryhim et al. (2021), much smaller than our 44 AD patients, suggesting a potential bias.

Bulut et al. (2018) found significant FAZ enlargement at SVC for AD patients but limited evidence in the literature. Conversely, negligible FAZ area changes at SCP, defined from ILM to IPL (Shin et al., 2021; Robbins et al., 2022), were reported by Robbins et al. (2022) between non-amnestic and amnestic MCI individually compared against HCs as well as by Shin et al. (2021) between MCI and HCs. Additionally, minor FAZ area changes at SCP, defined from ILM to GCL (Biscetti et al., 2021), between MCI and HCs were reported by Biscetti et al. (2021). Moreover, Shin et al. (2021) found a prominent DCP-FAZ area increase for MCI against HCs; however, the MCI group by Yang et al. (2022) had negligible DCP-FAZ area changes compared to HCs. This discrepancy could be explained due to the usage of distinct DCP definitions. Shin et al. (2021) defined DCP from INL to OPL, while Yang et al. (2022) defined DCP from IPL to OPL. Additionally, only 24 MCI were examined by Shin et al. (2021) compared to 268 MCI by Yang et al. (2022), further explaining this discrepancy.

Our OL-GLM failed to associate FAZ area changes with class variables; however, it demonstrated notable FAZ_Perim and AcirIndx changes linked with class variables. These minor FAZ area changes were confirmed by Yang et al. (2022); however, Yang et al. (2022) also showed negligible FAZ_Perim and AcirIndx changes for the MCI group. We believe that these parameters could be indeed correlated with a more severely impaired group such as AD; however, more future research with larger cohorts was needed to confirm this outcome.

### 4.6 GLM targeting MMSE and age analysis discussion

#### 4.6.1 FAZ area findings

The TW-GLM analysis, adjusted for age, revealed a strong correlation between FAZ area changes in IVC layers and MMSE scores. The age-related analysis failed to link old age to FAZ changes; however, this analysis was applied to HCs. Based on TW-GLM, FAZ changes might help detect cognitive decline, but more extensive future studies are still needed. The findings by Zhang et al. (2019), Peng et al. (2021), Robbins et al. (2022), Biscetti et al. (2021), and Yang et al. (2022) support our conclusion of minor FAZ area changes related to cognitive decline in initial or post-hoc analyses.

#### 4.6.2 Retinal VD findings

The age-related analysis linked some VD-based parameters with old age. This might explain inconsistencies between our cohort (Dem, MCI, HCs aged 82.20 ± 6.22, 80.70 ± 5.90, and 79.07 ± 5.90) and the cohort by Wu et al. (2020) (AD, MCI, HCs aged 69.94 ± 6.39, 67.81 ± 5.96, and 68.67 ± 5.85) and younger groups by Yang et al. (2022) (MCI, HCs aged 58.3 ± 8.3, and 51.0 ± 7.8). VD changes in deep retinal layers could be affected by age, impacting cognitive decline detectability. The TW-GLM, adjusted for age, found significant correlations between VD parameters and MMSE scores, confirming VD’s importance in detecting cognitive decline. Our findings highlighted the importance of significant VD changes in all G grid sections (G11 to G33); however, they were not linked with cognitive decline in the literature, requiring further investigation.

#### 4.6.3 Macular thickness/volume findings

Contradicting our statistical and post-hoc analysis, the age-adjusted TW-GLM model showed a strong correlation between thickness/volume changes around the fovea with MMSE scores. Age-related analysis revealed significant correlations between many Macula_3mm parameters (thickness/volume) and old age. Hence, TW-GLM identified the potential usage of these parameters by relating them with MMSE scores.

#### 4.6.4 GCC and ONH findings

The age-adjusted TW-GLM revealed significant correlations between many ONH-based and GCC-based parameters with MMSE scores, which is opposite to our initial statistical and post-hoc analyses. Contradicting our assumption, these predictors were not correlated with old age; however, the age analysis included only HCs without Dem/MCI groups. These results (GCC and ONH parameters) suggested that retinal structural changes (around the fovea and optic disc) might be linked to age and cognitive decline conjointly, requiring further research in larger cohorts.

### 4.7 Strengths

Our research work is unique because of the well-structured statistical and post-hoc analyses of OCT/OCTA machine-extracted parameters and their association with common neurodegenerative disorders. This is interesting since OCT/OCTA is a non-invasive brain study window. Moreover, our study also included age-related analysis to justify inconsistencies between our findings and the literature. Since many parameters were indeed correlated with age, we applied a TW-GLM analysis adjusted for age to find associations between predictors and MMSE scores. Additionally, we applied another OL-GLM to model class variables (Dem, MCI, and HCs) against studied parameters. This OL-GLM model, targeting class variables, was adjusted for age, gender, and various eye pathologies. To the best of our knowledge, our research is the first to find correlations between cognitive decline and VD changes based on G grid sections (G11 to G33). This finding invites further investigation into retinal VD changes as predictors for AD/MCI.

### 4.8 Limitations

One of the drawbacks of our study was the involvement of neurophysiological scores (MMSE, FAB) without gold standard biomarkers, i.e., CSF biomarkers ( $A\beta_{42}$ , t-tau, and p-tau) or MRI to support group diagnosis. Therefore, the diagnosis may not be properly verified by other reliable biomarkers/tools, which may lead to dementia caused by other pathological factors or reversible causes. Moreover, our study missed participants’ education and/or social status, which might have affected the results of neuropsychological tests. Furthermore, the lack of information regarding cardiovascular risk factors (e.g., diabetes, hypertension, and so on) represented another limitation, as these factors may influence retinal vasculature findings obtained via OCTA. Our cohort was also unbalanced (44 Dem, 139 MCI, 174 HCs) due to difficulties obtaining scans from cognitively declined patients.

Additionally, due the limited number of participants with healthy eyes—primarily because most were older patients—our study included individuals with various eye pathologies. As a result, our findings may differ from those in the literature, where most studies excluded participants with eye conditions. To account for this, we introduced OL-GLM, which adjusts parameters for confounding factors, including eye pathologies.

Moreover, the extracted Macula 6 mm parameters were excluded due to insufficient patients with this specific scan, and hence, a few parameters were missed in our analysis. Worth mentioning, the ETDRS grid system required 6 mm OCT b-scans around the fovea (Early Treatment Diabetic Retinopathy Study Research Group, 1991); however, our cohort’s patients had only 3 mm OCT b-scans around the fovea, restricting the analysis and preventing the use of the ETDRS grid quadrants system. Notably, our limited understanding of RTVue software’s development and evaluation may potentially affect the parameters’ reliability. Finally, the RTVue software failed to provide any parameters from the 12-mm 3D widefield mean choroid thickness (MCT) scan, thus restricting a more comprehensive analysis.

## 5 Conclusion

This research analyzed parameters’ statistical effectiveness in post-hoc comparisons. We examined VD changes using G grid sections (G11 to G33) between Dem, MCI, and HCs; however, further investigations are still required to verify this new biomarker. Significant VD reduction around the fovea was found, then confirmed by similar literature findings and by OL-GLM adjusted for multiple confounders.

The initial statistical analysis indicated a thickness increase in the SN1 section of the pRNFL for MCI compared to HCs and showcased a volumetric decrease in the ONH Cup volume for Dem compared to HCs. However, not enough evidence in the literature to support these parameters. Interestingly, other ONH-based parameters were also correlated with class variables analyzed by OL-GLM.

Our study highlighted the limitations of the FAZ area as a predictor of cognitive decline, supporting similar findings from the literature. The effectiveness of retinal parameters was compared against similar studies in the literature, finding consistent and inconsistent results.

Our initial analysis failed to link many macular thickness/volume changes with cognitive decline, contradicting the literature. We hypothesized these inconsistencies occurred due to confounding factors. Hence, age-related analysis, TW-GLM, and OL-GLM clarified these discrepancies. For instance, the age-adjusted TW-GLM correlated macular thickness/volume reductions with MMSE decline. Similarly, macular thickness/volume decline was also associated with the classes’ variable analyzed by OL-GLM, adjusted for age, gender, and eye pathologies.

We conclude the need to adjust the examined parameters for confounding factors and study individual layers independently. Future validation of newly discovered parameters with larger cohorts is still highly recommended. Future researchers may benefit from advanced segmentation tools to better define retinal layers and boundaries. Artificial intelligence (AI) may provide researchers with flexible analysis to explore more possible parameters’ interactions, correlating neurodegenerative disorders with thickness/volume changes of individuals or with various combinations of retinal layers. AI may also unravel new associations between AI-extracted parameters and cognitive decline, potentially facilitating earlier neurodegenerative disorder detection.

## Data Availability

The datasets presented in this article are not readily available because the cohort study data is not publicly available due to patient privacy restrictions and consent agreements. However, researchers affiliated to educational, or research institutions are encouraged to contact the authors for further information. Requests to access the datasets should be directed to YZ, yzheng@liv.ac.uk; RS, rodolfosardone@gmail.com.
