# Supplementary material for: Retinal microvascular density and inner thickness in Alzheimer’s disease and mild cognitive impairment
Source: Front Aging Neurosci. 2025 Feb 28;17:1477008. doi: 10.3389/fnagi.2025.1477008 (PMC11906703; doi:10.3389/fnagi.2025.1477008)
Supplement: Supplementary file 1 [file Table_1.DOCX]

Supplementary Material A

# Initial statistical analysis results

The results are presented in $mean (std)$ and arranged based on the various groups of parameters. Additionally, the statistically significant parameters have their p-value in **Bold** font. Importantly, the detailed description of all parameters’ names could be found under “Details of Parameters” section.

|  | **Parameter** | **Dem** | **MCI** | **HCs** | **p-value** |
| --- | --- | --- | --- | --- | --- |
| **GCC** | GCC_InnRet_Average | 96.388 (10.008) | 95.362 (9.661) | 96.874 (11.237) | 0.489 |
|  | GCC_InnRet_Superior_Avg | 96.725 (10.415) | 94.729 (9.846) | 96.391 (11.647) | 0.480 |
|  | GCC InnRet_Inferior_Avg | 96.025 (10.305) | 96.011 (10.373) | 97.371 (11.243) | 0.525 |
|  | GCC InnRet_S_I_Avg | 1.034 (5.590) | -1.391 (5.076) | -0.947 (4.655) | 0.112 |
|  | GCC_FLV | 1.510 (1.815) | 1.404 (2.011) | 1.385 (2.633) | 0.079 |
|  | GCC_GLV | 4.000 (4.164) | 4.415 (5.398) | 4.097 (5.372) | 0.437 |
|  | GCC_RMS | 0.103 (0.090) | 0.088 (0.062) | 0.091 (0.089) | 0.241 |
|  | GCC_FullRet_Average | 274.614 (18.272) | 274.769 (14.712) | 276.052 (16.375) | 0.647 |
|  | GCC_FullRet_Superior_Avg | 105.359 (46.525) | 92.054 (13.945) | 96.682 (17.952) | 0.134 |
|  | GCC_FullRet_Inferior_Avg | 108.978 (47.765) | 97.804 (13.464) | 99.280 (17.541) | 0.751 |
|  | GCC_FullRet_S_I_Avg | 0.847 (10.027) | -0.464 (8.846) | -0.689 (6.933) | 0.209 |
|  | GCC_OutRet_Average | 178.257 (12.716) | 179.323 (8.542) | 179.208 (9.193) | 0.848 |
|  | GCC_OutRet_Superior_Avg | 101.852 (24.748) | 95.745 (11.752) | 97.484 (13.996) | 0.491 |
|  | GCC_OutRet_Inferior_Avg | 104.642 (26.469) | 99.897 (18.805) | 99.788 (16.865) | 0.788 |
|  | GCC_OutRet_S_I_Avg | -0.315 (8.024) | -1.966 (5.632) | -1.369 (5.751) | 0.131 |
| **ONH**  **ONH** | ONH_DiscArea | 2.063 (0.389) | 1.980 (0.562) | 2.019 (0.388) | 0.789 |
|  | ONH_Area_C_D_ratio | 0.274 (0.162) | 0.286 (0.195) | 0.307 (0.159) | 0.335 |
|  | ONH_H_C_D_ratio | 0.505 (0.231) | 0.519 (0.261) | 0.572 (0.201) | 0.143 |
|  | ONH_V_C_D_ratio | 0.433 (0.224) | 0.457 (0.241) | 0.496 (0.188) | 0.283 |
|  | ONH_CupArea | 0.585 (0.394) | 0.601 (0.454) | 0.630 (0.370) | 0.480 |
|  | ONH_RimArea | 1.508 (0.391) | 1.364 (0.478) | 1.350 (0.341) | 0.081 |
|  | ONH_RimVolume | 0.166 (0.081) | 0.149 (0.088) | 0.144 (0.066) | 0.357 |
|  | ONH_Disc_Volume | 0.381 (0.194) | 0.328 (0.188) | 0.308 (0.139) | 0.076 |
|  | ONH_CupVolume | 0.071 (0.116) | 0.105 (0.141) | 0.111 (0.133) | **0.048** |
|  | ONH_Avg_RNFL | 95.560 (10.389) | 97.187 (10.892) | 97.005 (10.678) | 0.632 |
|  | ONH_RNFL_S-Hemi | 97.824 (10.271) | 98.689 (11.524) | 97.846 (11.472) | 0.971 |
|  | ONH_RNFL_I-Hemi | 94.507 (10.734) | 96.029 (11.705) | 96.104 (10.816) | 0.718 |
|  | ONH_RNFL_T | 73.485 (9.726) | 72.606 (10.597) | 72.617 (10.466) | 0.733 |
|  | ONH_RNFL_S | 117.576 (13.667) | 117.488 (15.702) | 116.940 (14.983) | 0.999 |
|  | ONH_RNFL_N | 72.673 (11.476) | 76.841 (12.420) | 76.188 (11.590) | 0.344 |
|  | ONH_RNFL_I | 120.785 (16.481) | 121.530 (16.690) | 122.242 (14.951) | 0.856 |
|  | ONH_RNFL_TU | 81.371 (11.851) | 79.101 (15.033) | 78.802 (13.782) | 0.408 |
|  | ONH_RNFL_ST | 128.834 (14.453) | 128.094 (18.485) | 129.389 (17.378) | 0.618 |
|  | ONH_RNFL_SN | 106.301 (15.616) | 107.591 (17.473) | 103.952 (16.406) | 0.157 |
|  | ONH_RNFL_NU | 75.171 (13.721) | 79.964 (13.710) | 79.765 (13.836) | 0.303 |
|  | ONH_RNFL_NL | 70.126 (10.477) | 73.966 (12.881) | 72.978 (11.374) | 0.321 |
|  | ONH_RNFL_IN | 112.263 (21.522) | 111.624 (21.120) | 112.103 (19.747) | 0.973 |
|  | ONH_RNFL_IT | 128.771 (19.076) | 131.520 (21.091) | 132.743 (17.540) | 0.720 |
|  | ONH_RNFL_TL | 64.599 (10.774) | 66.225 (10.819) | 66.617 (11.026) | 0.534 |
|  | ONH_RNFL_TU1 | 67.386 (9.816) | 65.497 (11.403) | 65.911 (11.181) | 0.617 |
|  | ONH_RNFL_TU2 | 95.611 (15.978) | 90.487 (18.528) | 91.602 (16.345) | 0.161 |
|  | ONH_RNFL_ST2 | 127.393 (16.891) | 123.588 (23.673) | 128.330 (21.911) | 0.185 |
|  | ONH_RNFL_ST1 | 128.338 (20.096) | 130.791 (22.317) | 131.070 (20.454) | 0.374 |
|  | ONH_RNFL_SN1 | 108.575 (17.820) | 111.181 (21.812) | 105.459 (19.013) | **0.048** |
|  | ONH_RNFL_SN2 | 103.180 (17.672) | 104.666 (16.568) | 102.220 (16.414) | 0.434 |
|  | ONH_RNFL_NU2 | 83.326 (17.250) | 89.032 (15.744) | 88.128 (17.135) | 0.283 |
|  | ONH_RNFL_NU1 | 66.556 (11.854) | 70.441 (13.769) | 70.883 (12.907) | 0.369 |
|  | ONH_RNFL_NL1 | 63.728 (9.269) | 67.245 (13.201) | 66.284 (10.632) | 0.412 |
|  | ONH_RNFL_NL2 | 76.392 (12.588) | 80.813 (14.470) | 79.564 (13.721) | 0.280 |
|  | ONH_RNFL_IN2 | 100.703 (20.707) | 101.363 (20.061) | 100.149 (17.853) | 0.800 |
|  | ONH_RNFL_IN1 | 123.222 (23.308) | 122.190 (24.833) | 122.757 (23.001) | 0.961 |
|  | ONH_RNFL_IT1 | 140.595 (19.536) | 141.399 (23.259) | 144.331 (19.457) | 0.583 |
|  | ONH_RNFL_IT2 | 117.269 (23.262) | 122.237 (26.183) | 121.354 (22.032) | 0.482 |
|  | ONH_RNFL_TL2 | 72.865 (14.198) | 75.344 (14.347) | 74.801 (13.600) | 0.588 |
|  | ONH_RNFL_TL1 | 56.226 (8.814) | 57.152 (9.303) | 58.247 (10.658) | 0.349 |
| **Macula_3mm**  **Macula_3mm**  **Macula_3mm**  **Macula_3mm**  **Macula_3mm** | Macula_3mm_Thk_ILM_IPL_um_Center_1 | 46.700 (9.850) | 47.754 (9.634) | 47.775 (9.963) | 0.695 |
|  | Macula_3mm_Thk_ILM_IPL_um_T_1minus3 | 100.039 (9.702) | 101.472 (10.617) | 102.793 (13.144) | 0.199 |
|  | Macula_3mm_Thk_ILM_IPL_um_S_1minus3 | 110.539 (11.610) | 111.239 (13.058) | 113.106 (14.588) | 0.242 |
|  | Macula_3mm_Thk_ILM_IPL_um_N_1minus3 | 106.005 (11.528) | 107.306 (12.247) | 108.159 (13.626) | 0.390 |
|  | Macula_3mm_Thk_ILM_IPL_um_I_1minus3 | 110.536 (11.644) | 111.612 (12.678) | 113.236 (12.920) | 0.359 |
|  | Macula_3mm_Thk_ILM_IPL_um_S_Hemi_1minus3 | 106.495 (10.518) | 107.512 (12.138) | 109.005 (13.787) | 0.221 |
|  | Macula_3mm_Thk_ILM_IPL_um_I_Hemi_1minus3 | 107.066 (11.126) | 108.296 (11.764) | 109.624 (12.902) | 0.346 |
|  | Macula_3mm_Thk_ILM_IPL_um_All_1minus3 | 106.791 (10.597) | 107.906 (11.660) | 109.319 (13.105) | 0.233 |
|  | Macula_3mm_Thk_ILM_IPL_um_S_Hemi_field | 101.239 (9.726) | 102.009 (11.485) | 103.478 (12.934) | 0.231 |
|  | Macula_3mm_Thk_ILM_IPL_um_I_Hemi_field | 101.993 (10.521) | 103.217 (11.142) | 104.399 (12.129) | 0.336 |
|  | Macula_3mm_Thk_ILM_IPL_um_All_field | 101.750 (9.856) | 102.725 (11.066) | 104.061 (12.272) | 0.238 |
|  | Macula_3mm_Thk_ILM_RPE_um_Center_1 | 255.009 (28.124) | 259.558 (25.126) | 260.024 (28.012) | 0.766 |
|  | Macula_3mm_Thk_ILM_RPE_um_T_1minus3 | 311.416 (22.782) | 316.753 (17.574) | 317.571 (21.521) | 0.451 |
|  | Macula_3mm_Thk_ILM_RPE_um_S_1minus3 | 321.509 (23.411) | 327.001 (18.236) | 328.978 (22.828) | 0.209 |
|  | Macula_3mm_Thk_ILM_RPE_um_N_1minus3 | 321.659 (23.320) | 328.632 (19.362) | 329.311 (22.112) | 0.217 |
|  | Macula_3mm_Thk_ILM_RPE_um_I_1minus3 | 320.005 (23.903) | 325.237 (19.067) | 326.672 (20.960) | 0.359 |
|  | Macula_3mm_Thk_ILM_RPE_um_S_Hemi_1minus3 | 318.707 (22.713) | 324.653 (17.907) | 325.991 (22.088) | 0.188 |
|  | Macula_3mm_Thk_ILM_RPE_um_I_Hemi_1minus3 | 318.600 (22.905) | 324.145 (18.422) | 325.245 (21.004) | 0.328 |
|  | Macula_3mm_Thk_ILM_RPE_um_All_1minus3 | 318.659 (22.433) | 324.394 (17.780) | 325.625 (21.350) | 0.247 |
|  | Macula_3mm_Thk_ILM_RPE_um_S_Hemi_field | 309.643 (21.982) | 315.237 (17.351) | 316.578 (21.444) | 0.210 |
|  | Macula_3mm_Thk_ILM_RPE_um_I_Hemi_field | 308.841 (21.446) | 313.963 (17.822) | 315.145 (20.223) | 0.303 |
|  | Macula_3mm_Thk_ILM_RPE_um_All_field | 309.332 (21.320) | 314.651 (17.261) | 315.921 (20.577) | 0.235 |
|  | Macula_3mm_Thk_ILM_BRM_um_Center_1 | 262.448 (27.831) | 266.690 (25.185) | 267.255 (28.084) | 0.794 |
|  | Macula_3mm_Thk_ILM_BRM_um_T_1minus3 | 316.318 (22.603) | 321.664 (17.744) | 322.460 (21.129) | 0.502 |
|  | Macula_3mm_Thk_ILM_BRM_um_S_1minus3 | 326.984 (22.256) | 332.209 (18.172) | 334.254 (22.896) | 0.216 |
|  | Macula_3mm_Thk_ILM_BRM_um_N_1minus3 | 327.741 (22.210) | 334.471 (19.530) | 335.097 (21.880) | 0.291 |
|  | Macula_3mm_Thk_ILM_BRM_um_I_1minus3 | 325.359 (22.464) | 330.301 (19.064) | 331.862 (19.949) | 0.412 |
|  | Macula_3mm_Thk_ILM_BRM_um_S_Hemi_1minus3 | 324.236 (21.680) | 329.925 (17.882) | 331.286 (22.091) | 0.233 |
|  | Macula_3mm_Thk_ILM_BRM_um_I_Hemi_1minus3 | 323.957 (21.959) | 329.378 (18.572) | 330.517 (20.196) | 0.417 |
|  | Macula_3mm_Thk_ILM_BRM_um_All_1minus3 | 324.105 (21.425) | 329.659 (17.855) | 330.911 (20.768) | 0.316 |
|  | Macula_3mm_Thk_ILM_BRM_um_S_Hemi_field | 315.307 (20.675) | 320.555 (17.354) | 321.911 (21.407) | 0.255 |
|  | Macula_3mm_Thk_ILM_BRM_um_I_Hemi_field | 314.245 (20.475) | 319.201 (17.920) | 320.489 (19.281) | 0.390 |
|  | Macula_3mm_Thk_ILM_BRM_um_All_field | 314.861 (20.149) | 319.921 (17.315) | 321.279 (19.873) | 0.307 |
|  | Macula_3mm_Thk_RPE_BRM_um_Center_1 | 7.443 (3.378) | 7.124 (2.193) | 7.230 (3.368) | 0.987 |
|  | Macula_3mm_Thk_RPE_BRM_um_T_1minus3 | 4.884 (2.218) | 4.914 (1.901) | 4.885 (2.585) | 0.635 |
|  | Macula_3mm_Thk_RPE_BRM_um_S_1minus3 | 5.477 (2.841) | 5.204 (1.990) | 5.275 (2.163) | 0.993 |
|  | Macula_3mm_Thk_RPE_BRM_um_N_1minus3 | 6.064 (3.376) | 5.845 (3.116) | 5.782 (2.483) | 0.984 |
|  | Macula_3mm_Thk_RPE_BRM_um_I_1minus3 | 5.339 (3.705) | 5.067 (3.639) | 5.180 (6.635) | 0.742 |
|  | Macula_3mm_Thk_RPE_BRM_um_S_Hemi_1minus3 | 5.543 (2.823) | 5.276 (2.050) | 5.292 (1.991) | 0.993 |
|  | Macula_3mm_Thk_RPE_BRM_um_I_Hemi_1minus3 | 5.355 (2.973) | 5.240 (3.075) | 5.272 (4.884) | 0.795 |
|  | Macula_3mm_Thk_RPE_BRM_um_All_1minus3 | 5.441 (2.815) | 5.260 (2.289) | 5.289 (3.116) | 0.924 |
|  | Macula_3mm_Thk_RPE_BRM_um_S_Hemi_field | 5.661 (2.931) | 5.315 (1.906) | 5.336 (1.945) | 0.989 |
|  | Macula_3mm_Thk_RPE_BRM_um_I_Hemi_field | 5.407 (2.868) | 5.237 (2.823) | 5.343 (5.136) | 0.814 |
|  | Macula_3mm_Thk_RPE_BRM_um_All_field | 5.530 (2.835) | 5.271 (2.137) | 5.360 (3.414) | 0.914 |
|  | Macula_3mm_Vol_ILM_IPL_mm3_Center_1 | 0.037 (0.008) | 0.038 (0.008) | 0.038 (0.008) | 0.692 |
|  | Macula_3mm_Vol_ILM_IPL_mm3_T_1minus3 | 0.156 (0.016) | 0.158 (0.017) | 0.160 (0.021) | 0.206 |
|  | Macula_3mm_Vol_ILM_IPL_mm3_S_1minus3 | 0.170 (0.018) | 0.171 (0.021) | 0.174 (0.023) | 0.315 |
|  | Macula_3mm_Vol_ILM_IPL_mm3_N_1minus3 | 0.163 (0.019) | 0.165 (0.020) | 0.167 (0.021) | 0.370 |
|  | Macula_3mm_Vol_ILM_IPL_mm3_I_1minus3 | 0.172 (0.019) | 0.175 (0.020) | 0.176 (0.021) | 0.528 |
|  | Macula_3mm_Vol_ILM_IPL_mm3_S_Hemi_1minus3 | 0.330 (0.033) | 0.332 (0.038) | 0.337 (0.042) | 0.210 |
|  | Macula_3mm_Vol_ILM_IPL_mm3_I_Hemi_1minus3 | 0.332 (0.035) | 0.336 (0.037) | 0.340 (0.040) | 0.377 |
|  | Macula_3mm_Vol_ILM_IPL_mm3_All_1minus3 | 0.662 (0.066) | 0.668 (0.073) | 0.678 (0.080) | 0.228 |
|  | Macula_3mm_Vol_ILM_IPL_mm3_S_Hemi_field | 0.450 (0.049) | 0.448 (0.056) | 0.459 (0.062) | 0.174 |
|  | Macula_3mm_Vol_ILM_IPL_mm3_I_Hemi_field | 0.468 (0.055) | 0.478 (0.054) | 0.479 (0.064) | 0.390 |
|  | Macula_3mm_Vol_ILM_IPL_mm3_All_field | 0.918 (0.089) | 0.927 (0.100) | 0.939 (0.111) | 0.241 |
|  | Macula_3mm_Vol_ILM_RPE_mm3_Center_1 | 0.200 (0.022) | 0.204 (0.020) | 0.204 (0.022) | 0.748 |
|  | Macula_3mm_Vol_ILM_RPE_mm3_T_1minus3 | 0.485 (0.037) | 0.492 (0.032) | 0.495 (0.035) | 0.467 |
|  | Macula_3mm_Vol_ILM_RPE_mm3_S_1minus3 | 0.495 (0.037) | 0.502 (0.032) | 0.506 (0.038) | 0.186 |
|  | Macula_3mm_Vol_ILM_RPE_mm3_N_1minus3 | 0.495 (0.039) | 0.506 (0.035) | 0.510 (0.035) | 0.147 |
|  | Macula_3mm_Vol_ILM_RPE_mm3_I_1minus3 | 0.499 (0.039) | 0.509 (0.030) | 0.509 (0.035) | 0.450 |
|  | Macula_3mm_Vol_ILM_RPE_mm3_S_Hemi_1minus3 | 0.986 (0.070) | 1.001 (0.059) | 1.009 (0.068) | 0.137 |
|  | Macula_3mm_Vol_ILM_RPE_mm3_I_Hemi_1minus3 | 0.988 (0.073) | 1.007 (0.058) | 1.010 (0.066) | 0.327 |
|  | Macula_3mm_Vol_ILM_RPE_mm3_All_1minus3 | 1.975 (0.139) | 2.008 (0.113) | 2.018 (0.130) | 0.215 |
|  | Macula_3mm_Vol_ILM_RPE_mm3_S_Hemi_field | 1.375 (0.111) | 1.385 (0.099) | 1.406 (0.117) | 0.095 |
|  | Macula_3mm_Vol_ILM_RPE_mm3_I_Hemi_field | 1.418 (0.126) | 1.456 (0.100) | 1.446 (0.123) | 0.220 |
|  | Macula_3mm_Vol_ILM_RPE_mm3_All_field | 2.792 (0.192) | 2.841 (0.156) | 2.852 (0.186) | 0.236 |
|  | Macula_3mm_Vol_ILM_BRM_mm3_Center_1 | 0.206 (0.022) | 0.210 (0.020) | 0.210 (0.022) | 0.766 |
|  | Macula_3mm_Vol_ILM_BRM_mm3_T_1minus3 | 0.493 (0.037) | 0.499 (0.032) | 0.502 (0.034) | 0.512 |
|  | Macula_3mm_Vol_ILM_BRM_mm3_S_1minus3 | 0.504 (0.035) | 0.510 (0.032) | 0.514 (0.038) | 0.219 |
|  | Macula_3mm_Vol_ILM_BRM_mm3_N_1minus3 | 0.505 (0.038) | 0.515 (0.035) | 0.519 (0.035) | 0.201 |
|  | Macula_3mm_Vol_ILM_BRM_mm3_I_1minus3 | 0.507 (0.038) | 0.517 (0.030) | 0.517 (0.033) | 0.457 |
|  | Macula_3mm_Vol_ILM_BRM_mm3_S_Hemi_1minus3 | 1.003 (0.067) | 1.018 (0.059) | 1.025 (0.068) | 0.172 |
|  | Macula_3mm_Vol_ILM_BRM_mm3_I_Hemi_1minus3 | 1.005 (0.071) | 1.023 (0.059) | 1.026 (0.064) | 0.412 |
|  | Macula_3mm_Vol_ILM_BRM_mm3_All_1minus3 | 2.009 (0.134) | 2.041 (0.113) | 2.051 (0.126) | 0.274 |
|  | Macula_3mm_Vol_ILM_BRM_mm3_S_Hemi_field | 1.400 (0.107) | 1.408 (0.100) | 1.429 (0.117) | 0.109 |
|  | Macula_3mm_Vol_ILM_BRM_mm3_I_Hemi_field | 1.442 (0.123) | 1.480 (0.101) | 1.471 (0.123) | 0.231 |
|  | Macula_3mm_Vol_ILM_BRM_mm3_All_field | 2.842 (0.182) | 2.888 (0.156) | 2.900 (0.179) | 0.307 |
|  | Macula_3mm_Vol_RPE_BRM_mm3_Center_1 | 0.006 (0.003) | 0.006 (0.002) | 0.006 (0.003) | 0.971 |
|  | Macula_3mm_Vol_RPE_BRM_mm3_T_1minus3 | 0.008 (0.003) | 0.008 (0.003) | 0.008 (0.004) | 0.665 |
|  | Macula_3mm_Vol_RPE_BRM_mm3_S_1minus3 | 0.008 (0.004) | 0.008 (0.003) | 0.008 (0.003) | 0.984 |
|  | Macula_3mm_Vol_RPE_BRM_mm3_N_1minus3 | 0.009 (0.005) | 0.009 (0.005) | 0.009 (0.004) | 0.968 |
|  | Macula_3mm_Vol_RPE_BRM_mm3_I_1minus3 | 0.008 (0.006) | 0.008 (0.006) | 0.008 (0.010) | 0.865 |
|  | Macula_3mm_Vol_RPE_BRM_mm3_S_Hemi_1minus3 | 0.017 (0.009) | 0.016 (0.006) | 0.016 (0.006) | 0.983 |
|  | Macula_3mm_Vol_RPE_BRM_mm3_I_Hemi_1minus3 | 0.017 (0.009) | 0.016 (0.010) | 0.016 (0.015) | 0.796 |
|  | Macula_3mm_Vol_RPE_BRM_mm3_All_1minus3 | 0.034 (0.018) | 0.033 (0.014) | 0.033 (0.019) | 0.959 |
|  | Macula_3mm_Vol_RPE_BRM_mm3_S_Hemi_field | 0.025 (0.013) | 0.023 (0.009) | 0.024 (0.008) | 0.983 |
|  | Macula_3mm_Vol_RPE_BRM_mm3_I_Hemi_field | 0.025 (0.013) | 0.024 (0.013) | 0.025 (0.026) | 0.691 |
|  | Macula_3mm_Vol_RPE_BRM_mm3_All_field | 0.050 (0.026) | 0.048 (0.019) | 0.048 (0.031) | 0.888 |
| **Retina3DflowDensity**  **Retina3DflowDensity**  **Retina3DflowDensity** | SVC_L1_DensityOfWhole_Image | 44.017 (3.699) | 45.528 (3.786) | 46.716 (4.225) | **<0.001** |
|  | SVC_L1_Whole_Image_S_Hemi | 44.095 (3.836) | 45.509 (3.830) | 46.659 (4.304) | **<0.001** |
|  | SVC_L1_Whole_Image_I_Hemi | 43.942 (3.870) | 45.538 (3.928) | 46.764 (4.337) | **<0.001** |
|  | SVC_L1_Whole_ETDRS | 43.673 (3.595) | 45.169 (3.762) | 46.310 (4.200) | **<0.001** |
|  | SVC_L1_Fovea | 18.801 (6.883) | 19.953 (6.524) | 21.204 (6.591) | 0.057 |
|  | SVC_L1_ParaFovea | 46.830 (3.563) | 48.367 (3.787) | 49.492 (4.341) | **<0.001** |
|  | SVC_L1_Para_S_Hemi | 46.935 (3.662) | 48.299 (3.921) | 49.431 (4.488) | **<0.001** |
|  | SVC_L1_Para_I_Hemi | 46.724 (3.870) | 48.422 (3.902) | 49.553 (4.439) | **<0.001** |
|  | SVC_L1_Para_T | 45.879 (4.221) | 47.215 (3.993) | 48.496 (4.557) | **<0.001** |
|  | SVC_L1_Para_S | 47.813 (3.899) | 49.392 (4.315) | 50.424 (4.668) | **<0.001** |
|  | SVC_L1_Para_N | 46.223 (3.690) | 47.645 (3.883) | 48.714 (4.679) | **<0.001** |
|  | SVC_L1_Para_I | 47.420 (4.667) | 49.193 (4.524) | 50.348 (4.637) | **<0.001** |
|  | SVC_L1_G11 | 45.834 (5.241) | 47.263 (4.858) | 49.006 (4.809) | **<0.001** |
|  | SVC_L1_G12 | 48.247 (3.790) | 49.633 (4.215) | 50.527 (4.818) | **0.001** |
|  | SVC_L1_G13 | 46.247 (4.715) | 48.020 (4.459) | 48.914 (5.196) | **0.002** |
|  | SVC_L1_G21 | 45.426 (4.011) | 47.166 (4.120) | 48.091 (4.633) | **<0.001** |
|  | SVC_L1_G22 | 24.417 (5.799) | 25.339 (6.035) | 26.657 (5.959) | **0.036** |
|  | SVC_L1_G23 | 46.233 (3.810) | 47.192 (4.161) | 48.294 (5.175) | **<0.001** |
|  | SVC_L1_G31 | 45.915 (4.950) | 47.622 (4.979) | 48.779 (5.061) | **0.001** |
|  | SVC_L1_G32 | 47.960 (4.826) | 49.445 (4.804) | 50.735 (4.825) | **<0.001** |
|  | SVC_L1_G33 | 45.748 (5.223) | 47.782 (5.136) | 49.299 (5.152) | **<0.001** |
|  | DVC_L2_DensityOfWhole_Image | 46.607 (3.868) | 48.086 (4.256) | 48.556 (4.421) | **0.009** |
|  | DVC_L2_Whole_Image_S_Hemi | 47.023 (3.730) | 48.282 (4.223) | 48.715 (4.513) | **0.024** |
|  | DVC_L2_Whole_Image_I_Hemi | 46.199 (4.245) | 47.893 (4.482) | 48.400 (4.522) | **0.007** |
|  | DVC_L2_Whole_ETDRS | 46.710 (3.553) | 47.955 (3.973) | 48.405 (4.270) | **0.015** |
|  | DVC_L2_Fovea | 30.408 (7.672) | 31.272 (6.811) | 31.426 (6.640) | 0.676 |
|  | DVC_L2_ParaFovea | 48.780 (3.972) | 50.068 (4.294) | 50.556 (4.563) | **0.018** |
|  | DVC_L2_Para_S_Hemi | 49.255 (3.722) | 50.245 (4.269) | 50.624 (4.636) | 0.067 |
|  | DVC_L2_Para_I_Hemi | 48.305 (4.504) | 49.864 (4.587) | 50.487 (4.669) | **0.009** |
|  | DVC_L2_Para_T | 49.450 (3.686) | 50.230 (4.318) | 50.831 (4.471) | **0.036** |
|  | DVC_L2_Para_S | 49.468 (4.173) | 50.262 (4.627) | 50.693 (5.111) | 0.172 |
|  | DVC_L2_Para_N | 48.802 (4.392) | 50.271 (4.453) | 50.536 (4.517) | **0.046** |
|  | DVC_L2_Para_I | 47.426 (5.602) | 49.514 (5.128) | 50.157 (5.191) | **0.008** |
|  | DVC_L2_G11 | 47.398 (5.434) | 49.211 (5.767) | 49.948 (5.399) | **0.020** |
|  | DVC_L2_G12 | 49.563 (4.608) | 50.611 (4.652) | 50.863 (5.044) | 0.217 |
|  | DVC_L2_G13 | 47.763 (5.394) | 49.290 (5.348) | 49.730 (5.757) | 0.077 |
|  | DVC_L2_G21 | 49.407 (3.542) | 50.560 (4.165) | 50.827 (4.365) | **0.040** |
|  | DVC_L2_G22 | 35.210 (5.897) | 35.677 (5.714) | 35.834 (5.477) | 0.805 |
|  | DVC_L2_G23 | 49.585 (3.808) | 50.789 (4.212) | 50.969 (4.808) | 0.051 |
|  | DVC_L2_G31 | 46.762 (5.374) | 48.643 (5.786) | 49.144 (5.901) | **0.023** |
|  | DVC_L2_G32 | 47.531 (6.036) | 49.858 (5.596) | 50.363 (5.434) | **0.012** |
|  | DVC_L2_G33 | 46.161 (6.051) | 48.115 (5.967) | 49.230 (5.603) | **0.006** |
|  | FAZ_Area | 0.301 (0.143) | 0.301 (0.118) | 0.305 (0.110) | 0.969 |
|  | Perimeter | 2.186 (0.608) | 2.198 (0.464) | 2.224 (0.429) | 0.840 |
|  | AcircularityIndex | 1.162 (0.059) | 1.152 (0.051) | 1.156 (0.054) | 0.419 |
|  | FD_300_Area_Density | 46.997 (5.300) | 48.529 (3.987) | 49.213 (4.512) | **0.008** |
|  | FD_300_Length_Density | 14.771 (2.218) | 15.873 (2.179) | 16.386 (2.199) | **<0.001** |
| **Retina Map**  **Retina Map**  **Retina Map**  **Retina Map** | FoveaInnRet_Thickness | 71.296 (15.203) | 73.329 (13.621) | 73.595 (13.887) | 0.597 |
|  | InnRet_Thk_ParaFovea | 122.455 (13.443) | 125.942 (11.198) | 127.667 (11.436) | **0.048** |
|  | InnRet_Thk_Para_S_Hemisphere | 122.136 (13.264) | 126.173 (12.434) | 127.667 (12.042) | **0.045** |
|  | InnRet_Thk_Para_I_Hemisphere | 122.545 (13.961) | 125.813 (11.330) | 127.701 (11.653) | 0.054 |
|  | InnRet_Thk_Para_Tempo | 115.568 (14.639) | 119.273 (10.670) | 120.684 (11.497) | 0.140 |
|  | InnRet_Thk_Para_Superior | 124.227 (14.791) | 128.532 (13.438) | 129.931 (12.511) | 0.096 |
|  | InnRet_Thk_Para_Nasal | 124.864 (15.490) | 128.942 (13.871) | 130.759 (13.305) | **0.038** |
|  | InnRet_Thk_Para_Inferior | 124.341 (15.305) | 127.273 (11.918) | 129.431 (12.281) | 0.063 |
|  | InnRet_Thk_PeriFovea | 107.114 (9.862) | 109.576 (9.720) | 111.006 (9.557) | **0.045** |
|  | InnRet_Thk_Peri_S_Hemisphere | 107.568 (10.144) | 110.309 (9.708) | 111.414 (9.780) | 0.081 |
|  | InnRet_Thk_Peri_I_Hemisphere | 106.682 (10.496) | 108.741 (10.617) | 110.782 (11.034) | **0.047** |
|  | InnRet_Thk_Peri_Tempo | 101.841 (12.378) | 103.971 (12.051) | 105.121 (9.858) | 0.263 |
|  | InnRet_Thk_Peri_Superior | 106.682 (11.328) | 109.187 (10.128) | 110.356 (9.902) | 0.130 |
|  | InnRet_Thk_Peri_Nasal | 115.886 (10.708) | 118.705 (10.970) | 120.195 (11.021) | 0.058 |
|  | InnRet_Thk_Peri_Inferior | 104.295 (12.489) | 106.245 (10.907) | 109.103 (17.555) | 0.118 |
|  | FoveaInnRet_Volumn | 0.056 (0.012) | 0.057 (0.011) | 0.058 (0.011) | 0.420 |
|  | InnRet_Vol_ParaFovea | 0.763 (0.083) | 0.790 (0.078) | 0.805 (0.072) | **0.005** |
|  | InnRet_Vol_Para_S_Hemisphere | 0.381 (0.042) | 0.395 (0.042) | 0.402 (0.038) | **0.006** |
|  | InnRet_Vol_Para_I_Hemisphere | 0.382 (0.044) | 0.395 (0.041) | 0.402 (0.037) | **0.008** |
|  | InnRet_Vol_Para_Tempo | 0.177 (0.026) | 0.187 (0.019) | 0.191 (0.020) | **0.007** |
|  | InnRet_Vol_Para_Superior | 0.193 (0.024) | 0.201 (0.023) | 0.204 (0.020) | **0.012** |
|  | InnRet_Vol_Para_Nasal | 0.199 (0.026) | 0.202 (0.024) | 0.205 (0.023) | 0.150 |
|  | InnRet_Vol_Para_Inferior | 0.192 (0.027) | 0.200 (0.019) | 0.203 (0.020) | **0.013** |
|  | InnRet_Vol_PeriFovea | 1.322 (0.158) | 1.375 (0.130) | 1.394 (0.124) | **0.010** |
|  | InnRet_Vol_Peri_S_Hemisphere | 0.664 (0.078) | 0.693 (0.069) | 0.700 (0.063) | **0.017** |
|  | InnRet_Vol_Peri_I_Hemisphere | 0.658 (0.089) | 0.682 (0.072) | 0.694 (0.068) | **0.022** |
|  | InnRet_Vol_Peri_Tempo | 0.316 (0.045) | 0.327 (0.041) | 0.330 (0.041) | 0.079 |
|  | InnRet_Vol_Peri_Superior | 0.327 (0.046) | 0.343 (0.039) | 0.348 (0.032) | **0.020** |
|  | InnRet_Vol_Peri_Nasal | 0.358 (0.049) | 0.373 (0.039) | 0.378 (0.035) | **0.039** |
|  | InnRet_Vol_Peri_Inferior | 0.321 (0.053) | 0.333 (0.037) | 0.338 (0.039) | 0.076 |
|  | FoveaFullRet_Thickness | 257.807 (26.223) | 260.574 (25.304) | 261.223 (27.713) | 0.896 |
|  | FullRet_Thk_ParaFovea | 312.705 (21.853) | 317.525 (18.699) | 319.552 (20.339) | 0.308 |
|  | FullRet_Thk_Para_S_Hemisphere | 312.705 (21.095) | 317.209 (19.066) | 319.460 (21.524) | 0.269 |
|  | FullRet_Thk_Para_I_Hemisphere | 312.682 (23.116) | 317.878 (19.331) | 319.138 (19.816) | 0.478 |
|  | FullRet_Thk_Para_Tempo | 303.932 (27.136) | 310.806 (18.811) | 312.075 (21.149) | 0.420 |
|  | FullRet_Thk_Para_Superior | 315.205 (21.010) | 318.784 (19.129) | 321.322 (22.011) | 0.249 |
|  | FullRet_Thk_Para_Nasal | 317.182 (21.256) | 322.547 (20.374) | 323.609 (21.666) | 0.292 |
|  | FullRet_Thk_Para_Inferior | 313.977 (24.070) | 317.935 (19.724) | 319.569 (19.620) | 0.671 |
|  | FullRet_Thk_PeriFovea | 279.500 (20.228) | 282.727 (17.227) | 284.793 (17.806) | 0.410 |
|  | FullRet_Thk_Peri_S_Hemisphere | 282.636 (19.821) | 285.259 (18.013) | 287.443 (18.922) | 0.370 |
|  | FullRet_Thk_Peri_I_Hemisphere | 276.818 (21.726) | 279.727 (17.055) | 282.178 (17.667) | 0.465 |
|  | FullRet_Thk_Peri_Tempo | 268.568 (30.895) | 274.273 (17.674) | 276.270 (19.202) | 0.309 |
|  | FullRet_Thk_Peri_Superior | 282.295 (20.533) | 283.921 (18.794) | 285.937 (20.287) | 0.441 |
|  | FullRet_Thk_Peri_Nasal | 295.386 (16.073) | 297.410 (19.172) | 299.874 (19.439) | 0.327 |
|  | FullRet_Thk_Peri_Inferior | 271.864 (22.575) | 273.856 (17.276) | 276.454 (18.524) | 0.535 |
|  | FoveaFullRet_Volumn | 0.202 (0.021) | 0.204 (0.020) | 0.206 (0.023) | 0.472 |
|  | FullRet_Vol_ParaFovea | 1.960 (0.139) | 1.990 (0.120) | 2.010 (0.129) | 0.140 |
|  | FullRet_Vol_Para_S_Hemisphere | 0.982 (0.068) | 0.993 (0.063) | 1.006 (0.068) | 0.123 |
|  | FullRet_Vol_Para_I_Hemisphere | 0.978 (0.074) | 0.997 (0.061) | 1.004 (0.063) | 0.180 |
|  | FullRet_Vol_Para_Tempo | 0.472 (0.046) | 0.487 (0.033) | 0.495 (0.041) | **0.046** |
|  | FullRet_Vol_Para_Superior | 0.496 (0.035) | 0.499 (0.033) | 0.505 (0.035) | 0.227 |
|  | FullRet_Vol_Para_Nasal | 0.499 (0.034) | 0.505 (0.032) | 0.508 (0.034) | 0.487 |
|  | FullRet_Vol_Para_Inferior | 0.492 (0.038) | 0.499 (0.031) | 0.502 (0.030) | 0.545 |
|  | FullRet_Vol_PeriFovea | 3.505 (0.253) | 3.537 (0.221) | 3.582 (0.226) | 0.139 |
|  | FullRet_Vol_Peri_S_Hemisphere | 1.765 (0.126) | 1.787 (0.120) | 1.810 (0.122) | 0.079 |
|  | FullRet_Vol_Peri_I_Hemisphere | 1.738 (0.137) | 1.749 (0.111) | 1.773 (0.115) | 0.168 |
|  | FullRet_Vol_Peri_Tempo | 0.838 (0.107) | 0.861 (0.059) | 0.875 (0.063) | 0.060 |
|  | FullRet_Vol_Peri_Superior | 0.881 (0.080) | 0.889 (0.067) | 0.900 (0.071) | 0.201 |
|  | FullRet_Vol_Peri_Nasal | 0.926 (0.057) | 0.932 (0.066) | 0.942 (0.068) | 0.270 |
|  | FullRet_Vol_Peri_Inferior | 0.857 (0.075) | 0.855 (0.060) | 0.865 (0.067) | 0.257 |
|  | FoveaRPE_Elevation_Height | -2.397 (15.025) | 0.324 (15.446) | 3.759 (22.234) | 0.277 |
|  | RPE_Elev_ParaFovea_Tempo | 1.736 (6.306) | 3.140 (12.488) | 4.347 (15.275) | 0.694 |
|  | RPE_Elev_Para_Superior | -0.448 (9.830) | 0.842 (11.582) | 1.920 (13.003) | 0.270 |
|  | RPE_Elev_Para_Nasal | 2.805 (8.669) | 5.773 (15.419) | 6.561 (18.218) | 0.403 |
|  | RPE_Elev_Para_Inferior | 1.314 (11.739) | 2.577 (10.748) | 5.017 (16.439) | **0.031** |
|  | RPE_Elev_PeriFovea_Tempo | 2.207 (5.387) | 6.371 (44.070) | 1.833 (6.366) | 0.184 |
|  | RPE_Elev_Peri_Superior | -0.225 (7.774) | 0.133 (4.650) | 1.280 (12.854) | 0.600 |
|  | RPE_Elev_Peri_Nasal | 1.943 (5.444) | 5.622 (12.791) | 5.163 (13.117) | 0.104 |
|  | RPE_Elev_Peri_Inferior | 1.216 (8.007) | 0.701 (7.458) | 1.047 (9.511) | 0.606 |

# Details of Parameters

GCC related parameters investigates Average, Superior (s), Inferior (I), I subtracted from S (S_I) thicknesses in the following layers definitions: 1) $Inner\_Retina$ (InnRet: ILM to 10 μm below OPL (1)), 2) $Full\_Retina$ (FullRet: ILM to RPE/BRM complex (1)), and 3) $Outer\_Retina$ (OutRet:10 μm below OPL to RPE/BRM complex (2) (3)). Additionally, global loss volume (GLV), focal loss volume (FLV), and root mean square (RMS) (4).

ONH parameters were categorized into 1) optic disc analysis and 2) peripapillary RNFL (pRNFL) thickness. Parameters based on optic disc analysis: optic disc area/volume (ONH_DiscArea, ONH_Disc_Volume), cup area/volume (ONH_CupArea, ONH_CupVolume), rim area/volume (ONH_RimArea, ONH_RimVolume), and cup-to-disc (area: ONH_Area_C_D_ratio, horizontal: ONH_H_C_D_ratio, vertical: ONH_V_C_D_ratio) ratios. Parameters based on pRNFL thickness: hemisphere S (S-Hemi) and hemisphere I (I-Hemi), and average pRNFL thickness (5). Additionally, other pRNFL regions included quadrants-based Superior (S), Inferior (I), Temporal (T), Nasal (N), as well as supertemporal (ST), superonasal (SN), inferotemporal (IT), inferonasal (IN), nasal upper (NU), nasal lower (NL), temporal upper (TU), and temporal lower (TL).

Macula_3mm parameters target retinal thickness/volume changes in various quadrants and layers (ILM to IPL, ILM to RPE, ILM to BRM, and RPE to BRM). The thickness/volume analyses for distinct layers definitions include $Center\_1$ (1mm foveal ring), quadrants (S, I, T, N) in the 3mm ring excluding 1mm (1minus3), S and I hemispheres in 1minus3, and combined hemispheres (All_1minus3). Additionally, the thickness/volume analyses for distinct layers definitions include S and I hemispheres field (including $Center\_1$), combined hemispheres field (All_field).

The retina map measures retinal OCT thickness (μm) and volume ($mm^{3}$) around the fovea using 1 mm, 3 mm, and 5 mm circles. The foveal thickness/volume was denoted by $Fovea$, whilst quadrants of ($S_{3}, I_{3}, T_{3}, N_{3}$) and ($S_{5}, I_{5}, T_{5}, N_{5}$) corresponded to parafovea (Para) and perifovea (Peri), respectively. Also, hemispheres $S\_Hemi_{3}$ (Para_S_Hemisphere) and $I\_Hemi_{3}$ (Para_I_Hemisphere) represented 3 mm rings, while $S\_Hemi_{5}$ (Peri_S_Hemisphere) and $I\_Hemi_{5}$ (Peri_I_Hemisphere) indicated 5 mm rings. Additionally, $parafovea$ was the combined $S\_Hemi_{3}$ and $I\_Hemi_{3}$, whilst $perifovea$was the combined $S\_Hemi_{5}$ and $I\_Hemi_{5}$. The retina map ($\mu m/mm^{3}$) was calculated in $Inner\_Retina$ and $Full\_Retina$ layers, similarly to GCC parameters, as well as in RPE thickness (Elevation).

Retina3DFlowDensity investigates retinal VD changes using quadrants (S, I, T, N), hemispheres (S and I), not following Early Treatment of Diabetic Retinopathy Study (ETDRS) ($Whole\_Image$), both hemispheres combined (Whole_ETDRS), $Whole Image$ in $S\_Hemi_{3}$ and$I\_Hemi_{3}$, the 3x3 grid G, and $FD\_300$ ($Area\_Density$ and $Length\_Density$). Other Retina3DFlowDensity parameters include $Fovea$, $FAZ\_Area$, $FAZ\_Perim$ (Perimeter) (6), and $AcirIndx$(AcircularityIndex) (7). Importantly, VD was computed for various retinal layers including A) superficial vascular complex (SVC) from ILM to 10 μm above the IPL, B) deep vascular complex (DVC) from 10 μm above IPL to 10 μm below OPL, C) inner vascular complex (IVC) from ILM to 10 μm below OPL.

# References

1. Hanumunthadu D, Keane PA, Balaskas K, Dubis AM, Kalitzeos A, Michaelides M, et al. Agreement between spectral-domain and swept-source optical coherence tomography retinal thickness measurements in macular and retinal disease. Ophthalmology and therapy. 2021;10:913-22.

2. Venkatesh R, Sinha S, Gangadharaiah D, Gadde SG, Mohan A, Shetty R, et al. Retinal structural-vascular-functional relationship using optical coherence tomography and optical coherence tomography–angiography in myopia. Eye and Vision. 2019;6(1):1-12.

3. Ye J, Wang M, Shen M, Huang S, Xue A, Lin J, et al. Deep retinal capillary plexus decreasing correlated with the outer retinal layer alteration and visual acuity impairment in pathological myopia. Investigative ophthalmology & visual science. 2020;61(4):45-.

4. Rao HL, Zangwill LM, Weinreb RN, Sample PA, Alencar LM, Medeiros FA. Comparison of different spectral domain optical coherence tomography scanning areas for glaucoma diagnosis. Ophthalmology. 2010;117(9):1692-9. e1.

5. González-García AO, Vizzeri G, Bowd C, Medeiros FA, Zangwill LM, Weinreb RN. Reproducibility of RTVue retinal nerve fiber layer thickness and optic disc measurements and agreement with Stratus optical coherence tomography measurements. American Journal of Ophthalmology. 2009;147(6):1067-74. e1.

6. Mo S, Krawitz B, Efstathiadis E, Geyman L, Weitz R, Chui TY, et al. Imaging foveal microvasculature: optical coherence tomography angiography versus adaptive optics scanning light ophthalmoscope fluorescein angiography. Investigative ophthalmology & visual science. 2016;57(9):OCT130-OCT40.

7. Tam J, Dhamdhere KP, Tiruveedhula P, Manzanera S, Barez S, Bearse MA, et al. Disruption of the retinal parafoveal capillary network in type 2 diabetes before the onset of diabetic retinopathy. Investigative Ophthalmology & Visual Science. 2011;52(12):9257-66.
