# Supplementary material for: Retinal microvascular density and inner thickness in Alzheimer’s disease and mild cognitive impairment
Source: Front Aging Neurosci. 2025 Feb 28;17:1477008. doi: 10.3389/fnagi.2025.1477008 (PMC11906703; doi:10.3389/fnagi.2025.1477008)
Supplement: Supplementary file 2 [file Table_2.DOCX]

Supplementary Material B

# POST-Hoc Results

This table presents a comprehensive POST-Hoc analysis results which includes: 1) the parameters that were statistically significant in the initial statistical plan, 2) the parameters that were insignificant in the initial statistics analysis. We preferred to document everything, since a few parameters were significant only in certain comparisons; however, did not qualify to be significant in all post-hoc contrasts. The results are presented in $mean (std)$ and arranged based on the various groups of parameters. Additionally, the statistically significant parameters have their p-value in **Bold** font. Importantly, the detailed description of all parameters’ names could be found under “Details of Parameters” section.

Keywords:

1. Normal distribution or not (NormOrNot), 0/1 means normal/non-normal distribution
2. Homogeneity of variance (HOV), 0/1 means unsatisfied/satisfied condition

| Parameter | Group#1 | Group#2 | NormOrNot | HOV | Post-HOC Test | p-value |
| --- | --- | --- | --- | --- | --- | --- |
| GCC_InnRet_Average | Dem | MCI | 0 | 0 | Mann-Whitney U | 0.740 |
| GCC_InnRet_Average | Dem | HCs | 0 | 0 | Mann-Whitney U | 0.582 |
| GCC_InnRet_Average | MCI | HCs | 0 | 0 | Mann-Whitney U | 0.244 |
| GCC_InnRet_Superior_Avg | Dem | MCI | 0 | 0 | Mann-Whitney U | 0.504 |
| GCC_InnRet_Superior_Avg | Dem | HCs | 0 | 0 | Mann-Whitney U | 0.863 |
| GCC_InnRet_Superior_Avg | MCI | HCs | 0 | 0 | Mann-Whitney U | 0.239 |
| GCC InnRet_Inferior_Avg | Dem | MCI | 0 | 0 | Mann-Whitney U | 0.828 |
| GCC InnRet_Inferior_Avg | Dem | HCs | 0 | 0 | Mann-Whitney U | 0.393 |
| GCC InnRet_Inferior_Avg | MCI | HCs | 0 | 0 | Mann-Whitney U | 0.336 |
| GCC InnRet_S_I_Avg | Dem | MCI | 0 | 0 | Mann-Whitney U | **0.039** |
| GCC InnRet_S_I_Avg | Dem | HCs | 0 | 0 | Mann-Whitney U | 0.110 |
| GCC InnRet_S_I_Avg | MCI | HCs | 0 | 0 | Mann-Whitney U | 0.399 |
| GCC_FLV | Dem | MCI | 0 | 0 | Mann-Whitney U | 0.381 |
| GCC_FLV | Dem | HCs | 0 | 0 | Mann-Whitney U | 0.050 |
| GCC_FLV | MCI | HCs | 0 | 0 | Mann-Whitney U | 0.103 |
| GCC_GLV | Dem | MCI | 0 | 0 | Mann-Whitney U | 0.846 |
| GCC_GLV | Dem | HCs | 0 | 0 | Mann-Whitney U | 0.489 |
| GCC_GLV | MCI | HCs | 0 | 0 | Mann-Whitney U | 0.216 |
| GCC_RMS | Dem | MCI | 0 | 0 | Mann-Whitney U | 0.327 |
| GCC_RMS | Dem | HCs | 0 | 0 | Mann-Whitney U | 0.102 |
| GCC_RMS | MCI | HCs | 0 | 0 | Mann-Whitney U | 0.370 |
| GCC_FullRet_Average | Dem | MCI | 0 | 0 | Mann-Whitney U | 0.498 |
| GCC_FullRet_Average | Dem | HCs | 0 | 0 | Mann-Whitney U | 0.356 |
| GCC_FullRet_Average | MCI | HCs | 0 | 0 | Mann-Whitney U | 0.724 |
| GCC_FullRet_Superior_Avg | Dem | MCI | 0 | 0 | Mann-Whitney U | 0.329 |
| GCC_FullRet_Superior_Avg | Dem | HCs | 0 | 0 | Mann-Whitney U | 0.759 |
| GCC_FullRet_Superior_Avg | MCI | HCs | 0 | 0 | Mann-Whitney U | **0.047** |
| GCC_FullRet_Inferior_Avg | Dem | MCI | 0 | 0 | Mann-Whitney U | 0.973 |
| GCC_FullRet_Inferior_Avg | Dem | HCs | 0 | 0 | Mann-Whitney U | 0.634 |
| GCC_FullRet_Inferior_Avg | MCI | HCs | 0 | 0 | Mann-Whitney U | 0.483 |
| GCC_FullRet_S_I_Avg | Dem | MCI | 0 | 0 | Mann-Whitney U | 0.090 |
| GCC_FullRet_S_I_Avg | Dem | HCs | 0 | 0 | Mann-Whitney U | 0.125 |
| GCC_FullRet_S_I_Avg | MCI | HCs | 0 | 0 | Mann-Whitney U | 0.628 |
| GCC_OutRet_Average | Dem | MCI | 0 | 0 | Mann-Whitney U | 0.600 |
| GCC_OutRet_Average | Dem | HCs | 0 | 0 | Mann-Whitney U | 0.781 |
| GCC_OutRet_Average | MCI | HCs | 0 | 0 | Mann-Whitney U | 0.689 |
| GCC_OutRet_Superior_Avg | Dem | MCI | 0 | 0 | Mann-Whitney U | 0.329 |
| GCC_OutRet_Superior_Avg | Dem | HCs | 0 | 0 | Mann-Whitney U | 0.734 |
| GCC_OutRet_Superior_Avg | MCI | HCs | 0 | 0 | Mann-Whitney U | 0.322 |
| GCC_OutRet_Inferior_Avg | Dem | MCI | 0 | 0 | Mann-Whitney U | 0.774 |
| GCC_OutRet_Inferior_Avg | Dem | HCs | 0 | 0 | Mann-Whitney U | 0.908 |
| GCC_OutRet_Inferior_Avg | MCI | HCs | 0 | 0 | Mann-Whitney U | 0.486 |
| GCC_OutRet_S_I_Avg | Dem | MCI | 0 | 0 | Mann-Whitney U | 0.056 |
| GCC_OutRet_S_I_Avg | Dem | HCs | 0 | 0 | Mann-Whitney U | 0.221 |
| GCC_OutRet_S_I_Avg | MCI | HCs | 0 | 0 | Mann-Whitney U | 0.230 |
| ONH_DiscArea | Dem | MCI | 0 | 0 | Mann-Whitney U | 0.526 |
| ONH_DiscArea | Dem | HCs | 0 | 0 | Mann-Whitney U | 0.510 |
| ONH_DiscArea | MCI | HCs | 0 | 0 | Mann-Whitney U | 0.996 |
| ONH_Area_C_D_ratio | Dem | MCI | 0 | 0 | Mann-Whitney U | 0.899 |
| ONH_Area_C_D_ratio | Dem | HCs | 0 | 0 | Mann-Whitney U | 0.274 |
| ONH_Area_C_D_ratio | MCI | HCs | 0 | 0 | Mann-Whitney U | 0.199 |
| ONH_H_C_D_ratio | Dem | MCI | 0 | 0 | Mann-Whitney U | 0.765 |
| ONH_H_C_D_ratio | Dem | HCs | 0 | 0 | Mann-Whitney U | 0.114 |
| ONH_H_C_D_ratio | MCI | HCs | 0 | 0 | Mann-Whitney U | 0.104 |
| ONH_V_C_D_ratio | Dem | MCI | 0 | 0 | Mann-Whitney U | 0.456 |
| ONH_V_C_D_ratio | Dem | HCs | 0 | 0 | Mann-Whitney U | 0.133 |
| ONH_V_C_D_ratio | MCI | HCs | 0 | 0 | Mann-Whitney U | 0.324 |
| ONH_CupArea | Dem | MCI | 0 | 0 | Mann-Whitney U | 0.982 |
| ONH_CupArea | Dem | HCs | 0 | 0 | Mann-Whitney U | 0.427 |
| ONH_CupArea | MCI | HCs | 0 | 0 | Mann-Whitney U | 0.267 |
| ONH_RimArea | Dem | MCI | 0 | 0 | Mann-Whitney U | 0.078 |
| ONH_RimArea | Dem | HCs | 0 | 0 | Mann-Whitney U | **0.025** |
| ONH_RimArea | MCI | HCs | 0 | 0 | Mann-Whitney U | 0.540 |
| ONH_RimVolume | Dem | MCI | 0 | 0 | Mann-Whitney U | 0.208 |
| ONH_RimVolume | Dem | HCs | 0 | 0 | Mann-Whitney U | 0.154 |
| ONH_RimVolume | MCI | HCs | 0 | 0 | Mann-Whitney U | 0.963 |
| ONH_Disc_Volume | Dem | MCI | 0 | 0 | Mann-Whitney U | 0.099 |
| ONH_Disc_Volume | Dem | HCs | 0 | 0 | Mann-Whitney U | **0.020** |
| ONH_Disc_Volume | MCI | HCs | 0 | 0 | Mann-Whitney U | 0.484 |
| ONH_CupVolume | Dem | MCI | 0 | 0 | Mann-Whitney U | 0.192 |
| ONH_CupVolume | Dem | HCs | 0 | 0 | Mann-Whitney U | **0.016** |
| ONH_CupVolume | MCI | HCs | 0 | 0 | Mann-Whitney U | 0.175 |
| ONH_Avg_RNFL | Dem | MCI | 0 | 0 | Mann-Whitney U | 0.426 |
| ONH_Avg_RNFL | Dem | HCs | 0 | 0 | Mann-Whitney U | 0.349 |
| ONH_Avg_RNFL | MCI | HCs | 0 | 0 | Mann-Whitney U | 0.808 |
| ONH_RNFL_S-Hemi | Dem | MCI | 0 | 0 | Mann-Whitney U | 0.813 |
| ONH_RNFL_S-Hemi | Dem | HCs | 0 | 0 | Mann-Whitney U | 0.840 |
| ONH_RNFL_S-Hemi | MCI | HCs | 0 | 0 | Mann-Whitney U | 0.945 |
| ONH_RNFL_I-Hemi | Dem | MCI | 0 | 0 | Mann-Whitney U | 0.511 |
| ONH_RNFL_I-Hemi | Dem | HCs | 0 | 0 | Mann-Whitney U | 0.402 |
| ONH_RNFL_I-Hemi | MCI | HCs | 0 | 0 | Mann-Whitney U | 0.914 |
| ONH_RNFL_T | Dem | MCI | 0 | 0 | Mann-Whitney U | 0.444 |
| ONH_RNFL_T | Dem | HCs | 0 | 0 | Mann-Whitney U | 0.621 |
| ONH_RNFL_T | MCI | HCs | 0 | 0 | Mann-Whitney U | 0.662 |
| ONH_RNFL_S | Dem | MCI | 0 | 0 | Mann-Whitney U | 0.934 |
| ONH_RNFL_S | Dem | HCs | 0 | 0 | Mann-Whitney U | 0.997 |
| ONH_RNFL_S | MCI | HCs | 0 | 0 | Mann-Whitney U | 0.987 |
| ONH_RNFL_N | Dem | MCI | 0 | 0 | Mann-Whitney U | 0.175 |
| ONH_RNFL_N | Dem | HCs | 0 | 0 | Mann-Whitney U | 0.166 |
| ONH_RNFL_N | MCI | HCs | 0 | 0 | Mann-Whitney U | 0.908 |
| ONH_RNFL_I | Dem | MCI | 0 | 0 | Mann-Whitney U | 0.610 |
| ONH_RNFL_I | Dem | HCs | 0 | 0 | Mann-Whitney U | 0.593 |
| ONH_RNFL_I | MCI | HCs | 0 | 0 | Mann-Whitney U | 0.986 |
| ONH_RNFL_TU | Dem | MCI | 0 | 0 | Mann-Whitney U | 0.186 |
| ONH_RNFL_TU | Dem | HCs | 0 | 0 | Mann-Whitney U | 0.259 |
| ONH_RNFL_TU | MCI | HCs | 0 | 0 | Mann-Whitney U | 0.721 |
| ONH_RNFL_ST | Dem | MCI | 0 | 0 | Mann-Whitney U | 0.986 |
| ONH_RNFL_ST | Dem | HCs | 0 | 0 | Mann-Whitney U | 0.491 |
| ONH_RNFL_ST | MCI | HCs | 0 | 0 | Mann-Whitney U | 0.381 |
| ONH_RNFL_SN | Dem | HCs | 1 | 1 | pairwise_tukeyhsd | 0.684 |
| ONH_RNFL_SN | Dem | MCI | 1 | 1 | pairwise_tukeyhsd | 0.896 |
| ONH_RNFL_SN | HCs | MCI | 1 | 1 | pairwise_tukeyhsd | 0.137 |
| ONH_RNFL_NU | Dem | MCI | 0 | 0 | Mann-Whitney U | 0.153 |
| ONH_RNFL_NU | Dem | HCs | 0 | 0 | Mann-Whitney U | 0.140 |
| ONH_RNFL_NU | MCI | HCs | 0 | 0 | Mann-Whitney U | 0.998 |
| ONH_RNFL_NL | Dem | MCI | 0 | 0 | Mann-Whitney U | 0.142 |
| ONH_RNFL_NL | Dem | HCs | 0 | 0 | Mann-Whitney U | 0.190 |
| ONH_RNFL_NL | MCI | HCs | 0 | 0 | Mann-Whitney U | 0.719 |
| ONH_RNFL_IN | Dem | HCs | 1 | 1 | pairwise_tukeyhsd | 0.999 |
| ONH_RNFL_IN | Dem | MCI | 1 | 1 | pairwise_tukeyhsd | 0.982 |
| ONH_RNFL_IN | HCs | MCI | 1 | 1 | pairwise_tukeyhsd | 0.977 |
| ONH_RNFL_IT | Dem | MCI | 0 | 0 | Mann-Whitney U | 0.397 |
| ONH_RNFL_IT | Dem | HCs | 0 | 0 | Mann-Whitney U | 0.488 |
| ONH_RNFL_IT | MCI | HCs | 0 | 0 | Mann-Whitney U | 0.980 |
| ONH_RNFL_TL | Dem | MCI | 0 | 0 | Mann-Whitney U | 0.492 |
| ONH_RNFL_TL | Dem | HCs | 0 | 0 | Mann-Whitney U | 0.259 |
| ONH_RNFL_TL | MCI | HCs | 0 | 0 | Mann-Whitney U | 0.608 |
| ONH_RNFL_TU1 | Dem | HCs | 1 | 1 | pairwise_tukeyhsd | 0.711 |
| ONH_RNFL_TU1 | Dem | MCI | 1 | 1 | pairwise_tukeyhsd | 0.588 |
| ONH_RNFL_TU1 | HCs | MCI | 1 | 1 | pairwise_tukeyhsd | 0.943 |
| ONH_RNFL_TU2 | Dem | MCI | 0 | 0 | Mann-Whitney U | 0.059 |
| ONH_RNFL_TU2 | Dem | HCs | 0 | 0 | Mann-Whitney U | 0.247 |
| ONH_RNFL_TU2 | MCI | HCs | 0 | 0 | Mann-Whitney U | 0.289 |
| ONH_RNFL_ST2 | Dem | MCI | 1 | 0 | Dunnett | 0.757 |
| ONH_RNFL_ST2 | Dem | HCs | 1 | 0 | Dunnett | 0.757 |
| ONH_RNFL_ST2 | MCI | HCs | 1 | 0 | Dunnett | 0.082 |
| ONH_RNFL_ST1 | Dem | MCI | 0 | 0 | Mann-Whitney U | 0.339 |
| ONH_RNFL_ST1 | Dem | HCs | 0 | 0 | Mann-Whitney U | 0.140 |
| ONH_RNFL_ST1 | MCI | HCs | 0 | 0 | Mann-Whitney U | 0.660 |
| ONH_RNFL_SN1 | Dem | MCI | 0 | 0 | Mann-Whitney U | 0.726 |
| ONH_RNFL_SN1 | Dem | HCs | 0 | 0 | Mann-Whitney U | 0.155 |
| ONH_RNFL_SN1 | MCI | HCs | 0 | 0 | Mann-Whitney U | **0.020** |
| ONH_RNFL_SN2 | Dem | HCs | 1 | 1 | pairwise_tukeyhsd | 0.938 |
| ONH_RNFL_SN2 | Dem | MCI | 1 | 1 | pairwise_tukeyhsd | 0.863 |
| ONH_RNFL_SN2 | HCs | MCI | 1 | 1 | pairwise_tukeyhsd | 0.400 |
| ONH_RNFL_NU2 | Dem | MCI | 0 | 0 | Mann-Whitney U | 0.111 |
| ONH_RNFL_NU2 | Dem | HCs | 0 | 0 | Mann-Whitney U | 0.192 |
| ONH_RNFL_NU2 | MCI | HCs | 0 | 0 | Mann-Whitney U | 0.655 |
| ONH_RNFL_NU1 | Dem | MCI | 0 | 0 | Mann-Whitney U | 0.302 |
| ONH_RNFL_NU1 | Dem | HCs | 0 | 0 | Mann-Whitney U | 0.170 |
| ONH_RNFL_NU1 | MCI | HCs | 0 | 0 | Mann-Whitney U | 0.565 |
| ONH_RNFL_NL1 | Dem | MCI | 0 | 0 | Mann-Whitney U | 0.201 |
| ONH_RNFL_NL1 | Dem | HCs | 0 | 0 | Mann-Whitney U | 0.229 |
| ONH_RNFL_NL1 | MCI | HCs | 0 | 0 | Mann-Whitney U | 0.828 |
| ONH_RNFL_NL2 | Dem | MCI | 0 | 0 | Mann-Whitney U | 0.117 |
| ONH_RNFL_NL2 | Dem | HCs | 0 | 0 | Mann-Whitney U | 0.201 |
| ONH_RNFL_NL2 | MCI | HCs | 0 | 0 | Mann-Whitney U | 0.577 |
| ONH_RNFL_IN2 | Dem | MCI | 0 | 0 | Mann-Whitney U | 0.644 |
| ONH_RNFL_IN2 | Dem | HCs | 0 | 0 | Mann-Whitney U | 0.729 |
| ONH_RNFL_IN2 | MCI | HCs | 0 | 0 | Mann-Whitney U | 0.575 |
| ONH_RNFL_IN1 | Dem | HCs | 1 | 1 | pairwise_tukeyhsd | 0.993 |
| ONH_RNFL_IN1 | Dem | MCI | 1 | 1 | pairwise_tukeyhsd | 0.966 |
| ONH_RNFL_IN1 | HCs | MCI | 1 | 1 | pairwise_tukeyhsd | 0.976 |
| ONH_RNFL_IT1 | Dem | MCI | 0 | 0 | Mann-Whitney U | 0.607 |
| ONH_RNFL_IT1 | Dem | HCs | 0 | 0 | Mann-Whitney U | 0.312 |
| ONH_RNFL_IT1 | MCI | HCs | 0 | 0 | Mann-Whitney U | 0.549 |
| ONH_RNFL_IT2 | Dem | HCs | 1 | 1 | pairwise_tukeyhsd | 0.569 |
| ONH_RNFL_IT2 | Dem | MCI | 1 | 1 | pairwise_tukeyhsd | 0.452 |
| ONH_RNFL_IT2 | HCs | MCI | 1 | 1 | pairwise_tukeyhsd | 0.943 |
| ONH_RNFL_TL2 | Dem | MCI | 0 | 0 | Mann-Whitney U | 0.327 |
| ONH_RNFL_TL2 | Dem | HCs | 0 | 0 | Mann-Whitney U | 0.343 |
| ONH_RNFL_TL2 | MCI | HCs | 0 | 0 | Mann-Whitney U | 0.897 |
| ONH_RNFL_TL1 | Dem | MCI | 0 | 0 | Mann-Whitney U | 0.644 |
| ONH_RNFL_TL1 | Dem | HCs | 0 | 0 | Mann-Whitney U | 0.225 |
| ONH_RNFL_TL1 | MCI | HCs | 0 | 0 | Mann-Whitney U | 0.265 |
| Macula_3mm_Thk_ILM_IPL_um_Center_1 | Dem | MCI | 0 | 0 | Mann-Whitney U | 0.455 |
| Macula_3mm_Thk_ILM_IPL_um_Center_1 | Dem | HCs | 0 | 0 | Mann-Whitney U | 0.407 |
| Macula_3mm_Thk_ILM_IPL_um_Center_1 | MCI | HCs | 0 | 0 | Mann-Whitney U | 0.890 |
| Macula_3mm_Thk_ILM_IPL_um_T_1minus3 | Dem | MCI | 0 | 0 | Mann-Whitney U | 0.308 |
| Macula_3mm_Thk_ILM_IPL_um_T_1minus3 | Dem | HCs | 0 | 0 | Mann-Whitney U | 0.082 |
| Macula_3mm_Thk_ILM_IPL_um_T_1minus3 | MCI | HCs | 0 | 0 | Mann-Whitney U | 0.327 |
| Macula_3mm_Thk_ILM_IPL_um_S_1minus3 | Dem | MCI | 0 | 0 | Mann-Whitney U | 0.347 |
| Macula_3mm_Thk_ILM_IPL_um_S_1minus3 | Dem | HCs | 0 | 0 | Mann-Whitney U | 0.101 |
| Macula_3mm_Thk_ILM_IPL_um_S_1minus3 | MCI | HCs | 0 | 0 | Mann-Whitney U | 0.359 |
| Macula_3mm_Thk_ILM_IPL_um_N_1minus3 | Dem | MCI | 0 | 0 | Mann-Whitney U | 0.296 |
| Macula_3mm_Thk_ILM_IPL_um_N_1minus3 | Dem | HCs | 0 | 0 | Mann-Whitney U | 0.165 |
| Macula_3mm_Thk_ILM_IPL_um_N_1minus3 | MCI | HCs | 0 | 0 | Mann-Whitney U | 0.691 |
| Macula_3mm_Thk_ILM_IPL_um_I_1minus3 | Dem | MCI | 0 | 0 | Mann-Whitney U | 0.499 |
| Macula_3mm_Thk_ILM_IPL_um_I_1minus3 | Dem | HCs | 0 | 0 | Mann-Whitney U | 0.177 |
| Macula_3mm_Thk_ILM_IPL_um_I_1minus3 | MCI | HCs | 0 | 0 | Mann-Whitney U | 0.373 |
| Macula_3mm_Thk_ILM_IPL_um_S_Hemi_1minus3 | Dem | MCI | 0 | 0 | Mann-Whitney U | 0.266 |
| Macula_3mm_Thk_ILM_IPL_um_S_Hemi_1minus3 | Dem | HCs | 0 | 0 | Mann-Whitney U | 0.090 |
| Macula_3mm_Thk_ILM_IPL_um_S_Hemi_1minus3 | MCI | HCs | 0 | 0 | Mann-Whitney U | 0.398 |
| Macula_3mm_Thk_ILM_IPL_um_I_Hemi_1minus3 | Dem | MCI | 0 | 0 | Mann-Whitney U | 0.437 |
| Macula_3mm_Thk_ILM_IPL_um_I_Hemi_1minus3 | Dem | HCs | 0 | 0 | Mann-Whitney U | 0.168 |
| Macula_3mm_Thk_ILM_IPL_um_I_Hemi_1minus3 | MCI | HCs | 0 | 0 | Mann-Whitney U | 0.392 |
| Macula_3mm_Thk_ILM_IPL_um_All_1minus3 | Dem | MCI | 0 | 0 | Mann-Whitney U | 0.337 |
| Macula_3mm_Thk_ILM_IPL_um_All_1minus3 | Dem | HCs | 0 | 0 | Mann-Whitney U | 0.102 |
| Macula_3mm_Thk_ILM_IPL_um_All_1minus3 | MCI | HCs | 0 | 0 | Mann-Whitney U | 0.338 |
| Macula_3mm_Thk_ILM_IPL_um_S_Hemi_field | Dem | MCI | 0 | 0 | Mann-Whitney U | 0.369 |
| Macula_3mm_Thk_ILM_IPL_um_S_Hemi_field | Dem | HCs | 0 | 0 | Mann-Whitney U | 0.097 |
| Macula_3mm_Thk_ILM_IPL_um_S_Hemi_field | MCI | HCs | 0 | 0 | Mann-Whitney U | 0.331 |
| Macula_3mm_Thk_ILM_IPL_um_I_Hemi_field | Dem | MCI | 0 | 0 | Mann-Whitney U | 0.396 |
| Macula_3mm_Thk_ILM_IPL_um_I_Hemi_field | Dem | HCs | 0 | 0 | Mann-Whitney U | 0.150 |
| Macula_3mm_Thk_ILM_IPL_um_I_Hemi_field | MCI | HCs | 0 | 0 | Mann-Whitney U | 0.434 |
| Macula_3mm_Thk_ILM_IPL_um_All_field | Dem | MCI | 0 | 0 | Mann-Whitney U | 0.344 |
| Macula_3mm_Thk_ILM_IPL_um_All_field | Dem | HCs | 0 | 0 | Mann-Whitney U | 0.106 |
| Macula_3mm_Thk_ILM_IPL_um_All_field | MCI | HCs | 0 | 0 | Mann-Whitney U | 0.337 |
| Macula_3mm_Thk_ILM_RPE_um_Center_1 | Dem | MCI | 0 | 0 | Mann-Whitney U | 0.515 |
| Macula_3mm_Thk_ILM_RPE_um_Center_1 | Dem | HCs | 0 | 0 | Mann-Whitney U | 0.482 |
| Macula_3mm_Thk_ILM_RPE_um_Center_1 | MCI | HCs | 0 | 0 | Mann-Whitney U | 0.911 |
| Macula_3mm_Thk_ILM_RPE_um_T_1minus3 | Dem | MCI | 0 | 0 | Mann-Whitney U | 0.277 |
| Macula_3mm_Thk_ILM_RPE_um_T_1minus3 | Dem | HCs | 0 | 0 | Mann-Whitney U | 0.218 |
| Macula_3mm_Thk_ILM_RPE_um_T_1minus3 | MCI | HCs | 0 | 0 | Mann-Whitney U | 0.809 |
| Macula_3mm_Thk_ILM_RPE_um_S_1minus3 | Dem | MCI | 0 | 0 | Mann-Whitney U | 0.177 |
| Macula_3mm_Thk_ILM_RPE_um_S_1minus3 | Dem | HCs | 0 | 0 | Mann-Whitney U | 0.078 |
| Macula_3mm_Thk_ILM_RPE_um_S_1minus3 | MCI | HCs | 0 | 0 | Mann-Whitney U | 0.562 |
| Macula_3mm_Thk_ILM_RPE_um_N_1minus3 | Dem | MCI | 0 | 0 | Mann-Whitney U | 0.102 |
| Macula_3mm_Thk_ILM_RPE_um_N_1minus3 | Dem | HCs | 0 | 0 | Mann-Whitney U | 0.098 |
| Macula_3mm_Thk_ILM_RPE_um_N_1minus3 | MCI | HCs | 0 | 0 | Mann-Whitney U | 0.974 |
| Macula_3mm_Thk_ILM_RPE_um_I_1minus3 | Dem | MCI | 0 | 0 | Mann-Whitney U | 0.284 |
| Macula_3mm_Thk_ILM_RPE_um_I_1minus3 | Dem | HCs | 0 | 0 | Mann-Whitney U | 0.157 |
| Macula_3mm_Thk_ILM_RPE_um_I_1minus3 | MCI | HCs | 0 | 0 | Mann-Whitney U | 0.610 |
| Macula_3mm_Thk_ILM_RPE_um_S_Hemi_1minus3 | Dem | MCI | 0 | 0 | Mann-Whitney U | 0.122 |
| Macula_3mm_Thk_ILM_RPE_um_S_Hemi_1minus3 | Dem | HCs | 0 | 0 | Mann-Whitney U | 0.071 |
| Macula_3mm_Thk_ILM_RPE_um_S_Hemi_1minus3 | MCI | HCs | 0 | 0 | Mann-Whitney U | 0.725 |
| Macula_3mm_Thk_ILM_RPE_um_I_Hemi_1minus3 | Dem | MCI | 0 | 0 | Mann-Whitney U | 0.208 |
| Macula_3mm_Thk_ILM_RPE_um_I_Hemi_1minus3 | Dem | HCs | 0 | 0 | Mann-Whitney U | 0.144 |
| Macula_3mm_Thk_ILM_RPE_um_I_Hemi_1minus3 | MCI | HCs | 0 | 0 | Mann-Whitney U | 0.729 |
| Macula_3mm_Thk_ILM_RPE_um_All_1minus3 | Dem | MCI | 0 | 0 | Mann-Whitney U | 0.160 |
| Macula_3mm_Thk_ILM_RPE_um_All_1minus3 | Dem | HCs | 0 | 0 | Mann-Whitney U | 0.102 |
| Macula_3mm_Thk_ILM_RPE_um_All_1minus3 | MCI | HCs | 0 | 0 | Mann-Whitney U | 0.690 |
| Macula_3mm_Thk_ILM_RPE_um_S_Hemi_field | Dem | MCI | 0 | 0 | Mann-Whitney U | 0.126 |
| Macula_3mm_Thk_ILM_RPE_um_S_Hemi_field | Dem | HCs | 0 | 0 | Mann-Whitney U | 0.083 |
| Macula_3mm_Thk_ILM_RPE_um_S_Hemi_field | MCI | HCs | 0 | 0 | Mann-Whitney U | 0.776 |
| Macula_3mm_Thk_ILM_RPE_um_I_Hemi_field | Dem | MCI | 0 | 0 | Mann-Whitney U | 0.228 |
| Macula_3mm_Thk_ILM_RPE_um_I_Hemi_field | Dem | HCs | 0 | 0 | Mann-Whitney U | 0.127 |
| Macula_3mm_Thk_ILM_RPE_um_I_Hemi_field | MCI | HCs | 0 | 0 | Mann-Whitney U | 0.620 |
| Macula_3mm_Thk_ILM_RPE_um_All_field | Dem | MCI | 0 | 0 | Mann-Whitney U | 0.155 |
| Macula_3mm_Thk_ILM_RPE_um_All_field | Dem | HCs | 0 | 0 | Mann-Whitney U | 0.099 |
| Macula_3mm_Thk_ILM_RPE_um_All_field | MCI | HCs | 0 | 0 | Mann-Whitney U | 0.642 |
| Macula_3mm_Thk_ILM_BRM_um_Center_1 | Dem | MCI | 0 | 0 | Mann-Whitney U | 0.521 |
| Macula_3mm_Thk_ILM_BRM_um_Center_1 | Dem | HCs | 0 | 0 | Mann-Whitney U | 0.530 |
| Macula_3mm_Thk_ILM_BRM_um_Center_1 | MCI | HCs | 0 | 0 | Mann-Whitney U | 0.913 |
| Macula_3mm_Thk_ILM_BRM_um_T_1minus3 | Dem | MCI | 0 | 0 | Mann-Whitney U | 0.306 |
| Macula_3mm_Thk_ILM_BRM_um_T_1minus3 | Dem | HCs | 0 | 0 | Mann-Whitney U | 0.246 |
| Macula_3mm_Thk_ILM_BRM_um_T_1minus3 | MCI | HCs | 0 | 0 | Mann-Whitney U | 0.906 |
| Macula_3mm_Thk_ILM_BRM_um_S_1minus3 | Dem | MCI | 0 | 0 | Mann-Whitney U | 0.183 |
| Macula_3mm_Thk_ILM_BRM_um_S_1minus3 | Dem | HCs | 0 | 0 | Mann-Whitney U | 0.080 |
| Macula_3mm_Thk_ILM_BRM_um_S_1minus3 | MCI | HCs | 0 | 0 | Mann-Whitney U | 0.579 |
| Macula_3mm_Thk_ILM_BRM_um_N_1minus3 | Dem | MCI | 0 | 0 | Mann-Whitney U | 0.128 |
| Macula_3mm_Thk_ILM_BRM_um_N_1minus3 | Dem | HCs | 0 | 0 | Mann-Whitney U | 0.151 |
| Macula_3mm_Thk_ILM_BRM_um_N_1minus3 | MCI | HCs | 0 | 0 | Mann-Whitney U | 0.923 |
| Macula_3mm_Thk_ILM_BRM_um_I_1minus3 | Dem | MCI | 0 | 0 | Mann-Whitney U | 0.272 |
| Macula_3mm_Thk_ILM_BRM_um_I_1minus3 | Dem | HCs | 0 | 0 | Mann-Whitney U | 0.189 |
| Macula_3mm_Thk_ILM_BRM_um_I_1minus3 | MCI | HCs | 0 | 0 | Mann-Whitney U | 0.746 |
| Macula_3mm_Thk_ILM_BRM_um_S_Hemi_1minus3 | Dem | MCI | 0 | 0 | Mann-Whitney U | 0.138 |
| Macula_3mm_Thk_ILM_BRM_um_S_Hemi_1minus3 | Dem | HCs | 0 | 0 | Mann-Whitney U | 0.095 |
| Macula_3mm_Thk_ILM_BRM_um_S_Hemi_1minus3 | MCI | HCs | 0 | 0 | Mann-Whitney U | 0.774 |
| Macula_3mm_Thk_ILM_BRM_um_I_Hemi_1minus3 | Dem | MCI | 0 | 0 | Mann-Whitney U | 0.223 |
| Macula_3mm_Thk_ILM_BRM_um_I_Hemi_1minus3 | Dem | HCs | 0 | 0 | Mann-Whitney U | 0.207 |
| Macula_3mm_Thk_ILM_BRM_um_I_Hemi_1minus3 | MCI | HCs | 0 | 0 | Mann-Whitney U | 0.930 |
| Macula_3mm_Thk_ILM_BRM_um_All_1minus3 | Dem | MCI | 0 | 0 | Mann-Whitney U | 0.174 |
| Macula_3mm_Thk_ILM_BRM_um_All_1minus3 | Dem | HCs | 0 | 0 | Mann-Whitney U | 0.146 |
| Macula_3mm_Thk_ILM_BRM_um_All_1minus3 | MCI | HCs | 0 | 0 | Mann-Whitney U | 0.796 |
| Macula_3mm_Thk_ILM_BRM_um_S_Hemi_field | Dem | MCI | 0 | 0 | Mann-Whitney U | 0.146 |
| Macula_3mm_Thk_ILM_BRM_um_S_Hemi_field | Dem | HCs | 0 | 0 | Mann-Whitney U | 0.106 |
| Macula_3mm_Thk_ILM_BRM_um_S_Hemi_field | MCI | HCs | 0 | 0 | Mann-Whitney U | 0.833 |
| Macula_3mm_Thk_ILM_BRM_um_I_Hemi_field | Dem | MCI | 0 | 0 | Mann-Whitney U | 0.258 |
| Macula_3mm_Thk_ILM_BRM_um_I_Hemi_field | Dem | HCs | 0 | 0 | Mann-Whitney U | 0.167 |
| Macula_3mm_Thk_ILM_BRM_um_I_Hemi_field | MCI | HCs | 0 | 0 | Mann-Whitney U | 0.803 |
| Macula_3mm_Thk_ILM_BRM_um_All_field | Dem | MCI | 0 | 0 | Mann-Whitney U | 0.184 |
| Macula_3mm_Thk_ILM_BRM_um_All_field | Dem | HCs | 0 | 0 | Mann-Whitney U | 0.133 |
| Macula_3mm_Thk_ILM_BRM_um_All_field | MCI | HCs | 0 | 0 | Mann-Whitney U | 0.768 |
| Macula_3mm_Thk_RPE_BRM_um_Center_1 | Dem | MCI | 0 | 0 | Mann-Whitney U | 0.941 |
| Macula_3mm_Thk_RPE_BRM_um_Center_1 | Dem | HCs | 0 | 0 | Mann-Whitney U | 0.967 |
| Macula_3mm_Thk_RPE_BRM_um_Center_1 | MCI | HCs | 0 | 0 | Mann-Whitney U | 0.869 |
| Macula_3mm_Thk_RPE_BRM_um_T_1minus3 | Dem | MCI | 0 | 0 | Mann-Whitney U | 0.513 |
| Macula_3mm_Thk_RPE_BRM_um_T_1minus3 | Dem | HCs | 0 | 0 | Mann-Whitney U | 0.801 |
| Macula_3mm_Thk_RPE_BRM_um_T_1minus3 | MCI | HCs | 0 | 0 | Mann-Whitney U | 0.389 |
| Macula_3mm_Thk_RPE_BRM_um_S_1minus3 | Dem | MCI | 0 | 0 | Mann-Whitney U | 0.974 |
| Macula_3mm_Thk_RPE_BRM_um_S_1minus3 | Dem | HCs | 0 | 0 | Mann-Whitney U | 0.879 |
| Macula_3mm_Thk_RPE_BRM_um_S_1minus3 | MCI | HCs | 0 | 0 | Mann-Whitney U | 0.973 |
| Macula_3mm_Thk_RPE_BRM_um_N_1minus3 | Dem | MCI | 0 | 0 | Mann-Whitney U | 0.941 |
| Macula_3mm_Thk_RPE_BRM_um_N_1minus3 | Dem | HCs | 0 | 0 | Mann-Whitney U | 0.997 |
| Macula_3mm_Thk_RPE_BRM_um_N_1minus3 | MCI | HCs | 0 | 0 | Mann-Whitney U | 0.855 |
| Macula_3mm_Thk_RPE_BRM_um_I_1minus3 | Dem | MCI | 0 | 0 | Mann-Whitney U | 0.993 |
| Macula_3mm_Thk_RPE_BRM_um_I_1minus3 | Dem | HCs | 0 | 0 | Mann-Whitney U | 0.684 |
| Macula_3mm_Thk_RPE_BRM_um_I_1minus3 | MCI | HCs | 0 | 0 | Mann-Whitney U | 0.452 |
| Macula_3mm_Thk_RPE_BRM_um_S_Hemi_1minus3 | Dem | MCI | 0 | 0 | Mann-Whitney U | 0.999 |
| Macula_3mm_Thk_RPE_BRM_um_S_Hemi_1minus3 | Dem | HCs | 0 | 0 | Mann-Whitney U | 0.914 |
| Macula_3mm_Thk_RPE_BRM_um_S_Hemi_1minus3 | MCI | HCs | 0 | 0 | Mann-Whitney U | 0.932 |
| Macula_3mm_Thk_RPE_BRM_um_I_Hemi_1minus3 | Dem | MCI | 0 | 0 | Mann-Whitney U | 0.832 |
| Macula_3mm_Thk_RPE_BRM_um_I_Hemi_1minus3 | Dem | HCs | 0 | 0 | Mann-Whitney U | 0.965 |
| Macula_3mm_Thk_RPE_BRM_um_I_Hemi_1minus3 | MCI | HCs | 0 | 0 | Mann-Whitney U | 0.477 |
| Macula_3mm_Thk_RPE_BRM_um_All_1minus3 | Dem | MCI | 0 | 0 | Mann-Whitney U | 0.842 |
| Macula_3mm_Thk_RPE_BRM_um_All_1minus3 | Dem | HCs | 0 | 0 | Mann-Whitney U | 0.903 |
| Macula_3mm_Thk_RPE_BRM_um_All_1minus3 | MCI | HCs | 0 | 0 | Mann-Whitney U | 0.695 |
| Macula_3mm_Thk_RPE_BRM_um_S_Hemi_field | Dem | MCI | 0 | 0 | Mann-Whitney U | 0.977 |
| Macula_3mm_Thk_RPE_BRM_um_S_Hemi_field | Dem | HCs | 0 | 0 | Mann-Whitney U | 0.975 |
| Macula_3mm_Thk_RPE_BRM_um_S_Hemi_field | MCI | HCs | 0 | 0 | Mann-Whitney U | 0.870 |
| Macula_3mm_Thk_RPE_BRM_um_I_Hemi_field | Dem | MCI | 0 | 0 | Mann-Whitney U | 0.861 |
| Macula_3mm_Thk_RPE_BRM_um_I_Hemi_field | Dem | HCs | 0 | 0 | Mann-Whitney U | 0.920 |
| Macula_3mm_Thk_RPE_BRM_um_I_Hemi_field | MCI | HCs | 0 | 0 | Mann-Whitney U | 0.502 |
| Macula_3mm_Thk_RPE_BRM_um_All_field | Dem | MCI | 0 | 0 | Mann-Whitney U | 0.861 |
| Macula_3mm_Thk_RPE_BRM_um_All_field | Dem | HCs | 0 | 0 | Mann-Whitney U | 0.950 |
| Macula_3mm_Thk_RPE_BRM_um_All_field | MCI | HCs | 0 | 0 | Mann-Whitney U | 0.661 |
| Macula_3mm_Vol_ILM_IPL_mm3_Center_1 | Dem | MCI | 0 | 0 | Mann-Whitney U | 0.462 |
| Macula_3mm_Vol_ILM_IPL_mm3_Center_1 | Dem | HCs | 0 | 0 | Mann-Whitney U | 0.397 |
| Macula_3mm_Vol_ILM_IPL_mm3_Center_1 | MCI | HCs | 0 | 0 | Mann-Whitney U | 0.889 |
| Macula_3mm_Vol_ILM_IPL_mm3_T_1minus3 | Dem | MCI | 0 | 0 | Mann-Whitney U | 0.343 |
| Macula_3mm_Vol_ILM_IPL_mm3_T_1minus3 | Dem | HCs | 0 | 0 | Mann-Whitney U | 0.090 |
| Macula_3mm_Vol_ILM_IPL_mm3_T_1minus3 | MCI | HCs | 0 | 0 | Mann-Whitney U | 0.302 |
| Macula_3mm_Vol_ILM_IPL_mm3_S_1minus3 | Dem | MCI | 0 | 0 | Mann-Whitney U | 0.584 |
| Macula_3mm_Vol_ILM_IPL_mm3_S_1minus3 | Dem | HCs | 0 | 0 | Mann-Whitney U | 0.169 |
| Macula_3mm_Vol_ILM_IPL_mm3_S_1minus3 | MCI | HCs | 0 | 0 | Mann-Whitney U | 0.286 |
| Macula_3mm_Vol_ILM_IPL_mm3_N_1minus3 | Dem | MCI | 0 | 0 | Mann-Whitney U | 0.387 |
| Macula_3mm_Vol_ILM_IPL_mm3_N_1minus3 | Dem | HCs | 0 | 0 | Mann-Whitney U | 0.178 |
| Macula_3mm_Vol_ILM_IPL_mm3_N_1minus3 | MCI | HCs | 0 | 0 | Mann-Whitney U | 0.454 |
| Macula_3mm_Vol_ILM_IPL_mm3_I_1minus3 | Dem | MCI | 0 | 0 | Mann-Whitney U | 0.452 |
| Macula_3mm_Vol_ILM_IPL_mm3_I_1minus3 | Dem | HCs | 0 | 0 | Mann-Whitney U | 0.254 |
| Macula_3mm_Vol_ILM_IPL_mm3_I_1minus3 | MCI | HCs | 0 | 0 | Mann-Whitney U | 0.644 |
| Macula_3mm_Vol_ILM_IPL_mm3_S_Hemi_1minus3 | Dem | MCI | 0 | 0 | Mann-Whitney U | 0.453 |
| Macula_3mm_Vol_ILM_IPL_mm3_S_Hemi_1minus3 | Dem | HCs | 0 | 0 | Mann-Whitney U | 0.117 |
| Macula_3mm_Vol_ILM_IPL_mm3_S_Hemi_1minus3 | MCI | HCs | 0 | 0 | Mann-Whitney U | 0.218 |
| Macula_3mm_Vol_ILM_IPL_mm3_I_Hemi_1minus3 | Dem | MCI | 0 | 0 | Mann-Whitney U | 0.422 |
| Macula_3mm_Vol_ILM_IPL_mm3_I_Hemi_1minus3 | Dem | HCs | 0 | 0 | Mann-Whitney U | 0.176 |
| Macula_3mm_Vol_ILM_IPL_mm3_I_Hemi_1minus3 | MCI | HCs | 0 | 0 | Mann-Whitney U | 0.452 |
| Macula_3mm_Vol_ILM_IPL_mm3_All_1minus3 | Dem | MCI | 0 | 0 | Mann-Whitney U | 0.419 |
| Macula_3mm_Vol_ILM_IPL_mm3_All_1minus3 | Dem | HCs | 0 | 0 | Mann-Whitney U | 0.103 |
| Macula_3mm_Vol_ILM_IPL_mm3_All_1minus3 | MCI | HCs | 0 | 0 | Mann-Whitney U | 0.287 |
| Macula_3mm_Vol_ILM_IPL_mm3_S_Hemi_field | Dem | MCI | 0 | 0 | Mann-Whitney U | 0.904 |
| Macula_3mm_Vol_ILM_IPL_mm3_S_Hemi_field | Dem | HCs | 0 | 0 | Mann-Whitney U | 0.199 |
| Macula_3mm_Vol_ILM_IPL_mm3_S_Hemi_field | MCI | HCs | 0 | 0 | Mann-Whitney U | 0.093 |
| Macula_3mm_Vol_ILM_IPL_mm3_I_Hemi_field | Dem | MCI | 0 | 0 | Mann-Whitney U | 0.155 |
| Macula_3mm_Vol_ILM_IPL_mm3_I_Hemi_field | Dem | HCs | 0 | 0 | Mann-Whitney U | 0.289 |
| Macula_3mm_Vol_ILM_IPL_mm3_I_Hemi_field | MCI | HCs | 0 | 0 | Mann-Whitney U | 0.673 |
| Macula_3mm_Vol_ILM_IPL_mm3_All_field | Dem | MCI | 0 | 0 | Mann-Whitney U | 0.345 |
| Macula_3mm_Vol_ILM_IPL_mm3_All_field | Dem | HCs | 0 | 0 | Mann-Whitney U | 0.108 |
| Macula_3mm_Vol_ILM_IPL_mm3_All_field | MCI | HCs | 0 | 0 | Mann-Whitney U | 0.340 |
| Macula_3mm_Vol_ILM_RPE_mm3_Center_1 | Dem | MCI | 0 | 0 | Mann-Whitney U | 0.511 |
| Macula_3mm_Vol_ILM_RPE_mm3_Center_1 | Dem | HCs | 0 | 0 | Mann-Whitney U | 0.453 |
| Macula_3mm_Vol_ILM_RPE_mm3_Center_1 | MCI | HCs | 0 | 0 | Mann-Whitney U | 0.903 |
| Macula_3mm_Vol_ILM_RPE_mm3_T_1minus3 | Dem | MCI | 0 | 0 | Mann-Whitney U | 0.282 |
| Macula_3mm_Vol_ILM_RPE_mm3_T_1minus3 | Dem | HCs | 0 | 0 | Mann-Whitney U | 0.227 |
| Macula_3mm_Vol_ILM_RPE_mm3_T_1minus3 | MCI | HCs | 0 | 0 | Mann-Whitney U | 0.859 |
| Macula_3mm_Vol_ILM_RPE_mm3_S_1minus3 | Dem | MCI | 0 | 0 | Mann-Whitney U | 0.292 |
| Macula_3mm_Vol_ILM_RPE_mm3_S_1minus3 | Dem | HCs | 0 | 0 | Mann-Whitney U | 0.082 |
| Macula_3mm_Vol_ILM_RPE_mm3_S_1minus3 | MCI | HCs | 0 | 0 | Mann-Whitney U | 0.301 |
| Macula_3mm_Vol_ILM_RPE_mm3_N_1minus3 | Dem | MCI | 0 | 0 | Mann-Whitney U | 0.120 |
| Macula_3mm_Vol_ILM_RPE_mm3_N_1minus3 | Dem | HCs | 0 | 0 | Mann-Whitney U | **0.048** |
| Macula_3mm_Vol_ILM_RPE_mm3_N_1minus3 | MCI | HCs | 0 | 0 | Mann-Whitney U | 0.644 |
| Macula_3mm_Vol_ILM_RPE_mm3_I_1minus3 | Dem | MCI | 0 | 0 | Mann-Whitney U | 0.205 |
| Macula_3mm_Vol_ILM_RPE_mm3_I_1minus3 | Dem | HCs | 0 | 0 | Mann-Whitney U | 0.265 |
| Macula_3mm_Vol_ILM_RPE_mm3_I_1minus3 | MCI | HCs | 0 | 0 | Mann-Whitney U | 0.932 |
| Macula_3mm_Vol_ILM_RPE_mm3_S_Hemi_1minus3 | Dem | MCI | 0 | 0 | Mann-Whitney U | 0.237 |
| Macula_3mm_Vol_ILM_RPE_mm3_S_Hemi_1minus3 | Dem | HCs | 0 | 0 | Mann-Whitney U | **0.047** |
| Macula_3mm_Vol_ILM_RPE_mm3_S_Hemi_1minus3 | MCI | HCs | 0 | 0 | Mann-Whitney U | 0.328 |
| Macula_3mm_Vol_ILM_RPE_mm3_I_Hemi_1minus3 | Dem | MCI | 0 | 0 | Mann-Whitney U | 0.198 |
| Macula_3mm_Vol_ILM_RPE_mm3_I_Hemi_1minus3 | Dem | HCs | 0 | 0 | Mann-Whitney U | 0.143 |
| Macula_3mm_Vol_ILM_RPE_mm3_I_Hemi_1minus3 | MCI | HCs | 0 | 0 | Mann-Whitney U | 0.780 |
| Macula_3mm_Vol_ILM_RPE_mm3_All_1minus3 | Dem | MCI | 0 | 0 | Mann-Whitney U | 0.202 |
| Macula_3mm_Vol_ILM_RPE_mm3_All_1minus3 | Dem | HCs | 0 | 0 | Mann-Whitney U | 0.083 |
| Macula_3mm_Vol_ILM_RPE_mm3_All_1minus3 | MCI | HCs | 0 | 0 | Mann-Whitney U | 0.508 |
| Macula_3mm_Vol_ILM_RPE_mm3_S_Hemi_field | Dem | MCI | 0 | 0 | Mann-Whitney U | 0.567 |
| Macula_3mm_Vol_ILM_RPE_mm3_S_Hemi_field | Dem | HCs | 0 | 0 | Mann-Whitney U | 0.090 |
| Macula_3mm_Vol_ILM_RPE_mm3_S_Hemi_field | MCI | HCs | 0 | 0 | Mann-Whitney U | 0.075 |
| Macula_3mm_Vol_ILM_RPE_mm3_I_Hemi_field | Dem | MCI | 0 | 0 | Mann-Whitney U | 0.096 |
| Macula_3mm_Vol_ILM_RPE_mm3_I_Hemi_field | Dem | HCs | 0 | 0 | Mann-Whitney U | 0.407 |
| Macula_3mm_Vol_ILM_RPE_mm3_I_Hemi_field | MCI | HCs | 0 | 0 | Mann-Whitney U | 0.245 |
| Macula_3mm_Vol_ILM_RPE_mm3_All_field | Dem | MCI | 0 | 0 | Mann-Whitney U | 0.155 |
| Macula_3mm_Vol_ILM_RPE_mm3_All_field | Dem | HCs | 0 | 0 | Mann-Whitney U | 0.099 |
| Macula_3mm_Vol_ILM_RPE_mm3_All_field | MCI | HCs | 0 | 0 | Mann-Whitney U | 0.644 |
| Macula_3mm_Vol_ILM_BRM_mm3_Center_1 | Dem | MCI | 0 | 0 | Mann-Whitney U | 0.487 |
| Macula_3mm_Vol_ILM_BRM_mm3_Center_1 | Dem | HCs | 0 | 0 | Mann-Whitney U | 0.504 |
| Macula_3mm_Vol_ILM_BRM_mm3_Center_1 | MCI | HCs | 0 | 0 | Mann-Whitney U | 0.908 |
| Macula_3mm_Vol_ILM_BRM_mm3_T_1minus3 | Dem | MCI | 0 | 0 | Mann-Whitney U | 0.285 |
| Macula_3mm_Vol_ILM_BRM_mm3_T_1minus3 | Dem | HCs | 0 | 0 | Mann-Whitney U | 0.271 |
| Macula_3mm_Vol_ILM_BRM_mm3_T_1minus3 | MCI | HCs | 0 | 0 | Mann-Whitney U | 0.938 |
| Macula_3mm_Vol_ILM_BRM_mm3_S_1minus3 | Dem | MCI | 0 | 0 | Mann-Whitney U | 0.343 |
| Macula_3mm_Vol_ILM_BRM_mm3_S_1minus3 | Dem | HCs | 0 | 0 | Mann-Whitney U | 0.102 |
| Macula_3mm_Vol_ILM_BRM_mm3_S_1minus3 | MCI | HCs | 0 | 0 | Mann-Whitney U | 0.302 |
| Macula_3mm_Vol_ILM_BRM_mm3_N_1minus3 | Dem | MCI | 0 | 0 | Mann-Whitney U | 0.153 |
| Macula_3mm_Vol_ILM_BRM_mm3_N_1minus3 | Dem | HCs | 0 | 0 | Mann-Whitney U | 0.073 |
| Macula_3mm_Vol_ILM_BRM_mm3_N_1minus3 | MCI | HCs | 0 | 0 | Mann-Whitney U | 0.636 |
| Macula_3mm_Vol_ILM_BRM_mm3_I_1minus3 | Dem | MCI | 0 | 0 | Mann-Whitney U | 0.210 |
| Macula_3mm_Vol_ILM_BRM_mm3_I_1minus3 | Dem | HCs | 0 | 0 | Mann-Whitney U | 0.286 |
| Macula_3mm_Vol_ILM_BRM_mm3_I_1minus3 | MCI | HCs | 0 | 0 | Mann-Whitney U | 0.800 |
| Macula_3mm_Vol_ILM_BRM_mm3_S_Hemi_1minus3 | Dem | MCI | 0 | 0 | Mann-Whitney U | 0.262 |
| Macula_3mm_Vol_ILM_BRM_mm3_S_Hemi_1minus3 | Dem | HCs | 0 | 0 | Mann-Whitney U | 0.061 |
| Macula_3mm_Vol_ILM_BRM_mm3_S_Hemi_1minus3 | MCI | HCs | 0 | 0 | Mann-Whitney U | 0.364 |
| Macula_3mm_Vol_ILM_BRM_mm3_I_Hemi_1minus3 | Dem | MCI | 0 | 0 | Mann-Whitney U | 0.223 |
| Macula_3mm_Vol_ILM_BRM_mm3_I_Hemi_1minus3 | Dem | HCs | 0 | 0 | Mann-Whitney U | 0.202 |
| Macula_3mm_Vol_ILM_BRM_mm3_I_Hemi_1minus3 | MCI | HCs | 0 | 0 | Mann-Whitney U | 0.932 |
| Macula_3mm_Vol_ILM_BRM_mm3_All_1minus3 | Dem | MCI | 0 | 0 | Mann-Whitney U | 0.220 |
| Macula_3mm_Vol_ILM_BRM_mm3_All_1minus3 | Dem | HCs | 0 | 0 | Mann-Whitney U | 0.113 |
| Macula_3mm_Vol_ILM_BRM_mm3_All_1minus3 | MCI | HCs | 0 | 0 | Mann-Whitney U | 0.575 |
| Macula_3mm_Vol_ILM_BRM_mm3_S_Hemi_field | Dem | MCI | 0 | 0 | Mann-Whitney U | 0.644 |
| Macula_3mm_Vol_ILM_BRM_mm3_S_Hemi_field | Dem | HCs | 0 | 0 | Mann-Whitney U | 0.118 |
| Macula_3mm_Vol_ILM_BRM_mm3_S_Hemi_field | MCI | HCs | 0 | 0 | Mann-Whitney U | 0.072 |
| Macula_3mm_Vol_ILM_BRM_mm3_I_Hemi_field | Dem | MCI | 0 | 0 | Mann-Whitney U | 0.105 |
| Macula_3mm_Vol_ILM_BRM_mm3_I_Hemi_field | Dem | HCs | 0 | 0 | Mann-Whitney U | 0.437 |
| Macula_3mm_Vol_ILM_BRM_mm3_I_Hemi_field | MCI | HCs | 0 | 0 | Mann-Whitney U | 0.240 |
| Macula_3mm_Vol_ILM_BRM_mm3_All_field | Dem | MCI | 0 | 0 | Mann-Whitney U | 0.185 |
| Macula_3mm_Vol_ILM_BRM_mm3_All_field | Dem | HCs | 0 | 0 | Mann-Whitney U | 0.133 |
| Macula_3mm_Vol_ILM_BRM_mm3_All_field | MCI | HCs | 0 | 0 | Mann-Whitney U | 0.766 |
| Macula_3mm_Vol_RPE_BRM_mm3_Center_1 | Dem | MCI | 0 | 0 | Mann-Whitney U | 0.988 |
| Macula_3mm_Vol_RPE_BRM_mm3_Center_1 | Dem | HCs | 0 | 0 | Mann-Whitney U | 0.936 |
| Macula_3mm_Vol_RPE_BRM_mm3_Center_1 | MCI | HCs | 0 | 0 | Mann-Whitney U | 0.800 |
| Macula_3mm_Vol_RPE_BRM_mm3_T_1minus3 | Dem | MCI | 0 | 0 | Mann-Whitney U | 0.701 |
| Macula_3mm_Vol_RPE_BRM_mm3_T_1minus3 | Dem | HCs | 0 | 0 | Mann-Whitney U | 0.910 |
| Macula_3mm_Vol_RPE_BRM_mm3_T_1minus3 | MCI | HCs | 0 | 0 | Mann-Whitney U | 0.360 |
| Macula_3mm_Vol_RPE_BRM_mm3_S_1minus3 | Dem | MCI | 0 | 0 | Mann-Whitney U | 0.988 |
| Macula_3mm_Vol_RPE_BRM_mm3_S_1minus3 | Dem | HCs | 0 | 0 | Mann-Whitney U | 0.909 |
| Macula_3mm_Vol_RPE_BRM_mm3_S_1minus3 | MCI | HCs | 0 | 0 | Mann-Whitney U | 0.866 |
| Macula_3mm_Vol_RPE_BRM_mm3_N_1minus3 | Dem | MCI | 0 | 0 | Mann-Whitney U | 0.884 |
| Macula_3mm_Vol_RPE_BRM_mm3_N_1minus3 | Dem | HCs | 0 | 0 | Mann-Whitney U | 0.990 |
| Macula_3mm_Vol_RPE_BRM_mm3_N_1minus3 | MCI | HCs | 0 | 0 | Mann-Whitney U | 0.804 |
| Macula_3mm_Vol_RPE_BRM_mm3_I_1minus3 | Dem | MCI | 0 | 0 | Mann-Whitney U | 0.978 |
| Macula_3mm_Vol_RPE_BRM_mm3_I_1minus3 | Dem | HCs | 0 | 0 | Mann-Whitney U | 0.760 |
| Macula_3mm_Vol_RPE_BRM_mm3_I_1minus3 | MCI | HCs | 0 | 0 | Mann-Whitney U | 0.606 |
| Macula_3mm_Vol_RPE_BRM_mm3_S_Hemi_1minus3 | Dem | MCI | 0 | 0 | Mann-Whitney U | 0.975 |
| Macula_3mm_Vol_RPE_BRM_mm3_S_Hemi_1minus3 | Dem | HCs | 0 | 0 | Mann-Whitney U | 0.872 |
| Macula_3mm_Vol_RPE_BRM_mm3_S_Hemi_1minus3 | MCI | HCs | 0 | 0 | Mann-Whitney U | 0.885 |
| Macula_3mm_Vol_RPE_BRM_mm3_I_Hemi_1minus3 | Dem | MCI | 0 | 0 | Mann-Whitney U | 0.838 |
| Macula_3mm_Vol_RPE_BRM_mm3_I_Hemi_1minus3 | Dem | HCs | 0 | 0 | Mann-Whitney U | 0.912 |
| Macula_3mm_Vol_RPE_BRM_mm3_I_Hemi_1minus3 | MCI | HCs | 0 | 0 | Mann-Whitney U | 0.483 |
| Macula_3mm_Vol_RPE_BRM_mm3_All_1minus3 | Dem | MCI | 0 | 0 | Mann-Whitney U | 0.917 |
| Macula_3mm_Vol_RPE_BRM_mm3_All_1minus3 | Dem | HCs | 0 | 0 | Mann-Whitney U | 0.966 |
| Macula_3mm_Vol_RPE_BRM_mm3_All_1minus3 | MCI | HCs | 0 | 0 | Mann-Whitney U | 0.762 |
| Macula_3mm_Vol_RPE_BRM_mm3_S_Hemi_field | Dem | MCI | 0 | 0 | Mann-Whitney U | 0.939 |
| Macula_3mm_Vol_RPE_BRM_mm3_S_Hemi_field | Dem | HCs | 0 | 0 | Mann-Whitney U | 0.943 |
| Macula_3mm_Vol_RPE_BRM_mm3_S_Hemi_field | MCI | HCs | 0 | 0 | Mann-Whitney U | 0.861 |
| Macula_3mm_Vol_RPE_BRM_mm3_I_Hemi_field | Dem | MCI | 0 | 0 | Mann-Whitney U | 0.747 |
| Macula_3mm_Vol_RPE_BRM_mm3_I_Hemi_field | Dem | HCs | 0 | 0 | Mann-Whitney U | 0.841 |
| Macula_3mm_Vol_RPE_BRM_mm3_I_Hemi_field | MCI | HCs | 0 | 0 | Mann-Whitney U | 0.386 |
| Macula_3mm_Vol_RPE_BRM_mm3_All_field | Dem | MCI | 0 | 0 | Mann-Whitney U | 0.838 |
| Macula_3mm_Vol_RPE_BRM_mm3_All_field | Dem | HCs | 0 | 0 | Mann-Whitney U | 0.947 |
| Macula_3mm_Vol_RPE_BRM_mm3_All_field | MCI | HCs | 0 | 0 | Mann-Whitney U | 0.616 |
| SVC_L1_DensityOfWhole_Image | Dem | MCI | 0 | 0 | Mann-Whitney U | **0.014** |
| SVC_L1_DensityOfWhole_Image | Dem | HCs | 0 | 0 | Mann-Whitney U | **<0.001** |
| SVC_L1_DensityOfWhole_Image | MCI | HCs | 0 | 0 | Mann-Whitney U | **0.002** |
| SVC_L1_Whole_Image_S_Hemi | Dem | MCI | 0 | 0 | Mann-Whitney U | **0.022** |
| SVC_L1_Whole_Image_S_Hemi | Dem | HCs | 0 | 0 | Mann-Whitney U | **<0.001** |
| SVC_L1_Whole_Image_S_Hemi | MCI | HCs | 0 | 0 | Mann-Whitney U | **0.004** |
| SVC_L1_Whole_Image_I_Hemi | Dem | MCI | 0 | 0 | Mann-Whitney U | **0.013** |
| SVC_L1_Whole_Image_I_Hemi | Dem | HCs | 0 | 0 | Mann-Whitney U | **<0.001** |
| SVC_L1_Whole_Image_I_Hemi | MCI | HCs | 0 | 0 | Mann-Whitney U | **0.001** |
| SVC_L1_Whole_ETDRS | Dem | MCI | 0 | 0 | Mann-Whitney U | **0.014** |
| SVC_L1_Whole_ETDRS | Dem | HCs | 0 | 0 | Mann-Whitney U | **<0.001** |
| SVC_L1_Whole_ETDRS | MCI | HCs | 0 | 0 | Mann-Whitney U | **0.003** |
| SVC_L1_Fovea | Dem | HCs | 1 | 1 | pairwise_tukeyhsd | 0.080 |
| SVC_L1_Fovea | Dem | MCI | 1 | 1 | pairwise_tukeyhsd | 0.572 |
| SVC_L1_Fovea | HCs | MCI | 1 | 1 | pairwise_tukeyhsd | 0.220 |
| SVC_L1_ParaFovea | Dem | MCI | 0 | 0 | Mann-Whitney U | **0.010** |
| SVC_L1_ParaFovea | Dem | HCs | 0 | 0 | Mann-Whitney U | **<0.001** |
| SVC_L1_ParaFovea | MCI | HCs | 0 | 0 | Mann-Whitney U | **0.004** |
| SVC_L1_Para_S_Hemi | Dem | MCI | 0 | 0 | Mann-Whitney U | **0.015** |
| SVC_L1_Para_S_Hemi | Dem | HCs | 0 | 0 | Mann-Whitney U | **<0.001** |
| SVC_L1_Para_S_Hemi | MCI | HCs | 0 | 0 | Mann-Whitney U | **0.005** |
| SVC_L1_Para_I_Hemi | Dem | MCI | 0 | 0 | Mann-Whitney U | **0.010** |
| SVC_L1_Para_I_Hemi | Dem | HCs | 0 | 0 | Mann-Whitney U | **<0.001** |
| SVC_L1_Para_I_Hemi | MCI | HCs | 0 | 0 | Mann-Whitney U | **0.003** |
| SVC_L1_Para_T | Dem | MCI | 0 | 0 | Mann-Whitney U | 0.061 |
| SVC_L1_Para_T | Dem | HCs | 0 | 0 | Mann-Whitney U | **<0.001** |
| SVC_L1_Para_T | MCI | HCs | 0 | 0 | Mann-Whitney U | **0.001** |
| SVC_L1_Para_S | Dem | MCI | 0 | 0 | Mann-Whitney U | **0.010** |
| SVC_L1_Para_S | Dem | HCs | 0 | 0 | Mann-Whitney U | **<0.001** |
| SVC_L1_Para_S | MCI | HCs | 0 | 0 | Mann-Whitney U | **0.021** |
| SVC_L1_Para_N | Dem | MCI | 0 | 0 | Mann-Whitney U | **0.040** |
| SVC_L1_Para_N | Dem | HCs | 0 | 0 | Mann-Whitney U | **<0.001** |
| SVC_L1_Para_N | MCI | HCs | 0 | 0 | Mann-Whitney U | **0.004** |
| SVC_L1_Para_I | Dem | MCI | 0 | 0 | Mann-Whitney U | **0.019** |
| SVC_L1_Para_I | Dem | HCs | 0 | 0 | Mann-Whitney U | **<0.001** |
| SVC_L1_Para_I | MCI | HCs | 0 | 0 | Mann-Whitney U | **0.016** |
| SVC_L1_G11 | Dem | MCI | 0 | 0 | Mann-Whitney U | 0.110 |
| SVC_L1_G11 | Dem | HCs | 0 | 0 | Mann-Whitney U | **<0.001** |
| SVC_L1_G11 | MCI | HCs | 0 | 0 | Mann-Whitney U | **<0.001** |
| SVC_L1_G12 | Dem | MCI | 0 | 0 | Mann-Whitney U | **0.023** |
| SVC_L1_G12 | Dem | HCs | 0 | 0 | Mann-Whitney U | **<0.001** |
| SVC_L1_G12 | MCI | HCs | 0 | 0 | Mann-Whitney U | **0.039** |
| SVC_L1_G13 | Dem | MCI | 0 | 0 | Mann-Whitney U | **0.023** |
| SVC_L1_G13 | Dem | HCs | 0 | 0 | Mann-Whitney U | **0.001** |
| SVC_L1_G13 | MCI | HCs | 0 | 0 | Mann-Whitney U | 0.053 |
| SVC_L1_G21 | Dem | MCI | 0 | 0 | Mann-Whitney U | **0.017** |
| SVC_L1_G21 | Dem | HCs | 0 | 0 | Mann-Whitney U | **<0.001** |
| SVC_L1_G21 | MCI | HCs | 0 | 0 | Mann-Whitney U | **0.025** |
| SVC_L1_G22 | Dem | HCs | 1 | 1 | pairwise_tukeyhsd | 0.069 |
| SVC_L1_G22 | Dem | MCI | 1 | 1 | pairwise_tukeyhsd | 0.645 |
| SVC_L1_G22 | HCs | MCI | 1 | 1 | pairwise_tukeyhsd | 0.129 |
| SVC_L1_G23 | Dem | MCI | 0 | 0 | Mann-Whitney U | 0.212 |
| SVC_L1_G23 | Dem | HCs | 0 | 0 | Mann-Whitney U | **0.001** |
| SVC_L1_G23 | MCI | HCs | 0 | 0 | Mann-Whitney U | **0.003** |
| SVC_L1_G31 | Dem | MCI | 0 | 0 | Mann-Whitney U | **0.046** |
| SVC_L1_G31 | Dem | HCs | 0 | 0 | Mann-Whitney U | **<0.001** |
| SVC_L1_G31 | MCI | HCs | 0 | 0 | Mann-Whitney U | **0.017** |
| SVC_L1_G32 | Dem | MCI | 0 | 0 | Mann-Whitney U | 0.073 |
| SVC_L1_G32 | Dem | HCs | 0 | 0 | Mann-Whitney U | **<0.001** |
| SVC_L1_G32 | MCI | HCs | 0 | 0 | Mann-Whitney U | **0.008** |
| SVC_L1_G33 | Dem | MCI | 0 | 0 | Mann-Whitney U | **0.021** |
| SVC_L1_G33 | Dem | HCs | 0 | 0 | Mann-Whitney U | **<0.001** |
| SVC_L1_G33 | MCI | HCs | 0 | 0 | Mann-Whitney U | **0.009** |
| DVC_L2_DensityOfWhole_Image | Dem | MCI | 0 | 0 | Mann-Whitney U | **0.026** |
| DVC_L2_DensityOfWhole_Image | Dem | HCs | 0 | 0 | Mann-Whitney U | **0.002** |
| DVC_L2_DensityOfWhole_Image | MCI | HCs | 0 | 0 | Mann-Whitney U | 0.271 |
| DVC_L2_Whole_Image_S_Hemi | Dem | MCI | 0 | 0 | Mann-Whitney U | **0.045** |
| DVC_L2_Whole_Image_S_Hemi | Dem | HCs | 0 | 0 | Mann-Whitney U | **0.006** |
| DVC_L2_Whole_Image_S_Hemi | MCI | HCs | 0 | 0 | Mann-Whitney U | 0.349 |
| DVC_L2_Whole_Image_I_Hemi | Dem | MCI | 0 | 0 | Mann-Whitney U | **0.019** |
| DVC_L2_Whole_Image_I_Hemi | Dem | HCs | 0 | 0 | Mann-Whitney U | **0.001** |
| DVC_L2_Whole_Image_I_Hemi | MCI | HCs | 0 | 0 | Mann-Whitney U | 0.273 |
| DVC_L2_Whole_ETDRS | Dem | MCI | 0 | 0 | Mann-Whitney U | 0.050 |
| DVC_L2_Whole_ETDRS | Dem | HCs | 0 | 0 | Mann-Whitney U | **0.004** |
| DVC_L2_Whole_ETDRS | MCI | HCs | 0 | 0 | Mann-Whitney U | 0.224 |
| DVC_L2_Fovea | Dem | HCs | 1 | 1 | pairwise_tukeyhsd | 0.652 |
| DVC_L2_Fovea | Dem | MCI | 1 | 1 | pairwise_tukeyhsd | 0.745 |
| DVC_L2_Fovea | HCs | MCI | 1 | 1 | pairwise_tukeyhsd | 0.979 |
| DVC_L2_ParaFovea | Dem | MCI | 0 | 0 | Mann-Whitney U | 0.061 |
| DVC_L2_ParaFovea | Dem | HCs | 0 | 0 | Mann-Whitney U | **0.004** |
| DVC_L2_ParaFovea | MCI | HCs | 0 | 0 | Mann-Whitney U | 0.224 |
| DVC_L2_Para_S_Hemi | Dem | MCI | 0 | 0 | Mann-Whitney U | 0.096 |
| DVC_L2_Para_S_Hemi | Dem | HCs | 0 | 0 | Mann-Whitney U | **0.021** |
| DVC_L2_Para_S_Hemi | MCI | HCs | 0 | 0 | Mann-Whitney U | 0.374 |
| DVC_L2_Para_I_Hemi | Dem | MCI | 0 | 0 | Mann-Whitney U | 0.058 |
| DVC_L2_Para_I_Hemi | Dem | HCs | 0 | 0 | Mann-Whitney U | **0.002** |
| DVC_L2_Para_I_Hemi | MCI | HCs | 0 | 0 | Mann-Whitney U | 0.149 |
| DVC_L2_Para_T | Dem | MCI | 0 | 0 | Mann-Whitney U | 0.205 |
| DVC_L2_Para_T | Dem | HCs | 0 | 0 | Mann-Whitney U | **0.013** |
| DVC_L2_Para_T | MCI | HCs | 0 | 0 | Mann-Whitney U | 0.127 |
| DVC_L2_Para_S | Dem | MCI | 0 | 0 | Mann-Whitney U | 0.202 |
| DVC_L2_Para_S | Dem | HCs | 0 | 0 | Mann-Whitney U | 0.067 |
| DVC_L2_Para_S | MCI | HCs | 0 | 0 | Mann-Whitney U | 0.397 |
| DVC_L2_Para_N | Dem | MCI | 0 | 0 | Mann-Whitney U | 0.071 |
| DVC_L2_Para_N | Dem | HCs | 0 | 0 | Mann-Whitney U | **0.011** |
| DVC_L2_Para_N | MCI | HCs | 0 | 0 | Mann-Whitney U | 0.435 |
| DVC_L2_Para_I | Dem | MCI | 0 | 0 | Mann-Whitney U | **0.033** |
| DVC_L2_Para_I | Dem | HCs | 0 | 0 | Mann-Whitney U | **0.002** |
| DVC_L2_Para_I | MCI | HCs | 0 | 0 | Mann-Whitney U | 0.182 |
| DVC_L2_G11 | Dem | MCI | 0 | 0 | Mann-Whitney U | 0.058 |
| DVC_L2_G11 | Dem | HCs | 0 | 0 | Mann-Whitney U | **0.004** |
| DVC_L2_G11 | MCI | HCs | 0 | 0 | Mann-Whitney U | 0.307 |
| DVC_L2_G12 | Dem | MCI | 0 | 0 | Mann-Whitney U | 0.174 |
| DVC_L2_G12 | Dem | HCs | 0 | 0 | Mann-Whitney U | 0.081 |
| DVC_L2_G12 | MCI | HCs | 0 | 0 | Mann-Whitney U | 0.603 |
| DVC_L2_G13 | Dem | MCI | 0 | 0 | Mann-Whitney U | 0.116 |
| DVC_L2_G13 | Dem | HCs | 0 | 0 | Mann-Whitney U | **0.025** |
| DVC_L2_G13 | MCI | HCs | 0 | 0 | Mann-Whitney U | 0.347 |
| DVC_L2_G21 | Dem | MCI | 0 | 0 | Mann-Whitney U | 0.079 |
| DVC_L2_G21 | Dem | HCs | 0 | 0 | Mann-Whitney U | **0.010** |
| DVC_L2_G21 | MCI | HCs | 0 | 0 | Mann-Whitney U | 0.332 |
| DVC_L2_G22 | Dem | HCs | 1 | 1 | pairwise_tukeyhsd | 0.788 |
| DVC_L2_G22 | Dem | MCI | 1 | 1 | pairwise_tukeyhsd | 0.881 |
| DVC_L2_G22 | HCs | MCI | 1 | 1 | pairwise_tukeyhsd | 0.967 |
| DVC_L2_G23 | Dem | MCI | 0 | 0 | Mann-Whitney U | 0.072 |
| DVC_L2_G23 | Dem | HCs | 0 | 0 | Mann-Whitney U | **0.014** |
| DVC_L2_G23 | MCI | HCs | 0 | 0 | Mann-Whitney U | 0.401 |
| DVC_L2_G31 | Dem | MCI | 0 | 0 | Mann-Whitney U | **0.029** |
| DVC_L2_G31 | Dem | HCs | 0 | 0 | Mann-Whitney U | **0.005** |
| DVC_L2_G31 | MCI | HCs | 0 | 0 | Mann-Whitney U | 0.534 |
| DVC_L2_G32 | Dem | MCI | 0 | 0 | Mann-Whitney U | **0.024** |
| DVC_L2_G32 | Dem | HCs | 0 | 0 | Mann-Whitney U | **0.003** |
| DVC_L2_G32 | MCI | HCs | 0 | 0 | Mann-Whitney U | 0.326 |
| DVC_L2_G33 | Dem | MCI | 0 | 0 | Mann-Whitney U | **0.035** |
| DVC_L2_G33 | Dem | HCs | 0 | 0 | Mann-Whitney U | **0.002** |
| DVC_L2_G33 | MCI | HCs | 0 | 0 | Mann-Whitney U | 0.142 |
| FAZ_Area | Dem | MCI | 0 | 0 | Mann-Whitney U | 1.000 |
| FAZ_Area | Dem | HCs | 0 | 0 | Mann-Whitney U | 0.866 |
| FAZ_Area | MCI | HCs | 0 | 0 | Mann-Whitney U | 0.820 |
| Perimeter | Dem | MCI | 1 | 0 | Dunnett | 1.000 |
| Perimeter | Dem | HCs | 1 | 0 | Dunnett | 1.000 |
| Perimeter | MCI | HCs | 1 | 0 | Dunnett | 1.000 |
| AcircularityIndex | Dem | MCI | 0 | 0 | Mann-Whitney U | 0.235 |
| AcircularityIndex | Dem | HCs | 0 | 0 | Mann-Whitney U | 0.541 |
| AcircularityIndex | MCI | HCs | 0 | 0 | Mann-Whitney U | 0.345 |
| FD_300_Area_Density | Dem | MCI | 0 | 0 | Mann-Whitney U | 0.069 |
| FD_300_Area_Density | Dem | HCs | 0 | 0 | Mann-Whitney U | **0.004** |
| FD_300_Area_Density | MCI | HCs | 0 | 0 | Mann-Whitney U | 0.081 |
| FD_300_Length_Density | Dem | MCI | 0 | 0 | Mann-Whitney U | **0.001** |
| FD_300_Length_Density | Dem | HCs | 0 | 0 | Mann-Whitney U | **<0.001** |
| FD_300_Length_Density | MCI | HCs | 0 | 0 | Mann-Whitney U | **0.026** |
| FoveaInnRet_Thickness | Dem | MCI | 0 | 0 | Mann-Whitney U | 0.443 |
| FoveaInnRet_Thickness | Dem | HCs | 0 | 0 | Mann-Whitney U | 0.329 |
| FoveaInnRet_Thickness | MCI | HCs | 0 | 0 | Mann-Whitney U | 0.682 |
| InnRet_Thk_ParaFovea | Dem | MCI | 0 | 0 | Mann-Whitney U | 0.080 |
| InnRet_Thk_ParaFovea | Dem | HCs | 0 | 0 | Mann-Whitney U | **0.016** |
| InnRet_Thk_ParaFovea | MCI | HCs | 0 | 0 | Mann-Whitney U | 0.303 |
| InnRet_Thk_Para_S_Hemisphere | Dem | MCI | 0 | 0 | Mann-Whitney U | 0.080 |
| InnRet_Thk_Para_S_Hemisphere | Dem | HCs | 0 | 0 | Mann-Whitney U | **0.012** |
| InnRet_Thk_Para_S_Hemisphere | MCI | HCs | 0 | 0 | Mann-Whitney U | 0.337 |
| InnRet_Thk_Para_I_Hemisphere | Dem | MCI | 0 | 0 | Mann-Whitney U | 0.159 |
| InnRet_Thk_Para_I_Hemisphere | Dem | HCs | 0 | 0 | Mann-Whitney U | **0.023** |
| InnRet_Thk_Para_I_Hemisphere | MCI | HCs | 0 | 0 | Mann-Whitney U | 0.173 |
| InnRet_Thk_Para_Tempo | Dem | MCI | 0 | 0 | Mann-Whitney U | 0.263 |
| InnRet_Thk_Para_Tempo | Dem | HCs | 0 | 0 | Mann-Whitney U | 0.061 |
| InnRet_Thk_Para_Tempo | MCI | HCs | 0 | 0 | Mann-Whitney U | 0.254 |
| InnRet_Thk_Para_Superior | Dem | MCI | 0 | 0 | Mann-Whitney U | 0.176 |
| InnRet_Thk_Para_Superior | Dem | HCs | 0 | 0 | Mann-Whitney U | **0.029** |
| InnRet_Thk_Para_Superior | MCI | HCs | 0 | 0 | Mann-Whitney U | 0.333 |
| InnRet_Thk_Para_Nasal | Dem | HCs | 1 | 1 | pairwise_tukeyhsd | **0.032** |
| InnRet_Thk_Para_Nasal | Dem | MCI | 1 | 1 | pairwise_tukeyhsd | 0.204 |
| InnRet_Thk_Para_Nasal | HCs | MCI | 1 | 1 | pairwise_tukeyhsd | 0.480 |
| InnRet_Thk_Para_Inferior | Dem | MCI | 0 | 0 | Mann-Whitney U | 0.306 |
| InnRet_Thk_Para_Inferior | Dem | HCs | 0 | 0 | Mann-Whitney U | **0.038** |
| InnRet_Thk_Para_Inferior | MCI | HCs | 0 | 0 | Mann-Whitney U | 0.102 |
| InnRet_Thk_PeriFovea | Dem | MCI | 0 | 0 | Mann-Whitney U | 0.131 |
| InnRet_Thk_PeriFovea | Dem | HCs | 0 | 0 | Mann-Whitney U | **0.016** |
| InnRet_Thk_PeriFovea | MCI | HCs | 0 | 0 | Mann-Whitney U | 0.203 |
| InnRet_Thk_Peri_S_Hemisphere | Dem | MCI | 0 | 0 | Mann-Whitney U | 0.099 |
| InnRet_Thk_Peri_S_Hemisphere | Dem | HCs | 0 | 0 | Mann-Whitney U | **0.026** |
| InnRet_Thk_Peri_S_Hemisphere | MCI | HCs | 0 | 0 | Mann-Whitney U | 0.421 |
| InnRet_Thk_Peri_I_Hemisphere | Dem | MCI | 0 | 0 | Mann-Whitney U | 0.228 |
| InnRet_Thk_Peri_I_Hemisphere | Dem | HCs | 0 | 0 | Mann-Whitney U | **0.022** |
| InnRet_Thk_Peri_I_Hemisphere | MCI | HCs | 0 | 0 | Mann-Whitney U | 0.117 |
| InnRet_Thk_Peri_Tempo | Dem | MCI | 0 | 0 | Mann-Whitney U | 0.550 |
| InnRet_Thk_Peri_Tempo | Dem | HCs | 0 | 0 | Mann-Whitney U | 0.174 |
| InnRet_Thk_Peri_Tempo | MCI | HCs | 0 | 0 | Mann-Whitney U | 0.213 |
| InnRet_Thk_Peri_Superior | Dem | MCI | 0 | 0 | Mann-Whitney U | 0.180 |
| InnRet_Thk_Peri_Superior | Dem | HCs | 0 | 0 | Mann-Whitney U | 0.051 |
| InnRet_Thk_Peri_Superior | MCI | HCs | 0 | 0 | Mann-Whitney U | 0.335 |
| InnRet_Thk_Peri_Nasal | Dem | MCI | 0 | 0 | Mann-Whitney U | 0.082 |
| InnRet_Thk_Peri_Nasal | Dem | HCs | 0 | 0 | Mann-Whitney U | **0.017** |
| InnRet_Thk_Peri_Nasal | MCI | HCs | 0 | 0 | Mann-Whitney U | 0.376 |
| InnRet_Thk_Peri_Inferior | Dem | MCI | 0 | 0 | Mann-Whitney U | 0.443 |
| InnRet_Thk_Peri_Inferior | Dem | HCs | 0 | 0 | Mann-Whitney U | 0.078 |
| InnRet_Thk_Peri_Inferior | MCI | HCs | 0 | 0 | Mann-Whitney U | 0.125 |
| FoveaInnRet_Volumn | Dem | MCI | 0 | 0 | Mann-Whitney U | 0.365 |
| FoveaInnRet_Volumn | Dem | HCs | 0 | 0 | Mann-Whitney U | 0.224 |
| FoveaInnRet_Volumn | MCI | HCs | 0 | 0 | Mann-Whitney U | 0.493 |
| InnRet_Vol_ParaFovea | Dem | MCI | 0 | 0 | Mann-Whitney U | **0.025** |
| InnRet_Vol_ParaFovea | Dem | HCs | 0 | 0 | Mann-Whitney U | **0.001** |
| InnRet_Vol_ParaFovea | MCI | HCs | 0 | 0 | Mann-Whitney U | 0.147 |
| InnRet_Vol_Para_S_Hemisphere | Dem | MCI | 0 | 0 | Mann-Whitney U | **0.037** |
| InnRet_Vol_Para_S_Hemisphere | Dem | HCs | 0 | 0 | Mann-Whitney U | **0.001** |
| InnRet_Vol_Para_S_Hemisphere | MCI | HCs | 0 | 0 | Mann-Whitney U | 0.166 |
| InnRet_Vol_Para_I_Hemisphere | Dem | MCI | 0 | 0 | Mann-Whitney U | **0.040** |
| InnRet_Vol_Para_I_Hemisphere | Dem | HCs | 0 | 0 | Mann-Whitney U | **0.003** |
| InnRet_Vol_Para_I_Hemisphere | MCI | HCs | 0 | 0 | Mann-Whitney U | 0.130 |
| InnRet_Vol_Para_Tempo | Dem | MCI | 0 | 0 | Mann-Whitney U | **0.044** |
| InnRet_Vol_Para_Tempo | Dem | HCs | 0 | 0 | Mann-Whitney U | **0.002** |
| InnRet_Vol_Para_Tempo | MCI | HCs | 0 | 0 | Mann-Whitney U | 0.114 |
| InnRet_Vol_Para_Superior | Dem | MCI | 0 | 0 | Mann-Whitney U | 0.072 |
| InnRet_Vol_Para_Superior | Dem | HCs | 0 | 0 | Mann-Whitney U | **0.003** |
| InnRet_Vol_Para_Superior | MCI | HCs | 0 | 0 | Mann-Whitney U | 0.158 |
| InnRet_Vol_Para_Nasal | Dem | MCI | 0 | 0 | Mann-Whitney U | 0.258 |
| InnRet_Vol_Para_Nasal | Dem | HCs | 0 | 0 | Mann-Whitney U | 0.057 |
| InnRet_Vol_Para_Nasal | MCI | HCs | 0 | 0 | Mann-Whitney U | 0.305 |
| InnRet_Vol_Para_Inferior | Dem | MCI | 0 | 0 | Mann-Whitney U | 0.092 |
| InnRet_Vol_Para_Inferior | Dem | HCs | 0 | 0 | Mann-Whitney U | **0.006** |
| InnRet_Vol_Para_Inferior | MCI | HCs | 0 | 0 | Mann-Whitney U | 0.086 |
| InnRet_Vol_PeriFovea | Dem | MCI | 0 | 0 | Mann-Whitney U | **0.043** |
| InnRet_Vol_PeriFovea | Dem | HCs | 0 | 0 | Mann-Whitney U | **0.003** |
| InnRet_Vol_PeriFovea | MCI | HCs | 0 | 0 | Mann-Whitney U | 0.157 |
| InnRet_Vol_Peri_S_Hemisphere | Dem | MCI | 0 | 0 | Mann-Whitney U | **0.027** |
| InnRet_Vol_Peri_S_Hemisphere | Dem | HCs | 0 | 0 | Mann-Whitney U | **0.004** |
| InnRet_Vol_Peri_S_Hemisphere | MCI | HCs | 0 | 0 | Mann-Whitney U | 0.403 |
| InnRet_Vol_Peri_I_Hemisphere | Dem | MCI | 0 | 0 | Mann-Whitney U | 0.128 |
| InnRet_Vol_Peri_I_Hemisphere | Dem | HCs | 0 | 0 | Mann-Whitney U | **0.009** |
| InnRet_Vol_Peri_I_Hemisphere | MCI | HCs | 0 | 0 | Mann-Whitney U | 0.104 |
| InnRet_Vol_Peri_Tempo | Dem | MCI | 0 | 0 | Mann-Whitney U | 0.218 |
| InnRet_Vol_Peri_Tempo | Dem | HCs | 0 | 0 | Mann-Whitney U | **0.040** |
| InnRet_Vol_Peri_Tempo | MCI | HCs | 0 | 0 | Mann-Whitney U | 0.163 |
| InnRet_Vol_Peri_Superior | Dem | MCI | 0 | 0 | Mann-Whitney U | 0.051 |
| InnRet_Vol_Peri_Superior | Dem | HCs | 0 | 0 | Mann-Whitney U | **0.006** |
| InnRet_Vol_Peri_Superior | MCI | HCs | 0 | 0 | Mann-Whitney U | 0.244 |
| InnRet_Vol_Peri_Nasal | Dem | MCI | 0 | 0 | Mann-Whitney U | 0.082 |
| InnRet_Vol_Peri_Nasal | Dem | HCs | 0 | 0 | Mann-Whitney U | **0.010** |
| InnRet_Vol_Peri_Nasal | MCI | HCs | 0 | 0 | Mann-Whitney U | 0.327 |
| InnRet_Vol_Peri_Inferior | Dem | MCI | 0 | 0 | Mann-Whitney U | 0.248 |
| InnRet_Vol_Peri_Inferior | Dem | HCs | 0 | 0 | Mann-Whitney U | **0.043** |
| InnRet_Vol_Peri_Inferior | MCI | HCs | 0 | 0 | Mann-Whitney U | 0.134 |
| FoveaFullRet_Thickness | Dem | MCI | 0 | 0 | Mann-Whitney U | 0.724 |
| FoveaFullRet_Thickness | Dem | HCs | 0 | 0 | Mann-Whitney U | 0.645 |
| FoveaFullRet_Thickness | MCI | HCs | 0 | 0 | Mann-Whitney U | 0.866 |
| FullRet_Thk_ParaFovea | Dem | MCI | 0 | 0 | Mann-Whitney U | 0.252 |
| FullRet_Thk_ParaFovea | Dem | HCs | 0 | 0 | Mann-Whitney U | 0.123 |
| FullRet_Thk_ParaFovea | MCI | HCs | 0 | 0 | Mann-Whitney U | 0.617 |
| FullRet_Thk_Para_S_Hemisphere | Dem | MCI | 0 | 0 | Mann-Whitney U | 0.198 |
| FullRet_Thk_Para_S_Hemisphere | Dem | HCs | 0 | 0 | Mann-Whitney U | 0.107 |
| FullRet_Thk_Para_S_Hemisphere | MCI | HCs | 0 | 0 | Mann-Whitney U | 0.653 |
| FullRet_Thk_Para_I_Hemisphere | Dem | MCI | 0 | 0 | Mann-Whitney U | 0.277 |
| FullRet_Thk_Para_I_Hemisphere | Dem | HCs | 0 | 0 | Mann-Whitney U | 0.235 |
| FullRet_Thk_Para_I_Hemisphere | MCI | HCs | 0 | 0 | Mann-Whitney U | 0.947 |
| FullRet_Thk_Para_Tempo | Dem | MCI | 0 | 0 | Mann-Whitney U | 0.219 |
| FullRet_Thk_Para_Tempo | Dem | HCs | 0 | 0 | Mann-Whitney U | 0.211 |
| FullRet_Thk_Para_Tempo | MCI | HCs | 0 | 0 | Mann-Whitney U | 0.997 |
| FullRet_Thk_Para_Superior | Dem | MCI | 0 | 0 | Mann-Whitney U | 0.270 |
| FullRet_Thk_Para_Superior | Dem | HCs | 0 | 0 | Mann-Whitney U | 0.100 |
| FullRet_Thk_Para_Superior | MCI | HCs | 0 | 0 | Mann-Whitney U | 0.450 |
| FullRet_Thk_Para_Nasal | Dem | MCI | 0 | 0 | Mann-Whitney U | 0.142 |
| FullRet_Thk_Para_Nasal | Dem | HCs | 0 | 0 | Mann-Whitney U | 0.138 |
| FullRet_Thk_Para_Nasal | MCI | HCs | 0 | 0 | Mann-Whitney U | 0.953 |
| FullRet_Thk_Para_Inferior | Dem | MCI | 0 | 0 | Mann-Whitney U | 0.520 |
| FullRet_Thk_Para_Inferior | Dem | HCs | 0 | 0 | Mann-Whitney U | 0.375 |
| FullRet_Thk_Para_Inferior | MCI | HCs | 0 | 0 | Mann-Whitney U | 0.736 |
| FullRet_Thk_PeriFovea | Dem | MCI | 0 | 0 | Mann-Whitney U | 0.450 |
| FullRet_Thk_PeriFovea | Dem | HCs | 0 | 0 | Mann-Whitney U | 0.192 |
| FullRet_Thk_PeriFovea | MCI | HCs | 0 | 0 | Mann-Whitney U | 0.476 |
| FullRet_Thk_Peri_S_Hemisphere | Dem | MCI | 0 | 0 | Mann-Whitney U | 0.450 |
| FullRet_Thk_Peri_S_Hemisphere | Dem | HCs | 0 | 0 | Mann-Whitney U | 0.173 |
| FullRet_Thk_Peri_S_Hemisphere | MCI | HCs | 0 | 0 | Mann-Whitney U | 0.428 |
| FullRet_Thk_Peri_I_Hemisphere | Dem | MCI | 0 | 0 | Mann-Whitney U | 0.673 |
| FullRet_Thk_Peri_I_Hemisphere | Dem | HCs | 0 | 0 | Mann-Whitney U | 0.268 |
| FullRet_Thk_Peri_I_Hemisphere | MCI | HCs | 0 | 0 | Mann-Whitney U | 0.376 |
| FullRet_Thk_Peri_Tempo | Dem | MCI | 0 | 0 | Mann-Whitney U | 0.355 |
| FullRet_Thk_Peri_Tempo | Dem | HCs | 0 | 0 | Mann-Whitney U | 0.127 |
| FullRet_Thk_Peri_Tempo | MCI | HCs | 0 | 0 | Mann-Whitney U | 0.460 |
| FullRet_Thk_Peri_Superior | Dem | MCI | 0 | 0 | Mann-Whitney U | 0.453 |
| FullRet_Thk_Peri_Superior | Dem | HCs | 0 | 0 | Mann-Whitney U | 0.222 |
| FullRet_Thk_Peri_Superior | MCI | HCs | 0 | 0 | Mann-Whitney U | 0.482 |
| FullRet_Thk_Peri_Nasal | Dem | MCI | 0 | 0 | Mann-Whitney U | 0.430 |
| FullRet_Thk_Peri_Nasal | Dem | HCs | 0 | 0 | Mann-Whitney U | 0.162 |
| FullRet_Thk_Peri_Nasal | MCI | HCs | 0 | 0 | Mann-Whitney U | 0.367 |
| FullRet_Thk_Peri_Inferior | Dem | MCI | 0 | 0 | Mann-Whitney U | 0.936 |
| FullRet_Thk_Peri_Inferior | Dem | HCs | 0 | 0 | Mann-Whitney U | 0.506 |
| FullRet_Thk_Peri_Inferior | MCI | HCs | 0 | 0 | Mann-Whitney U | 0.291 |
| FoveaFullRet_Volumn | Dem | MCI | 0 | 0 | Mann-Whitney U | 0.430 |
| FoveaFullRet_Volumn | Dem | HCs | 0 | 0 | Mann-Whitney U | 0.243 |
| FoveaFullRet_Volumn | MCI | HCs | 0 | 0 | Mann-Whitney U | 0.531 |
| FullRet_Vol_ParaFovea | Dem | MCI | 0 | 0 | Mann-Whitney U | 0.197 |
| FullRet_Vol_ParaFovea | Dem | HCs | 0 | 0 | Mann-Whitney U | 0.051 |
| FullRet_Vol_ParaFovea | MCI | HCs | 0 | 0 | Mann-Whitney U | 0.358 |
| FullRet_Vol_Para_S_Hemisphere | Dem | MCI | 0 | 0 | Mann-Whitney U | 0.221 |
| FullRet_Vol_Para_S_Hemisphere | Dem | HCs | 0 | 0 | Mann-Whitney U | **0.049** |
| FullRet_Vol_Para_S_Hemisphere | MCI | HCs | 0 | 0 | Mann-Whitney U | 0.275 |
| FullRet_Vol_Para_I_Hemisphere | Dem | MCI | 0 | 0 | Mann-Whitney U | 0.174 |
| FullRet_Vol_Para_I_Hemisphere | Dem | HCs | 0 | 0 | Mann-Whitney U | 0.062 |
| FullRet_Vol_Para_I_Hemisphere | MCI | HCs | 0 | 0 | Mann-Whitney U | 0.528 |
| FullRet_Vol_Para_Tempo | Dem | MCI | 0 | 0 | Mann-Whitney U | 0.084 |
| FullRet_Vol_Para_Tempo | Dem | HCs | 0 | 0 | Mann-Whitney U | **0.015** |
| FullRet_Vol_Para_Tempo | MCI | HCs | 0 | 0 | Mann-Whitney U | 0.288 |
| FullRet_Vol_Para_Superior | Dem | MCI | 0 | 0 | Mann-Whitney U | 0.453 |
| FullRet_Vol_Para_Superior | Dem | HCs | 0 | 0 | Mann-Whitney U | 0.115 |
| FullRet_Vol_Para_Superior | MCI | HCs | 0 | 0 | Mann-Whitney U | 0.252 |
| FullRet_Vol_Para_Nasal | Dem | MCI | 0 | 0 | Mann-Whitney U | 0.301 |
| FullRet_Vol_Para_Nasal | Dem | HCs | 0 | 0 | Mann-Whitney U | 0.235 |
| FullRet_Vol_Para_Nasal | MCI | HCs | 0 | 0 | Mann-Whitney U | 0.870 |
| FullRet_Vol_Para_Inferior | Dem | MCI | 0 | 0 | Mann-Whitney U | 0.392 |
| FullRet_Vol_Para_Inferior | Dem | HCs | 0 | 0 | Mann-Whitney U | 0.276 |
| FullRet_Vol_Para_Inferior | MCI | HCs | 0 | 0 | Mann-Whitney U | 0.723 |
| FullRet_Vol_PeriFovea | Dem | MCI | 0 | 0 | Mann-Whitney U | 0.550 |
| FullRet_Vol_PeriFovea | Dem | HCs | 0 | 0 | Mann-Whitney U | 0.092 |
| FullRet_Vol_PeriFovea | MCI | HCs | 0 | 0 | Mann-Whitney U | 0.129 |
| FullRet_Vol_Peri_S_Hemisphere | Dem | MCI | 0 | 0 | Mann-Whitney U | 0.252 |
| FullRet_Vol_Peri_S_Hemisphere | Dem | HCs | 0 | 0 | Mann-Whitney U | **0.028** |
| FullRet_Vol_Peri_S_Hemisphere | MCI | HCs | 0 | 0 | Mann-Whitney U | 0.196 |
| FullRet_Vol_Peri_I_Hemisphere | Dem | MCI | 0 | 0 | Mann-Whitney U | 0.962 |
| FullRet_Vol_Peri_I_Hemisphere | Dem | HCs | 0 | 0 | Mann-Whitney U | 0.193 |
| FullRet_Vol_Peri_I_Hemisphere | MCI | HCs | 0 | 0 | Mann-Whitney U | 0.090 |
| FullRet_Vol_Peri_Tempo | Dem | MCI | 0 | 0 | Mann-Whitney U | 0.402 |
| FullRet_Vol_Peri_Tempo | Dem | HCs | 0 | 0 | Mann-Whitney U | **0.036** |
| FullRet_Vol_Peri_Tempo | MCI | HCs | 0 | 0 | Mann-Whitney U | 0.086 |
| FullRet_Vol_Peri_Superior | Dem | MCI | 0 | 0 | Mann-Whitney U | 0.389 |
| FullRet_Vol_Peri_Superior | Dem | HCs | 0 | 0 | Mann-Whitney U | 0.095 |
| FullRet_Vol_Peri_Superior | MCI | HCs | 0 | 0 | Mann-Whitney U | 0.259 |
| FullRet_Vol_Peri_Nasal | Dem | MCI | 0 | 0 | Mann-Whitney U | 0.386 |
| FullRet_Vol_Peri_Nasal | Dem | HCs | 0 | 0 | Mann-Whitney U | 0.140 |
| FullRet_Vol_Peri_Nasal | MCI | HCs | 0 | 0 | Mann-Whitney U | 0.312 |
| FullRet_Vol_Peri_Inferior | Dem | MCI | 0 | 0 | Mann-Whitney U | 0.469 |
| FullRet_Vol_Peri_Inferior | Dem | HCs | 0 | 0 | Mann-Whitney U | 0.656 |
| FullRet_Vol_Peri_Inferior | MCI | HCs | 0 | 0 | Mann-Whitney U | 0.103 |
| FoveaRPE_Elevation_Height | Dem | MCI | 0 | 0 | Mann-Whitney U | 0.367 |
| FoveaRPE_Elevation_Height | Dem | HCs | 0 | 0 | Mann-Whitney U | 0.119 |
| FoveaRPE_Elevation_Height | MCI | HCs | 0 | 0 | Mann-Whitney U | 0.387 |
| RPE_Elev_ParaFovea_Tempo | Dem | MCI | 0 | 0 | Mann-Whitney U | 0.589 |
| RPE_Elev_ParaFovea_Tempo | Dem | HCs | 0 | 0 | Mann-Whitney U | 0.950 |
| RPE_Elev_ParaFovea_Tempo | MCI | HCs | 0 | 0 | Mann-Whitney U | 0.417 |
| RPE_Elev_Para_Superior | Dem | MCI | 0 | 0 | Mann-Whitney U | 0.607 |
| RPE_Elev_Para_Superior | Dem | HCs | 0 | 0 | Mann-Whitney U | 0.179 |
| RPE_Elev_Para_Superior | MCI | HCs | 0 | 0 | Mann-Whitney U | 0.211 |
| RPE_Elev_Para_Nasal | Dem | MCI | 0 | 0 | Mann-Whitney U | 0.193 |
| RPE_Elev_Para_Nasal | Dem | HCs | 0 | 0 | Mann-Whitney U | 0.220 |
| RPE_Elev_Para_Nasal | MCI | HCs | 0 | 0 | Mann-Whitney U | 0.867 |
| RPE_Elev_Para_Inferior | Dem | MCI | 0 | 0 | Mann-Whitney U | 0.111 |
| RPE_Elev_Para_Inferior | Dem | HCs | 0 | 0 | Mann-Whitney U | **0.017** |
| RPE_Elev_Para_Inferior | MCI | HCs | 0 | 0 | Mann-Whitney U | 0.116 |
| RPE_Elev_PeriFovea_Tempo | Dem | MCI | 0 | 0 | Mann-Whitney U | 0.915 |
| RPE_Elev_PeriFovea_Tempo | Dem | HCs | 0 | 0 | Mann-Whitney U | 0.294 |
| RPE_Elev_PeriFovea_Tempo | MCI | HCs | 0 | 0 | Mann-Whitney U | 0.079 |
| RPE_Elev_Peri_Superior | Dem | MCI | 0 | 0 | Mann-Whitney U | 0.386 |
| RPE_Elev_Peri_Superior | Dem | HCs | 0 | 0 | Mann-Whitney U | 0.708 |
| RPE_Elev_Peri_Superior | MCI | HCs | 0 | 0 | Mann-Whitney U | 0.430 |
| RPE_Elev_Peri_Nasal | Dem | MCI | 0 | 0 | Mann-Whitney U | **0.036** |
| RPE_Elev_Peri_Nasal | Dem | HCs | 0 | 0 | Mann-Whitney U | 0.184 |
| RPE_Elev_Peri_Nasal | MCI | HCs | 0 | 0 | Mann-Whitney U | 0.247 |
| RPE_Elev_Peri_Inferior | Dem | MCI | 0 | 0 | Mann-Whitney U | 0.411 |
| RPE_Elev_Peri_Inferior | Dem | HCs | 0 | 0 | Mann-Whitney U | 0.771 |
| RPE_Elev_Peri_Inferior | MCI | HCs | 0 | 0 | Mann-Whitney U | 0.408 |

# Details of Parameters

GCC related parameters investigates Average, Superior (s), Inferior (I), I subtracted from S (S_I) thicknesses in the following layers definitions: 1) $Inner\_Retina$ (InnRet: ILM to 10 μm below OPL (1)), 2) $Full\_Retina$ (FullRet: ILM to RPE/BRM complex (1)), and 3) $Outer\_Retina$ (OutRet:10 μm below OPL to RPE/BRM complex (2) (3)). Additionally, global loss volume (GLV), focal loss volume (FLV), and root mean square (RMS) (4).

ONH parameters were categorized into 1) optic disc analysis and 2) peripapillary RNFL (pRNFL) thickness. Parameters based on optic disc analysis: optic disc area/volume (ONH_DiscArea, ONH_Disc_Volume), cup area/volume (ONH_CupArea, ONH_CupVolume), rim area/volume (ONH_RimArea, ONH_RimVolume), and cup-to-disc (area: ONH_Area_C_D_ratio, horizontal: ONH_H_C_D_ratio, vertical: ONH_V_C_D_ratio) ratios. Parameters based on pRNFL thickness: hemisphere S (S-Hemi) and hemisphere I (I-Hemi), and average pRNFL thickness (5). Additionally, other pRNFL regions included quadrants-based Superior (S), Inferior (I), Temporal (T), Nasal (N), as well as supertemporal (ST), superonasal (SN), inferotemporal (IT), inferonasal (IN), nasal upper (NU), nasal lower (NL), temporal upper (TU), and temporal lower (TL).

Macula_3mm parameters target retinal thickness/volume changes in various quadrants and layers (ILM to IPL, ILM to RPE, ILM to BRM, and RPE to BRM). The thickness/volume analyses for distinct layers definitions include $Center\_1$ (1mm foveal ring), quadrants (S, I, T, N) in the 3mm ring excluding 1mm (1minus3), S and I hemispheres in 1minus3, and combined hemispheres (All_1minus3). Additionally, the thickness/volume analyses for distinct layers definitions include S and I hemispheres field (including $Center\_1$), combined hemispheres field (All_field).

The retina map measures retinal OCT thickness (μm) and volume ($mm^{3}$) around the fovea using 1 mm, 3 mm, and 5 mm circles. The foveal thickness/volume was denoted by $Fovea$, whilst quadrants of ($S_{3}, I_{3}, T_{3}, N_{3}$) and ($S_{5}, I_{5}, T_{5}, N_{5}$) corresponded to parafovea (Para) and perifovea (Peri), respectively. Also, hemispheres $S\_Hemi_{3}$ (Para_S_Hemisphere) and $I\_Hemi_{3}$ (Para_I_Hemisphere) represented 3 mm rings, while $S\_Hemi_{5}$ (Peri_S_Hemisphere) and $I\_Hemi_{5}$ (Peri_I_Hemisphere) indicated 5 mm rings. Additionally, $parafovea$ was the combined $S\_Hemi_{3}$ and $I\_Hemi_{3}$, whilst $perifovea$was the combined $S\_Hemi_{5}$ and $I\_Hemi_{5}$. The retina map ($\mu m/mm^{3}$) was calculated in $Inner\_Retina$ and $Full\_Retina$ layers, similarly to GCC parameters, as well as in RPE thickness (Elevation).

Retina3DFlowDensity investigates retinal VD changes using quadrants (S, I, T, N), hemispheres (S and I), not following Early Treatment of Diabetic Retinopathy Study (ETDRS) ($Whole\_Image$), both hemispheres combined (Whole_ETDRS), $Whole Image$ in $S\_Hemi_{3}$ and$I\_Hemi_{3}$, the 3x3 grid G, and $FD\_300$ ($Area\_Density$ and $Length\_Density$). Other Retina3DFlowDensity parameters include $Fovea$, $FAZ\_Area$, $FAZ\_Perim$ (Perimeter) (6), and $AcirIndx$(AcircularityIndex) (7). Importantly, VD was computed for various retinal layers including A) superficial vascular complex (SVC) from ILM to 10 μm above the IPL, B) deep vascular complex (DVC) from 10 μm above IPL to 10 μm below OPL, C) inner vascular complex (IVC) from ILM to 10 μm below OPL.

# References

1. Hanumunthadu D, Keane PA, Balaskas K, Dubis AM, Kalitzeos A, Michaelides M, et al. Agreement between spectral-domain and swept-source optical coherence tomography retinal thickness measurements in macular and retinal disease. Ophthalmology and therapy. 2021;10:913-22.

2. Venkatesh R, Sinha S, Gangadharaiah D, Gadde SG, Mohan A, Shetty R, et al. Retinal structural-vascular-functional relationship using optical coherence tomography and optical coherence tomography–angiography in myopia. Eye and Vision. 2019;6(1):1-12.

3. Ye J, Wang M, Shen M, Huang S, Xue A, Lin J, et al. Deep retinal capillary plexus decreasing correlated with the outer retinal layer alteration and visual acuity impairment in pathological myopia. Investigative ophthalmology & visual science. 2020;61(4):45-.

4. Rao HL, Zangwill LM, Weinreb RN, Sample PA, Alencar LM, Medeiros FA. Comparison of different spectral domain optical coherence tomography scanning areas for glaucoma diagnosis. Ophthalmology. 2010;117(9):1692-9. e1.

5. González-García AO, Vizzeri G, Bowd C, Medeiros FA, Zangwill LM, Weinreb RN. Reproducibility of RTVue retinal nerve fiber layer thickness and optic disc measurements and agreement with Stratus optical coherence tomography measurements. American Journal of Ophthalmology. 2009;147(6):1067-74. e1.

6. Mo S, Krawitz B, Efstathiadis E, Geyman L, Weitz R, Chui TY, et al. Imaging foveal microvasculature: optical coherence tomography angiography versus adaptive optics scanning light ophthalmoscope fluorescein angiography. Investigative ophthalmology & visual science. 2016;57(9):OCT130-OCT40.

7. Tam J, Dhamdhere KP, Tiruveedhula P, Manzanera S, Barez S, Bearse MA, et al. Disruption of the retinal parafoveal capillary network in type 2 diabetes before the onset of diabetic retinopathy. Investigative Ophthalmology & Visual Science. 2011;52(12):9257-66.
