# Supplementary material for: Retinal microvascular density and inner thickness in Alzheimer’s disease and mild cognitive impairment
Source: Front Aging Neurosci. 2025 Feb 28;17:1477008. doi: 10.3389/fnagi.2025.1477008 (PMC11906703; doi:10.3389/fnagi.2025.1477008)
Supplement: Supplementary file 4 [file Table_4.DOCX]

Supplementary Material D

# Generalized Linear Model (Target Variable: MMSE) Results

The statistically significant parameters have their p-value in **Bold** font. We present the results of generalized linear model (GLM) analysis performed by SPSS software with MMSE as a dependent variable. Starting with GLM model information followed by statistical details of variables under “Continuous Variable Information”. Moreover, statistical p-value significance shown under “Tests of Model Effects”. Finally, 95% Confidence Interval as well as statistical Hypothesis Test of Chi-Square is presented under “Parameter Estimates”. Importantly, the detailed description of all parameters’ names could be found under “Details of Parameters” section.

## Model Information

| Dependent Variable | MMSE |
| --- | --- |
| Probability Distribution | Tweedie (1.5) |
| Link Function | Log |
| Offset Variable | AgePerToday |

## Continuous Variable Information

|  | | N | Minimum | Maximum | Mean | Std. Deviation |
| --- | --- | --- | --- | --- | --- | --- |
| Dependent Variable | MMSE | 357 | 10 | 30 | 25.95 | 4.025 |
| Covariate | GCC_Inner_Retina_Average | 357 | 60.95 | 146.16 | 96.2255 | 10.49492 |
|  | GCC_Superior_Avg | 357 | 58.51 | 152.26 | 95.7850 | 10.83188 |
|  | GCC_Inferior_Avg | 357 | 62.00 | 140.04 | 96.6758 | 10.78998 |
|  | GCC_S_I_Avg | 357 | -18.29 | 19.40 | -.8755 | 4.98499 |
|  | GCC_FLV | 357 | .000 | 21.964 | 1.40788 | 2.310063 |
|  | GCC_GLV | 357 | .000 | 33.443 | 4.20881 | 5.238621 |
|  | GCC_RMS | 357 | .03 | 1.03 | .0912 | .07971 |
|  | GCC_Full_Retina_Average | 357 | 221.52 | 354.47 | 275.3752 | 15.96806 |
|  | GCC_Superior_Avg.1 | 357 | 42.77 | 286.26 | 95.9495 | 22.59594 |
|  | GCC_Inferior_Avg.1 | 357 | 46.43 | 287.46 | 99.9005 | 22.52670 |
|  | GCC_S_I_Avg.1 | 357 | -38.85 | 50.38 | -.4122 | 8.12804 |
|  | GCC_Outer_Retina_Average | 357 | 140.91 | 222.15 | 179.1355 | 9.43361 |
|  | GCC_Superior_Avg.2 | 357 | 42.77 | 185.88 | 97.3454 | 15.04068 |
|  | GCC_Inferior_Avg.2 | 357 | 62.00 | 189.77 | 100.4287 | 19.03752 |
|  | GCC_S_I_Avg.2 | 357 | -43.23 | 19.40 | -1.4717 | 6.03400 |
|  | ONH_DiscArea | 357 | .00 | 3.71 | 2.0090 | .46306 |
|  | ONH_Area_C_D_ratio | 357 | .00 | .86 | .2949 | .17401 |
|  | ONH_H_C_D_ratio | 357 | .00 | 1.00 | .5431 | .23057 |
|  | ONH_V_C_D_ratio | 357 | .00 | .99 | .4733 | .21529 |
|  | ONH_CupArea | 357 | .00 | 2.01 | .6132 | .40635 |
|  | ONH_RimArea | 357 | .00 | 2.88 | 1.3751 | .40772 |
|  | ONH_RimVolume | 357 | .000 | .490 | .14857 | .077161 |
|  | ONH_Disc_Volume | 357 | .000 | 1.168 | .32482 | .167875 |
|  | ONH_CupVolume | 357 | .000 | .842 | .10373 | .134492 |
|  | ONH_Avg_RNFL | 357 | 56.21 | 123.78 | 96.8978 | 10.70932 |
|  | ONH_Sup_RNFL | 357 | 60.53 | 125.76 | 98.1720 | 11.32904 |
|  | ONH_Inf_RNFL | 357 | 51.90 | 123.85 | 95.8780 | 11.14183 |
|  | ONH_Tempo | 357 | 35.39 | 111.94 | 72.7199 | 10.40557 |
|  | ONH_Superior | 357 | 70.31 | 175.84 | 117.2317 | 15.07685 |
|  | ONH_Nasal | 357 | 43.23 | 135.92 | 76.0089 | 11.94326 |
|  | ONH_Inferior | 357 | 57.92 | 160.08 | 121.7851 | 15.80073 |
|  | ONH_RNFL_TU | 357 | 43.048 | 149.563 | 79.23490 | 14.054446 |
|  | ONH_ST | 357 | 69.019 | 185.052 | 128.81629 | 17.458863 |
|  | ONH_SN | 357 | 55.840 | 149.790 | 105.65856 | 16.778563 |
|  | ONH_NU | 357 | 39.178 | 162.380 | 79.27634 | 13.820719 |
|  | ONH_NL | 357 | 44.732 | 137.261 | 73.01126 | 11.909073 |
|  | ONH_IN | 357 | 60.908 | 176.878 | 111.93620 | 20.455214 |
|  | ONH_IT | 357 | 52.183 | 177.365 | 131.77751 | 19.169946 |
|  | ONH_TL | 357 | 27.422 | 109.577 | 66.21564 | 10.903166 |
|  | ONH_TU1 | 357 | 31.194 | 104.474 | 65.93152 | 11.096264 |
|  | ONH_TU2 | 357 | 40.852 | 145.762 | 91.66192 | 17.210556 |
|  | ONH_ST2 | 357 | 57.749 | 189.491 | 126.36843 | 22.136315 |
|  | ONH_ST1 | 357 | 69.548 | 189.810 | 130.62458 | 21.116520 |
|  | ONH_SN1 | 357 | 46.075 | 190.281 | 108.07087 | 20.139711 |
|  | ONH_SN2 | 357 | 51.923 | 157.848 | 103.29061 | 16.624245 |
|  | ONH_NU2 | 357 | 41.384 | 195.892 | 87.88801 | 16.668098 |
|  | ONH_NU1 | 357 | 37.010 | 145.328 | 70.17748 | 13.164672 |
|  | ONH_NL1 | 357 | 41.086 | 135.105 | 66.34294 | 11.576895 |
|  | ONH_NL2 | 357 | 40.079 | 139.379 | 79.65962 | 13.915362 |
|  | ONH_IN2 | 357 | 58.659 | 175.982 | 100.68952 | 19.052836 |
|  | ONH_IN1 | 357 | 62.728 | 196.946 | 122.59321 | 23.703810 |
|  | ONH_IT1 | 357 | 50.105 | 193.581 | 142.72901 | 21.030749 |
|  | ONH_IT2 | 357 | 54.224 | 181.306 | 121.19402 | 23.860960 |
|  | ONH_TL2 | 357 | 38.382 | 126.133 | 74.77401 | 13.949765 |
|  | ONH_TL1 | 357 | 16.651 | 115.621 | 57.57140 | 9.932362 |
|  | Macula_3mm_Thk_ILM_IPL_um_Center_1 | 357 | 25.8 | 96.4 | 47.634 | 9.8009 |
|  | Macula_3mm_Thk_ILM_IPL_um_T_1minus3 | 357 | 54.0 | 186.2 | 101.939 | 11.8283 |
|  | Macula_3mm_Thk_ILM_IPL_um_S_1minus3 | 357 | 57.9 | 176.9 | 112.063 | 13.6703 |
|  | Macula_3mm_Thk_ILM_IPL_um_N_1minus3 | 357 | 57.7 | 169.2 | 107.561 | 12.8421 |
|  | Macula_3mm_Thk_ILM_IPL_um_I_1minus3 | 357 | 63.4 | 167.1 | 112.271 | 12.6806 |
|  | Macula_3mm_Thk_ILM_IPL_um_S_Hemi_1minus3 | 357 | 55.7 | 177.8 | 108.114 | 12.7947 |
|  | Macula_3mm_Thk_ILM_IPL_um_I_Hemi_1minus3 | 357 | 60.8 | 172.0 | 108.792 | 12.2593 |
|  | Macula_3mm_Thk_ILM_IPL_um_All_1minus3 | 357 | 58.3 | 174.8 | 108.457 | 12.2699 |
|  | Macula_3mm_Thk_ILM_IPL_um_S_Hemi_0minus3 | 357 | 52.4 | 168.1 | 101.294 | 12.0679 |
|  | Macula_3mm_Thk_ILM_IPL_um_I_Hemi_0minus3 | 357 | 56.9 | 163.5 | 101.919 | 11.5704 |
|  | Macula_3mm_Thk_ILM_IPL_um_All_0minus3 | 357 | 54.6 | 165.7 | 101.610 | 11.6042 |
|  | Macula_3mm_Thk_ILM_IPL_um_S_Hemi_field | 357 | 54.5 | 169.7 | 102.630 | 12.0247 |
|  | Macula_3mm_Thk_ILM_IPL_um_I_Hemi_field | 357 | 59.0 | 163.4 | 103.642 | 11.5618 |
|  | Macula_3mm_Thk_ILM_IPL_um_All_field | 357 | 56.8 | 166.2 | 103.256 | 11.5368 |
|  | Macula_3mm_Thk_ILM_RPE_um_Center_1 | 357 | 178.5 | 457.9 | 259.224 | 26.9095 |
|  | Macula_3mm_Thk_ILM_RPE_um_T_1minus3 | 357 | 218.8 | 451.3 | 316.494 | 20.2799 |
|  | Macula_3mm_Thk_ILM_RPE_um_S_1minus3 | 357 | 217.1 | 440.4 | 327.287 | 21.3049 |
|  | Macula_3mm_Thk_ILM_RPE_um_N_1minus3 | 357 | 237.2 | 427.9 | 328.104 | 21.3205 |
|  | Macula_3mm_Thk_ILM_RPE_um_I_1minus3 | 357 | 236.3 | 430.0 | 325.292 | 20.6836 |
|  | Macula_3mm_Thk_ILM_RPE_um_S_Hemi_1minus3 | 357 | 218.5 | 439.9 | 324.572 | 20.7108 |
|  | Macula_3mm_Thk_ILM_RPE_um_I_Hemi_1minus3 | 357 | 236.3 | 434.9 | 323.998 | 20.3391 |
|  | Macula_3mm_Thk_ILM_RPE_um_All_1minus3 | 357 | 227.7 | 437.3 | 324.287 | 20.2395 |
|  | Macula_3mm_Thk_ILM_RPE_um_S_Hemi_0minus3 | 357 | 217.8 | 442.1 | 317.207 | 20.5800 |
|  | Macula_3mm_Thk_ILM_RPE_um_I_Hemi_0minus3 | 357 | 233.6 | 437.5 | 316.720 | 20.2659 |
|  | Macula_3mm_Thk_ILM_RPE_um_All_0minus3 | 357 | 226.0 | 439.7 | 316.964 | 20.1947 |
|  | Macula_3mm_Thk_ILM_RPE_um_S_Hemi_field | 357 | 216.6 | 437.4 | 315.201 | 20.0818 |
|  | Macula_3mm_Thk_ILM_RPE_um_I_Hemi_field | 357 | 226.5 | 418.5 | 313.908 | 19.5279 |
|  | Macula_3mm_Thk_ILM_RPE_um_All_field | 357 | 222.2 | 426.4 | 314.615 | 19.5048 |
|  | Macula_3mm_Thk_ILM_BRM_um_Center_1 | 357 | 192.2 | 464.9 | 266.442 | 26.9263 |
|  | Macula_3mm_Thk_ILM_BRM_um_T_1minus3 | 357 | 246.4 | 454.3 | 321.393 | 20.1114 |
|  | Macula_3mm_Thk_ILM_BRM_um_S_1minus3 | 357 | 224.4 | 444.6 | 332.562 | 21.1630 |
|  | Macula_3mm_Thk_ILM_BRM_um_N_1minus3 | 357 | 259.8 | 433.5 | 333.946 | 21.1089 |
|  | Macula_3mm_Thk_ILM_BRM_um_I_1minus3 | 357 | 252.9 | 434.4 | 330.453 | 19.9856 |
|  | Macula_3mm_Thk_ILM_BRM_um_S_Hemi_1minus3 | 357 | 228.8 | 443.9 | 329.887 | 20.5615 |
|  | Macula_3mm_Thk_ILM_BRM_um_I_Hemi_1minus3 | 357 | 262.7 | 439.5 | 329.265 | 19.8597 |
|  | Macula_3mm_Thk_ILM_BRM_um_All_1minus3 | 357 | 257.6 | 441.6 | 329.585 | 19.8293 |
|  | Macula_3mm_Thk_ILM_BRM_um_S_Hemi_0minus3 | 357 | 228.9 | 446.4 | 322.739 | 20.4250 |
|  | Macula_3mm_Thk_ILM_BRM_um_I_Hemi_0minus3 | 357 | 256.0 | 442.3 | 322.214 | 19.8186 |
|  | Macula_3mm_Thk_ILM_BRM_um_All_0minus3 | 357 | 251.5 | 444.3 | 322.478 | 19.8225 |
|  | Macula_3mm_Thk_ILM_BRM_um_S_Hemi_field | 357 | 226.8 | 441.6 | 320.569 | 19.8839 |
|  | Macula_3mm_Thk_ILM_BRM_um_I_Hemi_field | 357 | 256.7 | 422.6 | 319.218 | 18.9638 |
|  | Macula_3mm_Thk_ILM_BRM_um_All_field | 357 | 252.0 | 430.6 | 319.959 | 19.0059 |
|  | Macula_3mm_Thk_RPE_BRM_um_Center_1 | 357 | 1.5 | 42.2 | 7.215 | 2.9606 |
|  | Macula_3mm_Thk_RPE_BRM_um_T_1minus3 | 357 | .8 | 29.9 | 4.896 | 2.2897 |
|  | Macula_3mm_Thk_RPE_BRM_um_S_1minus3 | 357 | .7 | 17.4 | 5.272 | 2.1888 |
|  | Macula_3mm_Thk_RPE_BRM_um_N_1minus3 | 357 | .4 | 25.6 | 5.841 | 2.8539 |
|  | Macula_3mm_Thk_RPE_BRM_um_I_1minus3 | 357 | .6 | 88.5 | 5.156 | 5.3095 |
|  | Macula_3mm_Thk_RPE_BRM_um_S_Hemi_1minus3 | 357 | .7 | 16.1 | 5.317 | 2.1271 |
|  | Macula_3mm_Thk_RPE_BRM_um_I_Hemi_1minus3 | 357 | .8 | 64.7 | 5.269 | 4.0405 |
|  | Macula_3mm_Thk_RPE_BRM_um_All_1minus3 | 357 | .8 | 38.5 | 5.296 | 2.7765 |
|  | Macula_3mm_Thk_RPE_BRM_um_S_Hemi_0minus3 | 357 | .8 | 15.8 | 5.527 | 2.1156 |
|  | Macula_3mm_Thk_RPE_BRM_um_I_Hemi_0minus3 | 357 | 1.0 | 59.4 | 5.490 | 3.7728 |
|  | Macula_3mm_Thk_RPE_BRM_um_All_0minus3 | 357 | .9 | 36.0 | 5.512 | 2.6861 |
|  | Macula_3mm_Thk_RPE_BRM_um_S_Hemi_field | 357 | .8 | 17.8 | 5.368 | 2.0726 |
|  | Macula_3mm_Thk_RPE_BRM_um_I_Hemi_field | 357 | .9 | 68.5 | 5.309 | 4.1113 |
|  | Macula_3mm_Thk_RPE_BRM_um_All_field | 357 | 1.0 | 43.6 | 5.346 | 2.9003 |
|  | Macula_3mm_Vol_ILM_IPL_mm3_Center_1 | 357 | .020 | .076 | .03742 | .007730 |
|  | Macula_3mm_Vol_ILM_IPL_mm3_T_1minus3 | 357 | .085 | .293 | .15859 | .018931 |
|  | Macula_3mm_Vol_ILM_IPL_mm3_S_1minus3 | 357 | .090 | .264 | .17213 | .021542 |
|  | Macula_3mm_Vol_ILM_IPL_mm3_N_1minus3 | 357 | .090 | .264 | .16603 | .020136 |
|  | Macula_3mm_Vol_ILM_IPL_mm3_I_1minus3 | 357 | .099 | .262 | .17513 | .020057 |
|  | Macula_3mm_Vol_ILM_IPL_mm3_S_Hemi_1minus3 | 357 | .174 | .513 | .33413 | .039497 |
|  | Macula_3mm_Vol_ILM_IPL_mm3_I_Hemi_1minus3 | 357 | .191 | .539 | .33783 | .038234 |
|  | Macula_3mm_Vol_ILM_IPL_mm3_All_1minus3 | 357 | .365 | 1.053 | .67191 | .075754 |
|  | Macula_3mm_Vol_ILM_IPL_mm3_S_Hemi_0minus3 | 357 | .185 | .551 | .35282 | .041928 |
|  | Macula_3mm_Vol_ILM_IPL_mm3_I_Hemi_0minus3 | 357 | .201 | .577 | .35650 | .040627 |
|  | Macula_3mm_Vol_ILM_IPL_mm3_All_0minus3 | 357 | .386 | 1.128 | .70935 | .080656 |
|  | Macula_3mm_Vol_ILM_IPL_mm3_S_Hemi_field | 357 | .242 | .708 | .45396 | .058266 |
|  | Macula_3mm_Vol_ILM_IPL_mm3_I_Hemi_field | 357 | .271 | .851 | .47760 | .059126 |
|  | Macula_3mm_Vol_ILM_IPL_mm3_All_field | 357 | .513 | 1.500 | .93162 | .104092 |
|  | Macula_3mm_Vol_ILM_RPE_mm3_Center_1 | 357 | .140 | .360 | .20369 | .021144 |
|  | Macula_3mm_Vol_ILM_RPE_mm3_T_1minus3 | 357 | .345 | .710 | .49234 | .033947 |
|  | Macula_3mm_Vol_ILM_RPE_mm3_S_1minus3 | 357 | .293 | .645 | .50281 | .035653 |
|  | Macula_3mm_Vol_ILM_RPE_mm3_N_1minus3 | 357 | .363 | .669 | .50659 | .035531 |
|  | Macula_3mm_Vol_ILM_RPE_mm3_I_1minus3 | 357 | .371 | .675 | .50742 | .033629 |
|  | Macula_3mm_Vol_ILM_RPE_mm3_S_Hemi_1minus3 | 357 | .638 | 1.270 | 1.00309 | .064913 |
|  | Macula_3mm_Vol_ILM_RPE_mm3_I_Hemi_1minus3 | 357 | .742 | 1.365 | 1.00603 | .064251 |
|  | Macula_3mm_Vol_ILM_RPE_mm3_All_1minus3 | 357 | 1.379 | 2.634 | 2.00911 | .125326 |
|  | Macula_3mm_Vol_ILM_RPE_mm3_S_Hemi_0minus3 | 357 | .721 | 1.450 | 1.10494 | .072057 |
|  | Macula_3mm_Vol_ILM_RPE_mm3_I_Hemi_0minus3 | 357 | .825 | 1.544 | 1.10791 | .071825 |
|  | Macula_3mm_Vol_ILM_RPE_mm3_All_0minus3 | 357 | 1.546 | 2.994 | 2.21287 | .140448 |
|  | Macula_3mm_Vol_ILM_RPE_mm3_S_Hemi_field | 357 | .840 | 1.805 | 1.39368 | .110084 |
|  | Macula_3mm_Vol_ILM_RPE_mm3_I_Hemi_field | 357 | 1.094 | 2.181 | 1.44647 | .115299 |
|  | Macula_3mm_Vol_ILM_RPE_mm3_All_field | 357 | 2.007 | 3.851 | 2.84016 | .176122 |
|  | Macula_3mm_Vol_ILM_BRM_mm3_Center_1 | 357 | .151 | .365 | .20935 | .021157 |
|  | Macula_3mm_Vol_ILM_BRM_mm3_T_1minus3 | 357 | .388 | .715 | .49994 | .033827 |
|  | Macula_3mm_Vol_ILM_BRM_mm3_S_1minus3 | 357 | .303 | .655 | .51093 | .035528 |
|  | Macula_3mm_Vol_ILM_BRM_mm3_N_1minus3 | 357 | .378 | .677 | .51560 | .035352 |
|  | Macula_3mm_Vol_ILM_BRM_mm3_I_1minus3 | 357 | .377 | .682 | .51546 | .032742 |
|  | Macula_3mm_Vol_ILM_BRM_mm3_S_Hemi_1minus3 | 357 | .668 | 1.281 | 1.01950 | .064494 |
|  | Macula_3mm_Vol_ILM_BRM_mm3_I_Hemi_1minus3 | 357 | .805 | 1.379 | 1.02241 | .062958 |
|  | Macula_3mm_Vol_ILM_BRM_mm3_All_1minus3 | 357 | 1.584 | 2.660 | 2.04196 | .122790 |
|  | Macula_3mm_Vol_ILM_BRM_mm3_S_Hemi_0minus3 | 357 | .758 | 1.464 | 1.12420 | .071559 |
|  | Macula_3mm_Vol_ILM_BRM_mm3_I_Hemi_0minus3 | 357 | .886 | 1.562 | 1.12713 | .070462 |
|  | Macula_3mm_Vol_ILM_BRM_mm3_All_0minus3 | 357 | 1.744 | 3.025 | 2.25134 | .137849 |
|  | Macula_3mm_Vol_ILM_BRM_mm3_S_Hemi_field | 357 | .880 | 1.837 | 1.41743 | .109947 |
|  | Macula_3mm_Vol_ILM_BRM_mm3_I_Hemi_field | 357 | 1.099 | 2.202 | 1.47103 | .115059 |
|  | Macula_3mm_Vol_ILM_BRM_mm3_All_field | 357 | 2.275 | 3.888 | 2.88847 | .171594 |
|  | Macula_3mm_Vol_RPE_BRM_mm3_Center_1 | 357 | .001 | .033 | .00567 | .002334 |
|  | Macula_3mm_Vol_RPE_BRM_mm3_T_1minus3 | 357 | .001 | .047 | .00763 | .003594 |
|  | Macula_3mm_Vol_RPE_BRM_mm3_S_1minus3 | 357 | .001 | .027 | .00811 | .003390 |
|  | Macula_3mm_Vol_RPE_BRM_mm3_N_1minus3 | 357 | .001 | .040 | .00901 | .004424 |
|  | Macula_3mm_Vol_RPE_BRM_mm3_I_1minus3 | 357 | .001 | .139 | .00803 | .008343 |
|  | Macula_3mm_Vol_RPE_BRM_mm3_S_Hemi_1minus3 | 357 | .002 | .050 | .01641 | .006561 |
|  | Macula_3mm_Vol_RPE_BRM_mm3_I_Hemi_1minus3 | 357 | .002 | .203 | .01638 | .012663 |
|  | Macula_3mm_Vol_RPE_BRM_mm3_All_1minus3 | 357 | .005 | .233 | .03283 | .017062 |
|  | Macula_3mm_Vol_RPE_BRM_mm3_S_Hemi_0minus3 | 357 | .003 | .056 | .01923 | .007379 |
|  | Macula_3mm_Vol_RPE_BRM_mm3_I_Hemi_0minus3 | 357 | .003 | .210 | .01922 | .013302 |
|  | Macula_3mm_Vol_RPE_BRM_mm3_All_0minus3 | 357 | .006 | .247 | .03844 | .018627 |
|  | Macula_3mm_Vol_RPE_BRM_mm3_S_Hemi_field | 357 | .004 | .081 | .02371 | .009209 |
|  | Macula_3mm_Vol_RPE_BRM_mm3_I_Hemi_field | 357 | .004 | .353 | .02457 | .020655 |
|  | Macula_3mm_Vol_RPE_BRM_mm3_All_field | 357 | .009 | .393 | .04828 | .026151 |
|  | SVC_L1_DensityOfWhole_Image | 357 | 26.09 | 56.89 | 45.9209 | 4.08692 |
|  | SVC_L1_Whole_Image_S_Hemi | 357 | 24.22 | 56.93 | 45.8949 | 4.14826 |
|  | SVC_L1_Whole_Image_I_Hemi | 357 | 27.75 | 56.86 | 45.9391 | 4.22127 |
|  | SVC_L1_Whole_ETDRS | 357 | 25.73 | 56.26 | 45.5407 | 4.04893 |
|  | SVC_L1_Fovea | 357 | 1.59 | 38.29 | 20.4209 | 6.63652 |
|  | SVC_L1_ParaFovea | 357 | 27.27 | 59.94 | 48.7259 | 4.12725 |
|  | SVC_L1_Para_S_Hemi | 357 | 25.74 | 60.88 | 48.6827 | 4.25150 |
|  | SVC_L1_Para_I_Hemi | 357 | 28.77 | 59.03 | 48.7637 | 4.26060 |
|  | SVC_L1_Para_T | 357 | 25.74 | 58.86 | 47.6749 | 4.38582 |
|  | SVC_L1_Para_S | 357 | 26.17 | 61.83 | 49.7005 | 4.51418 |
|  | SVC_L1_Para_N | 357 | 23.84 | 60.33 | 47.9908 | 4.33799 |
|  | SVC_L1_Para_I | 357 | 28.26 | 58.90 | 49.5370 | 4.68367 |
|  | SVC_L1_G11 | 357 | 21.07 | 58.41 | 47.9367 | 4.99859 |
|  | SVC_L1_G12 | 357 | 28.94 | 61.20 | 49.8978 | 4.52302 |
|  | SVC_L1_G13 | 357 | 23.30 | 61.41 | 48.2373 | 4.92422 |
|  | SVC_L1_G21 | 357 | 30.91 | 56.75 | 47.4022 | 4.43751 |
|  | SVC_L1_G22 | 357 | 7.87 | 42.47 | 25.8675 | 6.00944 |
|  | SVC_L1_G23 | 357 | 22.27 | 59.76 | 47.6106 | 4.69147 |
|  | SVC_L1_G31 | 357 | 28.20 | 59.78 | 47.9753 | 5.08977 |
|  | SVC_L1_G32 | 357 | 26.64 | 60.64 | 49.8907 | 4.89485 |
|  | SVC_L1_G33 | 357 | 27.50 | 60.28 | 48.2708 | 5.27379 |
|  | DVC_L2_DensityOfWhole_Image | 357 | 31.11 | 58.31 | 48.1327 | 4.32465 |
|  | DVC_L2_Whole_Image_S_Hemi | 357 | 31.19 | 58.33 | 48.3377 | 4.33301 |
|  | DVC_L2_Whole_Image_I_Hemi | 357 | 31.06 | 58.33 | 47.9315 | 4.51435 |
|  | DVC_L2_Whole_ETDRS | 357 | 31.39 | 58.50 | 48.0211 | 4.09753 |
|  | DVC_L2_Fovea | 357 | 14.15 | 50.38 | 31.2408 | 6.82786 |
|  | DVC_L2_ParaFovea | 357 | 31.78 | 61.01 | 50.1471 | 4.41429 |
|  | DVC_L2_Para_S_Hemi | 357 | 30.59 | 60.53 | 50.3076 | 4.40136 |
|  | DVC_L2_Para_I_Hemi | 357 | 32.88 | 61.47 | 49.9755 | 4.65574 |
|  | DVC_L2_Para_T | 357 | 35.68 | 60.51 | 50.4267 | 4.33539 |
|  | DVC_L2_Para_S | 357 | 29.43 | 60.12 | 50.3741 | 4.82209 |
|  | DVC_L2_Para_N | 357 | 32.23 | 64.03 | 50.2189 | 4.49745 |
|  | DVC_L2_Para_I | 357 | 29.41 | 61.34 | 49.5701 | 5.27418 |
|  | DVC_L2_G11 | 357 | 32.91 | 62.22 | 49.3466 | 5.59273 |
|  | DVC_L2_G12 | 357 | 32.49 | 60.33 | 50.6051 | 4.84606 |
|  | DVC_L2_G13 | 357 | 27.98 | 61.62 | 49.3161 | 5.57598 |
|  | DVC_L2_G21 | 357 | 35.37 | 60.33 | 50.5482 | 4.20688 |
|  | DVC_L2_G22 | 357 | 20.27 | 50.80 | 35.6961 | 5.61015 |
|  | DVC_L2_G23 | 357 | 34.50 | 63.27 | 50.7283 | 4.47819 |
|  | DVC_L2_G31 | 357 | 28.06 | 60.92 | 48.6557 | 5.82662 |
|  | DVC_L2_G32 | 357 | 28.27 | 62.92 | 49.8176 | 5.62894 |
|  | DVC_L2_G33 | 357 | 26.19 | 62.26 | 48.4176 | 5.87083 |
|  | FAZ_Area | 357 | .016 | .666 | .30303 | .117516 |
|  | Perimeter | 357 | .483 | 3.581 | 2.20923 | .466242 |
|  | AcircularityIndex | 357 | 1.06 | 1.42 | 1.1552 | .05350 |
|  | FD_300_Area_Density | 357 | 23.73 | 59.67 | 48.6737 | 4.46573 |
|  | FD_300_Length_Density | 357 | 6.15 | 19.54 | 15.9871 | 2.24722 |
|  | FoveaInnRet_Thickness | 357 | 45.67 | 143.34 | 73.2079 | 13.93155 |
|  | InnRet_Thk_ParaFovea | 357 | 83 | 160 | 126.35 | 11.695 |
|  | InnRet_Thk_Para_S_Hemisphere | 357 | 78 | 171 | 126.40 | 12.438 |
|  | InnRet_Thk_Para_I_Hemisphere | 357 | 85 | 161 | 126.33 | 11.920 |
|  | InnRet_Thk_Para_Tempo | 357 | 52 | 164 | 119.50 | 11.699 |
|  | InnRet_Thk_Para_Superior | 357 | 75 | 170 | 128.68 | 13.256 |
|  | InnRet_Thk_Para_Nasal | 357 | 84 | 174 | 129.32 | 13.897 |
|  | InnRet_Thk_Para_Inferior | 357 | 82 | 164 | 127.96 | 12.630 |
|  | InnRet_Thk_PeriFovea | 357 | 71 | 151 | 109.97 | 9.713 |
|  | InnRet_Thk_Peri_S_Hemisphere | 357 | 70 | 155 | 110.51 | 9.845 |
|  | InnRet_Thk_Peri_I_Hemisphere | 357 | 63 | 167 | 109.48 | 10.871 |
|  | InnRet_Thk_Peri_Tempo | 357 | 42 | 192 | 104.27 | 11.097 |
|  | InnRet_Thk_Peri_Superior | 357 | 63 | 150 | 109.45 | 10.212 |
|  | InnRet_Thk_Peri_Nasal | 357 | 73 | 158 | 119.08 | 11.020 |
|  | InnRet_Thk_Peri_Inferior | 357 | 50 | 285 | 107.40 | 14.759 |
|  | FoveaInnRet_Volumn | 357 | .030 | .113 | .05732 | .011287 |
|  | InnRet_Vol_ParaFovea | 357 | .443 | 1.009 | .79368 | .076663 |
|  | InnRet_Vol_Para_S_Hemisphere | 357 | .227 | .538 | .39650 | .040557 |
|  | InnRet_Vol_Para_I_Hemisphere | 357 | .183 | .505 | .39693 | .039910 |
|  | InnRet_Vol_Para_Tempo | 357 | .081 | .257 | .18782 | .020793 |
|  | InnRet_Vol_Para_Superior | 357 | .118 | .269 | .20165 | .021897 |
|  | InnRet_Vol_Para_Nasal | 357 | .079 | .273 | .20303 | .023804 |
|  | InnRet_Vol_Para_Inferior | 357 | .110 | .257 | .20054 | .020948 |
|  | InnRet_Vol_PeriFovea | 357 | .784 | 1.899 | 1.37761 | .132514 |
|  | InnRet_Vol_Peri_S_Hemisphere | 357 | .414 | .971 | .69284 | .068045 |
|  | InnRet_Vol_Peri_I_Hemisphere | 357 | .324 | 1.048 | .68468 | .072993 |
|  | InnRet_Vol_Peri_Tempo | 357 | .133 | .602 | .32700 | .041553 |
|  | InnRet_Vol_Peri_Superior | 357 | .128 | .471 | .34312 | .037034 |
|  | InnRet_Vol_Peri_Nasal | 357 | .177 | .495 | .37348 | .039010 |
|  | InnRet_Vol_Peri_Inferior | 357 | .126 | .531 | .33382 | .040504 |
|  | FoveaFullRet_Thickness | 357 | 193.88 | 459.26 | 260.5494 | 26.56362 |
|  | FullRet_Thk_ParaFovea | 357 | 232 | 428 | 317.92 | 19.974 |
|  | FullRet_Thk_Para_S_Hemisphere | 357 | 219 | 433 | 317.75 | 20.603 |
|  | FullRet_Thk_Para_I_Hemisphere | 357 | 244 | 423 | 317.85 | 20.108 |
|  | FullRet_Thk_Para_Tempo | 357 | 201 | 450 | 310.58 | 21.214 |
|  | FullRet_Thk_Para_Superior | 357 | 215 | 432 | 319.58 | 20.849 |
|  | FullRet_Thk_Para_Nasal | 357 | 238 | 417 | 322.40 | 21.159 |
|  | FullRet_Thk_Para_Inferior | 357 | 242 | 411 | 318.24 | 20.273 |
|  | FullRet_Thk_PeriFovea | 357 | 187 | 366 | 283.34 | 17.931 |
|  | FullRet_Thk_Peri_S_Hemisphere | 357 | 201 | 385 | 286.00 | 18.705 |
|  | FullRet_Thk_Peri_I_Hemisphere | 357 | 172 | 347 | 280.56 | 18.020 |
|  | FullRet_Thk_Peri_Tempo | 357 | 95 | 384 | 274.54 | 20.530 |
|  | FullRet_Thk_Peri_Superior | 357 | 206 | 388 | 284.70 | 19.737 |
|  | FullRet_Thk_Peri_Nasal | 357 | 222 | 367 | 298.36 | 18.970 |
|  | FullRet_Thk_Peri_Inferior | 357 | 168 | 353 | 274.88 | 18.621 |
|  | FoveaFullRet_Volumn | 357 | .142 | .361 | .20497 | .021361 |
|  | FullRet_Vol_ParaFovea | 357 | 1.455 | 2.687 | 1.99595 | .127698 |
|  | FullRet_Vol_Para_S_Hemisphere | 357 | .688 | 1.359 | .99776 | .066515 |
|  | FullRet_Vol_Para_I_Hemisphere | 357 | .767 | 1.328 | .99819 | .063836 |
|  | FullRet_Vol_Para_Tempo | 357 | .316 | .707 | .48902 | .039379 |
|  | FullRet_Vol_Para_Superior | 357 | .337 | .678 | .50163 | .034105 |
|  | FullRet_Vol_Para_Nasal | 357 | .373 | .655 | .50573 | .033608 |
|  | FullRet_Vol_Para_Inferior | 357 | .381 | .646 | .49955 | .031755 |
|  | FullRet_Vol_PeriFovea | 357 | 2.344 | 4.596 | 3.55502 | .228768 |
|  | FullRet_Vol_Peri_S_Hemisphere | 357 | 1.263 | 2.416 | 1.79546 | .122322 |
|  | FullRet_Vol_Peri_I_Hemisphere | 357 | 1.080 | 2.180 | 1.75931 | .116631 |
|  | FullRet_Vol_Peri_Tempo | 357 | .299 | 1.205 | .86484 | .069055 |
|  | FullRet_Vol_Peri_Superior | 357 | .573 | 1.219 | .89341 | .070995 |
|  | FullRet_Vol_Peri_Nasal | 357 | .697 | 1.250 | .93648 | .065819 |
|  | FullRet_Vol_Peri_Inferior | 357 | .512 | 1.108 | .86016 | .065606 |
|  | FoveaRPE_Elevation_Height | 357 | -25.34 | 149.85 | 1.6627 | 19.10136 |
|  | RPE_Elev_ParaFovea_Tempo | 357 | -54.7 | 101.5 | 3.555 | 13.3947 |
|  | RPE_Elev_Para_Superior | 357 | -19.5 | 83.5 | 1.209 | 12.1028 |
|  | RPE_Elev_Para_Nasal | 357 | -20.8 | 127.2 | 5.791 | 16.2456 |
|  | RPE_Elev_Para_Inferior | 357 | -62.7 | 91.7 | 3.610 | 13.9564 |
|  | RPE_Elev_PeriFovea_Tempo | 357 | -32.4 | 507.9 | 3.646 | 27.9435 |
|  | RPE_Elev_Peri_Superior | 357 | -23.6 | 120.6 | .648 | 9.8166 |
|  | RPE_Elev_Peri_Nasal | 357 | -25.3 | 82.9 | 4.945 | 12.3261 |
|  | RPE_Elev_Peri_Inferior | 357 | -17.1 | 89.6 | .933 | 8.5614 |
| Offset | AgePerToday | 357 | 53 | 99 | 80.10 | 6.056 |

## Tests of Model Effects

| Source | Type III | | |
| --- | --- | --- | --- |
|  | Wald Chi-Square | df | Sig. |
| (Intercept) | 35.050 | 1 | **<.001** |
| Gender_M2F1 | 4.906 | 1 | **.027** |
| GCC_Inner_Retina_Average | 23.209 | 1 | **<.001** |
| GCC_Superior_Avg | 8.245 | 1 | **.004** |
| GCC_Inferior_Avg | 21.658 | 1 | **<.001** |
| GCC_S_I_Avg | 1.778 | 1 | .182 |
| GCC_FLV | 42.258 | 1 | **<.001** |
| GCC_GLV | 11.044 | 1 | **<.001** |
| GCC_RMS | 20.930 | 1 | **<.001** |
| GCC_Full_Retina_Average | 1.362 | 1 | .243 |
| GCC_Superior_Avg.1 | 37.805 | 1 | **<.001** |
| GCC_Inferior_Avg.1 | 15.830 | 1 | **<.001** |
| GCC_S_I_Avg.1 | 5.376 | 1 | **.020** |
| GCC_Outer_Retina_Average | 1.253 | 1 | .263 |
| GCC_Superior_Avg.2 | 1.519 | 1 | .218 |
| GCC_Inferior_Avg.2 | 40.254 | 1 | **<.001** |
| GCC_S_I_Avg.2 | 15.342 | 1 | **<.001** |
| ONH_DiscArea | 66.609 | 1 | **<.001** |
| ONH_Area_C_D_ratio | 4.225 | 1 | **.040** |
| ONH_H_C_D_ratio | 24.245 | 1 | **<.001** |
| ONH_V_C_D_ratio | .419 | 1 | .517 |
| ONH_CupArea | 10.554 | 1 | **.001** |
| ONH_RimArea | 39.131 | 1 | **<.001** |
| ONH_RimVolume | .080 | 1 | .778 |
| ONH_Disc_Volume | .018 | 1 | .894 |
| ONH_CupVolume | 68.650 | 1 | **<.001** |
| ONH_Avg_RNFL | 6.901 | 1 | **.009** |
| ONH_Sup_RNFL | 76.610 | 1 | **.000** |
| ONH_Inf_RNFL | 17.819 | 1 | **<.001** |
| ONH_Tempo | 2.060 | 1 | .151 |
| ONH_Superior | .001 | 1 | .976 |
| ONH_Nasal | 52.422 | 1 | **<.001** |
| ONH_Inferior | 27.560 | 1 | **<.001** |
| ONH_RNFL_TU | 16.426 | 1 | **<.001** |
| ONH_ST | 30.469 | 1 | **<.001** |
| ONH_SN | 35.878 | 1 | **<.001** |
| ONH_NU | 29.194 | 1 | **<.001** |
| ONH_NL | 3.142 | 1 | .076 |
| ONH_IN | 6.043 | 1 | **.014** |
| ONH_IT | 18.720 | 1 | **<.001** |
| ONH_TL | 3.779 | 1 | .052 |
| ONH_TU1 | 11.442 | 1 | **<.001** |
| ONH_TU2 | 4.165 | 1 | **.041** |
| ONH_ST2 | 1.131 | 1 | .288 |
| ONH_ST1 | 15.266 | 1 | **<.001** |
| ONH_SN1 | 6.220 | 1 | **.013** |
| ONH_SN2 | 11.823 | 1 | **<.001** |
| ONH_NU2 | 53.676 | 1 | **<.001** |
| ONH_NU1 | 35.657 | 1 | **<.001** |
| ONH_NL1 | .092 | 1 | .762 |
| ONH_NL2 | .040 | 1 | .842 |
| ONH_IN2 | .308 | 1 | .579 |
| ONH_IN1 | 2.281 | 1 | .131 |
| ONH_IT1 | 12.115 | 1 | **<.001** |
| ONH_IT2 | .361 | 1 | .548 |
| ONH_TL2 | 5.032 | 1 | **.025** |
| ONH_TL1 | 1.591 | 1 | .207 |
| Macula_3mm_Thk_ILM_IPL_um_Center_1 | 7.382 | 1 | **.007** |
| Macula_3mm_Thk_ILM_IPL_um_T_1minus3 | 2.190 | 1 | .139 |
| Macula_3mm_Thk_ILM_IPL_um_S_1minus3 | 7.134 | 1 | **.008** |
| Macula_3mm_Thk_ILM_IPL_um_N_1minus3 | .175 | 1 | .676 |
| Macula_3mm_Thk_ILM_IPL_um_I_1minus3 | .037 | 1 | .847 |
| Macula_3mm_Thk_ILM_IPL_um_S_Hemi_1minus3 | 5.935 | 1 | **.015** |
| Macula_3mm_Thk_ILM_IPL_um_I_Hemi_1minus3 | .115 | 1 | .735 |
| Macula_3mm_Thk_ILM_IPL_um_All_1minus3 | 24.230 | 1 | **<.001** |
| Macula_3mm_Thk_ILM_IPL_um_S_Hemi_0minus3 | 7.310 | 1 | **.007** |
| Macula_3mm_Thk_ILM_IPL_um_I_Hemi_0minus3 | 8.523 | 1 | **.004** |
| Macula_3mm_Thk_ILM_IPL_um_All_0minus3 | 95.579 | 1 | **.000** |
| Macula_3mm_Thk_ILM_IPL_um_S_Hemi_field | 41.535 | 1 | **<.001** |
| Macula_3mm_Thk_ILM_IPL_um_I_Hemi_field | 9.080 | 1 | **.003** |
| Macula_3mm_Thk_ILM_IPL_um_All_field | 5.005 | 1 | **.025** |
| Macula_3mm_Thk_ILM_RPE_um_Center_1 | 11.231 | 1 | **<.001** |
| Macula_3mm_Thk_ILM_RPE_um_T_1minus3 | 7.766 | 1 | **.005** |
| Macula_3mm_Thk_ILM_RPE_um_S_1minus3 | 4.548 | 1 | **.033** |
| Macula_3mm_Thk_ILM_RPE_um_N_1minus3 | 7.233 | 1 | **.007** |
| Macula_3mm_Thk_ILM_RPE_um_I_1minus3 | 5.678 | 1 | **.017** |
| Macula_3mm_Thk_ILM_RPE_um_S_Hemi_1minus3 | 28.553 | 1 | **<.001** |
| Macula_3mm_Thk_ILM_RPE_um_I_Hemi_1minus3 | 10.819 | 1 | **.001** |
| Macula_3mm_Thk_ILM_RPE_um_All_1minus3 | 32.301 | 1 | **<.001** |
| Macula_3mm_Thk_ILM_RPE_um_S_Hemi_0minus3 | 70.371 | 1 | **.000** |
| Macula_3mm_Thk_ILM_RPE_um_I_Hemi_0minus3 | 3.224 | 1 | .073 |
| Macula_3mm_Thk_ILM_RPE_um_All_0minus3 | 27.322 | 1 | **<.001** |
| Macula_3mm_Thk_ILM_RPE_um_S_Hemi_field | 17.735 | 1 | **<.001** |
| Macula_3mm_Thk_ILM_RPE_um_I_Hemi_field | 32.228 | 1 | **<.001** |
| Macula_3mm_Thk_ILM_RPE_um_All_field | .388 | 1 | .533 |
| Macula_3mm_Thk_ILM_BRM_um_Center_1 | 16.268 | 1 | **<.001** |
| Macula_3mm_Thk_ILM_BRM_um_T_1minus3 | 7.487 | 1 | **.006** |
| Macula_3mm_Thk_ILM_BRM_um_S_1minus3 | 3.124 | 1 | .077 |
| Macula_3mm_Thk_ILM_BRM_um_N_1minus3 | 6.264 | 1 | **.012** |
| Macula_3mm_Thk_ILM_BRM_um_I_1minus3 | 5.474 | 1 | **.019** |
| Macula_3mm_Thk_ILM_BRM_um_S_Hemi_1minus3 | .905 | 1 | .342 |
| Macula_3mm_Thk_ILM_BRM_um_I_Hemi_1minus3 | .048 | 1 | .826 |
| Macula_3mm_Thk_ILM_BRM_um_All_1minus3 | 4.402 | 1 | **.036** |
| Macula_3mm_Thk_ILM_BRM_um_S_Hemi_0minus3 | 6.736 | 1 | **.009** |
| Macula_3mm_Thk_ILM_BRM_um_I_Hemi_0minus3 | 6.555 | 1 | **.010** |
| Macula_3mm_Thk_ILM_BRM_um_All_0minus3 | 13.832 | 1 | **<.001** |
| Macula_3mm_Thk_ILM_BRM_um_S_Hemi_field | 14.014 | 1 | **<.001** |
| Macula_3mm_Thk_ILM_BRM_um_I_Hemi_field | 30.392 | 1 | **<.001** |
| Macula_3mm_Thk_ILM_BRM_um_All_field | 58.653 | 1 | **<.001** |
| Macula_3mm_Thk_RPE_BRM_um_Center_1 | 3.361 | 1 | .067 |
| Macula_3mm_Thk_RPE_BRM_um_T_1minus3 | 3.788 | 1 | .052 |
| Macula_3mm_Thk_RPE_BRM_um_S_1minus3 | 7.058 | 1 | **.008** |
| Macula_3mm_Thk_RPE_BRM_um_N_1minus3 | 22.758 | 1 | **<.001** |
| Macula_3mm_Thk_RPE_BRM_um_I_1minus3 | 1.569 | 1 | .210 |
| Macula_3mm_Thk_RPE_BRM_um_S_Hemi_1minus3 | 4.914 | 1 | **.027** |
| Macula_3mm_Thk_RPE_BRM_um_I_Hemi_1minus3 | .031 | 1 | .859 |
| Macula_3mm_Thk_RPE_BRM_um_All_1minus3 | 1.847 | 1 | .174 |
| Macula_3mm_Thk_RPE_BRM_um_S_Hemi_0minus3 | 17.468 | 1 | **<.001** |
| Macula_3mm_Thk_RPE_BRM_um_I_Hemi_0minus3 | 2.663 | 1 | .103 |
| Macula_3mm_Thk_RPE_BRM_um_All_0minus3 | 1.097 | 1 | .295 |
| Macula_3mm_Thk_RPE_BRM_um_S_Hemi_field | 44.212 | 1 | **<.001** |
| Macula_3mm_Thk_RPE_BRM_um_I_Hemi_field | 37.979 | 1 | **<.001** |
| Macula_3mm_Thk_RPE_BRM_um_All_field | .894 | 1 | .344 |
| Macula_3mm_Vol_ILM_IPL_mm3_Center_1 | .221 | 1 | .638 |
| Macula_3mm_Vol_ILM_IPL_mm3_T_1minus3 | 52.124 | 1 | **<.001** |
| Macula_3mm_Vol_ILM_IPL_mm3_S_1minus3 | .073 | 1 | .786 |
| Macula_3mm_Vol_ILM_IPL_mm3_N_1minus3 | 19.917 | 1 | **<.001** |
| Macula_3mm_Vol_ILM_IPL_mm3_I_1minus3 | 9.189 | 1 | **.002** |
| Macula_3mm_Vol_ILM_IPL_mm3_S_Hemi_1minus3 | 30.136 | 1 | **<.001** |
| Macula_3mm_Vol_ILM_IPL_mm3_I_Hemi_1minus3 | 39.374 | 1 | **<.001** |
| Macula_3mm_Vol_ILM_IPL_mm3_All_1minus3 | .425 | 1 | .515 |
| Macula_3mm_Vol_ILM_IPL_mm3_S_Hemi_0minus3 | .103 | 1 | .748 |
| Macula_3mm_Vol_ILM_IPL_mm3_I_Hemi_0minus3 | 27.944 | 1 | **<.001** |
| Macula_3mm_Vol_ILM_IPL_mm3_All_0minus3 | 11.931 | 1 | **<.001** |
| Macula_3mm_Vol_ILM_IPL_mm3_S_Hemi_field | 3.020 | 1 | .082 |
| Macula_3mm_Vol_ILM_IPL_mm3_I_Hemi_field | 7.833 | 1 | **.005** |
| Macula_3mm_Vol_ILM_IPL_mm3_All_field | 5.993 | 1 | **.014** |
| Macula_3mm_Vol_ILM_RPE_mm3_Center_1 | 34.118 | 1 | **<.001** |
| Macula_3mm_Vol_ILM_RPE_mm3_T_1minus3 | 11.802 | 1 | **<.001** |
| Macula_3mm_Vol_ILM_RPE_mm3_S_1minus3 | .000 | 1 | .992 |
| Macula_3mm_Vol_ILM_RPE_mm3_N_1minus3 | 2.768 | 1 | .096 |
| Macula_3mm_Vol_ILM_RPE_mm3_I_1minus3 | 1.710 | 1 | .191 |
| Macula_3mm_Vol_ILM_RPE_mm3_S_Hemi_1minus3 | .127 | 1 | .721 |
| Macula_3mm_Vol_ILM_RPE_mm3_I_Hemi_1minus3 | 40.837 | 1 | **<.001** |
| Macula_3mm_Vol_ILM_RPE_mm3_All_1minus3 | 16.207 | 1 | **<.001** |
| Macula_3mm_Vol_ILM_RPE_mm3_S_Hemi_0minus3 | 23.212 | 1 | **<.001** |
| Macula_3mm_Vol_ILM_RPE_mm3_I_Hemi_0minus3 | 2.685 | 1 | **.101** |
| Macula_3mm_Vol_ILM_RPE_mm3_All_0minus3 | 28.154 | 1 | **<.001** |
| Macula_3mm_Vol_ILM_RPE_mm3_S_Hemi_field | 8.223 | 1 | **.004** |
| Macula_3mm_Vol_ILM_RPE_mm3_I_Hemi_field | 52.647 | 1 | **<.001** |
| Macula_3mm_Vol_ILM_RPE_mm3_All_field | 7.413 | 1 | **.006** |
| Macula_3mm_Vol_ILM_BRM_mm3_Center_1 | 4.797 | 1 | **.029** |
| Macula_3mm_Vol_ILM_BRM_mm3_T_1minus3 | 36.035 | 1 | **<.001** |
| Macula_3mm_Vol_ILM_BRM_mm3_S_1minus3 | 1.501 | 1 | .221 |
| Macula_3mm_Vol_ILM_BRM_mm3_N_1minus3 | .146 | 1 | .703 |
| Macula_3mm_Vol_ILM_BRM_mm3_I_1minus3 | .584 | 1 | .445 |
| Macula_3mm_Vol_ILM_BRM_mm3_S_Hemi_1minus3 | 9.902 | 1 | **.002** |
| Macula_3mm_Vol_ILM_BRM_mm3_I_Hemi_1minus3 | 5.591 | 1 | **.018** |
| Macula_3mm_Vol_ILM_BRM_mm3_All_1minus3 | 76.681 | 1 | **.000** |
| Macula_3mm_Vol_ILM_BRM_mm3_S_Hemi_0minus3 | 7.878 | 1 | **.005** |
| Macula_3mm_Vol_ILM_BRM_mm3_I_Hemi_0minus3 | .995 | 1 | .319 |
| Macula_3mm_Vol_ILM_BRM_mm3_All_0minus3 | 3.221 | 1 | .073 |
| Macula_3mm_Vol_ILM_BRM_mm3_S_Hemi_field | 5.415 | 1 | **.020** |
| Macula_3mm_Vol_ILM_BRM_mm3_I_Hemi_field | 2.670 | 1 | .102 |
| Macula_3mm_Vol_ILM_BRM_mm3_All_field | 25.643 | 1 | **<.001** |
| Macula_3mm_Vol_RPE_BRM_mm3_Center_1 | 27.530 | 1 | **<.001** |
| Macula_3mm_Vol_RPE_BRM_mm3_T_1minus3 | 6.814 | 1 | **.009** |
| Macula_3mm_Vol_RPE_BRM_mm3_S_1minus3 | .055 | 1 | .814 |
| Macula_3mm_Vol_RPE_BRM_mm3_N_1minus3 | 11.878 | 1 | **<.001** |
| Macula_3mm_Vol_RPE_BRM_mm3_I_1minus3 | .470 | 1 | .493 |
| Macula_3mm_Vol_RPE_BRM_mm3_S_Hemi_1minus3 | 49.559 | 1 | **<.001** |
| Macula_3mm_Vol_RPE_BRM_mm3_I_Hemi_1minus3 | .177 | 1 | .674 |
| Macula_3mm_Vol_RPE_BRM_mm3_All_1minus3 | 5.420 | 1 | **.020** |
| Macula_3mm_Vol_RPE_BRM_mm3_S_Hemi_0minus3 | 30.842 | 1 | **<.001** |
| Macula_3mm_Vol_RPE_BRM_mm3_I_Hemi_0minus3 | .110 | 1 | .740 |
| Macula_3mm_Vol_RPE_BRM_mm3_All_0minus3 | 12.754 | 1 | **<.001** |
| Macula_3mm_Vol_RPE_BRM_mm3_S_Hemi_field | 28.650 | 1 | **<.001** |
| Macula_3mm_Vol_RPE_BRM_mm3_I_Hemi_field | 20.114 | 1 | **<.001** |
| Macula_3mm_Vol_RPE_BRM_mm3_All_field | 2.205 | 1 | .138 |
| SVC_L1_DensityOfWhole_Image | 3.923 | 1 | **.048** |
| SVC_L1_Whole_Image_S_Hemi | 13.959 | 1 | **<.001** |
| SVC_L1_Whole_Image_I_Hemi | 13.498 | 1 | **<.001** |
| SVC_L1_Whole_ETDRS | 11.352 | 1 | **<.001** |
| SVC_L1_Fovea | 11.879 | 1 | **<.001** |
| SVC_L1_ParaFovea | .506 | 1 | .477 |
| SVC_L1_Para_S_Hemi | 5.999 | 1 | **.014** |
| SVC_L1_Para_I_Hemi | 6.080 | 1 | **.014** |
| SVC_L1_Para_T | 22.035 | 1 | **<.001** |
| SVC_L1_Para_S | 23.349 | 1 | **<.001** |
| SVC_L1_Para_N | 21.347 | 1 | **<.001** |
| SVC_L1_Para_I | 20.170 | 1 | **<.001** |
| SVC_L1_G11 | 8.137 | 1 | **.004** |
| SVC_L1_G12 | 9.196 | 1 | **.002** |
| SVC_L1_G13 | 8.387 | 1 | **.004** |
| SVC_L1_G21 | 8.344 | 1 | **.004** |
| SVC_L1_G22 | 9.176 | 1 | **.002** |
| SVC_L1_G23 | 8.554 | 1 | **.003** |
| SVC_L1_G31 | 8.636 | 1 | **.003** |
| SVC_L1_G32 | 9.426 | 1 | **.002** |
| SVC_L1_G33 | 8.371 | 1 | **.004** |
| DVC_L2_DensityOfWhole_Image | 6.821 | 1 | **.009** |
| DVC_L2_Whole_Image_S_Hemi | 1.660 | 1 | .198 |
| DVC_L2_Whole_Image_I_Hemi | 1.163 | 1 | .281 |
| DVC_L2_Whole_ETDRS | 19.490 | 1 | **<.001** |
| DVC_L2_Fovea | 18.683 | 1 | **<.001** |
| DVC_L2_ParaFovea | 3.162 | 1 | .075 |
| DVC_L2_Para_S_Hemi | 7.169 | 1 | **.007** |
| DVC_L2_Para_I_Hemi | 7.551 | 1 | **.006** |
| DVC_L2_Para_T | 1.494 | 1 | .222 |
| DVC_L2_Para_S | 2.128 | 1 | .145 |
| DVC_L2_Para_N | 1.644 | 1 | .200 |
| DVC_L2_Para_I | 1.558 | 1 | .212 |
| DVC_L2_G11 | 7.508 | 1 | **.006** |
| DVC_L2_G12 | 7.017 | 1 | **.008** |
| DVC_L2_G13 | 7.630 | 1 | **.006** |
| DVC_L2_G21 | 7.301 | 1 | **.007** |
| DVC_L2_G22 | 7.317 | 1 | **.007** |
| DVC_L2_G23 | 8.211 | 1 | **.004** |
| DVC_L2_G31 | 8.062 | 1 | **.005** |
| DVC_L2_G32 | 7.424 | 1 | **.006** |
| DVC_L2_G33 | 7.525 | 1 | **.006** |
| FAZ_Area | 10.347 | 1 | **.001** |
| Perimeter | 8.400 | 1 | **.004** |
| AcircularityIndex | 17.816 | 1 | **<.001** |
| FD_300_Area_Density | 7.349 | 1 | **.007** |
| FD_300_Length_Density | 15.229 | 1 | **<.001** |
| FoveaInnRet_Thickness | 1.878 | 1 | .171 |
| InnRet_Thk_ParaFovea | 2.001 | 1 | .157 |
| InnRet_Thk_Para_S_Hemisphere | .105 | 1 | .746 |
| InnRet_Thk_Para_I_Hemisphere | 1.045 | 1 | .307 |
| InnRet_Thk_Para_Tempo | 17.047 | 1 | **<.001** |
| InnRet_Thk_Para_Superior | 1.860 | 1 | .173 |
| InnRet_Thk_Para_Nasal | 25.731 | 1 | **<.001** |
| InnRet_Thk_Para_Inferior | 2.749 | 1 | .097 |
| InnRet_Thk_PeriFovea | 2.618 | 1 | .106 |
| InnRet_Thk_Peri_S_Hemisphere | 3.608 | 1 | .058 |
| InnRet_Thk_Peri_I_Hemisphere | 29.243 | 1 | **<.001** |
| InnRet_Thk_Peri_Tempo | 12.198 | 1 | **<.001** |
| InnRet_Thk_Peri_Superior | 1.614 | 1 | .204 |
| InnRet_Thk_Peri_Nasal | .104 | 1 | .747 |
| InnRet_Thk_Peri_Inferior | 52.348 | 1 | **<.001** |
| FoveaInnRet_Volumn | 2.575 | 1 | .109 |
| InnRet_Vol_ParaFovea | 2.457 | 1 | .117 |
| InnRet_Vol_Para_S_Hemisphere | .001 | 1 | .981 |
| InnRet_Vol_Para_I_Hemisphere | .013 | 1 | .911 |
| InnRet_Vol_Para_Tempo | 19.948 | 1 | **<.001** |
| InnRet_Vol_Para_Superior | .016 | 1 | .899 |
| InnRet_Vol_Para_Nasal | 21.259 | 1 | **<.001** |
| InnRet_Vol_Para_Inferior | .189 | 1 | .664 |
| InnRet_Vol_PeriFovea | .043 | 1 | .836 |
| InnRet_Vol_Peri_S_Hemisphere | .416 | 1 | .519 |
| InnRet_Vol_Peri_I_Hemisphere | 3.844 | 1 | .050 |
| InnRet_Vol_Peri_Tempo | .456 | 1 | .499 |
| InnRet_Vol_Peri_Superior | .161 | 1 | .689 |
| InnRet_Vol_Peri_Nasal | .066 | 1 | .798 |
| InnRet_Vol_Peri_Inferior | 10.126 | 1 | **.001** |
| FoveaFullRet_Thickness | 10.356 | 1 | **.001** |
| FullRet_Thk_ParaFovea | 21.478 | 1 | **<.001** |
| FullRet_Thk_Para_S_Hemisphere | 5.645 | 1 | **.018** |
| FullRet_Thk_Para_I_Hemisphere | .119 | 1 | .730 |
| FullRet_Thk_Para_Tempo | 44.621 | 1 | **<.001** |
| FullRet_Thk_Para_Superior | 60.639 | 1 | **<.001** |
| FullRet_Thk_Para_Nasal | 2.500 | 1 | .114 |
| FullRet_Thk_Para_Inferior | .249 | 1 | .618 |
| FullRet_Thk_PeriFovea | 15.459 | 1 | **<.001** |
| FullRet_Thk_Peri_S_Hemisphere | 1.801 | 1 | .180 |
| FullRet_Thk_Peri_I_Hemisphere | .004 | 1 | .951 |
| FullRet_Thk_Peri_Tempo | 24.167 | 1 | **<.001** |
| FullRet_Thk_Peri_Superior | .004 | 1 | .952 |
| FullRet_Thk_Peri_Nasal | 14.639 | 1 | **<.001** |
| FullRet_Thk_Peri_Inferior | 12.021 | 1 | **<.001** |
| FoveaFullRet_Volumn | .821 | 1 | .365 |
| FullRet_Vol_ParaFovea | 7.604 | 1 | **.006** |
| FullRet_Vol_Para_S_Hemisphere | 65.725 | 1 | **<.001** |
| FullRet_Vol_Para_I_Hemisphere | 25.432 | 1 | **<.001** |
| FullRet_Vol_Para_Tempo | 21.065 | 1 | **<.001** |
| FullRet_Vol_Para_Superior | 42.671 | 1 | **<.001** |
| FullRet_Vol_Para_Nasal | 9.287 | 1 | **.002** |
| FullRet_Vol_Para_Inferior | 5.138 | 1 | **.023** |
| FullRet_Vol_PeriFovea | 8.161 | 1 | **.004** |
| FullRet_Vol_Peri_S_Hemisphere | .669 | 1 | .413 |
| FullRet_Vol_Peri_I_Hemisphere | .339 | 1 | .560 |
| FullRet_Vol_Peri_Tempo | 5.307 | 1 | **.021** |
| FullRet_Vol_Peri_Superior | .130 | 1 | .718 |
| FullRet_Vol_Peri_Nasal | .015 | 1 | .902 |
| FullRet_Vol_Peri_Inferior | .042 | 1 | .838 |
| FoveaRPE_Elevation_Height | 66.913 | 1 | **<.001** |
| RPE_Elev_ParaFovea_Tempo | 180.558 | 1 | **.000** |
| RPE_Elev_Para_Superior | 11.382 | 1 | **<.001** |
| RPE_Elev_Para_Nasal | .915 | 1 | .339 |
| RPE_Elev_Para_Inferior | 50.459 | 1 | **<.001** |
| RPE_Elev_PeriFovea_Tempo | 19.214 | 1 | **<.001** |
| RPE_Elev_Peri_Superior | 24.872 | 1 | **<.001** |
| RPE_Elev_Peri_Nasal | .068 | 1 | .794 |
| RPE_Elev_Peri_Inferior | 17.931 | 1 | **<.001** |

Dependent Variable: MMSE

Model: (Intercept), Gender_M2F1, GCC_Inner_Retina_Average, GCC_Superior_Avg, GCC_Inferior_Avg, GCC_S_I_Avg, GCC_FLV, GCC_GLV, GCC_RMS, GCC_Full_Retina_Average, GCC_Superior_Avg.1, GCC_Inferior_Avg.1, GCC_S_I_Avg.1, GCC_Outer_Retina_Average, GCC_Superior_Avg.2, GCC_Inferior_Avg.2, GCC_S_I_Avg.2, ONH_DiscArea, ONH_Area_C_D_ratio, ONH_H_C_D_ratio, ONH_V_C_D_ratio, ONH_CupArea, ONH_RimArea, ONH_RimVolume, ONH_Disc_Volume, ONH_CupVolume, ONH_Avg_RNFL, ONH_Sup_RNFL, ONH_Inf_RNFL, ONH_Tempo, ONH_Superior, ONH_Nasal, ONH_Inferior, ONH_RNFL_TU, ONH_ST, ONH_SN, ONH_NU, ONH_NL, ONH_IN, ONH_IT, ONH_TL, ONH_TU1, ONH_TU2, ONH_ST2, ONH_ST1, ONH_SN1, ONH_SN2, ONH_NU2, ONH_NU1, ONH_NL1, ONH_NL2, ONH_IN2, ONH_IN1, ONH_IT1, ONH_IT2, ONH_TL2, ONH_TL1, Macula_3mm_Thk_ILM_IPL_um_Center_1, Macula_3mm_Thk_ILM_IPL_um_T_1minus3, Macula_3mm_Thk_ILM_IPL_um_S_1minus3, Macula_3mm_Thk_ILM_IPL_um_N_1minus3, Macula_3mm_Thk_ILM_IPL_um_I_1minus3, Macula_3mm_Thk_ILM_IPL_um_S_Hemi_1minus3, Macula_3mm_Thk_ILM_IPL_um_I_Hemi_1minus3, Macula_3mm_Thk_ILM_IPL_um_All_1minus3, Macula_3mm_Thk_ILM_IPL_um_S_Hemi_0minus3, Macula_3mm_Thk_ILM_IPL_um_I_Hemi_0minus3, Macula_3mm_Thk_ILM_IPL_um_All_0minus3, Macula_3mm_Thk_ILM_IPL_um_S_Hemi_field, Macula_3mm_Thk_ILM_IPL_um_I_Hemi_field, Macula_3mm_Thk_ILM_IPL_um_All_field, Macula_3mm_Thk_ILM_RPE_um_Center_1, Macula_3mm_Thk_ILM_RPE_um_T_1minus3, Macula_3mm_Thk_ILM_RPE_um_S_1minus3, Macula_3mm_Thk_ILM_RPE_um_N_1minus3, Macula_3mm_Thk_ILM_RPE_um_I_1minus3, Macula_3mm_Thk_ILM_RPE_um_S_Hemi_1minus3, Macula_3mm_Thk_ILM_RPE_um_I_Hemi_1minus3, Macula_3mm_Thk_ILM_RPE_um_All_1minus3, Macula_3mm_Thk_ILM_RPE_um_S_Hemi_0minus3, Macula_3mm_Thk_ILM_RPE_um_I_Hemi_0minus3, Macula_3mm_Thk_ILM_RPE_um_All_0minus3, Macula_3mm_Thk_ILM_RPE_um_S_Hemi_field, Macula_3mm_Thk_ILM_RPE_um_I_Hemi_field, Macula_3mm_Thk_ILM_RPE_um_All_field, Macula_3mm_Thk_ILM_BRM_um_Center_1, Macula_3mm_Thk_ILM_BRM_um_T_1minus3, Macula_3mm_Thk_ILM_BRM_um_S_1minus3, Macula_3mm_Thk_ILM_BRM_um_N_1minus3, Macula_3mm_Thk_ILM_BRM_um_I_1minus3, Macula_3mm_Thk_ILM_BRM_um_S_Hemi_1minus3, Macula_3mm_Thk_ILM_BRM_um_I_Hemi_1minus3, Macula_3mm_Thk_ILM_BRM_um_All_1minus3, Macula_3mm_Thk_ILM_BRM_um_S_Hemi_0minus3, Macula_3mm_Thk_ILM_BRM_um_I_Hemi_0minus3, Macula_3mm_Thk_ILM_BRM_um_All_0minus3, Macula_3mm_Thk_ILM_BRM_um_S_Hemi_field, Macula_3mm_Thk_ILM_BRM_um_I_Hemi_field, Macula_3mm_Thk_ILM_BRM_um_All_field, Macula_3mm_Thk_RPE_BRM_um_Center_1, Macula_3mm_Thk_RPE_BRM_um_T_1minus3, Macula_3mm_Thk_RPE_BRM_um_S_1minus3, Macula_3mm_Thk_RPE_BRM_um_N_1minus3, Macula_3mm_Thk_RPE_BRM_um_I_1minus3, Macula_3mm_Thk_RPE_BRM_um_S_Hemi_1minus3, Macula_3mm_Thk_RPE_BRM_um_I_Hemi_1minus3, Macula_3mm_Thk_RPE_BRM_um_All_1minus3, Macula_3mm_Thk_RPE_BRM_um_S_Hemi_0minus3, Macula_3mm_Thk_RPE_BRM_um_I_Hemi_0minus3, Macula_3mm_Thk_RPE_BRM_um_All_0minus3, Macula_3mm_Thk_RPE_BRM_um_S_Hemi_field, Macula_3mm_Thk_RPE_BRM_um_I_Hemi_field, Macula_3mm_Thk_RPE_BRM_um_All_field, Macula_3mm_Vol_ILM_IPL_mm3_Center_1, Macula_3mm_Vol_ILM_IPL_mm3_T_1minus3, Macula_3mm_Vol_ILM_IPL_mm3_S_1minus3, Macula_3mm_Vol_ILM_IPL_mm3_N_1minus3, Macula_3mm_Vol_ILM_IPL_mm3_I_1minus3, Macula_3mm_Vol_ILM_IPL_mm3_S_Hemi_1minus3, Macula_3mm_Vol_ILM_IPL_mm3_I_Hemi_1minus3, Macula_3mm_Vol_ILM_IPL_mm3_All_1minus3, Macula_3mm_Vol_ILM_IPL_mm3_S_Hemi_0minus3, Macula_3mm_Vol_ILM_IPL_mm3_I_Hemi_0minus3, Macula_3mm_Vol_ILM_IPL_mm3_All_0minus3, Macula_3mm_Vol_ILM_IPL_mm3_S_Hemi_field, Macula_3mm_Vol_ILM_IPL_mm3_I_Hemi_field, Macula_3mm_Vol_ILM_IPL_mm3_All_field, Macula_3mm_Vol_ILM_RPE_mm3_Center_1, Macula_3mm_Vol_ILM_RPE_mm3_T_1minus3, Macula_3mm_Vol_ILM_RPE_mm3_S_1minus3, Macula_3mm_Vol_ILM_RPE_mm3_N_1minus3, Macula_3mm_Vol_ILM_RPE_mm3_I_1minus3, Macula_3mm_Vol_ILM_RPE_mm3_S_Hemi_1minus3, Macula_3mm_Vol_ILM_RPE_mm3_I_Hemi_1minus3, Macula_3mm_Vol_ILM_RPE_mm3_All_1minus3, Macula_3mm_Vol_ILM_RPE_mm3_S_Hemi_0minus3, Macula_3mm_Vol_ILM_RPE_mm3_I_Hemi_0minus3, Macula_3mm_Vol_ILM_RPE_mm3_All_0minus3, Macula_3mm_Vol_ILM_RPE_mm3_S_Hemi_field, Macula_3mm_Vol_ILM_RPE_mm3_I_Hemi_field, Macula_3mm_Vol_ILM_RPE_mm3_All_field, Macula_3mm_Vol_ILM_BRM_mm3_Center_1, Macula_3mm_Vol_ILM_BRM_mm3_T_1minus3, Macula_3mm_Vol_ILM_BRM_mm3_S_1minus3, Macula_3mm_Vol_ILM_BRM_mm3_N_1minus3, Macula_3mm_Vol_ILM_BRM_mm3_I_1minus3, Macula_3mm_Vol_ILM_BRM_mm3_S_Hemi_1minus3, Macula_3mm_Vol_ILM_BRM_mm3_I_Hemi_1minus3, Macula_3mm_Vol_ILM_BRM_mm3_All_1minus3, Macula_3mm_Vol_ILM_BRM_mm3_S_Hemi_0minus3, Macula_3mm_Vol_ILM_BRM_mm3_I_Hemi_0minus3, Macula_3mm_Vol_ILM_BRM_mm3_All_0minus3, Macula_3mm_Vol_ILM_BRM_mm3_S_Hemi_field, Macula_3mm_Vol_ILM_BRM_mm3_I_Hemi_field, Macula_3mm_Vol_ILM_BRM_mm3_All_field, Macula_3mm_Vol_RPE_BRM_mm3_Center_1, Macula_3mm_Vol_RPE_BRM_mm3_T_1minus3, Macula_3mm_Vol_RPE_BRM_mm3_S_1minus3, Macula_3mm_Vol_RPE_BRM_mm3_N_1minus3, Macula_3mm_Vol_RPE_BRM_mm3_I_1minus3, Macula_3mm_Vol_RPE_BRM_mm3_S_Hemi_1minus3, Macula_3mm_Vol_RPE_BRM_mm3_I_Hemi_1minus3, Macula_3mm_Vol_RPE_BRM_mm3_All_1minus3, Macula_3mm_Vol_RPE_BRM_mm3_S_Hemi_0minus3, Macula_3mm_Vol_RPE_BRM_mm3_I_Hemi_0minus3, Macula_3mm_Vol_RPE_BRM_mm3_All_0minus3, Macula_3mm_Vol_RPE_BRM_mm3_S_Hemi_field, Macula_3mm_Vol_RPE_BRM_mm3_I_Hemi_field, Macula_3mm_Vol_RPE_BRM_mm3_All_field, SVC_L1_DensityOfWhole_Image, SVC_L1_Whole_Image_S_Hemi, SVC_L1_Whole_Image_I_Hemi, SVC_L1_Whole_ETDRS, SVC_L1_Fovea, SVC_L1_ParaFovea, SVC_L1_Para_S_Hemi, SVC_L1_Para_I_Hemi, SVC_L1_Para_T, SVC_L1_Para_S, SVC_L1_Para_N, SVC_L1_Para_I, SVC_L1_G11, SVC_L1_G12, SVC_L1_G13, SVC_L1_G21, SVC_L1_G22, SVC_L1_G23, SVC_L1_G31, SVC_L1_G32, SVC_L1_G33, DVC_L2_DensityOfWhole_Image, DVC_L2_Whole_Image_S_Hemi, DVC_L2_Whole_Image_I_Hemi, DVC_L2_Whole_ETDRS, DVC_L2_Fovea, DVC_L2_ParaFovea, DVC_L2_Para_S_Hemi, DVC_L2_Para_I_Hemi, DVC_L2_Para_T, DVC_L2_Para_S, DVC_L2_Para_N, DVC_L2_Para_I, DVC_L2_G11, DVC_L2_G12, DVC_L2_G13, DVC_L2_G21, DVC_L2_G22, DVC_L2_G23, DVC_L2_G31, DVC_L2_G32, DVC_L2_G33, FAZ_Area, Perimeter, AcircularityIndex, FD_300_Area_Density, FD_300_Length_Density, FoveaInnRet_Thickness, InnRet_Thk_ParaFovea, InnRet_Thk_Para_S_Hemisphere, InnRet_Thk_Para_I_Hemisphere, InnRet_Thk_Para_Tempo, InnRet_Thk_Para_Superior, InnRet_Thk_Para_Nasal, InnRet_Thk_Para_Inferior, InnRet_Thk_PeriFovea, InnRet_Thk_Peri_S_Hemisphere, InnRet_Thk_Peri_I_Hemisphere, InnRet_Thk_Peri_Tempo, InnRet_Thk_Peri_Superior, InnRet_Thk_Peri_Nasal, InnRet_Thk_Peri_Inferior, FoveaInnRet_Volumn, InnRet_Vol_ParaFovea, InnRet_Vol_Para_S_Hemisphere, InnRet_Vol_Para_I_Hemisphere, InnRet_Vol_Para_Tempo, InnRet_Vol_Para_Superior, InnRet_Vol_Para_Nasal, InnRet_Vol_Para_Inferior, InnRet_Vol_PeriFovea, InnRet_Vol_Peri_S_Hemisphere, InnRet_Vol_Peri_I_Hemisphere, InnRet_Vol_Peri_Tempo, InnRet_Vol_Peri_Superior, InnRet_Vol_Peri_Nasal, InnRet_Vol_Peri_Inferior, FoveaFullRet_Thickness, FullRet_Thk_ParaFovea, FullRet_Thk_Para_S_Hemisphere, FullRet_Thk_Para_I_Hemisphere, FullRet_Thk_Para_Tempo, FullRet_Thk_Para_Superior, FullRet_Thk_Para_Nasal, FullRet_Thk_Para_Inferior, FullRet_Thk_PeriFovea, FullRet_Thk_Peri_S_Hemisphere, FullRet_Thk_Peri_I_Hemisphere, FullRet_Thk_Peri_Tempo, FullRet_Thk_Peri_Superior, FullRet_Thk_Peri_Nasal, FullRet_Thk_Peri_Inferior, FoveaFullRet_Volumn, FullRet_Vol_ParaFovea, FullRet_Vol_Para_S_Hemisphere, FullRet_Vol_Para_I_Hemisphere, FullRet_Vol_Para_Tempo, FullRet_Vol_Para_Superior, FullRet_Vol_Para_Nasal, FullRet_Vol_Para_Inferior, FullRet_Vol_PeriFovea, FullRet_Vol_Peri_S_Hemisphere, FullRet_Vol_Peri_I_Hemisphere, FullRet_Vol_Peri_Tempo, FullRet_Vol_Peri_Superior, FullRet_Vol_Peri_Nasal, FullRet_Vol_Peri_Inferior, FoveaRPE_Elevation_Height, RPE_Elev_ParaFovea_Tempo, RPE_Elev_Para_Superior, RPE_Elev_Para_Nasal, RPE_Elev_Para_Inferior, RPE_Elev_PeriFovea_Tempo, RPE_Elev_Peri_Superior, RPE_Elev_Peri_Nasal, RPE_Elev_Peri_Inferior, offset = AgePerToday

## Parameter Estimates

| Parameter | B | Std. Error | 95% Wald Confidence Interval | | Hypothesis Test | | |
| --- | --- | --- | --- | --- | --- | --- | --- |
|  |  |  | Lower | Upper | Wald Chi-Square | df | Sig. |
| (Intercept) | -52.488 | 8.9620 | -70.053 | -34.923 | 34.301 | 1 | **<.001** |
| [Gender_M2F1=1] | -.822 | .3711 | -1.549 | -.095 | 4.906 | 1 | **.027** |
| [Gender_M2F1=2] | 0^a^ | . | . | . | . | . |  |
| GCC_Inner_Retina_Average | 6.010 | 1.2475 | 3.565 | 8.455 | 23.209 | 1 | **<.001** |
| GCC_Superior_Avg | -2.775 | .9666 | -4.670 | -.881 | 8.245 | 1 | **.004** |
| GCC_Inferior_Avg | -2.200 | .4726 | -3.126 | -1.273 | 21.658 | 1 | **<.001** |
| GCC_S_I_Avg | -.528 | .3962 | -1.305 | .248 | 1.778 | 1 | .182 |
| GCC_FLV | -1.042 | .1604 | -1.357 | -.728 | 42.258 | 1 | **<.001** |
| GCC_GLV | -.231 | .0695 | -.367 | -.095 | 11.044 | 1 | **<.001** |
| GCC_RMS | 21.038 | 4.5985 | 12.025 | 30.051 | 20.930 | 1 | **<.001** |
| GCC_Full_Retina_Average | -1.126 | .9647 | -3.017 | .765 | 1.362 | 1 | .243 |
| GCC_Superior_Avg.1 | -.324 | .0526 | -.427 | -.220 | 37.805 | 1 | **<.001** |
| GCC_Inferior_Avg.1 | .394 | .0990 | .200 | .588 | 15.830 | 1 | **<.001** |
| GCC_S_I_Avg.1 | .172 | .0740 | .027 | .317 | 5.376 | 1 | **.020** |
| GCC_Outer_Retina_Average | 1.084 | .9683 | -.814 | 2.982 | 1.253 | 1 | .263 |
| GCC_Superior_Avg.2 | .145 | .1175 | -.085 | .375 | 1.519 | 1 | .218 |
| GCC_Inferior_Avg.2 | -.281 | .0443 | -.368 | -.194 | 40.254 | 1 | **<.001** |
| GCC_S_I_Avg.2 | .703 | .1795 | .351 | 1.055 | 15.342 | 1 | **<.001** |
| ONH_DiscArea | -12.433 | 1.5233 | -15.418 | -9.447 | 66.609 | 1 | **<.001** |
| ONH_Area_C_D_ratio | -10.208 | 4.9664 | -19.942 | -.474 | 4.225 | 1 | **.040** |
| ONH_H_C_D_ratio | 9.837 | 1.9978 | 5.921 | 13.753 | 24.245 | 1 | **<.001** |
| ONH_V_C_D_ratio | -1.531 | 2.3653 | -6.167 | 3.105 | .419 | 1 | .517 |
| ONH_CupArea | 6.165 | 1.8978 | 2.446 | 9.885 | 10.554 | 1 | **.001** |
| ONH_RimArea | 10.003 | 1.5991 | 6.869 | 13.138 | 39.131 | 1 | **<.001** |
| ONH_RimVolume | 1.515 | 5.3710 | -9.012 | 12.042 | .080 | 1 | .778 |
| ONH_Disc_Volume | .445 | 3.3290 | -6.080 | 6.970 | .018 | 1 | .894 |
| ONH_CupVolume | 17.598 | 2.1239 | 13.435 | 21.761 | 68.650 | 1 | **<.001** |
| ONH_Avg_RNFL | -.299 | .1138 | -.522 | -.076 | 6.901 | 1 | **.009** |
| ONH_Sup_RNFL | -1.621 | .1852 | -1.984 | -1.258 | 76.610 | 1 | **.000** |
| ONH_Inf_RNFL | .447 | .1058 | .239 | .654 | 17.819 | 1 | **<.001** |
| ONH_Tempo | -.196 | .1362 | -.463 | .071 | 2.060 | 1 | .151 |
| ONH_Superior | .002 | .0566 | -.109 | .113 | .001 | 1 | .976 |
| ONH_Nasal | -2.149 | .2968 | -2.731 | -1.567 | 52.422 | 1 | **<.001** |
| ONH_Inferior | -.394 | .0750 | -.541 | -.247 | 27.560 | 1 | **<.001** |
| ONH_RNFL_TU | .288 | .0710 | .149 | .427 | 16.426 | 1 | **<.001** |
| ONH_ST | .331 | .0600 | .214 | .449 | 30.469 | 1 | **<.001** |
| ONH_SN | .452 | .0754 | .304 | .599 | 35.878 | 1 | **<.001** |
| ONH_NU | .452 | .0836 | .288 | .616 | 29.194 | 1 | **<.001** |
| ONH_NL | .844 | .4764 | -.089 | 1.778 | 3.142 | 1 | .076 |
| ONH_IN | .145 | .0588 | .029 | .260 | 6.043 | 1 | **.014** |
| ONH_IT | .265 | .0611 | .145 | .384 | 18.720 | 1 | **<.001** |
| ONH_TL | -.164 | .0846 | -.330 | .001 | 3.779 | 1 | .052 |
| ONH_TU1 | .133 | .0392 | .056 | .209 | 11.442 | 1 | **<.001** |
| ONH_TU2 | .071 | .0350 | .003 | .140 | 4.165 | 1 | **.041** |
| ONH_ST2 | .021 | .0198 | -.018 | .060 | 1.131 | 1 | .288 |
| ONH_ST1 | .075 | .0191 | .037 | .112 | 15.266 | 1 | **<.001** |
| ONH_SN1 | -.060 | .0241 | -.107 | -.013 | 6.220 | 1 | **.013** |
| ONH_SN2 | -.087 | .0254 | -.137 | -.038 | 11.823 | 1 | **<.001** |
| ONH_NU2 | .549 | .0749 | .402 | .695 | 53.676 | 1 | **<.001** |
| ONH_NU1 | .528 | .0884 | .354 | .701 | 35.657 | 1 | **<.001** |
| ONH_NL1 | .071 | .2346 | -.389 | .531 | .092 | 1 | .762 |
| ONH_NL2 | .047 | .2366 | -.417 | .511 | .040 | 1 | .842 |
| ONH_IN2 | -.015 | .0267 | -.067 | .037 | .308 | 1 | .579 |
| ONH_IN1 | .042 | .0275 | -.012 | .095 | 2.281 | 1 | .131 |
| ONH_IT1 | -.092 | .0264 | -.144 | -.040 | 12.115 | 1 | **<.001** |
| ONH_IT2 | -.015 | .0253 | -.065 | .034 | .361 | 1 | .548 |
| ONH_TL2 | .106 | .0475 | .013 | .199 | 5.032 | 1 | **.025** |
| ONH_TL1 | -.068 | .0542 | -.175 | .038 | 1.591 | 1 | .207 |
| Macula_3mm_Thk_ILM_IPL_um_Center_1 | 2.796 | 1.0292 | .779 | 4.813 | 7.382 | 1 | **.007** |
| Macula_3mm_Thk_ILM_IPL_um_T_1minus3 | 2.392 | 1.6169 | -.777 | 5.561 | 2.190 | 1 | .139 |
| Macula_3mm_Thk_ILM_IPL_um_S_1minus3 | -4.008 | 1.5006 | -6.949 | -1.067 | 7.134 | 1 | **.008** |
| Macula_3mm_Thk_ILM_IPL_um_N_1minus3 | -.553 | 1.3247 | -3.150 | 2.043 | .175 | 1 | .676 |
| Macula_3mm_Thk_ILM_IPL_um_I_1minus3 | -.321 | 1.6606 | -3.575 | 2.934 | .037 | 1 | .847 |
| Macula_3mm_Thk_ILM_IPL_um_S_Hemi_1minus3 | 9.612 | 3.9456 | 1.879 | 17.345 | 5.935 | 1 | **.015** |
| Macula_3mm_Thk_ILM_IPL_um_I_Hemi_1minus3 | 1.363 | 4.0224 | -6.520 | 9.247 | .115 | 1 | .735 |
| Macula_3mm_Thk_ILM_IPL_um_All_1minus3 | 23.380 | 4.7496 | 14.071 | 32.689 | 24.230 | 1 | **<.001** |
| Macula_3mm_Thk_ILM_IPL_um_S_Hemi_0minus3 | 12.411 | 4.5903 | 3.414 | 21.408 | 7.310 | 1 | **.007** |
| Macula_3mm_Thk_ILM_IPL_um_I_Hemi_0minus3 | -12.599 | 4.3156 | -21.057 | -4.141 | 8.523 | 1 | **.004** |
| Macula_3mm_Thk_ILM_IPL_um_All_0minus3 | -44.301 | 4.5314 | -53.183 | -35.420 | 95.579 | 1 | **.000** |
| Macula_3mm_Thk_ILM_IPL_um_S_Hemi_field | -9.023 | 1.4001 | -11.767 | -6.279 | 41.535 | 1 | **<.001** |
| Macula_3mm_Thk_ILM_IPL_um_I_Hemi_field | -4.762 | 1.5805 | -7.860 | -1.665 | 9.080 | 1 | **.003** |
| Macula_3mm_Thk_ILM_IPL_um_All_field | 9.486 | 4.2402 | 1.176 | 17.797 | 5.005 | 1 | **.025** |
| Macula_3mm_Thk_ILM_RPE_um_Center_1 | -9.978 | 2.9773 | -15.813 | -4.142 | 11.231 | 1 | **<.001** |
| Macula_3mm_Thk_ILM_RPE_um_T_1minus3 | 8.465 | 3.0374 | 2.512 | 14.418 | 7.766 | 1 | **.005** |
| Macula_3mm_Thk_ILM_RPE_um_S_1minus3 | 8.397 | 3.9378 | .679 | 16.115 | 4.548 | 1 | **.033** |
| Macula_3mm_Thk_ILM_RPE_um_N_1minus3 | 8.779 | 3.2644 | 2.381 | 15.177 | 7.233 | 1 | **.007** |
| Macula_3mm_Thk_ILM_RPE_um_I_1minus3 | -8.197 | 3.4401 | -14.940 | -1.455 | 5.678 | 1 | **.017** |
| Macula_3mm_Thk_ILM_RPE_um_S_Hemi_1minus3 | 24.456 | 4.5767 | 15.486 | 33.426 | 28.553 | 1 | **<.001** |
| Macula_3mm_Thk_ILM_RPE_um_I_Hemi_1minus3 | 13.471 | 4.0955 | 5.444 | 21.498 | 10.819 | 1 | **.001** |
| Macula_3mm_Thk_ILM_RPE_um_All_1minus3 | -29.324 | 5.1596 | -39.436 | -19.211 | 32.301 | 1 | **<.001** |
| Macula_3mm_Thk_ILM_RPE_um_S_Hemi_0minus3 | -35.001 | 4.1724 | -43.179 | -26.823 | 70.371 | 1 | **.000** |
| Macula_3mm_Thk_ILM_RPE_um_I_Hemi_0minus3 | 9.108 | 5.0728 | -.834 | 19.051 | 3.224 | 1 | .073 |
| Macula_3mm_Thk_ILM_RPE_um_All_0minus3 | 27.795 | 5.3176 | 17.373 | 38.217 | 27.322 | 1 | **<.001** |
| Macula_3mm_Thk_ILM_RPE_um_S_Hemi_field | 14.295 | 3.3946 | 7.642 | 20.949 | 17.735 | 1 | **<.001** |
| Macula_3mm_Thk_ILM_RPE_um_I_Hemi_field | -21.511 | 3.7891 | -28.937 | -14.084 | 32.228 | 1 | **<.001** |
| Macula_3mm_Thk_ILM_RPE_um_All_field | -3.064 | 4.9202 | -12.707 | 6.579 | .388 | 1 | .533 |
| Macula_3mm_Thk_ILM_BRM_um_Center_1 | 11.530 | 2.8586 | 5.927 | 17.133 | 16.268 | 1 | **<.001** |
| Macula_3mm_Thk_ILM_BRM_um_T_1minus3 | -8.234 | 3.0092 | -14.132 | -2.336 | 7.487 | 1 | **.006** |
| Macula_3mm_Thk_ILM_BRM_um_S_1minus3 | -6.627 | 3.7493 | -13.975 | .722 | 3.124 | 1 | .077 |
| Macula_3mm_Thk_ILM_BRM_um_N_1minus3 | -8.354 | 3.3377 | -14.895 | -1.812 | 6.264 | 1 | **.012** |
| Macula_3mm_Thk_ILM_BRM_um_I_1minus3 | 8.176 | 3.4947 | 1.327 | 15.025 | 5.474 | 1 | **.019** |
| Macula_3mm_Thk_ILM_BRM_um_S_Hemi_1minus3 | -3.939 | 4.1410 | -12.055 | 4.178 | .905 | 1 | .342 |
| Macula_3mm_Thk_ILM_BRM_um_I_Hemi_1minus3 | .967 | 4.3932 | -7.643 | 9.578 | .048 | 1 | .826 |
| Macula_3mm_Thk_ILM_BRM_um_All_1minus3 | 9.486 | 4.5216 | .624 | 18.348 | 4.402 | 1 | **.036** |
| Macula_3mm_Thk_ILM_BRM_um_S_Hemi_0minus3 | 12.133 | 4.6747 | 2.971 | 21.295 | 6.736 | 1 | **.009** |
| Macula_3mm_Thk_ILM_BRM_um_I_Hemi_0minus3 | -12.331 | 4.8166 | -21.772 | -2.891 | 6.555 | 1 | **.010** |
| Macula_3mm_Thk_ILM_BRM_um_All_0minus3 | -17.775 | 4.7793 | -27.142 | -8.408 | 13.832 | 1 | **<.001** |
| Macula_3mm_Thk_ILM_BRM_um_S_Hemi_field | -12.116 | 3.2364 | -18.459 | -5.772 | 14.014 | 1 | **<.001** |
| Macula_3mm_Thk_ILM_BRM_um_I_Hemi_field | 20.739 | 3.7620 | 13.366 | 28.113 | 30.392 | 1 | **<.001** |
| Macula_3mm_Thk_ILM_BRM_um_All_field | -31.265 | 4.0824 | -39.267 | -23.264 | 58.653 | 1 | **<.001** |
| Macula_3mm_Thk_RPE_BRM_um_Center_1 | -5.504 | 3.0024 | -11.389 | .380 | 3.361 | 1 | .067 |
| Macula_3mm_Thk_RPE_BRM_um_T_1minus3 | 7.121 | 3.6586 | -.050 | 14.292 | 3.788 | 1 | .052 |
| Macula_3mm_Thk_RPE_BRM_um_S_1minus3 | 11.683 | 4.3975 | 3.064 | 20.301 | 7.058 | 1 | **.008** |
| Macula_3mm_Thk_RPE_BRM_um_N_1minus3 | 16.243 | 3.4049 | 9.570 | 22.917 | 22.758 | 1 | **<.001** |
| Macula_3mm_Thk_RPE_BRM_um_I_1minus3 | -4.916 | 3.9241 | -12.607 | 2.775 | 1.569 | 1 | .210 |
| Macula_3mm_Thk_RPE_BRM_um_S_Hemi_1minus3 | -9.532 | 4.2999 | -17.960 | -1.104 | 4.914 | 1 | **.027** |
| Macula_3mm_Thk_RPE_BRM_um_I_Hemi_1minus3 | .750 | 4.2300 | -7.540 | 9.041 | .031 | 1 | .859 |
| Macula_3mm_Thk_RPE_BRM_um_All_1minus3 | 7.244 | 5.3306 | -3.204 | 17.692 | 1.847 | 1 | .174 |
| Macula_3mm_Thk_RPE_BRM_um_S_Hemi_0minus3 | -19.936 | 4.7701 | -29.286 | -10.587 | 17.468 | 1 | **<.001** |
| Macula_3mm_Thk_RPE_BRM_um_I_Hemi_0minus3 | -7.255 | 4.4458 | -15.969 | 1.459 | 2.663 | 1 | .103 |
| Macula_3mm_Thk_RPE_BRM_um_All_0minus3 | 5.599 | 5.3452 | -4.878 | 16.075 | 1.097 | 1 | .295 |
| Macula_3mm_Thk_RPE_BRM_um_S_Hemi_field | 21.885 | 3.2913 | 15.434 | 28.336 | 44.212 | 1 | **<.001** |
| Macula_3mm_Thk_RPE_BRM_um_I_Hemi_field | -27.106 | 4.3985 | -35.727 | -18.486 | 37.979 | 1 | **<.001** |
| Macula_3mm_Thk_RPE_BRM_um_All_field | 4.057 | 4.2914 | -4.354 | 12.468 | .894 | 1 | .344 |
| Macula_3mm_Vol_ILM_IPL_mm3_Center_1 | 250.255 | 532.0681 | -792.579 | 1293.089 | .221 | 1 | .638 |
| Macula_3mm_Vol_ILM_IPL_mm3_T_1minus3 | -3397.386 | 470.5706 | -4319.687 | -2475.084 | 52.124 | 1 | **<.001** |
| Macula_3mm_Vol_ILM_IPL_mm3_S_1minus3 | -147.353 | 543.5590 | -1212.709 | 918.004 | .073 | 1 | .786 |
| Macula_3mm_Vol_ILM_IPL_mm3_N_1minus3 | -1656.197 | 371.1110 | -2383.561 | -928.833 | 19.917 | 1 | **<.001** |
| Macula_3mm_Vol_ILM_IPL_mm3_I_1minus3 | -1370.434 | 452.0818 | -2256.498 | -484.370 | 9.189 | 1 | **.002** |
| Macula_3mm_Vol_ILM_IPL_mm3_S_Hemi_1minus3 | -2467.828 | 449.5443 | -3348.919 | -1586.738 | 30.136 | 1 | **<.001** |
| Macula_3mm_Vol_ILM_IPL_mm3_I_Hemi_1minus3 | 3231.718 | 515.0279 | 2222.282 | 4241.154 | 39.374 | 1 | **<.001** |
| Macula_3mm_Vol_ILM_IPL_mm3_All_1minus3 | -346.944 | 532.2630 | -1390.161 | 696.272 | .425 | 1 | .515 |
| Macula_3mm_Vol_ILM_IPL_mm3_S_Hemi_0minus3 | 157.155 | 489.2320 | -801.722 | 1116.032 | .103 | 1 | .748 |
| Macula_3mm_Vol_ILM_IPL_mm3_I_Hemi_0minus3 | 2864.254 | 541.8307 | 1802.285 | 3926.222 | 27.944 | 1 | **<.001** |
| Macula_3mm_Vol_ILM_IPL_mm3_All_0minus3 | 1612.583 | 466.8518 | 697.570 | 2527.596 | 11.931 | 1 | **<.001** |
| Macula_3mm_Vol_ILM_IPL_mm3_S_Hemi_field | -629.785 | 362.4149 | -1340.106 | 80.535 | 3.020 | 1 | .082 |
| Macula_3mm_Vol_ILM_IPL_mm3_I_Hemi_field | -1121.117 | 400.5652 | -1906.211 | -336.024 | 7.833 | 1 | **.005** |
| Macula_3mm_Vol_ILM_IPL_mm3_All_field | 1122.302 | 458.4509 | 223.755 | 2020.849 | 5.993 | 1 | **.014** |
| Macula_3mm_Vol_ILM_RPE_mm3_Center_1 | 2859.331 | 489.5227 | 1899.884 | 3818.777 | 34.118 | 1 | **<.001** |
| Macula_3mm_Vol_ILM_RPE_mm3_T_1minus3 | -1405.660 | 409.1693 | -2207.617 | -603.703 | 11.802 | 1 | **<.001** |
| Macula_3mm_Vol_ILM_RPE_mm3_S_1minus3 | -4.319 | 447.9531 | -882.291 | 873.653 | .000 | 1 | .992 |
| Macula_3mm_Vol_ILM_RPE_mm3_N_1minus3 | 623.417 | 374.7144 | -111.009 | 1357.844 | 2.768 | 1 | .096 |
| Macula_3mm_Vol_ILM_RPE_mm3_I_1minus3 | 550.541 | 420.9645 | -274.534 | 1375.617 | 1.710 | 1 | .191 |
| Macula_3mm_Vol_ILM_RPE_mm3_S_Hemi_1minus3 | 208.406 | 584.5325 | -937.257 | 1354.068 | .127 | 1 | .721 |
| Macula_3mm_Vol_ILM_RPE_mm3_I_Hemi_1minus3 | -3275.840 | 512.6205 | -4280.558 | -2271.122 | 40.837 | 1 | **<.001** |
| Macula_3mm_Vol_ILM_RPE_mm3_All_1minus3 | 2085.324 | 517.9917 | 1070.079 | 3100.569 | 16.207 | 1 | **<.001** |
| Macula_3mm_Vol_ILM_RPE_mm3_S_Hemi_0minus3 | -2378.815 | 493.7497 | -3346.546 | -1411.083 | 23.212 | 1 | **<.001** |
| Macula_3mm_Vol_ILM_RPE_mm3_I_Hemi_0minus3 | -783.112 | 477.8970 | -1719.773 | 153.548 | 2.685 | 1 | **.101** |
| Macula_3mm_Vol_ILM_RPE_mm3_All_0minus3 | -2348.882 | 442.6835 | -3216.526 | -1481.238 | 28.154 | 1 | **<.001** |
| Macula_3mm_Vol_ILM_RPE_mm3_S_Hemi_field | 1305.253 | 455.1775 | 413.121 | 2197.384 | 8.223 | 1 | **.004** |
| Macula_3mm_Vol_ILM_RPE_mm3_I_Hemi_field | 3097.026 | 426.8320 | 2260.451 | 3933.601 | 52.647 | 1 | **<.001** |
| Macula_3mm_Vol_ILM_RPE_mm3_All_field | -1397.786 | 513.3934 | -2404.019 | -391.554 | 7.413 | 1 | **.006** |
| Macula_3mm_Vol_ILM_BRM_mm3_Center_1 | 1266.873 | 578.4011 | 133.228 | 2400.518 | 4.797 | 1 | **.029** |
| Macula_3mm_Vol_ILM_BRM_mm3_T_1minus3 | 2280.950 | 379.9730 | 1536.217 | 3025.684 | 36.035 | 1 | **<.001** |
| Macula_3mm_Vol_ILM_BRM_mm3_S_1minus3 | -567.112 | 462.9009 | -1474.381 | 340.157 | 1.501 | 1 | .221 |
| Macula_3mm_Vol_ILM_BRM_mm3_N_1minus3 | -171.397 | 449.1322 | -1051.680 | 708.886 | .146 | 1 | .703 |
| Macula_3mm_Vol_ILM_BRM_mm3_I_1minus3 | 318.298 | 416.4932 | -498.013 | 1134.610 | .584 | 1 | .445 |
| Macula_3mm_Vol_ILM_BRM_mm3_S_Hemi_1minus3 | -1847.703 | 587.1735 | -2998.542 | -696.864 | 9.902 | 1 | **.002** |
| Macula_3mm_Vol_ILM_BRM_mm3_I_Hemi_1minus3 | -1270.098 | 537.1478 | -2322.888 | -217.307 | 5.591 | 1 | **.018** |
| Macula_3mm_Vol_ILM_BRM_mm3_All_1minus3 | 4601.898 | 525.5246 | 3571.889 | 5631.907 | 76.681 | 1 | **.000** |
| Macula_3mm_Vol_ILM_BRM_mm3_S_Hemi_0minus3 | 1502.696 | 535.3830 | 453.365 | 2552.027 | 7.878 | 1 | **.005** |
| Macula_3mm_Vol_ILM_BRM_mm3_I_Hemi_0minus3 | -468.723 | 469.8993 | -1389.709 | 452.263 | .995 | 1 | .319 |
| Macula_3mm_Vol_ILM_BRM_mm3_All_0minus3 | -989.355 | 551.2362 | -2069.758 | 91.048 | 3.221 | 1 | .073 |
| Macula_3mm_Vol_ILM_BRM_mm3_S_Hemi_field | 1019.737 | 438.2335 | 160.815 | 1878.659 | 5.415 | 1 | **.020** |
| Macula_3mm_Vol_ILM_BRM_mm3_I_Hemi_field | -749.457 | 458.6365 | -1648.368 | 149.454 | 2.670 | 1 | .102 |
| Macula_3mm_Vol_ILM_BRM_mm3_All_field | 2666.472 | 526.5706 | 1634.413 | 3698.532 | 25.643 | 1 | **<.001** |
| Macula_3mm_Vol_RPE_BRM_mm3_Center_1 | -3038.161 | 579.0372 | -4173.053 | -1903.269 | 27.530 | 1 | **<.001** |
| Macula_3mm_Vol_RPE_BRM_mm3_T_1minus3 | 1304.026 | 499.5699 | 324.886 | 2283.165 | 6.814 | 1 | **.009** |
| Macula_3mm_Vol_RPE_BRM_mm3_S_1minus3 | -122.392 | 519.7323 | -1141.048 | 896.265 | .055 | 1 | .814 |
| Macula_3mm_Vol_RPE_BRM_mm3_N_1minus3 | -1811.982 | 525.7460 | -2842.425 | -781.538 | 11.878 | 1 | **<.001** |
| Macula_3mm_Vol_RPE_BRM_mm3_I_1minus3 | 384.008 | 560.2088 | -713.981 | 1481.997 | .470 | 1 | .493 |
| Macula_3mm_Vol_RPE_BRM_mm3_S_Hemi_1minus3 | -4355.278 | 618.6668 | -5567.843 | -3142.713 | 49.559 | 1 | **<.001** |
| Macula_3mm_Vol_RPE_BRM_mm3_I_Hemi_1minus3 | -205.039 | 487.0167 | -1159.575 | 749.496 | .177 | 1 | .674 |
| Macula_3mm_Vol_RPE_BRM_mm3_All_1minus3 | 1227.461 | 527.2489 | 194.072 | 2260.849 | 5.420 | 1 | **.020** |
| Macula_3mm_Vol_RPE_BRM_mm3_S_Hemi_0minus3 | 2984.190 | 537.3460 | 1931.011 | 4037.368 | 30.842 | 1 | **<.001** |
| Macula_3mm_Vol_RPE_BRM_mm3_I_Hemi_0minus3 | 182.497 | 550.1226 | -895.724 | 1260.717 | .110 | 1 | .740 |
| Macula_3mm_Vol_RPE_BRM_mm3_All_0minus3 | -1748.467 | 489.5849 | -2708.036 | -788.898 | 12.754 | 1 | **<.001** |
| Macula_3mm_Vol_RPE_BRM_mm3_S_Hemi_field | -2349.237 | 438.8965 | -3209.458 | -1489.016 | 28.650 | 1 | **<.001** |
| Macula_3mm_Vol_RPE_BRM_mm3_I_Hemi_field | 1723.155 | 384.2166 | 970.104 | 2476.206 | 20.114 | 1 | **<.001** |
| Macula_3mm_Vol_RPE_BRM_mm3_All_field | 652.111 | 439.1480 | -208.604 | 1512.825 | 2.205 | 1 | .138 |
| SVC_L1_DensityOfWhole_Image | 54.888 | 27.7124 | .573 | 109.203 | 3.923 | 1 | **.048** |
| SVC_L1_Whole_Image_S_Hemi | 13.472 | 3.6058 | 6.405 | 20.539 | 13.959 | 1 | **<.001** |
| SVC_L1_Whole_Image_I_Hemi | 14.241 | 3.8764 | 6.644 | 21.839 | 13.498 | 1 | **<.001** |
| SVC_L1_Whole_ETDRS | 103.322 | 30.6663 | 43.217 | 163.427 | 11.352 | 1 | **<.001** |
| SVC_L1_Fovea | -12.044 | 3.4945 | -18.893 | -5.195 | 11.879 | 1 | **<.001** |
| SVC_L1_ParaFovea | 25.182 | 35.3983 | -44.197 | 94.562 | .506 | 1 | .477 |
| SVC_L1_Para_S_Hemi | -37.706 | 15.3943 | -67.878 | -7.534 | 5.999 | 1 | **.014** |
| SVC_L1_Para_I_Hemi | -38.298 | 15.5323 | -68.741 | -7.855 | 6.080 | 1 | **.014** |
| SVC_L1_Para_T | -10.851 | 2.3116 | -15.382 | -6.320 | 22.035 | 1 | **<.001** |
| SVC_L1_Para_S | -11.033 | 2.2832 | -15.508 | -6.558 | 23.349 | 1 | **<.001** |
| SVC_L1_Para_N | -10.574 | 2.2886 | -15.059 | -6.088 | 21.347 | 1 | **<.001** |
| SVC_L1_Para_I | -10.238 | 2.2797 | -14.707 | -5.770 | 20.170 | 1 | **<.001** |
| SVC_L1_G11 | -8.737 | 3.0628 | -14.740 | -2.734 | 8.137 | 1 | **.004** |
| SVC_L1_G12 | -9.175 | 3.0254 | -15.105 | -3.245 | 9.196 | 1 | **.002** |
| SVC_L1_G13 | -8.793 | 3.0362 | -14.744 | -2.842 | 8.387 | 1 | **.004** |
| SVC_L1_G21 | -8.776 | 3.0381 | -14.730 | -2.821 | 8.344 | 1 | **.004** |
| SVC_L1_G22 | -9.102 | 3.0047 | -14.991 | -3.213 | 9.176 | 1 | **.002** |
| SVC_L1_G23 | -8.787 | 3.0046 | -14.676 | -2.899 | 8.554 | 1 | **.003** |
| SVC_L1_G31 | -8.973 | 3.0534 | -14.958 | -2.989 | 8.636 | 1 | **.003** |
| SVC_L1_G32 | -9.298 | 3.0286 | -15.234 | -3.362 | 9.426 | 1 | **.002** |
| SVC_L1_G33 | -8.752 | 3.0248 | -14.680 | -2.823 | 8.371 | 1 | **.004** |
| DVC_L2_DensityOfWhole_Image | 117.574 | 45.0176 | 29.341 | 205.806 | 6.821 | 1 | **.009** |
| DVC_L2_Whole_Image_S_Hemi | 3.656 | 2.8382 | -1.906 | 9.219 | 1.660 | 1 | .198 |
| DVC_L2_Whole_Image_I_Hemi | 3.263 | 3.0267 | -2.669 | 9.196 | 1.163 | 1 | .281 |
| DVC_L2_Whole_ETDRS | -120.021 | 27.1866 | -173.306 | -66.736 | 19.490 | 1 | **<.001** |
| DVC_L2_Fovea | 13.406 | 3.1016 | 7.327 | 19.485 | 18.683 | 1 | **<.001** |
| DVC_L2_ParaFovea | 52.750 | 29.6647 | -5.392 | 110.892 | 3.162 | 1 | .075 |
| DVC_L2_Para_S_Hemi | 32.255 | 12.0468 | 8.644 | 55.866 | 7.169 | 1 | **.007** |
| DVC_L2_Para_I_Hemi | 33.322 | 12.1263 | 9.555 | 57.090 | 7.551 | 1 | **.006** |
| DVC_L2_Para_T | -2.641 | 2.1605 | -6.876 | 1.593 | 1.494 | 1 | .222 |
| DVC_L2_Para_S | -3.129 | 2.1451 | -7.333 | 1.075 | 2.128 | 1 | .145 |
| DVC_L2_Para_N | -2.778 | 2.1668 | -7.025 | 1.469 | 1.644 | 1 | .200 |
| DVC_L2_Para_I | -2.619 | 2.0981 | -6.731 | 1.493 | 1.558 | 1 | .212 |
| DVC_L2_G11 | -14.048 | 5.1268 | -24.096 | -3.999 | 7.508 | 1 | **.006** |
| DVC_L2_G12 | -13.480 | 5.0886 | -23.453 | -3.506 | 7.017 | 1 | **.008** |
| DVC_L2_G13 | -14.053 | 5.0875 | -24.025 | -4.082 | 7.630 | 1 | **.006** |
| DVC_L2_G21 | -13.667 | 5.0579 | -23.581 | -3.754 | 7.301 | 1 | **.007** |
| DVC_L2_G22 | -13.508 | 4.9937 | -23.295 | -3.720 | 7.317 | 1 | **.007** |
| DVC_L2_G23 | -14.379 | 5.0182 | -24.215 | -4.544 | 8.211 | 1 | **.004** |
| DVC_L2_G31 | -14.391 | 5.0684 | -24.325 | -4.457 | 8.062 | 1 | **.005** |
| DVC_L2_G32 | -13.702 | 5.0286 | -23.557 | -3.846 | 7.424 | 1 | **.006** |
| DVC_L2_G33 | -13.771 | 5.0203 | -23.611 | -3.932 | 7.525 | 1 | **.006** |
| FAZ_Area | -32.070 | 9.9700 | -51.611 | -12.529 | 10.347 | 1 | **.001** |
| Perimeter | 6.878 | 2.3730 | 2.227 | 11.529 | 8.400 | 1 | **.004** |
| AcircularityIndex | -24.291 | 5.7550 | -35.571 | -13.012 | 17.816 | 1 | **<.001** |
| FD_300_Area_Density | -.255 | .0940 | -.439 | -.071 | 7.349 | 1 | **.007** |
| FD_300_Length_Density | .774 | .1984 | .385 | 1.163 | 15.229 | 1 | **<.001** |
| FoveaInnRet_Thickness | .388 | .2830 | -.167 | .942 | 1.878 | 1 | .171 |
| InnRet_Thk_ParaFovea | .716 | .5060 | -.276 | 1.708 | 2.001 | 1 | .157 |
| InnRet_Thk_Para_S_Hemisphere | -.126 | .3901 | -.891 | .638 | .105 | 1 | .746 |
| InnRet_Thk_Para_I_Hemisphere | -.484 | .4730 | -1.411 | .444 | 1.045 | 1 | .307 |
| InnRet_Thk_Para_Tempo | 1.502 | .3638 | .789 | 2.215 | 17.047 | 1 | **<.001** |
| InnRet_Thk_Para_Superior | -.439 | .3221 | -1.071 | .192 | 1.860 | 1 | .173 |
| InnRet_Thk_Para_Nasal | 1.162 | .2290 | .713 | 1.610 | 25.731 | 1 | **<.001** |
| InnRet_Thk_Para_Inferior | -.629 | .3793 | -1.372 | .115 | 2.749 | 1 | .097 |
| InnRet_Thk_PeriFovea | .707 | .4367 | -.149 | 1.562 | 2.618 | 1 | .106 |
| InnRet_Thk_Peri_S_Hemisphere | -.967 | .5092 | -1.965 | .031 | 3.608 | 1 | .058 |
| InnRet_Thk_Peri_I_Hemisphere | -2.663 | .4925 | -3.628 | -1.698 | 29.243 | 1 | **<.001** |
| InnRet_Thk_Peri_Tempo | -1.310 | .3750 | -2.045 | -.575 | 12.198 | 1 | **<.001** |
| InnRet_Thk_Peri_Superior | -.408 | .3208 | -1.036 | .221 | 1.614 | 1 | .204 |
| InnRet_Thk_Peri_Nasal | .083 | .2581 | -.423 | .589 | .104 | 1 | .747 |
| InnRet_Thk_Peri_Inferior | 1.986 | .2745 | 1.448 | 2.524 | 52.348 | 1 | **<.001** |
| FoveaInnRet_Volumn | -566.574 | 353.0635 | -1258.566 | 125.418 | 2.575 | 1 | .109 |
| InnRet_Vol_ParaFovea | 405.972 | 259.0194 | -101.697 | 913.641 | 2.457 | 1 | .117 |
| InnRet_Vol_Para_S_Hemisphere | 7.068 | 291.2841 | -563.838 | 577.974 | .001 | 1 | .981 |
| InnRet_Vol_Para_I_Hemisphere | -30.599 | 272.4713 | -564.633 | 503.435 | .013 | 1 | .911 |
| InnRet_Vol_Para_Tempo | -1354.377 | 303.2389 | -1948.715 | -760.040 | 19.948 | 1 | **<.001** |
| InnRet_Vol_Para_Superior | -37.234 | 294.3573 | -614.164 | 539.696 | .016 | 1 | .899 |
| InnRet_Vol_Para_Nasal | -1253.960 | 271.9622 | -1786.996 | -720.924 | 21.259 | 1 | **<.001** |
| InnRet_Vol_Para_Inferior | 69.151 | 159.2070 | -242.889 | 381.191 | .189 | 1 | .664 |
| InnRet_Vol_PeriFovea | 44.914 | 217.3608 | -381.105 | 470.933 | .043 | 1 | .836 |
| InnRet_Vol_Peri_S_Hemisphere | 203.942 | 316.3783 | -416.148 | 824.032 | .416 | 1 | .519 |
| InnRet_Vol_Peri_I_Hemisphere | 601.605 | 306.8639 | .163 | 1203.047 | 3.844 | 1 | .050 |
| InnRet_Vol_Peri_Tempo | 199.163 | 294.9056 | -378.841 | 777.168 | .456 | 1 | .499 |
| InnRet_Vol_Peri_Superior | -118.555 | 295.8883 | -698.486 | 461.375 | .161 | 1 | .689 |
| InnRet_Vol_Peri_Nasal | -76.268 | 297.7458 | -659.839 | 507.303 | .066 | 1 | .798 |
| InnRet_Vol_Peri_Inferior | -964.772 | 303.1902 | -1559.014 | -370.530 | 10.126 | 1 | **.001** |
| FoveaFullRet_Thickness | .608 | .1888 | .238 | .978 | 10.356 | 1 | **.001** |
| FullRet_Thk_ParaFovea | 2.128 | .4591 | 1.228 | 3.027 | 21.478 | 1 | **<.001** |
| FullRet_Thk_Para_S_Hemisphere | .895 | .3769 | .157 | 1.634 | 5.645 | 1 | **.018** |
| FullRet_Thk_Para_I_Hemisphere | -.106 | .3065 | -.706 | .495 | .119 | 1 | .730 |
| FullRet_Thk_Para_Tempo | -1.484 | .2222 | -1.920 | -1.049 | 44.621 | 1 | **<.001** |
| FullRet_Thk_Para_Superior | -2.022 | .2596 | -2.531 | -1.513 | 60.639 | 1 | **<.001** |
| FullRet_Thk_Para_Nasal | -.263 | .1663 | -.589 | .063 | 2.500 | 1 | .114 |
| FullRet_Thk_Para_Inferior | -.091 | .1820 | -.448 | .266 | .249 | 1 | .618 |
| FullRet_Thk_PeriFovea | 1.199 | .3049 | .601 | 1.797 | 15.459 | 1 | **<.001** |
| FullRet_Thk_Peri_S_Hemisphere | -.449 | .3342 | -1.104 | .207 | 1.801 | 1 | .180 |
| FullRet_Thk_Peri_I_Hemisphere | -.020 | .3181 | -.643 | .604 | .004 | 1 | .951 |
| FullRet_Thk_Peri_Tempo | 1.046 | .2129 | .629 | 1.464 | 24.167 | 1 | **<.001** |
| FullRet_Thk_Peri_Superior | -.009 | .1541 | -.311 | .293 | .004 | 1 | .952 |
| FullRet_Thk_Peri_Nasal | -.670 | .1750 | -1.013 | -.327 | 14.639 | 1 | **<.001** |
| FullRet_Thk_Peri_Inferior | -.497 | .1434 | -.778 | -.216 | 12.021 | 1 | **<.001** |
| FoveaFullRet_Volumn | -218.834 | 241.4478 | -692.063 | 254.395 | .821 | 1 | .365 |
| FullRet_Vol_ParaFovea | 1017.921 | 369.1388 | 294.422 | 1741.420 | 7.604 | 1 | **.006** |
| FullRet_Vol_Para_S_Hemisphere | -2603.517 | 321.1411 | -3232.942 | -1974.092 | 65.725 | 1 | **<.001** |
| FullRet_Vol_Para_I_Hemisphere | -1822.606 | 361.4128 | -2530.962 | -1114.249 | 25.432 | 1 | **<.001** |
| FullRet_Vol_Para_Tempo | 1240.608 | 270.3072 | 710.815 | 1770.400 | 21.065 | 1 | **<.001** |
| FullRet_Vol_Para_Superior | 2288.009 | 350.2611 | 1601.510 | 2974.508 | 42.671 | 1 | **<.001** |
| FullRet_Vol_Para_Nasal | 778.813 | 255.5589 | 277.927 | 1279.699 | 9.287 | 1 | **.002** |
| FullRet_Vol_Para_Inferior | 638.804 | 281.8229 | 86.441 | 1191.167 | 5.138 | 1 | **.023** |
| FullRet_Vol_PeriFovea | 278.177 | 97.3767 | 87.322 | 469.031 | 8.161 | 1 | **.004** |
| FullRet_Vol_Peri_S_Hemisphere | -173.130 | 211.6858 | -588.026 | 241.767 | .669 | 1 | .413 |
| FullRet_Vol_Peri_I_Hemisphere | -123.004 | 211.1342 | -536.819 | 290.811 | .339 | 1 | .560 |
| FullRet_Vol_Peri_Tempo | -494.037 | 214.4569 | -914.364 | -73.709 | 5.307 | 1 | **.021** |
| FullRet_Vol_Peri_Superior | -74.830 | 207.2300 | -480.994 | 331.333 | .130 | 1 | .718 |
| FullRet_Vol_Peri_Nasal | 26.505 | 214.9019 | -394.695 | 447.705 | .015 | 1 | .902 |
| FullRet_Vol_Peri_Inferior | -44.171 | 215.6795 | -466.895 | 378.553 | .042 | 1 | .838 |
| FoveaRPE_Elevation_Height | .135 | .0166 | .103 | .168 | 66.913 | 1 | **<.001** |
| RPE_Elev_ParaFovea_Tempo | -.330 | .0246 | -.378 | -.282 | 180.558 | 1 | **.000** |
| RPE_Elev_Para_Superior | -.092 | .0274 | -.146 | -.039 | 11.382 | 1 | **<.001** |
| RPE_Elev_Para_Nasal | .019 | .0200 | -.020 | .058 | .915 | 1 | .339 |
| RPE_Elev_Para_Inferior | .129 | .0182 | .094 | .165 | 50.459 | 1 | <.001 |
| RPE_Elev_PeriFovea_Tempo | .074 | .0168 | .041 | .107 | 19.214 | 1 | <.001 |
| RPE_Elev_Peri_Superior | .140 | .0281 | .085 | .195 | 24.872 | 1 | <.001 |
| RPE_Elev_Peri_Nasal | -.006 | .0211 | -.047 | .036 | .068 | 1 | .794 |
| RPE_Elev_Peri_Inferior | -.101 | .0238 | -.147 | -.054 | 17.931 | 1 | <.001 |
| (Scale) | 15.207^b^ | .6290 | 14.022 | 16.491 |  |  |  |
| Dependent Variable: MMSE  Model: (Intercept), Gender_M2F1, GCC_Inner_Retina_Average, GCC_Superior_Avg, GCC_Inferior_Avg, GCC_S_I_Avg, GCC_FLV, GCC_GLV, GCC_RMS, GCC_Full_Retina_Average, GCC_Superior_Avg.1, GCC_Inferior_Avg.1, GCC_S_I_Avg.1, GCC_Outer_Retina_Average, GCC_Superior_Avg.2, GCC_Inferior_Avg.2, GCC_S_I_Avg.2, ONH_DiscArea, ONH_Area_C_D_ratio, ONH_H_C_D_ratio, ONH_V_C_D_ratio, ONH_CupArea, ONH_RimArea, ONH_RimVolume, ONH_Disc_Volume, ONH_CupVolume, ONH_Avg_RNFL, ONH_Sup_RNFL, ONH_Inf_RNFL, ONH_Tempo, ONH_Superior, ONH_Nasal, ONH_Inferior, ONH_RNFL_TU, ONH_ST, ONH_SN, ONH_NU, ONH_NL, ONH_IN, ONH_IT, ONH_TL, ONH_TU1, ONH_TU2, ONH_ST2, ONH_ST1, ONH_SN1, ONH_SN2, ONH_NU2, ONH_NU1, ONH_NL1, ONH_NL2, ONH_IN2, ONH_IN1, ONH_IT1, ONH_IT2, ONH_TL2, ONH_TL1, Macula_3mm_Thk_ILM_IPL_um_Center_1, Macula_3mm_Thk_ILM_IPL_um_T_1minus3, Macula_3mm_Thk_ILM_IPL_um_S_1minus3, Macula_3mm_Thk_ILM_IPL_um_N_1minus3, Macula_3mm_Thk_ILM_IPL_um_I_1minus3, Macula_3mm_Thk_ILM_IPL_um_S_Hemi_1minus3, Macula_3mm_Thk_ILM_IPL_um_I_Hemi_1minus3, Macula_3mm_Thk_ILM_IPL_um_All_1minus3, Macula_3mm_Thk_ILM_IPL_um_S_Hemi_0minus3, Macula_3mm_Thk_ILM_IPL_um_I_Hemi_0minus3, Macula_3mm_Thk_ILM_IPL_um_All_0minus3, Macula_3mm_Thk_ILM_IPL_um_S_Hemi_field, Macula_3mm_Thk_ILM_IPL_um_I_Hemi_field, Macula_3mm_Thk_ILM_IPL_um_All_field, Macula_3mm_Thk_ILM_RPE_um_Center_1, Macula_3mm_Thk_ILM_RPE_um_T_1minus3, Macula_3mm_Thk_ILM_RPE_um_S_1minus3, Macula_3mm_Thk_ILM_RPE_um_N_1minus3, Macula_3mm_Thk_ILM_RPE_um_I_1minus3, Macula_3mm_Thk_ILM_RPE_um_S_Hemi_1minus3, Macula_3mm_Thk_ILM_RPE_um_I_Hemi_1minus3, Macula_3mm_Thk_ILM_RPE_um_All_1minus3, Macula_3mm_Thk_ILM_RPE_um_S_Hemi_0minus3, Macula_3mm_Thk_ILM_RPE_um_I_Hemi_0minus3, Macula_3mm_Thk_ILM_RPE_um_All_0minus3, Macula_3mm_Thk_ILM_RPE_um_S_Hemi_field, Macula_3mm_Thk_ILM_RPE_um_I_Hemi_field, Macula_3mm_Thk_ILM_RPE_um_All_field, Macula_3mm_Thk_ILM_BRM_um_Center_1, Macula_3mm_Thk_ILM_BRM_um_T_1minus3, Macula_3mm_Thk_ILM_BRM_um_S_1minus3, Macula_3mm_Thk_ILM_BRM_um_N_1minus3, Macula_3mm_Thk_ILM_BRM_um_I_1minus3, Macula_3mm_Thk_ILM_BRM_um_S_Hemi_1minus3, Macula_3mm_Thk_ILM_BRM_um_I_Hemi_1minus3, Macula_3mm_Thk_ILM_BRM_um_All_1minus3, Macula_3mm_Thk_ILM_BRM_um_S_Hemi_0minus3, Macula_3mm_Thk_ILM_BRM_um_I_Hemi_0minus3, Macula_3mm_Thk_ILM_BRM_um_All_0minus3, Macula_3mm_Thk_ILM_BRM_um_S_Hemi_field, Macula_3mm_Thk_ILM_BRM_um_I_Hemi_field, Macula_3mm_Thk_ILM_BRM_um_All_field, Macula_3mm_Thk_RPE_BRM_um_Center_1, Macula_3mm_Thk_RPE_BRM_um_T_1minus3, Macula_3mm_Thk_RPE_BRM_um_S_1minus3, Macula_3mm_Thk_RPE_BRM_um_N_1minus3, Macula_3mm_Thk_RPE_BRM_um_I_1minus3, Macula_3mm_Thk_RPE_BRM_um_S_Hemi_1minus3, Macula_3mm_Thk_RPE_BRM_um_I_Hemi_1minus3, Macula_3mm_Thk_RPE_BRM_um_All_1minus3, Macula_3mm_Thk_RPE_BRM_um_S_Hemi_0minus3, Macula_3mm_Thk_RPE_BRM_um_I_Hemi_0minus3, Macula_3mm_Thk_RPE_BRM_um_All_0minus3, Macula_3mm_Thk_RPE_BRM_um_S_Hemi_field, Macula_3mm_Thk_RPE_BRM_um_I_Hemi_field, Macula_3mm_Thk_RPE_BRM_um_All_field, Macula_3mm_Vol_ILM_IPL_mm3_Center_1, Macula_3mm_Vol_ILM_IPL_mm3_T_1minus3, Macula_3mm_Vol_ILM_IPL_mm3_S_1minus3, Macula_3mm_Vol_ILM_IPL_mm3_N_1minus3, Macula_3mm_Vol_ILM_IPL_mm3_I_1minus3, Macula_3mm_Vol_ILM_IPL_mm3_S_Hemi_1minus3, Macula_3mm_Vol_ILM_IPL_mm3_I_Hemi_1minus3, Macula_3mm_Vol_ILM_IPL_mm3_All_1minus3, Macula_3mm_Vol_ILM_IPL_mm3_S_Hemi_0minus3, Macula_3mm_Vol_ILM_IPL_mm3_I_Hemi_0minus3, Macula_3mm_Vol_ILM_IPL_mm3_All_0minus3, Macula_3mm_Vol_ILM_IPL_mm3_S_Hemi_field, Macula_3mm_Vol_ILM_IPL_mm3_I_Hemi_field, Macula_3mm_Vol_ILM_IPL_mm3_All_field, Macula_3mm_Vol_ILM_RPE_mm3_Center_1, Macula_3mm_Vol_ILM_RPE_mm3_T_1minus3, Macula_3mm_Vol_ILM_RPE_mm3_S_1minus3, Macula_3mm_Vol_ILM_RPE_mm3_N_1minus3, Macula_3mm_Vol_ILM_RPE_mm3_I_1minus3, Macula_3mm_Vol_ILM_RPE_mm3_S_Hemi_1minus3, Macula_3mm_Vol_ILM_RPE_mm3_I_Hemi_1minus3, Macula_3mm_Vol_ILM_RPE_mm3_All_1minus3, Macula_3mm_Vol_ILM_RPE_mm3_S_Hemi_0minus3, Macula_3mm_Vol_ILM_RPE_mm3_I_Hemi_0minus3, Macula_3mm_Vol_ILM_RPE_mm3_All_0minus3, Macula_3mm_Vol_ILM_RPE_mm3_S_Hemi_field, Macula_3mm_Vol_ILM_RPE_mm3_I_Hemi_field, Macula_3mm_Vol_ILM_RPE_mm3_All_field, Macula_3mm_Vol_ILM_BRM_mm3_Center_1, Macula_3mm_Vol_ILM_BRM_mm3_T_1minus3, Macula_3mm_Vol_ILM_BRM_mm3_S_1minus3, Macula_3mm_Vol_ILM_BRM_mm3_N_1minus3, Macula_3mm_Vol_ILM_BRM_mm3_I_1minus3, Macula_3mm_Vol_ILM_BRM_mm3_S_Hemi_1minus3, Macula_3mm_Vol_ILM_BRM_mm3_I_Hemi_1minus3, Macula_3mm_Vol_ILM_BRM_mm3_All_1minus3, Macula_3mm_Vol_ILM_BRM_mm3_S_Hemi_0minus3, Macula_3mm_Vol_ILM_BRM_mm3_I_Hemi_0minus3, Macula_3mm_Vol_ILM_BRM_mm3_All_0minus3, Macula_3mm_Vol_ILM_BRM_mm3_S_Hemi_field, Macula_3mm_Vol_ILM_BRM_mm3_I_Hemi_field, Macula_3mm_Vol_ILM_BRM_mm3_All_field, Macula_3mm_Vol_RPE_BRM_mm3_Center_1, Macula_3mm_Vol_RPE_BRM_mm3_T_1minus3, Macula_3mm_Vol_RPE_BRM_mm3_S_1minus3, Macula_3mm_Vol_RPE_BRM_mm3_N_1minus3, Macula_3mm_Vol_RPE_BRM_mm3_I_1minus3, Macula_3mm_Vol_RPE_BRM_mm3_S_Hemi_1minus3, Macula_3mm_Vol_RPE_BRM_mm3_I_Hemi_1minus3, Macula_3mm_Vol_RPE_BRM_mm3_All_1minus3, Macula_3mm_Vol_RPE_BRM_mm3_S_Hemi_0minus3, Macula_3mm_Vol_RPE_BRM_mm3_I_Hemi_0minus3, Macula_3mm_Vol_RPE_BRM_mm3_All_0minus3, Macula_3mm_Vol_RPE_BRM_mm3_S_Hemi_field, Macula_3mm_Vol_RPE_BRM_mm3_I_Hemi_field, Macula_3mm_Vol_RPE_BRM_mm3_All_field, SVC_L1_DensityOfWhole_Image, SVC_L1_Whole_Image_S_Hemi, SVC_L1_Whole_Image_I_Hemi, SVC_L1_Whole_ETDRS, SVC_L1_Fovea, SVC_L1_ParaFovea, SVC_L1_Para_S_Hemi, SVC_L1_Para_I_Hemi, SVC_L1_Para_T, SVC_L1_Para_S, SVC_L1_Para_N, SVC_L1_Para_I, SVC_L1_G11, SVC_L1_G12, SVC_L1_G13, SVC_L1_G21, SVC_L1_G22, SVC_L1_G23, SVC_L1_G31, SVC_L1_G32, SVC_L1_G33, DVC_L2_DensityOfWhole_Image, DVC_L2_Whole_Image_S_Hemi, DVC_L2_Whole_Image_I_Hemi, DVC_L2_Whole_ETDRS, DVC_L2_Fovea, DVC_L2_ParaFovea, DVC_L2_Para_S_Hemi, DVC_L2_Para_I_Hemi, DVC_L2_Para_T, DVC_L2_Para_S, DVC_L2_Para_N, DVC_L2_Para_I, DVC_L2_G11, DVC_L2_G12, DVC_L2_G13, DVC_L2_G21, DVC_L2_G22, DVC_L2_G23, DVC_L2_G31, DVC_L2_G32, DVC_L2_G33, FAZ_Area, Perimeter, AcircularityIndex, FD_300_Area_Density, FD_300_Length_Density, FoveaInnRet_Thickness, InnRet_Thk_ParaFovea, InnRet_Thk_Para_S_Hemisphere, InnRet_Thk_Para_I_Hemisphere, InnRet_Thk_Para_Tempo, InnRet_Thk_Para_Superior, InnRet_Thk_Para_Nasal, InnRet_Thk_Para_Inferior, InnRet_Thk_PeriFovea, InnRet_Thk_Peri_S_Hemisphere, InnRet_Thk_Peri_I_Hemisphere, InnRet_Thk_Peri_Tempo, InnRet_Thk_Peri_Superior, InnRet_Thk_Peri_Nasal, InnRet_Thk_Peri_Inferior, FoveaInnRet_Volumn, InnRet_Vol_ParaFovea, InnRet_Vol_Para_S_Hemisphere, InnRet_Vol_Para_I_Hemisphere, InnRet_Vol_Para_Tempo, InnRet_Vol_Para_Superior, InnRet_Vol_Para_Nasal, InnRet_Vol_Para_Inferior, InnRet_Vol_PeriFovea, InnRet_Vol_Peri_S_Hemisphere, InnRet_Vol_Peri_I_Hemisphere, InnRet_Vol_Peri_Tempo, InnRet_Vol_Peri_Superior, InnRet_Vol_Peri_Nasal, InnRet_Vol_Peri_Inferior, FoveaFullRet_Thickness, FullRet_Thk_ParaFovea, FullRet_Thk_Para_S_Hemisphere, FullRet_Thk_Para_I_Hemisphere, FullRet_Thk_Para_Tempo, FullRet_Thk_Para_Superior, FullRet_Thk_Para_Nasal, FullRet_Thk_Para_Inferior, FullRet_Thk_PeriFovea, FullRet_Thk_Peri_S_Hemisphere, FullRet_Thk_Peri_I_Hemisphere, FullRet_Thk_Peri_Tempo, FullRet_Thk_Peri_Superior, FullRet_Thk_Peri_Nasal, FullRet_Thk_Peri_Inferior, FoveaFullRet_Volumn, FullRet_Vol_ParaFovea, FullRet_Vol_Para_S_Hemisphere, FullRet_Vol_Para_I_Hemisphere, FullRet_Vol_Para_Tempo, FullRet_Vol_Para_Superior, FullRet_Vol_Para_Nasal, FullRet_Vol_Para_Inferior, FullRet_Vol_PeriFovea, FullRet_Vol_Peri_S_Hemisphere, FullRet_Vol_Peri_I_Hemisphere, FullRet_Vol_Peri_Tempo, FullRet_Vol_Peri_Superior, FullRet_Vol_Peri_Nasal, FullRet_Vol_Peri_Inferior, FoveaRPE_Elevation_Height, RPE_Elev_ParaFovea_Tempo, RPE_Elev_Para_Superior, RPE_Elev_Para_Nasal, RPE_Elev_Para_Inferior, RPE_Elev_PeriFovea_Tempo, RPE_Elev_Peri_Superior, RPE_Elev_Peri_Nasal, RPE_Elev_Peri_Inferior, offset = AgePerToday  a. Set to zero because this parameter is redundant.  b. Maximum likelihood estimate. | | | | | | | |
|  | | | | | | | |

# Generalized Linear Model (Target Variable: Classes) Results

The statistically significant parameters have their p-value in **Bold** font. We present the results of generalized linear model (GLM) analysis performed by SPSS software with Class_Num as a dependent variable. The Class_Num is the classes variable converted into numeric equivalent such that HCs, MCI, and Dem were denoted by 0, 1, 2 respectively.

When developing GLM model on SPSS, we had to split retina map parameters into 3 groups namely: inner retina thickness, inner retina volume, full retina thickness/volume and RPE. Without this splitting mechanism, the GLM model was not able to converge.

Starting with GLM model information followed by statistical details of variables under “Categorical Variable Information” and “Continuous Variable Information”. In contrast to previous GLM, we included Goodness of fit and Omnibus test as a reference. Moreover, statistical p-value significance shown under “Tests of Model Effects”. Finally, 95% Confidence Interval as well as statistical Hypothesis Test of Chi-Square is presented under “Parameter Estimates”.

## Model Information

| Dependent Variable | Class_Num^a^ |
| --- | --- |
| Probability Distribution | Multinomial |
| Link Function | Cumulative logit |
| Offset Variable | AgePerToday |

## Categorical Variable Information

| **Categorical Variable Information** | | | | |
| --- | --- | --- | --- | --- |
|  | | | N | Percent |
| Dependent Variable | Class_Num | 0 | 174 | 48.7% |
|  |  | 1 | 139 | 38.9% |
|  |  | 2 | 44 | 12.3% |
|  |  | Total | 357 | 100.0% |
| Factor | Gender_M2F1 | 1 | 206 | 57.7% |
|  |  | 2 | 151 | 42.3% |
|  |  | Total | 357 | 100.0% |
|  | AMD | 0 | 269 | 75.4% |
|  |  | 1 | 88 | 24.6% |
|  |  | Total | 357 | 100.0% |
|  | MACULOPATHY | 0 | 305 | 85.4% |
|  |  | 1 | 52 | 14.6% |
|  |  | Total | 357 | 100.0% |
|  | DR | 0 | 351 | 98.3% |
|  |  | 1 | 6 | 1.7% |
|  |  | Total | 357 | 100.0% |
|  | GLAUCOMA | 0 | 336 | 94.1% |
|  |  | 1 | 21 | 5.9% |
|  |  | Total | 357 | 100.0% |

Note: Gender_M2F1 is a given numerical value of 1 for female and 2 for male participants

## Continuous Variable Information

| **Continuous Variable Information** | | | | | | |
| --- | --- | --- | --- | --- | --- | --- |
|  | | N | Minimum | Maximum | Mean | Std. Deviation |
| Covariate | GCC_Inner_Retina_Average | 357 | 60.95 | 146.16 | 96.2255 | 10.49492 |
|  | GCC_Superior_Avg | 357 | 58.51 | 152.26 | 95.7850 | 10.83188 |
|  | GCC_Inferior_Avg | 357 | 62.00 | 140.04 | 96.6758 | 10.78998 |
|  | GCC_S_I_Avg | 357 | -18.29 | 19.40 | -.8755 | 4.98499 |
|  | GCC_FLV | 357 | .000 | 21.964 | 1.40788 | 2.310063 |
|  | GCC_GLV | 357 | .000 | 33.443 | 4.20881 | 5.238621 |
|  | GCC_RMS | 357 | .03 | 1.03 | .0912 | .07971 |
|  | GCC_Full_Retina_Average | 357 | 221.52 | 354.47 | 275.3752 | 15.96806 |
|  | GCC_Superior_Avg.1 | 357 | 42.77 | 286.26 | 95.9495 | 22.59594 |
|  | GCC_Inferior_Avg.1 | 357 | 46.43 | 287.46 | 99.9005 | 22.52670 |
|  | GCC_S_I_Avg.1 | 357 | -38.85 | 50.38 | -.4122 | 8.12804 |
|  | GCC_Outer_Retina_Average | 357 | 140.91 | 222.15 | 179.1355 | 9.43361 |
|  | GCC_Superior_Avg.2 | 357 | 42.77 | 185.88 | 97.3454 | 15.04068 |
|  | GCC_Inferior_Avg.2 | 357 | 62.00 | 189.77 | 100.4287 | 19.03752 |
|  | GCC_S_I_Avg.2 | 357 | -43.23 | 19.40 | -1.4717 | 6.03400 |
|  | ONH_DiscArea | 357 | .00 | 3.71 | 2.0090 | .46306 |
|  | ONH_Area_C_D_ratio | 357 | .00 | .86 | .2949 | .17401 |
|  | ONH_H_C_D_ratio | 357 | .00 | 1.00 | .5431 | .23057 |
|  | ONH_V_C_D_ratio | 357 | .00 | .99 | .4733 | .21529 |
|  | ONH_CupArea | 357 | .00 | 2.01 | .6132 | .40635 |
|  | ONH_RimArea | 357 | .00 | 2.88 | 1.3751 | .40772 |
|  | ONH_RimVolume | 357 | .000 | .490 | .14857 | .077161 |
|  | ONH_Disc_Volume | 357 | .000 | 1.168 | .32482 | .167875 |
|  | ONH_CupVolume | 357 | .000 | .842 | .10373 | .134492 |
|  | ONH_Avg_RNFL | 357 | 56.21 | 123.78 | 96.8978 | 10.70932 |
|  | ONH_Sup_RNFL | 357 | 60.53 | 125.76 | 98.1720 | 11.32904 |
|  | ONH_Inf_RNFL | 357 | 51.90 | 123.85 | 95.8780 | 11.14183 |
|  | ONH_Tempo | 357 | 35.39 | 111.94 | 72.7199 | 10.40557 |
|  | ONH_Superior | 357 | 70.31 | 175.84 | 117.2317 | 15.07685 |
|  | ONH_Nasal | 357 | 43.23 | 135.92 | 76.0089 | 11.94326 |
|  | ONH_Inferior | 357 | 57.92 | 160.08 | 121.7851 | 15.80073 |
|  | ONH_RNFL_TU | 357 | 43.048 | 149.563 | 79.23490 | 14.054446 |
|  | ONH_ST | 357 | 69.019 | 185.052 | 128.81629 | 17.458863 |
|  | ONH_SN | 357 | 55.840 | 149.790 | 105.65856 | 16.778563 |
|  | ONH_NU | 357 | 39.178 | 162.380 | 79.27634 | 13.820719 |
|  | ONH_NL | 357 | 44.732 | 137.261 | 73.01126 | 11.909073 |
|  | ONH_IN | 357 | 60.908 | 176.878 | 111.93620 | 20.455214 |
|  | ONH_IT | 357 | 52.183 | 177.365 | 131.77751 | 19.169946 |
|  | ONH_TL | 357 | 27.422 | 109.577 | 66.21564 | 10.903166 |
|  | ONH_TU1 | 357 | 31.194 | 104.474 | 65.93152 | 11.096264 |
|  | ONH_TU2 | 357 | 40.852 | 145.762 | 91.66192 | 17.210556 |
|  | ONH_ST2 | 357 | 57.749 | 189.491 | 126.36843 | 22.136315 |
|  | ONH_ST1 | 357 | 69.548 | 189.810 | 130.62458 | 21.116520 |
|  | ONH_SN1 | 357 | 46.075 | 190.281 | 108.07087 | 20.139711 |
|  | ONH_SN2 | 357 | 51.923 | 157.848 | 103.29061 | 16.624245 |
|  | ONH_NU2 | 357 | 41.384 | 195.892 | 87.88801 | 16.668098 |
|  | ONH_NU1 | 357 | 37.010 | 145.328 | 70.17748 | 13.164672 |
|  | ONH_NL1 | 357 | 41.086 | 135.105 | 66.34294 | 11.576895 |
|  | ONH_NL2 | 357 | 40.079 | 139.379 | 79.65962 | 13.915362 |
|  | ONH_IN2 | 357 | 58.659 | 175.982 | 100.68952 | 19.052836 |
|  | ONH_IN1 | 357 | 62.728 | 196.946 | 122.59321 | 23.703810 |
|  | ONH_IT1 | 357 | 50.105 | 193.581 | 142.72901 | 21.030749 |
|  | ONH_IT2 | 357 | 54.224 | 181.306 | 121.19402 | 23.860960 |
|  | ONH_TL2 | 357 | 38.382 | 126.133 | 74.77401 | 13.949765 |
|  | ONH_TL1 | 357 | 16.651 | 115.621 | 57.57140 | 9.932362 |
|  | Macula_3mm_Thk_ILM_IPL_um_Center_1 | 357 | 25.8 | 96.4 | 47.634 | 9.8009 |
|  | Macula_3mm_Thk_ILM_IPL_um_T_1minus3 | 357 | 54.0 | 186.2 | 101.939 | 11.8283 |
|  | Macula_3mm_Thk_ILM_IPL_um_S_1minus3 | 357 | 57.9 | 176.9 | 112.063 | 13.6703 |
|  | Macula_3mm_Thk_ILM_IPL_um_N_1minus3 | 357 | 57.7 | 169.2 | 107.561 | 12.8421 |
|  | Macula_3mm_Thk_ILM_IPL_um_I_1minus3 | 357 | 63.4 | 167.1 | 112.271 | 12.6806 |
|  | Macula_3mm_Thk_ILM_IPL_um_S_Hemi_1minus3 | 357 | 55.7 | 177.8 | 108.114 | 12.7947 |
|  | Macula_3mm_Thk_ILM_IPL_um_I_Hemi_1minus3 | 357 | 60.8 | 172.0 | 108.792 | 12.2593 |
|  | Macula_3mm_Thk_ILM_IPL_um_All_1minus3 | 357 | 58.3 | 174.8 | 108.457 | 12.2699 |
|  | Macula_3mm_Thk_ILM_IPL_um_S_Hemi_0minus3 | 357 | 52.4 | 168.1 | 101.294 | 12.0679 |
|  | Macula_3mm_Thk_ILM_IPL_um_I_Hemi_0minus3 | 357 | 56.9 | 163.5 | 101.919 | 11.5704 |
|  | Macula_3mm_Thk_ILM_IPL_um_All_0minus3 | 357 | 54.6 | 165.7 | 101.610 | 11.6042 |
|  | Macula_3mm_Thk_ILM_IPL_um_S_Hemi_field | 357 | 54.5 | 169.7 | 102.630 | 12.0247 |
|  | Macula_3mm_Thk_ILM_IPL_um_I_Hemi_field | 357 | 59.0 | 163.4 | 103.642 | 11.5618 |
|  | Macula_3mm_Thk_ILM_IPL_um_All_field | 357 | 56.8 | 166.2 | 103.256 | 11.5368 |
|  | Macula_3mm_Thk_ILM_RPE_um_Center_1 | 357 | 178.5 | 457.9 | 259.224 | 26.9095 |
|  | Macula_3mm_Thk_ILM_RPE_um_T_1minus3 | 357 | 218.8 | 451.3 | 316.494 | 20.2799 |
|  | Macula_3mm_Thk_ILM_RPE_um_S_1minus3 | 357 | 217.1 | 440.4 | 327.287 | 21.3049 |
|  | Macula_3mm_Thk_ILM_RPE_um_N_1minus3 | 357 | 237.2 | 427.9 | 328.104 | 21.3205 |
|  | Macula_3mm_Thk_ILM_RPE_um_I_1minus3 | 357 | 236.3 | 430.0 | 325.292 | 20.6836 |
|  | Macula_3mm_Thk_ILM_RPE_um_S_Hemi_1minus3 | 357 | 218.5 | 439.9 | 324.572 | 20.7108 |
|  | Macula_3mm_Thk_ILM_RPE_um_I_Hemi_1minus3 | 357 | 236.3 | 434.9 | 323.998 | 20.3391 |
|  | Macula_3mm_Thk_ILM_RPE_um_All_1minus3 | 357 | 227.7 | 437.3 | 324.287 | 20.2395 |
|  | Macula_3mm_Thk_ILM_RPE_um_S_Hemi_0minus3 | 357 | 217.8 | 442.1 | 317.207 | 20.5800 |
|  | Macula_3mm_Thk_ILM_RPE_um_I_Hemi_0minus3 | 357 | 233.6 | 437.5 | 316.720 | 20.2659 |
|  | Macula_3mm_Thk_ILM_RPE_um_All_0minus3 | 357 | 226.0 | 439.7 | 316.964 | 20.1947 |
|  | Macula_3mm_Thk_ILM_RPE_um_S_Hemi_field | 357 | 216.6 | 437.4 | 315.201 | 20.0818 |
|  | Macula_3mm_Thk_ILM_RPE_um_I_Hemi_field | 357 | 226.5 | 418.5 | 313.908 | 19.5279 |
|  | Macula_3mm_Thk_ILM_RPE_um_All_field | 357 | 222.2 | 426.4 | 314.615 | 19.5048 |
|  | Macula_3mm_Thk_ILM_BRM_um_Center_1 | 357 | 192.2 | 464.9 | 266.442 | 26.9263 |
|  | Macula_3mm_Thk_ILM_BRM_um_T_1minus3 | 357 | 246.4 | 454.3 | 321.393 | 20.1114 |
|  | Macula_3mm_Thk_ILM_BRM_um_S_1minus3 | 357 | 224.4 | 444.6 | 332.562 | 21.1630 |
|  | Macula_3mm_Thk_ILM_BRM_um_N_1minus3 | 357 | 259.8 | 433.5 | 333.946 | 21.1089 |
|  | Macula_3mm_Thk_ILM_BRM_um_I_1minus3 | 357 | 252.9 | 434.4 | 330.453 | 19.9856 |
|  | Macula_3mm_Thk_ILM_BRM_um_S_Hemi_1minus3 | 357 | 228.8 | 443.9 | 329.887 | 20.5615 |
|  | Macula_3mm_Thk_ILM_BRM_um_I_Hemi_1minus3 | 357 | 262.7 | 439.5 | 329.265 | 19.8597 |
|  | Macula_3mm_Thk_ILM_BRM_um_All_1minus3 | 357 | 257.6 | 441.6 | 329.585 | 19.8293 |
|  | Macula_3mm_Thk_ILM_BRM_um_S_Hemi_0minus3 | 357 | 228.9 | 446.4 | 322.739 | 20.4250 |
|  | Macula_3mm_Thk_ILM_BRM_um_I_Hemi_0minus3 | 357 | 256.0 | 442.3 | 322.214 | 19.8186 |
|  | Macula_3mm_Thk_ILM_BRM_um_All_0minus3 | 357 | 251.5 | 444.3 | 322.478 | 19.8225 |
|  | Macula_3mm_Thk_ILM_BRM_um_S_Hemi_field | 357 | 226.8 | 441.6 | 320.569 | 19.8839 |
|  | Macula_3mm_Thk_ILM_BRM_um_I_Hemi_field | 357 | 256.7 | 422.6 | 319.218 | 18.9638 |
|  | Macula_3mm_Thk_ILM_BRM_um_All_field | 357 | 252.0 | 430.6 | 319.959 | 19.0059 |
|  | Macula_3mm_Thk_RPE_BRM_um_Center_1 | 357 | 1.5 | 42.2 | 7.215 | 2.9606 |
|  | Macula_3mm_Thk_RPE_BRM_um_T_1minus3 | 357 | .8 | 29.9 | 4.896 | 2.2897 |
|  | Macula_3mm_Thk_RPE_BRM_um_S_1minus3 | 357 | .7 | 17.4 | 5.272 | 2.1888 |
|  | Macula_3mm_Thk_RPE_BRM_um_N_1minus3 | 357 | .4 | 25.6 | 5.841 | 2.8539 |
|  | Macula_3mm_Thk_RPE_BRM_um_I_1minus3 | 357 | .6 | 88.5 | 5.156 | 5.3095 |
|  | Macula_3mm_Thk_RPE_BRM_um_S_Hemi_1minus3 | 357 | .7 | 16.1 | 5.317 | 2.1271 |
|  | Macula_3mm_Thk_RPE_BRM_um_I_Hemi_1minus3 | 357 | .8 | 64.7 | 5.269 | 4.0405 |
|  | Macula_3mm_Thk_RPE_BRM_um_All_1minus3 | 357 | .8 | 38.5 | 5.296 | 2.7765 |
|  | Macula_3mm_Thk_RPE_BRM_um_S_Hemi_0minus3 | 357 | .8 | 15.8 | 5.527 | 2.1156 |
|  | Macula_3mm_Thk_RPE_BRM_um_I_Hemi_0minus3 | 357 | 1.0 | 59.4 | 5.490 | 3.7728 |
|  | Macula_3mm_Thk_RPE_BRM_um_All_0minus3 | 357 | .9 | 36.0 | 5.512 | 2.6861 |
|  | Macula_3mm_Thk_RPE_BRM_um_S_Hemi_field | 357 | .8 | 17.8 | 5.368 | 2.0726 |
|  | Macula_3mm_Thk_RPE_BRM_um_I_Hemi_field | 357 | .9 | 68.5 | 5.309 | 4.1113 |
|  | Macula_3mm_Thk_RPE_BRM_um_All_field | 357 | 1.0 | 43.6 | 5.346 | 2.9003 |
|  | Macula_3mm_Vol_ILM_IPL_mm3_Center_1 | 357 | .020 | .076 | .03742 | .007730 |
|  | Macula_3mm_Vol_ILM_IPL_mm3_T_1minus3 | 357 | .085 | .293 | .15859 | .018931 |
|  | Macula_3mm_Vol_ILM_IPL_mm3_S_1minus3 | 357 | .090 | .264 | .17213 | .021542 |
|  | Macula_3mm_Vol_ILM_IPL_mm3_N_1minus3 | 357 | .090 | .264 | .16603 | .020136 |
|  | Macula_3mm_Vol_ILM_IPL_mm3_I_1minus3 | 357 | .099 | .262 | .17513 | .020057 |
|  | Macula_3mm_Vol_ILM_IPL_mm3_S_Hemi_1minus3 | 357 | .174 | .513 | .33413 | .039497 |
|  | Macula_3mm_Vol_ILM_IPL_mm3_I_Hemi_1minus3 | 357 | .191 | .539 | .33783 | .038234 |
|  | Macula_3mm_Vol_ILM_IPL_mm3_All_1minus3 | 357 | .365 | 1.053 | .67191 | .075754 |
|  | Macula_3mm_Vol_ILM_IPL_mm3_S_Hemi_0minus3 | 357 | .185 | .551 | .35282 | .041928 |
|  | Macula_3mm_Vol_ILM_IPL_mm3_I_Hemi_0minus3 | 357 | .201 | .577 | .35650 | .040627 |
|  | Macula_3mm_Vol_ILM_IPL_mm3_All_0minus3 | 357 | .386 | 1.128 | .70935 | .080656 |
|  | Macula_3mm_Vol_ILM_IPL_mm3_S_Hemi_field | 357 | .242 | .708 | .45396 | .058266 |
|  | Macula_3mm_Vol_ILM_IPL_mm3_I_Hemi_field | 357 | .271 | .851 | .47760 | .059126 |
|  | Macula_3mm_Vol_ILM_IPL_mm3_All_field | 357 | .513 | 1.500 | .93162 | .104092 |
|  | Macula_3mm_Vol_ILM_RPE_mm3_Center_1 | 357 | .140 | .360 | .20369 | .021144 |
|  | Macula_3mm_Vol_ILM_RPE_mm3_T_1minus3 | 357 | .345 | .710 | .49234 | .033947 |
|  | Macula_3mm_Vol_ILM_RPE_mm3_S_1minus3 | 357 | .293 | .645 | .50281 | .035653 |
|  | Macula_3mm_Vol_ILM_RPE_mm3_N_1minus3 | 357 | .363 | .669 | .50659 | .035531 |
|  | Macula_3mm_Vol_ILM_RPE_mm3_I_1minus3 | 357 | .371 | .675 | .50742 | .033629 |
|  | Macula_3mm_Vol_ILM_RPE_mm3_S_Hemi_1minus3 | 357 | .638 | 1.270 | 1.00309 | .064913 |
|  | Macula_3mm_Vol_ILM_RPE_mm3_I_Hemi_1minus3 | 357 | .742 | 1.365 | 1.00603 | .064251 |
|  | Macula_3mm_Vol_ILM_RPE_mm3_All_1minus3 | 357 | 1.379 | 2.634 | 2.00911 | .125326 |
|  | Macula_3mm_Vol_ILM_RPE_mm3_S_Hemi_0minus3 | 357 | .721 | 1.450 | 1.10494 | .072057 |
|  | Macula_3mm_Vol_ILM_RPE_mm3_I_Hemi_0minus3 | 357 | .825 | 1.544 | 1.10791 | .071825 |
|  | Macula_3mm_Vol_ILM_RPE_mm3_All_0minus3 | 357 | 1.546 | 2.994 | 2.21287 | .140448 |
|  | Macula_3mm_Vol_ILM_RPE_mm3_S_Hemi_field | 357 | .840 | 1.805 | 1.39368 | .110084 |
|  | Macula_3mm_Vol_ILM_RPE_mm3_I_Hemi_field | 357 | 1.094 | 2.181 | 1.44647 | .115299 |
|  | Macula_3mm_Vol_ILM_RPE_mm3_All_field | 357 | 2.007 | 3.851 | 2.84016 | .176122 |
|  | Macula_3mm_Vol_ILM_BRM_mm3_Center_1 | 357 | .151 | .365 | .20935 | .021157 |
|  | Macula_3mm_Vol_ILM_BRM_mm3_T_1minus3 | 357 | .388 | .715 | .49994 | .033827 |
|  | Macula_3mm_Vol_ILM_BRM_mm3_S_1minus3 | 357 | .303 | .655 | .51093 | .035528 |
|  | Macula_3mm_Vol_ILM_BRM_mm3_N_1minus3 | 357 | .378 | .677 | .51560 | .035352 |
|  | Macula_3mm_Vol_ILM_BRM_mm3_I_1minus3 | 357 | .377 | .682 | .51546 | .032742 |
|  | Macula_3mm_Vol_ILM_BRM_mm3_S_Hemi_1minus3 | 357 | .668 | 1.281 | 1.01950 | .064494 |
|  | Macula_3mm_Vol_ILM_BRM_mm3_I_Hemi_1minus3 | 357 | .805 | 1.379 | 1.02241 | .062958 |
|  | Macula_3mm_Vol_ILM_BRM_mm3_All_1minus3 | 357 | 1.584 | 2.660 | 2.04196 | .122790 |
|  | Macula_3mm_Vol_ILM_BRM_mm3_S_Hemi_0minus3 | 357 | .758 | 1.464 | 1.12420 | .071559 |
|  | Macula_3mm_Vol_ILM_BRM_mm3_I_Hemi_0minus3 | 357 | .886 | 1.562 | 1.12713 | .070462 |
|  | Macula_3mm_Vol_ILM_BRM_mm3_All_0minus3 | 357 | 1.744 | 3.025 | 2.25134 | .137849 |
|  | Macula_3mm_Vol_ILM_BRM_mm3_S_Hemi_field | 357 | .880 | 1.837 | 1.41743 | .109947 |
|  | Macula_3mm_Vol_ILM_BRM_mm3_I_Hemi_field | 357 | 1.099 | 2.202 | 1.47103 | .115059 |
|  | Macula_3mm_Vol_ILM_BRM_mm3_All_field | 357 | 2.275 | 3.888 | 2.88847 | .171594 |
|  | Macula_3mm_Vol_RPE_BRM_mm3_Center_1 | 357 | .001 | .033 | .00567 | .002334 |
|  | Macula_3mm_Vol_RPE_BRM_mm3_T_1minus3 | 357 | .001 | .047 | .00763 | .003594 |
|  | Macula_3mm_Vol_RPE_BRM_mm3_S_1minus3 | 357 | .001 | .027 | .00811 | .003390 |
|  | Macula_3mm_Vol_RPE_BRM_mm3_N_1minus3 | 357 | .001 | .040 | .00901 | .004424 |
|  | Macula_3mm_Vol_RPE_BRM_mm3_I_1minus3 | 357 | .001 | .139 | .00803 | .008343 |
|  | Macula_3mm_Vol_RPE_BRM_mm3_S_Hemi_1minus3 | 357 | .002 | .050 | .01641 | .006561 |
|  | Macula_3mm_Vol_RPE_BRM_mm3_I_Hemi_1minus3 | 357 | .002 | .203 | .01638 | .012663 |
|  | Macula_3mm_Vol_RPE_BRM_mm3_All_1minus3 | 357 | .005 | .233 | .03283 | .017062 |
|  | Macula_3mm_Vol_RPE_BRM_mm3_S_Hemi_0minus3 | 357 | .003 | .056 | .01923 | .007379 |
|  | Macula_3mm_Vol_RPE_BRM_mm3_I_Hemi_0minus3 | 357 | .003 | .210 | .01922 | .013302 |
|  | Macula_3mm_Vol_RPE_BRM_mm3_All_0minus3 | 357 | .006 | .247 | .03844 | .018627 |
|  | Macula_3mm_Vol_RPE_BRM_mm3_S_Hemi_field | 357 | .004 | .081 | .02371 | .009209 |
|  | Macula_3mm_Vol_RPE_BRM_mm3_I_Hemi_field | 357 | .004 | .353 | .02457 | .020655 |
|  | Macula_3mm_Vol_RPE_BRM_mm3_All_field | 357 | .009 | .393 | .04828 | .026151 |
|  | SVC_L1_DensityOfWhole_Image | 357 | 26.09 | 56.89 | 45.9209 | 4.08692 |
|  | SVC_L1_Whole_Image_S_Hemi | 357 | 24.22 | 56.93 | 45.8949 | 4.14826 |
|  | SVC_L1_Whole_Image_I_Hemi | 357 | 27.75 | 56.86 | 45.9391 | 4.22127 |
|  | SVC_L1_Whole_ETDRS | 357 | 25.73 | 56.26 | 45.5407 | 4.04893 |
|  | SVC_L1_Fovea | 357 | 1.59 | 38.29 | 20.4209 | 6.63652 |
|  | SVC_L1_ParaFovea | 357 | 27.27 | 59.94 | 48.7259 | 4.12725 |
|  | SVC_L1_Para_S_Hemi | 357 | 25.74 | 60.88 | 48.6827 | 4.25150 |
|  | SVC_L1_Para_I_Hemi | 357 | 28.77 | 59.03 | 48.7637 | 4.26060 |
|  | SVC_L1_Para_T | 357 | 25.74 | 58.86 | 47.6749 | 4.38582 |
|  | SVC_L1_Para_S | 357 | 26.17 | 61.83 | 49.7005 | 4.51418 |
|  | SVC_L1_Para_N | 357 | 23.84 | 60.33 | 47.9908 | 4.33799 |
|  | SVC_L1_Para_I | 357 | 28.26 | 58.90 | 49.5370 | 4.68367 |
|  | SVC_L1_G11 | 357 | 21.07 | 58.41 | 47.9367 | 4.99859 |
|  | SVC_L1_G12 | 357 | 28.94 | 61.20 | 49.8978 | 4.52302 |
|  | SVC_L1_G13 | 357 | 23.30 | 61.41 | 48.2373 | 4.92422 |
|  | SVC_L1_G21 | 357 | 30.91 | 56.75 | 47.4022 | 4.43751 |
|  | SVC_L1_G22 | 357 | 7.87 | 42.47 | 25.8675 | 6.00944 |
|  | SVC_L1_G23 | 357 | 22.27 | 59.76 | 47.6106 | 4.69147 |
|  | SVC_L1_G31 | 357 | 28.20 | 59.78 | 47.9753 | 5.08977 |
|  | SVC_L1_G32 | 357 | 26.64 | 60.64 | 49.8907 | 4.89485 |
|  | SVC_L1_G33 | 357 | 27.50 | 60.28 | 48.2708 | 5.27379 |
|  | DVC_L2_DensityOfWhole_Image | 357 | 31.11 | 58.31 | 48.1327 | 4.32465 |
|  | DVC_L2_Whole_Image_S_Hemi | 357 | 31.19 | 58.33 | 48.3377 | 4.33301 |
|  | DVC_L2_Whole_Image_I_Hemi | 357 | 31.06 | 58.33 | 47.9315 | 4.51435 |
|  | DVC_L2_Whole_ETDRS | 357 | 31.39 | 58.50 | 48.0211 | 4.09753 |
|  | DVC_L2_Fovea | 357 | 14.15 | 50.38 | 31.2408 | 6.82786 |
|  | DVC_L2_ParaFovea | 357 | 31.78 | 61.01 | 50.1471 | 4.41429 |
|  | DVC_L2_Para_S_Hemi | 357 | 30.59 | 60.53 | 50.3076 | 4.40136 |
|  | DVC_L2_Para_I_Hemi | 357 | 32.88 | 61.47 | 49.9755 | 4.65574 |
|  | DVC_L2_Para_T | 357 | 35.68 | 60.51 | 50.4267 | 4.33539 |
|  | DVC_L2_Para_S | 357 | 29.43 | 60.12 | 50.3741 | 4.82209 |
|  | DVC_L2_Para_N | 357 | 32.23 | 64.03 | 50.2189 | 4.49745 |
|  | DVC_L2_Para_I | 357 | 29.41 | 61.34 | 49.5701 | 5.27418 |
|  | DVC_L2_G11 | 357 | 32.91 | 62.22 | 49.3466 | 5.59273 |
|  | DVC_L2_G12 | 357 | 32.49 | 60.33 | 50.6051 | 4.84606 |
|  | DVC_L2_G13 | 357 | 27.98 | 61.62 | 49.3161 | 5.57598 |
|  | DVC_L2_G21 | 357 | 35.37 | 60.33 | 50.5482 | 4.20688 |
|  | DVC_L2_G22 | 357 | 20.27 | 50.80 | 35.6961 | 5.61015 |
|  | DVC_L2_G23 | 357 | 34.50 | 63.27 | 50.7283 | 4.47819 |
|  | DVC_L2_G31 | 357 | 28.06 | 60.92 | 48.6557 | 5.82662 |
|  | DVC_L2_G32 | 357 | 28.27 | 62.92 | 49.8176 | 5.62894 |
|  | DVC_L2_G33 | 357 | 26.19 | 62.26 | 48.4176 | 5.87083 |
|  | FAZ_Area | 357 | .016 | .666 | .30303 | .117516 |
|  | Perimeter | 357 | .483 | 3.581 | 2.20923 | .466242 |
|  | AcircularityIndex | 357 | 1.06 | 1.42 | 1.1552 | .05350 |
|  | FD_300_Area_Density | 357 | 23.73 | 59.67 | 48.6737 | 4.46573 |
|  | FD_300_Length_Density | 357 | 6.15 | 19.54 | 15.9871 | 2.24722 |
|  | FoveaInnRet_Thickness | 357 | 45.67 | 143.34 | 73.2079 | 13.93155 |
|  | InnRet_Thk_ParaFovea | 357 | 83 | 160 | 126.35 | 11.695 |
|  | InnRet_Thk_Para_S_Hemisphere | 357 | 78 | 171 | 126.40 | 12.438 |
|  | InnRet_Thk_Para_I_Hemisphere | 357 | 85 | 161 | 126.33 | 11.920 |
|  | InnRet_Thk_Para_Tempo | 357 | 52 | 164 | 119.50 | 11.699 |
|  | InnRet_Thk_Para_Superior | 357 | 75 | 170 | 128.68 | 13.256 |
|  | InnRet_Thk_Para_Nasal | 357 | 84 | 174 | 129.32 | 13.897 |
|  | InnRet_Thk_Para_Inferior | 357 | 82 | 164 | 127.96 | 12.630 |
|  | InnRet_Thk_PeriFovea | 357 | 71 | 151 | 109.97 | 9.713 |
|  | InnRet_Thk_Peri_S_Hemisphere | 357 | 70 | 155 | 110.51 | 9.845 |
|  | InnRet_Thk_Peri_I_Hemisphere | 357 | 63 | 167 | 109.48 | 10.871 |
|  | InnRet_Thk_Peri_Tempo | 357 | 42 | 192 | 104.27 | 11.097 |
|  | InnRet_Thk_Peri_Superior | 357 | 63 | 150 | 109.45 | 10.212 |
|  | InnRet_Thk_Peri_Nasal | 357 | 73 | 158 | 119.08 | 11.020 |
|  | InnRet_Thk_Peri_Inferior | 357 | 50 | 285 | 107.40 | 14.759 |
|  | FoveaInnRet_Volumn | 357 | .030 | .113 | .05732 | .011287 |
|  | InnRet_Vol_ParaFovea | 357 | .443 | 1.009 | .79368 | .076663 |
|  | InnRet_Vol_Para_S_Hemisphere | 357 | .227 | .538 | .39650 | .040557 |
|  | InnRet_Vol_Para_I_Hemisphere | 357 | .183 | .505 | .39693 | .039910 |
|  | InnRet_Vol_Para_Tempo | 357 | .081 | .257 | .18782 | .020793 |
|  | InnRet_Vol_Para_Superior | 357 | .118 | .269 | .20165 | .021897 |
|  | InnRet_Vol_Para_Nasal | 357 | .079 | .273 | .20303 | .023804 |
|  | InnRet_Vol_Para_Inferior | 357 | .110 | .257 | .20054 | .020948 |
|  | InnRet_Vol_PeriFovea | 357 | .784 | 1.899 | 1.37761 | .132514 |
|  | InnRet_Vol_Peri_S_Hemisphere | 357 | .414 | .971 | .69284 | .068045 |
|  | InnRet_Vol_Peri_I_Hemisphere | 357 | .324 | 1.048 | .68468 | .072993 |
|  | InnRet_Vol_Peri_Tempo | 357 | .133 | .602 | .32700 | .041553 |
|  | InnRet_Vol_Peri_Superior | 357 | .128 | .471 | .34312 | .037034 |
|  | InnRet_Vol_Peri_Nasal | 357 | .177 | .495 | .37348 | .039010 |
|  | InnRet_Vol_Peri_Inferior | 357 | .126 | .531 | .33382 | .040504 |
|  | FoveaFullRet_Thickness | 357 | 193.88 | 459.26 | 260.5494 | 26.56362 |
|  | FullRet_Thk_ParaFovea | 357 | 232 | 428 | 317.92 | 19.974 |
|  | FullRet_Thk_Para_S_Hemisphere | 357 | 219 | 433 | 317.75 | 20.603 |
|  | FullRet_Thk_Para_I_Hemisphere | 357 | 244 | 423 | 317.85 | 20.108 |
|  | FullRet_Thk_Para_Tempo | 357 | 201 | 450 | 310.58 | 21.214 |
|  | FullRet_Thk_Para_Superior | 357 | 215 | 432 | 319.58 | 20.849 |
|  | FullRet_Thk_Para_Nasal | 357 | 238 | 417 | 322.40 | 21.159 |
|  | FullRet_Thk_Para_Inferior | 357 | 242 | 411 | 318.24 | 20.273 |
|  | FullRet_Thk_PeriFovea | 357 | 187 | 366 | 283.34 | 17.931 |
|  | FullRet_Thk_Peri_S_Hemisphere | 357 | 201 | 385 | 286.00 | 18.705 |
|  | FullRet_Thk_Peri_I_Hemisphere | 357 | 172 | 347 | 280.56 | 18.020 |
|  | FullRet_Thk_Peri_Tempo | 357 | 95 | 384 | 274.54 | 20.530 |
|  | FullRet_Thk_Peri_Superior | 357 | 206 | 388 | 284.70 | 19.737 |
|  | FullRet_Thk_Peri_Nasal | 357 | 222 | 367 | 298.36 | 18.970 |
|  | FullRet_Thk_Peri_Inferior | 357 | 168 | 353 | 274.88 | 18.621 |
|  | FoveaFullRet_Volumn | 357 | .142 | .361 | .20497 | .021361 |
|  | FullRet_Vol_ParaFovea | 357 | 1.455 | 2.687 | 1.99595 | .127698 |
|  | FullRet_Vol_Para_S_Hemisphere | 357 | .688 | 1.359 | .99776 | .066515 |
|  | FullRet_Vol_Para_I_Hemisphere | 357 | .767 | 1.328 | .99819 | .063836 |
|  | FullRet_Vol_Para_Tempo | 357 | .316 | .707 | .48902 | .039379 |
|  | FullRet_Vol_Para_Superior | 357 | .337 | .678 | .50163 | .034105 |
|  | FullRet_Vol_Para_Nasal | 357 | .373 | .655 | .50573 | .033608 |
|  | FullRet_Vol_Para_Inferior | 357 | .381 | .646 | .49955 | .031755 |
|  | FullRet_Vol_PeriFovea | 357 | 2.344 | 4.596 | 3.55502 | .228768 |
|  | FullRet_Vol_Peri_S_Hemisphere | 357 | 1.263 | 2.416 | 1.79546 | .122322 |
|  | FullRet_Vol_Peri_I_Hemisphere | 357 | 1.080 | 2.180 | 1.75931 | .116631 |
|  | FullRet_Vol_Peri_Tempo | 357 | .299 | 1.205 | .86484 | .069055 |
|  | FullRet_Vol_Peri_Superior | 357 | .573 | 1.219 | .89341 | .070995 |
|  | FullRet_Vol_Peri_Nasal | 357 | .697 | 1.250 | .93648 | .065819 |
|  | FullRet_Vol_Peri_Inferior | 357 | .512 | 1.108 | .86016 | .065606 |
|  | FoveaRPE_Elevation_Height | 357 | -25.34 | 149.85 | 1.6627 | 19.10136 |
|  | RPE_Elev_ParaFovea_Tempo | 357 | -54.7 | 101.5 | 3.555 | 13.3947 |
|  | RPE_Elev_Para_Superior | 357 | -19.5 | 83.5 | 1.209 | 12.1028 |
|  | RPE_Elev_Para_Nasal | 357 | -20.8 | 127.2 | 5.791 | 16.2456 |
|  | RPE_Elev_Para_Inferior | 357 | -62.7 | 91.7 | 3.610 | 13.9564 |
|  | RPE_Elev_PeriFovea_Tempo | 357 | -32.4 | 507.9 | 3.646 | 27.9435 |
|  | RPE_Elev_Peri_Superior | 357 | -23.6 | 120.6 | .648 | 9.8166 |
|  | RPE_Elev_Peri_Nasal | 357 | -25.3 | 82.9 | 4.945 | 12.3261 |
|  | RPE_Elev_Peri_Inferior | 357 | -17.1 | 89.6 | .933 | 8.5614 |
|  | Predicted Category Value | 357 | 2 | 2 | 2.00 | .000 |
|  | Predicted Value of Linear Predictor for [Class_Num=0] | 357 | -182.494 | -128.032 | -159.10747 | 7.161082 |
|  | Predicted Value of Linear Predictor for [Class_Num=1] | 357 | -179.092 | -124.631 | -155.70627 | 7.161082 |
|  | Estimated Standard Error of Predicted Value of Linear Predictor for [Class_Num=0] | 357 | .327 | 3.150 | .64251 | .383441 |
|  | Estimated Standard Error of Predicted Value of Linear Predictor for [Class_Num=1] | 357 | .382 | 3.162 | .67344 | .378289 |
| Offset | AgePerToday | 357 | 53 | 99 | 80.10 | 6.056 |

## Goodness of Fit and Omnibus Test

### Using GCC parameters

| \| **Goodness of Fit^a^** \| \| \| \| \| --- \| --- \| --- \| --- \| \|  \| Value \| df \| Value/df \| \| Deviance \| 2488.983 \| 697 \| 3.571 \| \| Scaled Deviance \| 2488.983 \| 697 \|  \| \| Pearson Chi-Square \| 4873797082.087 \| 697 \| 6992535.268 \| \| Scaled Pearson Chi-Square \| 4873797082.087 \| 697 \|  \| \| Log Likelihood^b^ \| -1244.491 \|  \|  \| \| Akaike's Information Criterion (AIC) \| 2522.983 \|  \|  \| \| Finite Sample Corrected AIC (AICC) \| 2524.788 \|  \|  \| \| Bayesian Information Criterion (BIC) \| 2588.904 \|  \|  \| \| Consistent AIC (CAIC) \| 2605.904 \|  \|  \| \| Dependent Variable: Class_Num  Model: (Threshold), GCC_Inner_Retina_Average, GCC_Superior_Avg, GCC_Inferior_Avg, GCC_S_I_Avg, GCC_FLV, GCC_GLV, GCC_RMS, GCC_Full_Retina_Average, GCC_Superior_Avg.1, GCC_Inferior_Avg.1, GCC_S_I_Avg.1, GCC_Outer_Retina_Average, GCC_Superior_Avg.2, GCC_Inferior_Avg.2, GCC_S_I_Avg.2, offset = AgePerToday^a^ \| \| \| \| \| a. Information criteria are in smaller-is-better form. \| \| \| \| \| b. The full log likelihood function is displayed and used in computing information criteria. \| \| \| \| | \| **Omnibus Test^a^** \| \| \| \| --- \| --- \| --- \| \| Likelihood Ratio Chi-Square \| df \| Sig. \| \| 194.483 \| 15 \| .000 \| \| Dependent Variable: Class_Num  Model: (Threshold), GCC_Inner_Retina_Average, GCC_Superior_Avg, GCC_Inferior_Avg, GCC_S_I_Avg, GCC_FLV, GCC_GLV, GCC_RMS, GCC_Full_Retina_Average, GCC_Superior_Avg.1, GCC_Inferior_Avg.1, GCC_S_I_Avg.1, GCC_Outer_Retina_Average, GCC_Superior_Avg.2, GCC_Inferior_Avg.2, GCC_S_I_Avg.2, offset = AgePerToday^a^ \| \| \| \| a. Compares the fitted model against the thresholds-only model. \| \| \| |
| --- | --- | --- | --- | --- | --- | --- | --- | --- | --- | --- | --- | --- | --- | --- | --- | --- | --- | --- | --- | --- | --- | --- | --- | --- | --- | --- | --- | --- | --- | --- | --- | --- | --- | --- | --- | --- | --- | --- | --- | --- | --- | --- | --- | --- | --- | --- | --- | --- | --- | --- | --- | --- | --- | --- | --- | --- | --- | --- | --- | --- | --- | --- | --- | --- | --- | --- | --- | --- | --- | --- | --- | --- |

### Using ONH parameters

| \| **Goodness of Fit^a^** \| \| \| \| \| --- \| --- \| --- \| --- \| \|  \| Value \| df \| Value/df \| \| Deviance \| 2118.813 \| 672 \| 3.153 \| \| Scaled Deviance \| 2118.813 \| 672 \|  \| \| Pearson Chi-Square \| 4710271518.058 \| 672 \| 7009332.616 \| \| Scaled Pearson Chi-Square \| 4710271518.058 \| 672 \|  \| \| Log Likelihood^b^ \| -1059.406 \|  \|  \| \| Akaike's Information Criterion (AIC) \| 2202.813 \|  \|  \| \| Finite Sample Corrected AIC (AICC) \| 2214.316 \|  \|  \| \| Bayesian Information Criterion (BIC) \| 2365.678 \|  \|  \| \| Consistent AIC (CAIC) \| 2407.678 \|  \|  \| \| Dependent Variable: Class_Num  Model: (Threshold), ONH_DiscArea, ONH_Area_C_D_ratio, ONH_H_C_D_ratio, ONH_V_C_D_ratio, ONH_CupArea, ONH_RimArea, ONH_RimVolume, ONH_Disc_Volume, ONH_CupVolume, ONH_Avg_RNFL, ONH_Sup_RNFL, ONH_Inf_RNFL, ONH_Tempo, ONH_Superior, ONH_Nasal, ONH_Inferior, ONH_RNFL_TU, ONH_ST, ONH_SN, ONH_NU, ONH_NL, ONH_IN, ONH_IT, ONH_TL, ONH_TU1, ONH_TU2, ONH_ST2, ONH_ST1, ONH_SN1, ONH_SN2, ONH_NU2, ONH_NU1, ONH_NL1, ONH_NL2, ONH_IN2, ONH_IN1, ONH_IT1, ONH_IT2, ONH_TL2, ONH_TL1, offset = AgePerToday^a^ \| \| \| \| \| a. Information criteria are in smaller-is-better form. \| \| \| \| \| b. The full log likelihood function is displayed and used in computing information criteria. \| \| \| \| | \| **Omnibus Test^a^** \| \| \| \| --- \| --- \| --- \| \| Likelihood Ratio Chi-Square \| df \| Sig. \| \| 564.653 \| 40 \| .000 \| \| Dependent Variable: Class_Num  Model: (Threshold), ONH_DiscArea, ONH_Area_C_D_ratio, ONH_H_C_D_ratio, ONH_V_C_D_ratio, ONH_CupArea, ONH_RimArea, ONH_RimVolume, ONH_Disc_Volume, ONH_CupVolume, ONH_Avg_RNFL, ONH_Sup_RNFL, ONH_Inf_RNFL, ONH_Tempo, ONH_Superior, ONH_Nasal, ONH_Inferior, ONH_RNFL_TU, ONH_ST, ONH_SN, ONH_NU, ONH_NL, ONH_IN, ONH_IT, ONH_TL, ONH_TU1, ONH_TU2, ONH_ST2, ONH_ST1, ONH_SN1, ONH_SN2, ONH_NU2, ONH_NU1, ONH_NL1, ONH_NL2, ONH_IN2, ONH_IN1, ONH_IT1, ONH_IT2, ONH_TL2, ONH_TL1, offset = AgePerToday^a^ \| \| \| \| a. Compares the fitted model against the thresholds-only model. \| \| \| |
| --- | --- | --- | --- | --- | --- | --- | --- | --- | --- | --- | --- | --- | --- | --- | --- | --- | --- | --- | --- | --- | --- | --- | --- | --- | --- | --- | --- | --- | --- | --- | --- | --- | --- | --- | --- | --- | --- | --- | --- | --- | --- | --- | --- | --- | --- | --- | --- | --- | --- | --- | --- | --- | --- | --- | --- | --- | --- | --- | --- | --- | --- | --- | --- | --- | --- | --- | --- | --- | --- | --- | --- | --- |

### Using Macula_3mm parameters

| \| **Goodness of Fit^a^** \| \| \| \| \| --- \| --- \| --- \| --- \| \|  \| Value \| df \| Value/df \| \| Deviance \| 1656.476 \| 600 \| 2.761 \| \| Scaled Deviance \| 1656.476 \| 600 \|  \| \| Pearson Chi-Square \| 29819315324.458 \| 600 \| 49698858.874 \| \| Scaled Pearson Chi-Square \| 29819315324.458 \| 600 \|  \| \| Log Likelihood^b^ \| -828.238 \|  \|  \| \| Akaike's Information Criterion (AIC) \| 1884.476 \|  \|  \| \| Finite Sample Corrected AIC (AICC) \| 1992.823 \|  \|  \| \| Bayesian Information Criterion (BIC) \| 2326.537 \|  \|  \| \| Consistent AIC (CAIC) \| 2440.537 \|  \|  \| \| Dependent Variable: Class_Num  Model: (Threshold), Macula_3mm_Thk_ILM_IPL_um_Center_1, Macula_3mm_Thk_ILM_IPL_um_T_1minus3, Macula_3mm_Thk_ILM_IPL_um_S_1minus3, Macula_3mm_Thk_ILM_IPL_um_N_1minus3, Macula_3mm_Thk_ILM_IPL_um_I_1minus3, Macula_3mm_Thk_ILM_IPL_um_S_Hemi_1minus3, Macula_3mm_Thk_ILM_IPL_um_I_Hemi_1minus3, Macula_3mm_Thk_ILM_IPL_um_All_1minus3, Macula_3mm_Thk_ILM_IPL_um_S_Hemi_0minus3, Macula_3mm_Thk_ILM_IPL_um_I_Hemi_0minus3, Macula_3mm_Thk_ILM_IPL_um_All_0minus3, Macula_3mm_Thk_ILM_IPL_um_S_Hemi_field, Macula_3mm_Thk_ILM_IPL_um_I_Hemi_field, Macula_3mm_Thk_ILM_IPL_um_All_field, Macula_3mm_Thk_ILM_RPE_um_Center_1, Macula_3mm_Thk_ILM_RPE_um_T_1minus3, Macula_3mm_Thk_ILM_RPE_um_S_1minus3, Macula_3mm_Thk_ILM_RPE_um_N_1minus3, Macula_3mm_Thk_ILM_RPE_um_I_1minus3, Macula_3mm_Thk_ILM_RPE_um_S_Hemi_1minus3, Macula_3mm_Thk_ILM_RPE_um_I_Hemi_1minus3, Macula_3mm_Thk_ILM_RPE_um_All_1minus3, Macula_3mm_Thk_ILM_RPE_um_S_Hemi_0minus3, Macula_3mm_Thk_ILM_RPE_um_I_Hemi_0minus3, Macula_3mm_Thk_ILM_RPE_um_All_0minus3, Macula_3mm_Thk_ILM_RPE_um_S_Hemi_field, Macula_3mm_Thk_ILM_RPE_um_I_Hemi_field, Macula_3mm_Thk_ILM_RPE_um_All_field, Macula_3mm_Thk_ILM_BRM_um_Center_1, Macula_3mm_Thk_ILM_BRM_um_T_1minus3, Macula_3mm_Thk_ILM_BRM_um_S_1minus3, Macula_3mm_Thk_ILM_BRM_um_N_1minus3, Macula_3mm_Thk_ILM_BRM_um_I_1minus3, Macula_3mm_Thk_ILM_BRM_um_S_Hemi_1minus3, Macula_3mm_Thk_ILM_BRM_um_I_Hemi_1minus3, Macula_3mm_Thk_ILM_BRM_um_All_1minus3, Macula_3mm_Thk_ILM_BRM_um_S_Hemi_0minus3, Macula_3mm_Thk_ILM_BRM_um_I_Hemi_0minus3, Macula_3mm_Thk_ILM_BRM_um_All_0minus3, Macula_3mm_Thk_ILM_BRM_um_S_Hemi_field, Macula_3mm_Thk_ILM_BRM_um_I_Hemi_field, Macula_3mm_Thk_ILM_BRM_um_All_field, Macula_3mm_Thk_RPE_BRM_um_Center_1, Macula_3mm_Thk_RPE_BRM_um_T_1minus3, Macula_3mm_Thk_RPE_BRM_um_S_1minus3, Macula_3mm_Thk_RPE_BRM_um_N_1minus3, Macula_3mm_Thk_RPE_BRM_um_I_1minus3, Macula_3mm_Thk_RPE_BRM_um_S_Hemi_1minus3, Macula_3mm_Thk_RPE_BRM_um_I_Hemi_1minus3, Macula_3mm_Thk_RPE_BRM_um_All_1minus3, Macula_3mm_Thk_RPE_BRM_um_S_Hemi_0minus3, Macula_3mm_Thk_RPE_BRM_um_I_Hemi_0minus3, Macula_3mm_Thk_RPE_BRM_um_All_0minus3, Macula_3mm_Thk_RPE_BRM_um_S_Hemi_field, Macula_3mm_Thk_RPE_BRM_um_I_Hemi_field, Macula_3mm_Thk_RPE_BRM_um_All_field, Macula_3mm_Vol_ILM_IPL_mm3_Center_1, Macula_3mm_Vol_ILM_IPL_mm3_T_1minus3, Macula_3mm_Vol_ILM_IPL_mm3_S_1minus3, Macula_3mm_Vol_ILM_IPL_mm3_N_1minus3, Macula_3mm_Vol_ILM_IPL_mm3_I_1minus3, Macula_3mm_Vol_ILM_IPL_mm3_S_Hemi_1minus3, Macula_3mm_Vol_ILM_IPL_mm3_I_Hemi_1minus3, Macula_3mm_Vol_ILM_IPL_mm3_All_1minus3, Macula_3mm_Vol_ILM_IPL_mm3_S_Hemi_0minus3, Macula_3mm_Vol_ILM_IPL_mm3_I_Hemi_0minus3, Macula_3mm_Vol_ILM_IPL_mm3_All_0minus3, Macula_3mm_Vol_ILM_IPL_mm3_S_Hemi_field, Macula_3mm_Vol_ILM_IPL_mm3_I_Hemi_field, Macula_3mm_Vol_ILM_IPL_mm3_All_field, Macula_3mm_Vol_ILM_RPE_mm3_Center_1, Macula_3mm_Vol_ILM_RPE_mm3_T_1minus3, Macula_3mm_Vol_ILM_RPE_mm3_S_1minus3, Macula_3mm_Vol_ILM_RPE_mm3_N_1minus3, Macula_3mm_Vol_ILM_RPE_mm3_I_1minus3, Macula_3mm_Vol_ILM_RPE_mm3_S_Hemi_1minus3, Macula_3mm_Vol_ILM_RPE_mm3_I_Hemi_1minus3, Macula_3mm_Vol_ILM_RPE_mm3_All_1minus3, Macula_3mm_Vol_ILM_RPE_mm3_S_Hemi_0minus3, Macula_3mm_Vol_ILM_RPE_mm3_I_Hemi_0minus3, Macula_3mm_Vol_ILM_RPE_mm3_All_0minus3, Macula_3mm_Vol_ILM_RPE_mm3_S_Hemi_field, Macula_3mm_Vol_ILM_RPE_mm3_I_Hemi_field, Macula_3mm_Vol_ILM_RPE_mm3_All_field, Macula_3mm_Vol_ILM_BRM_mm3_Center_1, Macula_3mm_Vol_ILM_BRM_mm3_T_1minus3, Macula_3mm_Vol_ILM_BRM_mm3_S_1minus3, Macula_3mm_Vol_ILM_BRM_mm3_N_1minus3, Macula_3mm_Vol_ILM_BRM_mm3_I_1minus3, Macula_3mm_Vol_ILM_BRM_mm3_S_Hemi_1minus3, Macula_3mm_Vol_ILM_BRM_mm3_I_Hemi_1minus3, Macula_3mm_Vol_ILM_BRM_mm3_All_1minus3, Macula_3mm_Vol_ILM_BRM_mm3_S_Hemi_0minus3, Macula_3mm_Vol_ILM_BRM_mm3_I_Hemi_0minus3, Macula_3mm_Vol_ILM_BRM_mm3_All_0minus3, Macula_3mm_Vol_ILM_BRM_mm3_S_Hemi_field, Macula_3mm_Vol_ILM_BRM_mm3_I_Hemi_field, Macula_3mm_Vol_ILM_BRM_mm3_All_field, Macula_3mm_Vol_RPE_BRM_mm3_Center_1, Macula_3mm_Vol_RPE_BRM_mm3_T_1minus3, Macula_3mm_Vol_RPE_BRM_mm3_S_1minus3, Macula_3mm_Vol_RPE_BRM_mm3_N_1minus3, Macula_3mm_Vol_RPE_BRM_mm3_I_1minus3, Macula_3mm_Vol_RPE_BRM_mm3_S_Hemi_1minus3, Macula_3mm_Vol_RPE_BRM_mm3_I_Hemi_1minus3, Macula_3mm_Vol_RPE_BRM_mm3_All_1minus3, Macula_3mm_Vol_RPE_BRM_mm3_S_Hemi_0minus3, Macula_3mm_Vol_RPE_BRM_mm3_I_Hemi_0minus3, Macula_3mm_Vol_RPE_BRM_mm3_All_0minus3, Macula_3mm_Vol_RPE_BRM_mm3_S_Hemi_field, Macula_3mm_Vol_RPE_BRM_mm3_I_Hemi_field, Macula_3mm_Vol_RPE_BRM_mm3_All_field, offset = AgePerToday^a^ \| \| \| \| \| a. Information criteria are in smaller-is-better form. \| \| \| \| \| b. The full log likelihood function is displayed and used in computing information criteria. \| \| \| \| | \| **Omnibus Test^a^** \| \| \| \| --- \| --- \| --- \| \| Likelihood Ratio Chi-Square \| df \| Sig. \| \| 1026.990 \| 112 \| .000 \| \| Dependent Variable: Class_Num  Model: (Threshold), Macula_3mm_Thk_ILM_IPL_um_Center_1, Macula_3mm_Thk_ILM_IPL_um_T_1minus3, Macula_3mm_Thk_ILM_IPL_um_S_1minus3, Macula_3mm_Thk_ILM_IPL_um_N_1minus3, Macula_3mm_Thk_ILM_IPL_um_I_1minus3, Macula_3mm_Thk_ILM_IPL_um_S_Hemi_1minus3, Macula_3mm_Thk_ILM_IPL_um_I_Hemi_1minus3, Macula_3mm_Thk_ILM_IPL_um_All_1minus3, Macula_3mm_Thk_ILM_IPL_um_S_Hemi_0minus3, Macula_3mm_Thk_ILM_IPL_um_I_Hemi_0minus3, Macula_3mm_Thk_ILM_IPL_um_All_0minus3, Macula_3mm_Thk_ILM_IPL_um_S_Hemi_field, Macula_3mm_Thk_ILM_IPL_um_I_Hemi_field, Macula_3mm_Thk_ILM_IPL_um_All_field, Macula_3mm_Thk_ILM_RPE_um_Center_1, Macula_3mm_Thk_ILM_RPE_um_T_1minus3, Macula_3mm_Thk_ILM_RPE_um_S_1minus3, Macula_3mm_Thk_ILM_RPE_um_N_1minus3, Macula_3mm_Thk_ILM_RPE_um_I_1minus3, Macula_3mm_Thk_ILM_RPE_um_S_Hemi_1minus3, Macula_3mm_Thk_ILM_RPE_um_I_Hemi_1minus3, Macula_3mm_Thk_ILM_RPE_um_All_1minus3, Macula_3mm_Thk_ILM_RPE_um_S_Hemi_0minus3, Macula_3mm_Thk_ILM_RPE_um_I_Hemi_0minus3, Macula_3mm_Thk_ILM_RPE_um_All_0minus3, Macula_3mm_Thk_ILM_RPE_um_S_Hemi_field, Macula_3mm_Thk_ILM_RPE_um_I_Hemi_field, Macula_3mm_Thk_ILM_RPE_um_All_field, Macula_3mm_Thk_ILM_BRM_um_Center_1, Macula_3mm_Thk_ILM_BRM_um_T_1minus3, Macula_3mm_Thk_ILM_BRM_um_S_1minus3, Macula_3mm_Thk_ILM_BRM_um_N_1minus3, Macula_3mm_Thk_ILM_BRM_um_I_1minus3, Macula_3mm_Thk_ILM_BRM_um_S_Hemi_1minus3, Macula_3mm_Thk_ILM_BRM_um_I_Hemi_1minus3, Macula_3mm_Thk_ILM_BRM_um_All_1minus3, Macula_3mm_Thk_ILM_BRM_um_S_Hemi_0minus3, Macula_3mm_Thk_ILM_BRM_um_I_Hemi_0minus3, Macula_3mm_Thk_ILM_BRM_um_All_0minus3, Macula_3mm_Thk_ILM_BRM_um_S_Hemi_field, Macula_3mm_Thk_ILM_BRM_um_I_Hemi_field, Macula_3mm_Thk_ILM_BRM_um_All_field, Macula_3mm_Thk_RPE_BRM_um_Center_1, Macula_3mm_Thk_RPE_BRM_um_T_1minus3, Macula_3mm_Thk_RPE_BRM_um_S_1minus3, Macula_3mm_Thk_RPE_BRM_um_N_1minus3, Macula_3mm_Thk_RPE_BRM_um_I_1minus3, Macula_3mm_Thk_RPE_BRM_um_S_Hemi_1minus3, Macula_3mm_Thk_RPE_BRM_um_I_Hemi_1minus3, Macula_3mm_Thk_RPE_BRM_um_All_1minus3, Macula_3mm_Thk_RPE_BRM_um_S_Hemi_0minus3, Macula_3mm_Thk_RPE_BRM_um_I_Hemi_0minus3, Macula_3mm_Thk_RPE_BRM_um_All_0minus3, Macula_3mm_Thk_RPE_BRM_um_S_Hemi_field, Macula_3mm_Thk_RPE_BRM_um_I_Hemi_field, Macula_3mm_Thk_RPE_BRM_um_All_field, Macula_3mm_Vol_ILM_IPL_mm3_Center_1, Macula_3mm_Vol_ILM_IPL_mm3_T_1minus3, Macula_3mm_Vol_ILM_IPL_mm3_S_1minus3, Macula_3mm_Vol_ILM_IPL_mm3_N_1minus3, Macula_3mm_Vol_ILM_IPL_mm3_I_1minus3, Macula_3mm_Vol_ILM_IPL_mm3_S_Hemi_1minus3, Macula_3mm_Vol_ILM_IPL_mm3_I_Hemi_1minus3, Macula_3mm_Vol_ILM_IPL_mm3_All_1minus3, Macula_3mm_Vol_ILM_IPL_mm3_S_Hemi_0minus3, Macula_3mm_Vol_ILM_IPL_mm3_I_Hemi_0minus3, Macula_3mm_Vol_ILM_IPL_mm3_All_0minus3, Macula_3mm_Vol_ILM_IPL_mm3_S_Hemi_field, Macula_3mm_Vol_ILM_IPL_mm3_I_Hemi_field, Macula_3mm_Vol_ILM_IPL_mm3_All_field, Macula_3mm_Vol_ILM_RPE_mm3_Center_1, Macula_3mm_Vol_ILM_RPE_mm3_T_1minus3, Macula_3mm_Vol_ILM_RPE_mm3_S_1minus3, Macula_3mm_Vol_ILM_RPE_mm3_N_1minus3, Macula_3mm_Vol_ILM_RPE_mm3_I_1minus3, Macula_3mm_Vol_ILM_RPE_mm3_S_Hemi_1minus3, Macula_3mm_Vol_ILM_RPE_mm3_I_Hemi_1minus3, Macula_3mm_Vol_ILM_RPE_mm3_All_1minus3, Macula_3mm_Vol_ILM_RPE_mm3_S_Hemi_0minus3, Macula_3mm_Vol_ILM_RPE_mm3_I_Hemi_0minus3, Macula_3mm_Vol_ILM_RPE_mm3_All_0minus3, Macula_3mm_Vol_ILM_RPE_mm3_S_Hemi_field, Macula_3mm_Vol_ILM_RPE_mm3_I_Hemi_field, Macula_3mm_Vol_ILM_RPE_mm3_All_field, Macula_3mm_Vol_ILM_BRM_mm3_Center_1, Macula_3mm_Vol_ILM_BRM_mm3_T_1minus3, Macula_3mm_Vol_ILM_BRM_mm3_S_1minus3, Macula_3mm_Vol_ILM_BRM_mm3_N_1minus3, Macula_3mm_Vol_ILM_BRM_mm3_I_1minus3, Macula_3mm_Vol_ILM_BRM_mm3_S_Hemi_1minus3, Macula_3mm_Vol_ILM_BRM_mm3_I_Hemi_1minus3, Macula_3mm_Vol_ILM_BRM_mm3_All_1minus3, Macula_3mm_Vol_ILM_BRM_mm3_S_Hemi_0minus3, Macula_3mm_Vol_ILM_BRM_mm3_I_Hemi_0minus3, Macula_3mm_Vol_ILM_BRM_mm3_All_0minus3, Macula_3mm_Vol_ILM_BRM_mm3_S_Hemi_field, Macula_3mm_Vol_ILM_BRM_mm3_I_Hemi_field, Macula_3mm_Vol_ILM_BRM_mm3_All_field, Macula_3mm_Vol_RPE_BRM_mm3_Center_1, Macula_3mm_Vol_RPE_BRM_mm3_T_1minus3, Macula_3mm_Vol_RPE_BRM_mm3_S_1minus3, Macula_3mm_Vol_RPE_BRM_mm3_N_1minus3, Macula_3mm_Vol_RPE_BRM_mm3_I_1minus3, Macula_3mm_Vol_RPE_BRM_mm3_S_Hemi_1minus3, Macula_3mm_Vol_RPE_BRM_mm3_I_Hemi_1minus3, Macula_3mm_Vol_RPE_BRM_mm3_All_1minus3, Macula_3mm_Vol_RPE_BRM_mm3_S_Hemi_0minus3, Macula_3mm_Vol_RPE_BRM_mm3_I_Hemi_0minus3, Macula_3mm_Vol_RPE_BRM_mm3_All_0minus3, Macula_3mm_Vol_RPE_BRM_mm3_S_Hemi_field, Macula_3mm_Vol_RPE_BRM_mm3_I_Hemi_field, Macula_3mm_Vol_RPE_BRM_mm3_All_field, offset = AgePerToday^a^ \| \| \| \| a. Compares the fitted model against the thresholds-only model. \| \| \| |
| --- | --- | --- | --- | --- | --- | --- | --- | --- | --- | --- | --- | --- | --- | --- | --- | --- | --- | --- | --- | --- | --- | --- | --- | --- | --- | --- | --- | --- | --- | --- | --- | --- | --- | --- | --- | --- | --- | --- | --- | --- | --- | --- | --- | --- | --- | --- | --- | --- | --- | --- | --- | --- | --- | --- | --- | --- | --- | --- | --- | --- | --- | --- | --- | --- | --- | --- | --- | --- | --- | --- | --- | --- |

### Using Retina3DflowDensity parameters

| \| \| **Goodness of Fit^a^** \| \| \| \| \| --- \| --- \| --- \| --- \| \|  \| Value \| df \| Value/df \| \| Deviance \| 1949.884 \| 665 \| 2.932 \| \| Scaled Deviance \| 1949.884 \| 665 \|  \| \| Pearson Chi-Square \| 415233593.990 \| 665 \| 624411.420 \| \| Scaled Pearson Chi-Square \| 415233593.990 \| 665 \|  \| \| Log Likelihood^b^ \| -974.942 \|  \|  \| \| Akaike's Information Criterion (AIC) \| 2047.884 \|  \|  \| \| Finite Sample Corrected AIC (AICC) \| 2063.845 \|  \|  \| \| Bayesian Information Criterion (BIC) \| 2237.893 \|  \|  \| \| Consistent AIC (CAIC) \| 2286.893 \|  \|  \| \| Dependent Variable: Class_Num  Model: (Threshold), SVC_L1_DensityOfWhole_Image, SVC_L1_Whole_Image_S_Hemi, SVC_L1_Whole_Image_I_Hemi, SVC_L1_Whole_ETDRS, SVC_L1_Fovea, SVC_L1_ParaFovea, SVC_L1_Para_S_Hemi, SVC_L1_Para_I_Hemi, SVC_L1_Para_T, SVC_L1_Para_S, SVC_L1_Para_N, SVC_L1_Para_I, SVC_L1_G11, SVC_L1_G12, SVC_L1_G13, SVC_L1_G21, SVC_L1_G22, SVC_L1_G23, SVC_L1_G31, SVC_L1_G32, SVC_L1_G33, DVC_L2_DensityOfWhole_Image, DVC_L2_Whole_Image_S_Hemi, DVC_L2_Whole_Image_I_Hemi, DVC_L2_Whole_ETDRS, DVC_L2_Fovea, DVC_L2_ParaFovea, DVC_L2_Para_S_Hemi, DVC_L2_Para_I_Hemi, DVC_L2_Para_T, DVC_L2_Para_S, DVC_L2_Para_N, DVC_L2_Para_I, DVC_L2_G11, DVC_L2_G12, DVC_L2_G13, DVC_L2_G21, DVC_L2_G22, DVC_L2_G23, DVC_L2_G31, DVC_L2_G32, DVC_L2_G33, FAZ_Area, Perimeter, AcircularityIndex, FD_300_Area_Density, FD_300_Length_Density, offset = AgePerToday^a^ \| \| \| \| \| a. Information criteria are in smaller-is-better form. \| \| \| \| \| b. The full log likelihood function is displayed and used in computing information criteria. \| \| \| \| \| \| --- \| --- \| --- \| --- \| --- \| --- \| --- \| --- \| --- \| --- \| --- \| --- \| --- \| --- \| --- \| --- \| --- \| --- \| --- \| --- \| --- \| --- \| --- \| --- \| --- \| --- \| --- \| --- \| --- \| --- \| --- \| --- \| --- \| --- \| --- \| --- \| --- \| --- \| --- \| --- \| --- \| --- \| --- \| --- \| --- \| --- \| --- \| --- \| --- \| --- \| --- \| --- \| --- \| --- \| --- \| --- \| --- \| | \| **Omnibus Test^a^** \| \| \| \| --- \| --- \| --- \| \| Likelihood Ratio Chi-Square \| df \| Sig. \| \| 733.581 \| 47 \| .000 \| \| Dependent Variable: Class_Num  Model: (Threshold), SVC_L1_DensityOfWhole_Image, SVC_L1_Whole_Image_S_Hemi, SVC_L1_Whole_Image_I_Hemi, SVC_L1_Whole_ETDRS, SVC_L1_Fovea, SVC_L1_ParaFovea, SVC_L1_Para_S_Hemi, SVC_L1_Para_I_Hemi, SVC_L1_Para_T, SVC_L1_Para_S, SVC_L1_Para_N, SVC_L1_Para_I, SVC_L1_G11, SVC_L1_G12, SVC_L1_G13, SVC_L1_G21, SVC_L1_G22, SVC_L1_G23, SVC_L1_G31, SVC_L1_G32, SVC_L1_G33, DVC_L2_DensityOfWhole_Image, DVC_L2_Whole_Image_S_Hemi, DVC_L2_Whole_Image_I_Hemi, DVC_L2_Whole_ETDRS, DVC_L2_Fovea, DVC_L2_ParaFovea, DVC_L2_Para_S_Hemi, DVC_L2_Para_I_Hemi, DVC_L2_Para_T, DVC_L2_Para_S, DVC_L2_Para_N, DVC_L2_Para_I, DVC_L2_G11, DVC_L2_G12, DVC_L2_G13, DVC_L2_G21, DVC_L2_G22, DVC_L2_G23, DVC_L2_G31, DVC_L2_G32, DVC_L2_G33, FAZ_Area, Perimeter, AcircularityIndex, FD_300_Area_Density, FD_300_Length_Density, offset = AgePerToday^a^ \| \| \| \| a. Compares the fitted model against the thresholds-only model. \| \| \| |
| --- | --- | --- | --- | --- | --- | --- | --- | --- | --- | --- | --- | --- | --- | --- | --- | --- | --- | --- | --- | --- | --- | --- | --- | --- | --- | --- | --- | --- | --- | --- | --- | --- | --- | --- | --- | --- | --- | --- | --- | --- | --- | --- | --- | --- | --- | --- | --- | --- | --- | --- | --- | --- | --- | --- | --- | --- | --- | --- | --- | --- | --- | --- | --- | --- | --- | --- | --- | --- | --- | --- | --- | --- | --- |

### Using Retina Map - Inner Retina Thickness parameters

| \| \| **Goodness of Fit^a^** \| \| \| \| \| --- \| --- \| --- \| --- \| \|  \| Value \| df \| Value/df \| \| Deviance \| 2371.330 \| 697 \| 3.402 \| \| Scaled Deviance \| 2371.330 \| 697 \|  \| \| Pearson Chi-Square \| 20757012713.755 \| 697 \| 29780506.046 \| \| Scaled Pearson Chi-Square \| 20757012713.755 \| 697 \|  \| \| Log Likelihood^b^ \| -1185.665 \|  \|  \| \| Akaike's Information Criterion (AIC) \| 2405.330 \|  \|  \| \| Finite Sample Corrected AIC (AICC) \| 2407.135 \|  \|  \| \| Bayesian Information Criterion (BIC) \| 2471.251 \|  \|  \| \| Consistent AIC (CAIC) \| 2488.251 \|  \|  \| \| Dependent Variable: Class_Num  Model: (Threshold), FoveaInnRet_Thickness, InnRet_Thk_ParaFovea, InnRet_Thk_Para_S_Hemisphere, InnRet_Thk_Para_I_Hemisphere, InnRet_Thk_Para_Tempo, InnRet_Thk_Para_Superior, InnRet_Thk_Para_Nasal, InnRet_Thk_Para_Inferior, InnRet_Thk_PeriFovea, InnRet_Thk_Peri_S_Hemisphere, InnRet_Thk_Peri_I_Hemisphere, InnRet_Thk_Peri_Tempo, InnRet_Thk_Peri_Superior, InnRet_Thk_Peri_Nasal, InnRet_Thk_Peri_Inferior, offset = AgePerToday^a^ \| \| \| \| \| a. Information criteria are in smaller-is-better form. \| \| \| \| \| b. The full log likelihood function is displayed and used in computing information criteria. \| \| \| \| \| \| --- \| --- \| --- \| --- \| --- \| --- \| --- \| --- \| --- \| --- \| --- \| --- \| --- \| --- \| --- \| --- \| --- \| --- \| --- \| --- \| --- \| --- \| --- \| --- \| --- \| --- \| --- \| --- \| --- \| --- \| --- \| --- \| --- \| --- \| --- \| --- \| --- \| --- \| --- \| --- \| --- \| --- \| --- \| --- \| --- \| --- \| --- \| --- \| --- \| --- \| --- \| --- \| --- \| --- \| --- \| --- \| --- \| \|  \| \|  \| | \| **Omnibus Test^a^** \| \| \| \| --- \| --- \| --- \| \| Likelihood Ratio Chi-Square \| df \| Sig. \| \| 312.136 \| 15 \| .000 \| \| Dependent Variable: Class_Num  Model: (Threshold), FoveaInnRet_Thickness, InnRet_Thk_ParaFovea, InnRet_Thk_Para_S_Hemisphere, InnRet_Thk_Para_I_Hemisphere, InnRet_Thk_Para_Tempo, InnRet_Thk_Para_Superior, InnRet_Thk_Para_Nasal, InnRet_Thk_Para_Inferior, InnRet_Thk_PeriFovea, InnRet_Thk_Peri_S_Hemisphere, InnRet_Thk_Peri_I_Hemisphere, InnRet_Thk_Peri_Tempo, InnRet_Thk_Peri_Superior, InnRet_Thk_Peri_Nasal, InnRet_Thk_Peri_Inferior, offset = AgePerToday^a^ \| \| \| \| a. Compares the fitted model against the thresholds-only model. \| \| \| |
| --- | --- | --- | --- | --- | --- | --- | --- | --- | --- | --- | --- | --- | --- | --- | --- | --- | --- | --- | --- | --- | --- | --- | --- | --- | --- | --- | --- | --- | --- | --- | --- | --- | --- | --- | --- | --- | --- | --- | --- | --- | --- | --- | --- | --- | --- | --- | --- | --- | --- | --- | --- | --- | --- | --- | --- | --- | --- | --- | --- | --- | --- | --- | --- | --- | --- | --- | --- | --- | --- | --- | --- | --- | --- | --- | --- |

### Using Retina Map - Inner Retina Volume parameters

| \| \| **Goodness of Fit^a^** \| \| \| \| \| --- \| --- \| --- \| --- \| \|  \| Value \| df \| Value/df \| \| Deviance \| 2379.674 \| 697 \| 3.414 \| \| Scaled Deviance \| 2379.674 \| 697 \|  \| \| Pearson Chi-Square \| 6724247388.406 \| 697 \| 9647413.757 \| \| Scaled Pearson Chi-Square \| 6724247388.406 \| 697 \|  \| \| Log Likelihood^b^ \| -1189.837 \|  \|  \| \| Akaike's Information Criterion (AIC) \| 2413.674 \|  \|  \| \| Finite Sample Corrected AIC (AICC) \| 2415.479 \|  \|  \| \| Bayesian Information Criterion (BIC) \| 2479.595 \|  \|  \| \| Consistent AIC (CAIC) \| 2496.595 \|  \|  \| \| Dependent Variable: Class_Num  Model: (Threshold), FoveaInnRet_Volumn, InnRet_Vol_ParaFovea, InnRet_Vol_Para_S_Hemisphere, InnRet_Vol_Para_I_Hemisphere, InnRet_Vol_Para_Tempo, InnRet_Vol_Para_Superior, InnRet_Vol_Para_Nasal, InnRet_Vol_Para_Inferior, InnRet_Vol_PeriFovea, InnRet_Vol_Peri_S_Hemisphere, InnRet_Vol_Peri_I_Hemisphere, InnRet_Vol_Peri_Tempo, InnRet_Vol_Peri_Superior, InnRet_Vol_Peri_Nasal, InnRet_Vol_Peri_Inferior, offset = AgePerToday^a^ \| \| \| \| \| a. Information criteria are in smaller-is-better form. \| \| \| \| \| b. The full log likelihood function is displayed and used in computing information criteria. \| \| \| \| \| \| --- \| --- \| --- \| --- \| --- \| --- \| --- \| --- \| --- \| --- \| --- \| --- \| --- \| --- \| --- \| --- \| --- \| --- \| --- \| --- \| --- \| --- \| --- \| --- \| --- \| --- \| --- \| --- \| --- \| --- \| --- \| --- \| --- \| --- \| --- \| --- \| --- \| --- \| --- \| --- \| --- \| --- \| --- \| --- \| --- \| --- \| --- \| --- \| --- \| --- \| --- \| --- \| --- \| --- \| --- \| --- \| --- \| \|  \| \|  \| | \| **Omnibus Test^a^** \| \| \| \| --- \| --- \| --- \| \| Likelihood Ratio Chi-Square \| df \| Sig. \| \| 303.792 \| 15 \| .000 \| \| Dependent Variable: Class_Num  Model: (Threshold), FoveaInnRet_Volumn, InnRet_Vol_ParaFovea, InnRet_Vol_Para_S_Hemisphere, InnRet_Vol_Para_I_Hemisphere, InnRet_Vol_Para_Tempo, InnRet_Vol_Para_Superior, InnRet_Vol_Para_Nasal, InnRet_Vol_Para_Inferior, InnRet_Vol_PeriFovea, InnRet_Vol_Peri_S_Hemisphere, InnRet_Vol_Peri_I_Hemisphere, InnRet_Vol_Peri_Tempo, InnRet_Vol_Peri_Superior, InnRet_Vol_Peri_Nasal, InnRet_Vol_Peri_Inferior, offset = AgePerToday^a^ \| \| \| \| a. Compares the fitted model against the thresholds-only model. \| \| \| |
| --- | --- | --- | --- | --- | --- | --- | --- | --- | --- | --- | --- | --- | --- | --- | --- | --- | --- | --- | --- | --- | --- | --- | --- | --- | --- | --- | --- | --- | --- | --- | --- | --- | --- | --- | --- | --- | --- | --- | --- | --- | --- | --- | --- | --- | --- | --- | --- | --- | --- | --- | --- | --- | --- | --- | --- | --- | --- | --- | --- | --- | --- | --- | --- | --- | --- | --- | --- | --- | --- | --- | --- | --- | --- | --- | --- |

### Using Retina Map - Full Retina Thickness/Volume and RPE parameters

| \| \| **Goodness of Fit^a^** \| \| \| \| \| --- \| --- \| --- \| --- \| \|  \| Value \| df \| Value/df \| \| Deviance \| 2099.569 \| 673 \| 3.120 \| \| Scaled Deviance \| 2099.569 \| 673 \|  \| \| Pearson Chi-Square \| 22365126637.913 \| 673 \| 33231986.089 \| \| Scaled Pearson Chi-Square \| 22365126637.913 \| 673 \|  \| \| Log Likelihood^b^ \| -1049.785 \|  \|  \| \| Akaike's Information Criterion (AIC) \| 2181.569 \|  \|  \| \| Finite Sample Corrected AIC (AICC) \| 2192.502 \|  \|  \| \| Bayesian Information Criterion (BIC) \| 2340.556 \|  \|  \| \| Consistent AIC (CAIC) \| 2381.556 \|  \|  \| \| Dependent Variable: Class_Num  Model: (Threshold), FoveaFullRet_Thickness, FullRet_Thk_ParaFovea, FullRet_Thk_Para_S_Hemisphere, FullRet_Thk_Para_I_Hemisphere, FullRet_Thk_Para_Tempo, FullRet_Thk_Para_Superior, FullRet_Thk_Para_Nasal, FullRet_Thk_Para_Inferior, FullRet_Thk_PeriFovea, FullRet_Thk_Peri_S_Hemisphere, FullRet_Thk_Peri_I_Hemisphere, FullRet_Thk_Peri_Tempo, FullRet_Thk_Peri_Superior, FullRet_Thk_Peri_Nasal, FullRet_Thk_Peri_Inferior, FoveaFullRet_Volumn, FullRet_Vol_ParaFovea, FullRet_Vol_Para_S_Hemisphere, FullRet_Vol_Para_I_Hemisphere, FullRet_Vol_Para_Tempo, FullRet_Vol_Para_Superior, FullRet_Vol_Para_Nasal, FullRet_Vol_Para_Inferior, FullRet_Vol_PeriFovea, FullRet_Vol_Peri_S_Hemisphere, FullRet_Vol_Peri_I_Hemisphere, FullRet_Vol_Peri_Tempo, FullRet_Vol_Peri_Superior, FullRet_Vol_Peri_Nasal, FullRet_Vol_Peri_Inferior, FoveaRPE_Elevation_Height, RPE_Elev_ParaFovea_Tempo, RPE_Elev_Para_Superior, RPE_Elev_Para_Nasal, RPE_Elev_Para_Inferior, RPE_Elev_PeriFovea_Tempo, RPE_Elev_Peri_Superior, RPE_Elev_Peri_Nasal, RPE_Elev_Peri_Inferior, offset = AgePerToday^a^ \| \| \| \| \| a. Information criteria are in smaller-is-better form. \| \| \| \| \| b. The full log likelihood function is displayed and used in computing information criteria. \| \| \| \| \| \| --- \| --- \| --- \| --- \| --- \| --- \| --- \| --- \| --- \| --- \| --- \| --- \| --- \| --- \| --- \| --- \| --- \| --- \| --- \| --- \| --- \| --- \| --- \| --- \| --- \| --- \| --- \| --- \| --- \| --- \| --- \| --- \| --- \| --- \| --- \| --- \| --- \| --- \| --- \| --- \| --- \| --- \| --- \| --- \| --- \| --- \| --- \| --- \| --- \| --- \| --- \| --- \| --- \| --- \| --- \| --- \| --- \| | \| **Omnibus Test^a^** \| \| \| \| --- \| --- \| --- \| \| Likelihood Ratio Chi-Square \| df \| Sig. \| \| 583.896 \| 39 \| .000 \| \| Dependent Variable: Class_Num  Model: (Threshold), FoveaFullRet_Thickness, FullRet_Thk_ParaFovea, FullRet_Thk_Para_S_Hemisphere, FullRet_Thk_Para_I_Hemisphere, FullRet_Thk_Para_Tempo, FullRet_Thk_Para_Superior, FullRet_Thk_Para_Nasal, FullRet_Thk_Para_Inferior, FullRet_Thk_PeriFovea, FullRet_Thk_Peri_S_Hemisphere, FullRet_Thk_Peri_I_Hemisphere, FullRet_Thk_Peri_Tempo, FullRet_Thk_Peri_Superior, FullRet_Thk_Peri_Nasal, FullRet_Thk_Peri_Inferior, FoveaFullRet_Volumn, FullRet_Vol_ParaFovea, FullRet_Vol_Para_S_Hemisphere, FullRet_Vol_Para_I_Hemisphere, FullRet_Vol_Para_Tempo, FullRet_Vol_Para_Superior, FullRet_Vol_Para_Nasal, FullRet_Vol_Para_Inferior, FullRet_Vol_PeriFovea, FullRet_Vol_Peri_S_Hemisphere, FullRet_Vol_Peri_I_Hemisphere, FullRet_Vol_Peri_Tempo, FullRet_Vol_Peri_Superior, FullRet_Vol_Peri_Nasal, FullRet_Vol_Peri_Inferior, FoveaRPE_Elevation_Height, RPE_Elev_ParaFovea_Tempo, RPE_Elev_Para_Superior, RPE_Elev_Para_Nasal, RPE_Elev_Para_Inferior, RPE_Elev_PeriFovea_Tempo, RPE_Elev_Peri_Superior, RPE_Elev_Peri_Nasal, RPE_Elev_Peri_Inferior, offset = AgePerToday^a^ \| \| \| \| a. Compares the fitted model against the thresholds-only model. \| \| \| |
| --- | --- | --- | --- | --- | --- | --- | --- | --- | --- | --- | --- | --- | --- | --- | --- | --- | --- | --- | --- | --- | --- | --- | --- | --- | --- | --- | --- | --- | --- | --- | --- | --- | --- | --- | --- | --- | --- | --- | --- | --- | --- | --- | --- | --- | --- | --- | --- | --- | --- | --- | --- | --- | --- | --- | --- | --- | --- | --- | --- | --- | --- | --- | --- | --- | --- | --- | --- | --- | --- | --- | --- | --- | --- |

## Tests of Model Effects

### Using GCC parameters

| **Tests of Model Effects** | | | |
| --- | --- | --- | --- |
| Source | Type III | | |
|  | Wald Chi-Square | df | Sig. |
| GCC_Inner_Retina_Average | 1.499 | 1 | .221 |
| GCC_Superior_Avg | 1.256 | 1 | .262 |
| GCC_Inferior_Avg | .609 | 1 | .435 |
| GCC_S_I_Avg | 1.431 | 1 | .232 |
| GCC_FLV | .035 | 1 | .852 |
| GCC_GLV | .000 | 1 | .987 |
| GCC_RMS | 1.538 | 1 | .215 |
| GCC_Full_Retina_Average | 1.292 | 1 | .256 |
| GCC_Superior_Avg.1 | .149 | 1 | .699 |
| GCC_Inferior_Avg.1 | .286 | 1 | .593 |
| GCC_S_I_Avg.1 | .044 | 1 | .834 |
| GCC_Outer_Retina_Average | 1.324 | 1 | .250 |
| GCC_Superior_Avg.2 | .098 | 1 | .754 |
| GCC_Inferior_Avg.2 | 1.140 | 1 | .286 |
| GCC_S_I_Avg.2 | .151 | 1 | .697 |
| Dependent Variable: Class_Num  Model: (Threshold), GCC_Inner_Retina_Average, GCC_Superior_Avg, GCC_Inferior_Avg, GCC_S_I_Avg, GCC_FLV, GCC_GLV, GCC_RMS, GCC_Full_Retina_Average, GCC_Superior_Avg.1, GCC_Inferior_Avg.1, GCC_S_I_Avg.1, GCC_Outer_Retina_Average, GCC_Superior_Avg.2, GCC_Inferior_Avg.2, GCC_S_I_Avg.2, offset = AgePerToday | | | |

### Using ONH parameters

| **Tests of Model Effects** | | | |
| --- | --- | --- | --- |
| Source | Type III | | |
|  | Wald Chi-Square | df | Sig. |
| ONH_DiscArea | 6.134 | 1 | **.013** |
| ONH_Area_C_D_ratio | 22.861 | 1 | **<.001** |
| ONH_H_C_D_ratio | 19.762 | 1 | **<.001** |
| ONH_V_C_D_ratio | 33.148 | 1 | **<.001** |
| ONH_CupArea | 13.473 | 1 | **<.001** |
| ONH_RimArea | 8.843 | 1 | **.003** |
| ONH_RimVolume | 6.271 | 1 | **.012** |
| ONH_Disc_Volume | .948 | 1 | .330 |
| ONH_CupVolume | 37.742 | 1 | **<.001** |
| ONH_Avg_RNFL | 3.921 | 1 | **.048** |
| ONH_Sup_RNFL | 2.346 | 1 | .126 |
| ONH_Inf_RNFL | 4.565 | 1 | **.033** |
| ONH_Tempo | 22.020 | 1 | **<.001** |
| ONH_Superior | 2.581 | 1 | .108 |
| ONH_Nasal | 11.487 | 1 | **<.001** |
| ONH_Inferior | 3.878 | 1 | **.049** |
| ONH_RNFL_TU | 5.947 | 1 | **.015** |
| ONH_ST | .265 | 1 | .607 |
| ONH_SN | .089 | 1 | .766 |
| ONH_NU | 14.580 | 1 | **<.001** |
| ONH_NL | 34.231 | 1 | **<.001** |
| ONH_IN | .735 | 1 | .391 |
| ONH_IT | 19.772 | 1 | **<.001** |
| ONH_TL | 45.351 | 1 | **<.001** |
| ONH_TU1 | 8.447 | 1 | **.004** |
| ONH_TU2 | .073 | 1 | .787 |
| ONH_ST2 | .727 | 1 | .394 |
| ONH_ST1 | 18.069 | 1 | **<.001** |
| ONH_SN1 | 12.638 | 1 | **<.001** |
| ONH_SN2 | .076 | 1 | .783 |
| ONH_NU2 | .757 | 1 | .384 |
| ONH_NU1 | .001 | 1 | .970 |
| ONH_NL1 | 31.911 | 1 | **<.001** |
| ONH_NL2 | 33.025 | 1 | **<.001** |
| ONH_IN2 | 5.637 | 1 | **.018** |
| ONH_IN1 | 6.526 | 1 | **.011** |
| ONH_IT1 | 1.537 | 1 | .215 |
| ONH_IT2 | 1.087 | 1 | .297 |
| ONH_TL2 | 20.846 | 1 | **<.001** |
| ONH_TL1 | 2.420 | 1 | .120 |
| Dependent Variable: Class_Num  Model: (Threshold), ONH_DiscArea, ONH_Area_C_D_ratio, ONH_H_C_D_ratio, ONH_V_C_D_ratio, ONH_CupArea, ONH_RimArea, ONH_RimVolume, ONH_Disc_Volume, ONH_CupVolume, ONH_Avg_RNFL, ONH_Sup_RNFL, ONH_Inf_RNFL, ONH_Tempo, ONH_Superior, ONH_Nasal, ONH_Inferior, ONH_RNFL_TU, ONH_ST, ONH_SN, ONH_NU, ONH_NL, ONH_IN, ONH_IT, ONH_TL, ONH_TU1, ONH_TU2, ONH_ST2, ONH_ST1, ONH_SN1, ONH_SN2, ONH_NU2, ONH_NU1, ONH_NL1, ONH_NL2, ONH_IN2, ONH_IN1, ONH_IT1, ONH_IT2, ONH_TL2, ONH_TL1, offset = AgePerToday | | | |

### Using Macula_3mm parameters

| **Tests of Model Effects** | | | |
| --- | --- | --- | --- |
| Source | Type III | | |
|  | Wald Chi-Square | df | Sig. |
| Macula_3mm_Thk_ILM_IPL_um_Center_1 | 2.922 | 1 | .087 |
| Macula_3mm_Thk_ILM_IPL_um_T_1minus3 | .673 | 1 | .412 |
| Macula_3mm_Thk_ILM_IPL_um_S_1minus3 | .583 | 1 | .445 |
| Macula_3mm_Thk_ILM_IPL_um_N_1minus3 | .507 | 1 | .477 |
| Macula_3mm_Thk_ILM_IPL_um_I_1minus3 | .446 | 1 | .504 |
| Macula_3mm_Thk_ILM_IPL_um_S_Hemi_1minus3 | 60.661 | 1 | **<.001** |
| Macula_3mm_Thk_ILM_IPL_um_I_Hemi_1minus3 | .605 | 1 | .437 |
| Macula_3mm_Thk_ILM_IPL_um_All_1minus3 | 3.833 | 1 | .050 |
| Macula_3mm_Thk_ILM_IPL_um_S_Hemi_0minus3 | 22.101 | 1 | **<.001** |
| Macula_3mm_Thk_ILM_IPL_um_I_Hemi_0minus3 | 3.155 | 1 | .076 |
| Macula_3mm_Thk_ILM_IPL_um_All_0minus3 | 6.610 | 1 | **.010** |
| Macula_3mm_Thk_ILM_IPL_um_S_Hemi_field | .822 | 1 | .365 |
| Macula_3mm_Thk_ILM_IPL_um_I_Hemi_field | .071 | 1 | .790 |
| Macula_3mm_Thk_ILM_IPL_um_All_field | 20.916 | 1 | **<.001** |
| Macula_3mm_Thk_ILM_RPE_um_Center_1 | 2.534 | 1 | .111 |
| Macula_3mm_Thk_ILM_RPE_um_T_1minus3 | 9.236 | 1 | **.002** |
| Macula_3mm_Thk_ILM_RPE_um_S_1minus3 | 1.748 | 1 | .186 |
| Macula_3mm_Thk_ILM_RPE_um_N_1minus3 | 2.978 | 1 | .084 |
| Macula_3mm_Thk_ILM_RPE_um_I_1minus3 | 2.405 | 1 | .121 |
| Macula_3mm_Thk_ILM_RPE_um_S_Hemi_1minus3 | 7.169 | 1 | **.007** |
| Macula_3mm_Thk_ILM_RPE_um_I_Hemi_1minus3 | 1.415 | 1 | .234 |
| Macula_3mm_Thk_ILM_RPE_um_All_1minus3 | 5.103 | 1 | **.024** |
| Macula_3mm_Thk_ILM_RPE_um_S_Hemi_0minus3 | .612 | 1 | .434 |
| Macula_3mm_Thk_ILM_RPE_um_I_Hemi_0minus3 | 7.776 | 1 | **.005** |
| Macula_3mm_Thk_ILM_RPE_um_All_0minus3 | 8.682 | 1 | **.003** |
| Macula_3mm_Thk_ILM_RPE_um_S_Hemi_field | 14.341 | 1 | **<.001** |
| Macula_3mm_Thk_ILM_RPE_um_I_Hemi_field | 2.589 | 1 | .108 |
| Macula_3mm_Thk_ILM_RPE_um_All_field | 20.743 | 1 | **<.001** |
| Macula_3mm_Thk_ILM_BRM_um_Center_1 | 5.126 | 1 | **.024** |
| Macula_3mm_Thk_ILM_BRM_um_T_1minus3 | 5.781 | 1 | **.016** |
| Macula_3mm_Thk_ILM_BRM_um_S_1minus3 | .626 | 1 | .429 |
| Macula_3mm_Thk_ILM_BRM_um_N_1minus3 | 1.783 | 1 | .182 |
| Macula_3mm_Thk_ILM_BRM_um_I_1minus3 | 3.928 | 1 | **.047** |
| Macula_3mm_Thk_ILM_BRM_um_S_Hemi_1minus3 | 10.281 | 1 | **.001** |
| Macula_3mm_Thk_ILM_BRM_um_I_Hemi_1minus3 | 5.398 | 1 | **.020** |
| Macula_3mm_Thk_ILM_BRM_um_All_1minus3 | .672 | 1 | .412 |
| Macula_3mm_Thk_ILM_BRM_um_S_Hemi_0minus3 | .484 | 1 | .487 |
| Macula_3mm_Thk_ILM_BRM_um_I_Hemi_0minus3 | .802 | 1 | .371 |
| Macula_3mm_Thk_ILM_BRM_um_All_0minus3 | 2.558 | 1 | .110 |
| Macula_3mm_Thk_ILM_BRM_um_S_Hemi_field | 15.988 | 1 | **<.001** |
| Macula_3mm_Thk_ILM_BRM_um_I_Hemi_field | 4.484 | 1 | **.034** |
| Macula_3mm_Thk_ILM_BRM_um_All_field | 42.177 | 1 | **<.001** |
| Macula_3mm_Thk_RPE_BRM_um_Center_1 | 9.947 | 1 | **.002** |
| Macula_3mm_Thk_RPE_BRM_um_T_1minus3 | 6.265 | 1 | **.012** |
| Macula_3mm_Thk_RPE_BRM_um_S_1minus3 | 3.631 | 1 | .057 |
| Macula_3mm_Thk_RPE_BRM_um_N_1minus3 | .689 | 1 | .406 |
| Macula_3mm_Thk_RPE_BRM_um_I_1minus3 | 4.612 | 1 | **.032** |
| Macula_3mm_Thk_RPE_BRM_um_S_Hemi_1minus3 | 17.998 | 1 | **<.001** |
| Macula_3mm_Thk_RPE_BRM_um_I_Hemi_1minus3 | .061 | 1 | .805 |
| Macula_3mm_Thk_RPE_BRM_um_All_1minus3 | 18.189 | 1 | **<.001** |
| Macula_3mm_Thk_RPE_BRM_um_S_Hemi_0minus3 | 8.463 | 1 | **.004** |
| Macula_3mm_Thk_RPE_BRM_um_I_Hemi_0minus3 | .310 | 1 | .578 |
| Macula_3mm_Thk_RPE_BRM_um_All_0minus3 | .091 | 1 | .762 |
| Macula_3mm_Thk_RPE_BRM_um_S_Hemi_field | 19.762 | 1 | **<.001** |
| Macula_3mm_Thk_RPE_BRM_um_I_Hemi_field | 1.132 | 1 | .287 |
| Macula_3mm_Thk_RPE_BRM_um_All_field | 2.569 | 1 | .109 |
| Macula_3mm_Vol_ILM_IPL_mm3_Center_1 | .202 | 1 | .653 |
| Macula_3mm_Vol_ILM_IPL_mm3_T_1minus3 | .543 | 1 | .461 |
| Macula_3mm_Vol_ILM_IPL_mm3_S_1minus3 | 2.357 | 1 | .125 |
| Macula_3mm_Vol_ILM_IPL_mm3_N_1minus3 | 9.892 | 1 | **.002** |
| Macula_3mm_Vol_ILM_IPL_mm3_I_1minus3 | 2.395 | 1 | .122 |
| Macula_3mm_Vol_ILM_IPL_mm3_S_Hemi_1minus3 | .430 | 1 | .512 |
| Macula_3mm_Vol_ILM_IPL_mm3_I_Hemi_1minus3 | 13.905 | 1 | **<.001** |
| Macula_3mm_Vol_ILM_IPL_mm3_All_1minus3 | .473 | 1 | .491 |
| Macula_3mm_Vol_ILM_IPL_mm3_S_Hemi_0minus3 | .325 | 1 | .569 |
| Macula_3mm_Vol_ILM_IPL_mm3_I_Hemi_0minus3 | 1.257 | 1 | .262 |
| Macula_3mm_Vol_ILM_IPL_mm3_All_0minus3 | 17.444 | 1 | **<.001** |
| Macula_3mm_Vol_ILM_IPL_mm3_S_Hemi_field | 20.697 | 1 | **<.001** |
| Macula_3mm_Vol_ILM_IPL_mm3_I_Hemi_field | 8.898 | 1 | **.003** |
| Macula_3mm_Vol_ILM_IPL_mm3_All_field | 2.673 | 1 | .102 |
| Macula_3mm_Vol_ILM_RPE_mm3_Center_1 | 12.701 | 1 | **<.001** |
| Macula_3mm_Vol_ILM_RPE_mm3_T_1minus3 | .621 | 1 | .431 |
| Macula_3mm_Vol_ILM_RPE_mm3_S_1minus3 | 2.669 | 1 | .102 |
| Macula_3mm_Vol_ILM_RPE_mm3_N_1minus3 | 1.098 | 1 | .295 |
| Macula_3mm_Vol_ILM_RPE_mm3_I_1minus3 | 10.488 | 1 | **.001** |
| Macula_3mm_Vol_ILM_RPE_mm3_S_Hemi_1minus3 | 3.261 | 1 | .071 |
| Macula_3mm_Vol_ILM_RPE_mm3_I_Hemi_1minus3 | 16.909 | 1 | **<.001** |
| Macula_3mm_Vol_ILM_RPE_mm3_All_1minus3 | 2.190 | 1 | .139 |
| Macula_3mm_Vol_ILM_RPE_mm3_S_Hemi_0minus3 | 23.118 | 1 | **<.001** |
| Macula_3mm_Vol_ILM_RPE_mm3_I_Hemi_0minus3 | 15.379 | 1 | **<.001** |
| Macula_3mm_Vol_ILM_RPE_mm3_All_0minus3 | .663 | 1 | .416 |
| Macula_3mm_Vol_ILM_RPE_mm3_S_Hemi_field | 3.742 | 1 | .053 |
| Macula_3mm_Vol_ILM_RPE_mm3_I_Hemi_field | 1.155 | 1 | .283 |
| Macula_3mm_Vol_ILM_RPE_mm3_All_field | .238 | 1 | .626 |
| Macula_3mm_Vol_ILM_BRM_mm3_Center_1 | 36.039 | 1 | **<.001** |
| Macula_3mm_Vol_ILM_BRM_mm3_T_1minus3 | 1.024 | 1 | .311 |
| Macula_3mm_Vol_ILM_BRM_mm3_S_1minus3 | 1.533 | 1 | .216 |
| Macula_3mm_Vol_ILM_BRM_mm3_N_1minus3 | 3.492 | 1 | .062 |
| Macula_3mm_Vol_ILM_BRM_mm3_I_1minus3 | 5.112 | 1 | **.024** |
| Macula_3mm_Vol_ILM_BRM_mm3_S_Hemi_1minus3 | 10.596 | 1 | **.001** |
| Macula_3mm_Vol_ILM_BRM_mm3_I_Hemi_1minus3 | .717 | 1 | .397 |
| Macula_3mm_Vol_ILM_BRM_mm3_All_1minus3 | .263 | 1 | .608 |
| Macula_3mm_Vol_ILM_BRM_mm3_S_Hemi_0minus3 | 3.146 | 1 | .076 |
| Macula_3mm_Vol_ILM_BRM_mm3_I_Hemi_0minus3 | 8.142 | 1 | **.004** |
| Macula_3mm_Vol_ILM_BRM_mm3_All_0minus3 | .811 | 1 | .368 |
| Macula_3mm_Vol_ILM_BRM_mm3_S_Hemi_field | 4.637 | 1 | **.031** |
| Macula_3mm_Vol_ILM_BRM_mm3_I_Hemi_field | 1.990 | 1 | .158 |
| Macula_3mm_Vol_ILM_BRM_mm3_All_field | 9.494 | 1 | **.002** |
| Macula_3mm_Vol_RPE_BRM_mm3_Center_1 | 9.738 | 1 | **.002** |
| Macula_3mm_Vol_RPE_BRM_mm3_T_1minus3 | 2.780 | 1 | .095 |
| Macula_3mm_Vol_RPE_BRM_mm3_S_1minus3 | 3.713 | 1 | .054 |
| Macula_3mm_Vol_RPE_BRM_mm3_N_1minus3 | 1.033 | 1 | .309 |
| Macula_3mm_Vol_RPE_BRM_mm3_I_1minus3 | 7.376 | 1 | **.007** |
| Macula_3mm_Vol_RPE_BRM_mm3_S_Hemi_1minus3 | 3.225 | 1 | .073 |
| Macula_3mm_Vol_RPE_BRM_mm3_I_Hemi_1minus3 | 3.571 | 1 | .059 |
| Macula_3mm_Vol_RPE_BRM_mm3_All_1minus3 | 5.554 | 1 | **.018** |
| Macula_3mm_Vol_RPE_BRM_mm3_S_Hemi_0minus3 | 5.496 | 1 | **.019** |
| Macula_3mm_Vol_RPE_BRM_mm3_I_Hemi_0minus3 | 1.261 | 1 | .261 |
| Macula_3mm_Vol_RPE_BRM_mm3_All_0minus3 | 5.722 | 1 | **.017** |
| Macula_3mm_Vol_RPE_BRM_mm3_S_Hemi_field | 25.026 | 1 | **<.001** |
| Macula_3mm_Vol_RPE_BRM_mm3_I_Hemi_field | 7.363 | 1 | **.007** |
| Macula_3mm_Vol_RPE_BRM_mm3_All_field | 5.189 | 1 | .023 |
| Dependent Variable: Class_Num  Model: (Threshold), Macula_3mm_Thk_ILM_IPL_um_Center_1, Macula_3mm_Thk_ILM_IPL_um_T_1minus3, Macula_3mm_Thk_ILM_IPL_um_S_1minus3, Macula_3mm_Thk_ILM_IPL_um_N_1minus3, Macula_3mm_Thk_ILM_IPL_um_I_1minus3, Macula_3mm_Thk_ILM_IPL_um_S_Hemi_1minus3, Macula_3mm_Thk_ILM_IPL_um_I_Hemi_1minus3, Macula_3mm_Thk_ILM_IPL_um_All_1minus3, Macula_3mm_Thk_ILM_IPL_um_S_Hemi_0minus3, Macula_3mm_Thk_ILM_IPL_um_I_Hemi_0minus3, Macula_3mm_Thk_ILM_IPL_um_All_0minus3, Macula_3mm_Thk_ILM_IPL_um_S_Hemi_field, Macula_3mm_Thk_ILM_IPL_um_I_Hemi_field, Macula_3mm_Thk_ILM_IPL_um_All_field, Macula_3mm_Thk_ILM_RPE_um_Center_1, Macula_3mm_Thk_ILM_RPE_um_T_1minus3, Macula_3mm_Thk_ILM_RPE_um_S_1minus3, Macula_3mm_Thk_ILM_RPE_um_N_1minus3, Macula_3mm_Thk_ILM_RPE_um_I_1minus3, Macula_3mm_Thk_ILM_RPE_um_S_Hemi_1minus3, Macula_3mm_Thk_ILM_RPE_um_I_Hemi_1minus3, Macula_3mm_Thk_ILM_RPE_um_All_1minus3, Macula_3mm_Thk_ILM_RPE_um_S_Hemi_0minus3, Macula_3mm_Thk_ILM_RPE_um_I_Hemi_0minus3, Macula_3mm_Thk_ILM_RPE_um_All_0minus3, Macula_3mm_Thk_ILM_RPE_um_S_Hemi_field, Macula_3mm_Thk_ILM_RPE_um_I_Hemi_field, Macula_3mm_Thk_ILM_RPE_um_All_field, Macula_3mm_Thk_ILM_BRM_um_Center_1, Macula_3mm_Thk_ILM_BRM_um_T_1minus3, Macula_3mm_Thk_ILM_BRM_um_S_1minus3, Macula_3mm_Thk_ILM_BRM_um_N_1minus3, Macula_3mm_Thk_ILM_BRM_um_I_1minus3, Macula_3mm_Thk_ILM_BRM_um_S_Hemi_1minus3, Macula_3mm_Thk_ILM_BRM_um_I_Hemi_1minus3, Macula_3mm_Thk_ILM_BRM_um_All_1minus3, Macula_3mm_Thk_ILM_BRM_um_S_Hemi_0minus3, Macula_3mm_Thk_ILM_BRM_um_I_Hemi_0minus3, Macula_3mm_Thk_ILM_BRM_um_All_0minus3, Macula_3mm_Thk_ILM_BRM_um_S_Hemi_field, Macula_3mm_Thk_ILM_BRM_um_I_Hemi_field, Macula_3mm_Thk_ILM_BRM_um_All_field, Macula_3mm_Thk_RPE_BRM_um_Center_1, Macula_3mm_Thk_RPE_BRM_um_T_1minus3, Macula_3mm_Thk_RPE_BRM_um_S_1minus3, Macula_3mm_Thk_RPE_BRM_um_N_1minus3, Macula_3mm_Thk_RPE_BRM_um_I_1minus3, Macula_3mm_Thk_RPE_BRM_um_S_Hemi_1minus3, Macula_3mm_Thk_RPE_BRM_um_I_Hemi_1minus3, Macula_3mm_Thk_RPE_BRM_um_All_1minus3, Macula_3mm_Thk_RPE_BRM_um_S_Hemi_0minus3, Macula_3mm_Thk_RPE_BRM_um_I_Hemi_0minus3, Macula_3mm_Thk_RPE_BRM_um_All_0minus3, Macula_3mm_Thk_RPE_BRM_um_S_Hemi_field, Macula_3mm_Thk_RPE_BRM_um_I_Hemi_field, Macula_3mm_Thk_RPE_BRM_um_All_field, Macula_3mm_Vol_ILM_IPL_mm3_Center_1, Macula_3mm_Vol_ILM_IPL_mm3_T_1minus3, Macula_3mm_Vol_ILM_IPL_mm3_S_1minus3, Macula_3mm_Vol_ILM_IPL_mm3_N_1minus3, Macula_3mm_Vol_ILM_IPL_mm3_I_1minus3, Macula_3mm_Vol_ILM_IPL_mm3_S_Hemi_1minus3, Macula_3mm_Vol_ILM_IPL_mm3_I_Hemi_1minus3, Macula_3mm_Vol_ILM_IPL_mm3_All_1minus3, Macula_3mm_Vol_ILM_IPL_mm3_S_Hemi_0minus3, Macula_3mm_Vol_ILM_IPL_mm3_I_Hemi_0minus3, Macula_3mm_Vol_ILM_IPL_mm3_All_0minus3, Macula_3mm_Vol_ILM_IPL_mm3_S_Hemi_field, Macula_3mm_Vol_ILM_IPL_mm3_I_Hemi_field, Macula_3mm_Vol_ILM_IPL_mm3_All_field, Macula_3mm_Vol_ILM_RPE_mm3_Center_1, Macula_3mm_Vol_ILM_RPE_mm3_T_1minus3, Macula_3mm_Vol_ILM_RPE_mm3_S_1minus3, Macula_3mm_Vol_ILM_RPE_mm3_N_1minus3, Macula_3mm_Vol_ILM_RPE_mm3_I_1minus3, Macula_3mm_Vol_ILM_RPE_mm3_S_Hemi_1minus3, Macula_3mm_Vol_ILM_RPE_mm3_I_Hemi_1minus3, Macula_3mm_Vol_ILM_RPE_mm3_All_1minus3, Macula_3mm_Vol_ILM_RPE_mm3_S_Hemi_0minus3, Macula_3mm_Vol_ILM_RPE_mm3_I_Hemi_0minus3, Macula_3mm_Vol_ILM_RPE_mm3_All_0minus3, Macula_3mm_Vol_ILM_RPE_mm3_S_Hemi_field, Macula_3mm_Vol_ILM_RPE_mm3_I_Hemi_field, Macula_3mm_Vol_ILM_RPE_mm3_All_field, Macula_3mm_Vol_ILM_BRM_mm3_Center_1, Macula_3mm_Vol_ILM_BRM_mm3_T_1minus3, Macula_3mm_Vol_ILM_BRM_mm3_S_1minus3, Macula_3mm_Vol_ILM_BRM_mm3_N_1minus3, Macula_3mm_Vol_ILM_BRM_mm3_I_1minus3, Macula_3mm_Vol_ILM_BRM_mm3_S_Hemi_1minus3, Macula_3mm_Vol_ILM_BRM_mm3_I_Hemi_1minus3, Macula_3mm_Vol_ILM_BRM_mm3_All_1minus3, Macula_3mm_Vol_ILM_BRM_mm3_S_Hemi_0minus3, Macula_3mm_Vol_ILM_BRM_mm3_I_Hemi_0minus3, Macula_3mm_Vol_ILM_BRM_mm3_All_0minus3, Macula_3mm_Vol_ILM_BRM_mm3_S_Hemi_field, Macula_3mm_Vol_ILM_BRM_mm3_I_Hemi_field, Macula_3mm_Vol_ILM_BRM_mm3_All_field, Macula_3mm_Vol_RPE_BRM_mm3_Center_1, Macula_3mm_Vol_RPE_BRM_mm3_T_1minus3, Macula_3mm_Vol_RPE_BRM_mm3_S_1minus3, Macula_3mm_Vol_RPE_BRM_mm3_N_1minus3, Macula_3mm_Vol_RPE_BRM_mm3_I_1minus3, Macula_3mm_Vol_RPE_BRM_mm3_S_Hemi_1minus3, Macula_3mm_Vol_RPE_BRM_mm3_I_Hemi_1minus3, Macula_3mm_Vol_RPE_BRM_mm3_All_1minus3, Macula_3mm_Vol_RPE_BRM_mm3_S_Hemi_0minus3, Macula_3mm_Vol_RPE_BRM_mm3_I_Hemi_0minus3, Macula_3mm_Vol_RPE_BRM_mm3_All_0minus3, Macula_3mm_Vol_RPE_BRM_mm3_S_Hemi_field, Macula_3mm_Vol_RPE_BRM_mm3_I_Hemi_field, Macula_3mm_Vol_RPE_BRM_mm3_All_field, offset = AgePerToday | | | |

### Using Retina3DflowDensity parameters

| **Tests of Model Effects** | | | |
| --- | --- | --- | --- |
| Source | Type III | | |
|  | Wald Chi-Square | df | Sig. |
| SVC_L1_DensityOfWhole_Image | 11.105 | 1 | **<.001** |
| SVC_L1_Whole_Image_S_Hemi | 5.553 | 1 | **.018** |
| SVC_L1_Whole_Image_I_Hemi | 4.949 | 1 | **.026** |
| SVC_L1_Whole_ETDRS | 4.608 | 1 | **.032** |
| SVC_L1_Fovea | 5.526 | 1 | **.019** |
| SVC_L1_ParaFovea | .025 | 1 | .875 |
| SVC_L1_Para_S_Hemi | .497 | 1 | .481 |
| SVC_L1_Para_I_Hemi | .516 | 1 | .473 |
| SVC_L1_Para_T | 7.282 | 1 | **.007** |
| SVC_L1_Para_S | 5.632 | 1 | **.018** |
| SVC_L1_Para_N | 7.236 | 1 | **.007** |
| SVC_L1_Para_I | 6.932 | 1 | **.008** |
| SVC_L1_G11 | 7.299 | 1 | **.007** |
| SVC_L1_G12 | 6.986 | 1 | **.008** |
| SVC_L1_G13 | 7.780 | 1 | **.005** |
| SVC_L1_G21 | 7.721 | 1 | **.005** |
| SVC_L1_G22 | 8.967 | 1 | **.003** |
| SVC_L1_G23 | 8.399 | 1 | **.004** |
| SVC_L1_G31 | 7.582 | 1 | **.006** |
| SVC_L1_G32 | 7.283 | 1 | **.007** |
| SVC_L1_G33 | 7.318 | 1 | **.007** |
| DVC_L2_DensityOfWhole_Image | 24.056 | 1 | **<.001** |
| DVC_L2_Whole_Image_S_Hemi | 22.625 | 1 | **<.001** |
| DVC_L2_Whole_Image_I_Hemi | 15.437 | 1 | **<.001** |
| DVC_L2_Whole_ETDRS | .544 | 1 | .461 |
| DVC_L2_Fovea | .460 | 1 | .498 |
| DVC_L2_ParaFovea | 7.354 | 1 | **.007** |
| DVC_L2_Para_S_Hemi | 11.340 | 1 | **<.001** |
| DVC_L2_Para_I_Hemi | 10.486 | 1 | **.001** |
| DVC_L2_Para_T | 5.675 | 1 | **.017** |
| DVC_L2_Para_S | 4.570 | 1 | **.033** |
| DVC_L2_Para_N | 5.425 | 1 | **.020** |
| DVC_L2_Para_I | 4.608 | 1 | **.032** |
| DVC_L2_G11 | 27.997 | 1 | **<.001** |
| DVC_L2_G12 | 28.897 | 1 | **<.001** |
| DVC_L2_G13 | 28.946 | 1 | **<.001** |
| DVC_L2_G21 | 27.402 | 1 | **<.001** |
| DVC_L2_G22 | 28.359 | 1 | **<.001** |
| DVC_L2_G23 | 27.578 | 1 | **<.001** |
| DVC_L2_G31 | 28.753 | 1 | **<.001** |
| DVC_L2_G32 | 28.459 | 1 | **<.001** |
| DVC_L2_G33 | 28.231 | 1 | **<.001** |
| FAZ_Area | 2.311 | 1 | .128 |
| Perimeter | 12.498 | 1 | **<.001** |
| AcircularityIndex | 6.679 | 1 | **.010** |
| FD_300_Area_Density | 6.387 | 1 | **.011** |
| FD_300_Length_Density | 32.070 | 1 | **<.001** |
| Dependent Variable: Class_Num  Model: (Threshold), SVC_L1_DensityOfWhole_Image, SVC_L1_Whole_Image_S_Hemi, SVC_L1_Whole_Image_I_Hemi, SVC_L1_Whole_ETDRS, SVC_L1_Fovea, SVC_L1_ParaFovea, SVC_L1_Para_S_Hemi, SVC_L1_Para_I_Hemi, SVC_L1_Para_T, SVC_L1_Para_S, SVC_L1_Para_N, SVC_L1_Para_I, SVC_L1_G11, SVC_L1_G12, SVC_L1_G13, SVC_L1_G21, SVC_L1_G22, SVC_L1_G23, SVC_L1_G31, SVC_L1_G32, SVC_L1_G33, DVC_L2_DensityOfWhole_Image, DVC_L2_Whole_Image_S_Hemi, DVC_L2_Whole_Image_I_Hemi, DVC_L2_Whole_ETDRS, DVC_L2_Fovea, DVC_L2_ParaFovea, DVC_L2_Para_S_Hemi, DVC_L2_Para_I_Hemi, DVC_L2_Para_T, DVC_L2_Para_S, DVC_L2_Para_N, DVC_L2_Para_I, DVC_L2_G11, DVC_L2_G12, DVC_L2_G13, DVC_L2_G21, DVC_L2_G22, DVC_L2_G23, DVC_L2_G31, DVC_L2_G32, DVC_L2_G33, FAZ_Area, Perimeter, AcircularityIndex, FD_300_Area_Density, FD_300_Length_Density, offset = AgePerToday | | | |

### Using Retina Map - Inner Retina Thickness parameters

| **Tests of Model Effects** | | | |
| --- | --- | --- | --- |
| Source | Type III | | |
|  | Wald Chi-Square | df | Sig. |
| FoveaInnRet_Thickness | 7.445 | 1 | **.006** |
| InnRet_Thk_ParaFovea | 15.289 | 1 | **<.001** |
| InnRet_Thk_Para_S_Hemisphere | 9.620 | 1 | **.002** |
| InnRet_Thk_Para_I_Hemisphere | .007 | 1 | .931 |
| InnRet_Thk_Para_Tempo | 13.875 | 1 | **<.001** |
| InnRet_Thk_Para_Superior | .055 | 1 | .815 |
| InnRet_Thk_Para_Nasal | 4.346 | 1 | **.037** |
| InnRet_Thk_Para_Inferior | 8.735 | 1 | **.003** |
| InnRet_Thk_PeriFovea | 3.157 | 1 | .076 |
| InnRet_Thk_Peri_S_Hemisphere | .706 | 1 | .401 |
| InnRet_Thk_Peri_I_Hemisphere | 3.482 | 1 | .062 |
| InnRet_Thk_Peri_Tempo | .443 | 1 | .506 |
| InnRet_Thk_Peri_Superior | .046 | 1 | .830 |
| InnRet_Thk_Peri_Nasal | 1.477 | 1 | .224 |
| InnRet_Thk_Peri_Inferior | .046 | 1 | .830 |
| Dependent Variable: Class_Num  Model: (Threshold), FoveaInnRet_Thickness, InnRet_Thk_ParaFovea, InnRet_Thk_Para_S_Hemisphere, InnRet_Thk_Para_I_Hemisphere, InnRet_Thk_Para_Tempo, InnRet_Thk_Para_Superior, InnRet_Thk_Para_Nasal, InnRet_Thk_Para_Inferior, InnRet_Thk_PeriFovea, InnRet_Thk_Peri_S_Hemisphere, InnRet_Thk_Peri_I_Hemisphere, InnRet_Thk_Peri_Tempo, InnRet_Thk_Peri_Superior, InnRet_Thk_Peri_Nasal, InnRet_Thk_Peri_Inferior, offset = AgePerToday | | | |

### Using Retina Map - Inner Retina Volume parameters

| **Tests of Model Effects** | | | |
| --- | --- | --- | --- |
| Source | Type III | | |
|  | Wald Chi-Square | df | Sig. |
| FoveaInnRet_Volumn | 4.880 | 1 | **.027** |
| InnRet_Vol_ParaFovea | 10.020 | 1 | **.002** |
| InnRet_Vol_Para_S_Hemisphere | 11.015 | 1 | **<.001** |
| InnRet_Vol_Para_I_Hemisphere | 13.614 | 1 | **<.001** |
| InnRet_Vol_Para_Tempo | 12.019 | 1 | **<.001** |
| InnRet_Vol_Para_Superior | 2.549 | 1 | .110 |
| InnRet_Vol_Para_Nasal | 4.885 | 1 | **.027** |
| InnRet_Vol_Para_Inferior | 2.564 | 1 | .109 |
| InnRet_Vol_PeriFovea | 4.238 | 1 | **.040** |
| InnRet_Vol_Peri_S_Hemisphere | 5.010 | 1 | **.025** |
| InnRet_Vol_Peri_I_Hemisphere | 7.588 | 1 | **.006** |
| InnRet_Vol_Peri_Tempo | 14.408 | 1 | **<.001** |
| InnRet_Vol_Peri_Superior | 11.554 | 1 | **<.001** |
| InnRet_Vol_Peri_Nasal | 12.539 | 1 | **<.001** |
| InnRet_Vol_Peri_Inferior | 13.444 | 1 | **<.001** |
| Dependent Variable: Class_Num  Model: (Threshold), FoveaInnRet_Volumn, InnRet_Vol_ParaFovea, InnRet_Vol_Para_S_Hemisphere, InnRet_Vol_Para_I_Hemisphere, InnRet_Vol_Para_Tempo, InnRet_Vol_Para_Superior, InnRet_Vol_Para_Nasal, InnRet_Vol_Para_Inferior, InnRet_Vol_PeriFovea, InnRet_Vol_Peri_S_Hemisphere, InnRet_Vol_Peri_I_Hemisphere, InnRet_Vol_Peri_Tempo, InnRet_Vol_Peri_Superior, InnRet_Vol_Peri_Nasal, InnRet_Vol_Peri_Inferior, offset = AgePerToday | | | |

### Using Retina Map - Full Retina Thickness/Volume and RPE parameters

| **Tests of Model Effects** | | | |
| --- | --- | --- | --- |
| Source | Type III | | |
|  | Wald Chi-Square | df | Sig. |
| FoveaFullRet_Thickness | 5.106 | 1 | **.024** |
| FullRet_Thk_ParaFovea | 18.870 | 1 | **<.001** |
| FullRet_Thk_Para_S_Hemisphere | .224 | 1 | .636 |
| FullRet_Thk_Para_I_Hemisphere | 22.503 | 1 | **<.001** |
| FullRet_Thk_Para_Tempo | 3.067 | 1 | .080 |
| FullRet_Thk_Para_Superior | 2.657 | 1 | .103 |
| FullRet_Thk_Para_Nasal | 1.046 | 1 | .306 |
| FullRet_Thk_Para_Inferior | 6.733 | 1 | **.009** |
| FullRet_Thk_PeriFovea | 5.890 | 1 | **.015** |
| FullRet_Thk_Peri_S_Hemisphere | 28.081 | 1 | **<.001** |
| FullRet_Thk_Peri_I_Hemisphere | 4.417 | 1 | **.036** |
| FullRet_Thk_Peri_Tempo | 10.963 | 1 | **<.001** |
| FullRet_Thk_Peri_Superior | 14.420 | 1 | **<.001** |
| FullRet_Thk_Peri_Nasal | 3.009 | 1 | .083 |
| FullRet_Thk_Peri_Inferior | 3.294 | 1 | .070 |
| FoveaFullRet_Volumn | 4.563 | 1 | **.033** |
| FullRet_Vol_ParaFovea | 2.721 | 1 | .099 |
| FullRet_Vol_Para_S_Hemisphere | 23.094 | 1 | **<.001** |
| FullRet_Vol_Para_I_Hemisphere | 20.217 | 1 | **<.001** |
| FullRet_Vol_Para_Tempo | 18.182 | 1 | **<.001** |
| FullRet_Vol_Para_Superior | 14.778 | 1 | **<.001** |
| FullRet_Vol_Para_Nasal | 8.054 | 1 | **.005** |
| FullRet_Vol_Para_Inferior | 5.583 | 1 | **.018** |
| FullRet_Vol_PeriFovea | 6.454 | 1 | **.011** |
| FullRet_Vol_Peri_S_Hemisphere | 1.128 | 1 | .288 |
| FullRet_Vol_Peri_I_Hemisphere | 1.280 | 1 | .258 |
| FullRet_Vol_Peri_Tempo | 3.734 | 1 | .053 |
| FullRet_Vol_Peri_Superior | 3.257 | 1 | .071 |
| FullRet_Vol_Peri_Nasal | 4.392 | 1 | **.036** |
| FullRet_Vol_Peri_Inferior | 2.748 | 1 | .097 |
| FoveaRPE_Elevation_Height | .099 | 1 | .753 |
| RPE_Elev_ParaFovea_Tempo | 15.495 | 1 | **<.001** |
| RPE_Elev_Para_Superior | 2.653 | 1 | .103 |
| RPE_Elev_Para_Nasal | .017 | 1 | .895 |
| RPE_Elev_Para_Inferior | 30.837 | 1 | **<.001** |
| RPE_Elev_PeriFovea_Tempo | 9.959 | 1 | **.002** |
| RPE_Elev_Peri_Superior | 23.167 | 1 | **<.001** |
| RPE_Elev_Peri_Nasal | 1.243 | 1 | .265 |
| RPE_Elev_Peri_Inferior | 10.871 | 1 | **<.001** |
| Dependent Variable: Class_Num  Model: (Threshold), FoveaFullRet_Thickness, FullRet_Thk_ParaFovea, FullRet_Thk_Para_S_Hemisphere, FullRet_Thk_Para_I_Hemisphere, FullRet_Thk_Para_Tempo, FullRet_Thk_Para_Superior, FullRet_Thk_Para_Nasal, FullRet_Thk_Para_Inferior, FullRet_Thk_PeriFovea, FullRet_Thk_Peri_S_Hemisphere, FullRet_Thk_Peri_I_Hemisphere, FullRet_Thk_Peri_Tempo, FullRet_Thk_Peri_Superior, FullRet_Thk_Peri_Nasal, FullRet_Thk_Peri_Inferior, FoveaFullRet_Volumn, FullRet_Vol_ParaFovea, FullRet_Vol_Para_S_Hemisphere, FullRet_Vol_Para_I_Hemisphere, FullRet_Vol_Para_Tempo, FullRet_Vol_Para_Superior, FullRet_Vol_Para_Nasal, FullRet_Vol_Para_Inferior, FullRet_Vol_PeriFovea, FullRet_Vol_Peri_S_Hemisphere, FullRet_Vol_Peri_I_Hemisphere, FullRet_Vol_Peri_Tempo, FullRet_Vol_Peri_Superior, FullRet_Vol_Peri_Nasal, FullRet_Vol_Peri_Inferior, FoveaRPE_Elevation_Height, RPE_Elev_ParaFovea_Tempo, RPE_Elev_Para_Superior, RPE_Elev_Para_Nasal, RPE_Elev_Para_Inferior, RPE_Elev_PeriFovea_Tempo, RPE_Elev_Peri_Superior, RPE_Elev_Peri_Nasal, RPE_Elev_Peri_Inferior, offset = AgePerToday | | | |

## Parameter Estimates

### Using GCC parameters

| **Parameter Estimates** | | | | | | | | |
| --- | --- | --- | --- | --- | --- | --- | --- | --- |
| Parameter | | B | Std. Error | 95% Wald Confidence Interval | | Hypothesis Test | | |
|  |  |  |  | Lower | Upper | Wald Chi-Square | df | Sig. |
| Threshold | [Class_Num=0] | -100.000 | 5.5364 | -110.851 | -89.148 | 326.241 | 1 | .000 |
|  | [Class_Num=1] | -96.504 | 5.5324 | -107.347 | -85.661 | 304.270 | 1 | .000 |
| GCC_Inner_Retina_Average | | -8.739 | 7.1376 | -22.728 | 5.251 | 1.499 | 1 | .221 |
| GCC_Superior_Avg | | 3.012 | 2.6882 | -2.256 | 8.281 | 1.256 | 1 | .262 |
| GCC_Inferior_Avg | | 1.081 | 1.3852 | -1.634 | 3.796 | .609 | 1 | .435 |
| GCC_S_I_Avg | | -1.013 | .8472 | -2.674 | .647 | 1.431 | 1 | .232 |
| GCC_FLV | | -.031 | .1671 | -.359 | .296 | .035 | 1 | .852 |
| GCC_GLV | | .001 | .0874 | -.170 | .173 | .000 | 1 | .987 |
| GCC_RMS | | 8.143 | 6.5671 | -4.728 | 21.015 | 1.538 | 1 | .215 |
| GCC_Full_Retina_Average | | 4.528 | 3.9835 | -3.279 | 12.336 | 1.292 | 1 | .256 |
| GCC_Superior_Avg.1 | | -.048 | .1241 | -.291 | .195 | .149 | 1 | .699 |
| GCC_Inferior_Avg.1 | | .099 | .1861 | -.265 | .464 | .286 | 1 | .593 |
| GCC_S_I_Avg.1 | | -.040 | .1918 | -.416 | .336 | .044 | 1 | .834 |
| GCC_Outer_Retina_Average | | -4.582 | 3.9829 | -12.389 | 3.224 | 1.324 | 1 | .250 |
| GCC_Superior_Avg.2 | | .081 | .2583 | -.425 | .587 | .098 | 1 | .754 |
| GCC_Inferior_Avg.2 | | -.137 | .1280 | -.387 | .114 | 1.140 | 1 | .286 |
| GCC_S_I_Avg.2 | | .165 | .4252 | -.668 | .999 | .151 | 1 | .697 |
| (Scale) | | 1^a^ |  |  |  |  |  |  |
| Dependent Variable: Class_Num  Model: (Threshold), GCC_Inner_Retina_Average, GCC_Superior_Avg, GCC_Inferior_Avg, GCC_S_I_Avg, GCC_FLV, GCC_GLV, GCC_RMS, GCC_Full_Retina_Average, GCC_Superior_Avg.1, GCC_Inferior_Avg.1, GCC_S_I_Avg.1, GCC_Outer_Retina_Average, GCC_Superior_Avg.2, GCC_Inferior_Avg.2, GCC_S_I_Avg.2, offset = AgePerToday | | | | | | | | |
| a. Fixed at the displayed value. | | | | | | | | |

### Using ONH parameters

| **Parameter Estimates** | | | | | | | | |
| --- | --- | --- | --- | --- | --- | --- | --- | --- |
| Parameter | | B | Std. Error | 95% Wald Confidence Interval | | Hypothesis Test | | |
|  |  |  |  | Lower | Upper | Wald Chi-Square | df | Sig. |
| Threshold | [Class_Num=0] | -82.679 | 2.8566 | -88.278 | -77.080 | 837.699 | 1 | .000 |
|  | [Class_Num=1] | -79.272 | 2.8765 | -84.909 | -73.634 | 759.462 | 1 | .000 |
| ONH_DiscArea | | -4.811 | 1.9426 | -8.619 | -1.004 | 6.134 | 1 | **.013** |
| ONH_Area_C_D_ratio | | 33.277 | 6.9598 | 19.636 | 46.918 | 22.861 | 1 | **<.001** |
| ONH_H_C_D_ratio | | -12.108 | 2.7238 | -17.447 | -6.770 | 19.762 | 1 | **<.001** |
| ONH_V_C_D_ratio | | -15.093 | 2.6216 | -20.232 | -9.955 | 33.148 | 1 | **<.001** |
| ONH_CupArea | | 8.054 | 2.1943 | 3.754 | 12.355 | 13.473 | 1 | **<.001** |
| ONH_RimArea | | 6.982 | 2.3479 | 2.380 | 11.584 | 8.843 | 1 | **.003** |
| ONH_RimVolume | | -19.114 | 7.6329 | -34.074 | -4.154 | 6.271 | 1 | **.012** |
| ONH_Disc_Volume | | 4.137 | 4.2498 | -4.193 | 12.466 | .948 | 1 | .330 |
| ONH_CupVolume | | -14.890 | 2.4238 | -19.641 | -10.140 | 37.742 | 1 | **<.001** |
| ONH_Avg_RNFL | | .203 | .1026 | .002 | .404 | 3.921 | 1 | **.048** |
| ONH_Sup_RNFL | | .215 | .1404 | -.060 | .490 | 2.346 | 1 | .126 |
| ONH_Inf_RNFL | | .278 | .1299 | .023 | .532 | 4.565 | 1 | **.033** |
| ONH_Tempo | | .486 | .1036 | .283 | .689 | 22.020 | 1 | **<.001** |
| ONH_Superior | | -.115 | .0719 | -.256 | .025 | 2.581 | 1 | .108 |
| ONH_Nasal | | .837 | .2470 | .353 | 1.321 | 11.487 | 1 | **<.001** |
| ONH_Inferior | | .148 | .0751 | .001 | .295 | 3.878 | 1 | **.049** |
| ONH_RNFL_TU | | -.111 | .0453 | -.199 | -.022 | 5.947 | 1 | **.015** |
| ONH_ST | | -.035 | .0671 | -.166 | .097 | .265 | 1 | .607 |
| ONH_SN | | .025 | .0835 | -.139 | .189 | .089 | 1 | .766 |
| ONH_NU | | -.480 | .1258 | -.727 | -.234 | 14.580 | 1 | **<.001** |
| ONH_NL | | -3.661 | .6257 | -4.887 | -2.435 | 34.231 | 1 | **<.001** |
| ONH_IN | | .062 | .0720 | -.079 | .203 | .735 | 1 | .391 |
| ONH_IT | | -.308 | .0693 | -.444 | -.172 | 19.772 | 1 | **<.001** |
| ONH_TL | | -.719 | .1068 | -.928 | -.510 | 45.351 | 1 | **<.001** |
| ONH_TU1 | | -.140 | .0482 | -.234 | -.046 | 8.447 | 1 | **.004** |
| ONH_TU2 | | .008 | .0314 | -.053 | .070 | .073 | 1 | .787 |
| ONH_ST2 | | -.019 | .0221 | -.062 | .025 | .727 | 1 | .394 |
| ONH_ST1 | | -.079 | .0186 | -.115 | -.042 | 18.069 | 1 | **<.001** |
| ONH_SN1 | | .100 | .0281 | .045 | .155 | 12.638 | 1 | **<.001** |
| ONH_SN2 | | -.008 | .0301 | -.067 | .051 | .076 | 1 | .783 |
| ONH_NU2 | | -.055 | .0632 | -.179 | .069 | .757 | 1 | .384 |
| ONH_NU1 | | -.003 | .0747 | -.149 | .144 | .001 | 1 | .970 |
| ONH_NL1 | | 1.591 | .2816 | 1.039 | 2.142 | 31.911 | 1 | **<.001** |
| ONH_NL2 | | 1.583 | .2755 | 1.043 | 2.123 | 33.025 | 1 | **<.001** |
| ONH_IN2 | | -.110 | .0465 | -.201 | -.019 | 5.637 | 1 | **.018** |
| ONH_IN1 | | -.116 | .0454 | -.205 | -.027 | 6.526 | 1 | **.011** |
| ONH_IT1 | | .036 | .0294 | -.021 | .094 | 1.537 | 1 | .215 |
| ONH_IT2 | | .030 | .0285 | -.026 | .085 | 1.087 | 1 | .297 |
| ONH_TL2 | | .265 | .0580 | .151 | .378 | 20.846 | 1 | **<.001** |
| ONH_TL1 | | .100 | .0640 | -.026 | .225 | 2.420 | 1 | .120 |
| (Scale) | | 1^a^ |  |  |  |  |  |  |
| Dependent Variable: Class_Num  Model: (Threshold), ONH_DiscArea, ONH_Area_C_D_ratio, ONH_H_C_D_ratio, ONH_V_C_D_ratio, ONH_CupArea, ONH_RimArea, ONH_RimVolume, ONH_Disc_Volume, ONH_CupVolume, ONH_Avg_RNFL, ONH_Sup_RNFL, ONH_Inf_RNFL, ONH_Tempo, ONH_Superior, ONH_Nasal, ONH_Inferior, ONH_RNFL_TU, ONH_ST, ONH_SN, ONH_NU, ONH_NL, ONH_IN, ONH_IT, ONH_TL, ONH_TU1, ONH_TU2, ONH_ST2, ONH_ST1, ONH_SN1, ONH_SN2, ONH_NU2, ONH_NU1, ONH_NL1, ONH_NL2, ONH_IN2, ONH_IN1, ONH_IT1, ONH_IT2, ONH_TL2, ONH_TL1, offset = AgePerToday | | | | | | | | |
| a. Fixed at the displayed value. | | | | | | | | |

### Using Macula_3mm parameters

| **Parameter Estimates** | | | | | | | | |
| --- | --- | --- | --- | --- | --- | --- | --- | --- |
| Parameter | | B | Std. Error | 95% Wald Confidence Interval | | Hypothesis Test | | |
|  |  |  |  | Lower | Upper | Wald Chi-Square | df | Sig. |
| Threshold | [Class_Num=0] | -104.446 | 5.7149 | -115.648 | -93.245 | 334.012 | 1 | .000 |
|  | [Class_Num=1] | -100.167 | 5.6859 | -111.311 | -89.022 | 310.343 | 1 | .000 |
| Macula_3mm_Thk_ILM_IPL_um_Center_1 | | -2.629 | 1.5381 | -5.644 | .386 | 2.922 | 1 | .087 |
| Macula_3mm_Thk_ILM_IPL_um_T_1minus3 | | 2.001 | 2.4386 | -2.779 | 6.780 | .673 | 1 | .412 |
| Macula_3mm_Thk_ILM_IPL_um_S_1minus3 | | 1.706 | 2.2349 | -2.674 | 6.086 | .583 | 1 | .445 |
| Macula_3mm_Thk_ILM_IPL_um_N_1minus3 | | -1.342 | 1.8848 | -5.036 | 2.353 | .507 | 1 | .477 |
| Macula_3mm_Thk_ILM_IPL_um_I_1minus3 | | -1.610 | 2.4094 | -6.332 | 3.113 | .446 | 1 | .504 |
| Macula_3mm_Thk_ILM_IPL_um_S_Hemi_1minus3 | | -42.397 | 5.4435 | -53.066 | -31.728 | 60.661 | 1 | **<.001** |
| Macula_3mm_Thk_ILM_IPL_um_I_Hemi_1minus3 | | -5.266 | 6.7728 | -18.541 | 8.008 | .605 | 1 | .437 |
| Macula_3mm_Thk_ILM_IPL_um_All_1minus3 | | 13.487 | 6.8886 | -.015 | 26.988 | 3.833 | 1 | .050 |
| Macula_3mm_Thk_ILM_IPL_um_S_Hemi_0minus3 | | 30.910 | 6.5749 | 18.023 | 43.797 | 22.101 | 1 | **<.001** |
| Macula_3mm_Thk_ILM_IPL_um_I_Hemi_0minus3 | | -12.067 | 6.7933 | -25.382 | 1.248 | 3.155 | 1 | .076 |
| Macula_3mm_Thk_ILM_IPL_um_All_0minus3 | | 17.456 | 6.7896 | 4.148 | 30.763 | 6.610 | 1 | **.010** |
| Macula_3mm_Thk_ILM_IPL_um_S_Hemi_field | | 2.178 | 2.4032 | -2.532 | 6.888 | .822 | 1 | .365 |
| Macula_3mm_Thk_ILM_IPL_um_I_Hemi_field | | .701 | 2.6290 | -4.451 | 5.854 | .071 | 1 | .790 |
| Macula_3mm_Thk_ILM_IPL_um_All_field | | -31.385 | 6.8625 | -44.835 | -17.935 | 20.916 | 1 | **<.001** |
| Macula_3mm_Thk_ILM_RPE_um_Center_1 | | -7.687 | 4.8292 | -17.152 | 1.778 | 2.534 | 1 | .111 |
| Macula_3mm_Thk_ILM_RPE_um_T_1minus3 | | -15.963 | 5.2526 | -26.258 | -5.668 | 9.236 | 1 | **.002** |
| Macula_3mm_Thk_ILM_RPE_um_S_1minus3 | | -7.652 | 5.7878 | -18.996 | 3.692 | 1.748 | 1 | .186 |
| Macula_3mm_Thk_ILM_RPE_um_N_1minus3 | | -8.496 | 4.9231 | -18.145 | 1.153 | 2.978 | 1 | .084 |
| Macula_3mm_Thk_ILM_RPE_um_I_1minus3 | | 8.463 | 5.4571 | -2.233 | 19.159 | 2.405 | 1 | .121 |
| Macula_3mm_Thk_ILM_RPE_um_S_Hemi_1minus3 | | 16.504 | 6.1642 | 4.423 | 28.586 | 7.169 | 1 | .007 |
| Macula_3mm_Thk_ILM_RPE_um_I_Hemi_1minus3 | | -8.873 | 7.4591 | -23.493 | 5.746 | 1.415 | 1 | .234 |
| Macula_3mm_Thk_ILM_RPE_um_All_1minus3 | | 17.827 | 7.8914 | 2.360 | 33.294 | 5.103 | 1 | .024 |
| Macula_3mm_Thk_ILM_RPE_um_S_Hemi_0minus3 | | 5.110 | 6.5316 | -7.692 | 17.912 | .612 | 1 | .434 |
| Macula_3mm_Thk_ILM_RPE_um_I_Hemi_0minus3 | | 20.021 | 7.1796 | 5.949 | 34.093 | 7.776 | 1 | **.005** |
| Macula_3mm_Thk_ILM_RPE_um_All_0minus3 | | -21.861 | 7.4194 | -36.403 | -7.319 | 8.682 | 1 | **.003** |
| Macula_3mm_Thk_ILM_RPE_um_S_Hemi_field | | -19.723 | 5.2081 | -29.930 | -9.515 | 14.341 | 1 | **<.001** |
| Macula_3mm_Thk_ILM_RPE_um_I_Hemi_field | | -8.533 | 5.3031 | -18.927 | 1.861 | 2.589 | 1 | .108 |
| Macula_3mm_Thk_ILM_RPE_um_All_field | | -29.863 | 6.5569 | -42.714 | -17.012 | 20.743 | 1 | **<.001** |
| Macula_3mm_Thk_ILM_BRM_um_Center_1 | | 10.691 | 4.7221 | 1.436 | 19.946 | 5.126 | 1 | **.024** |
| Macula_3mm_Thk_ILM_BRM_um_T_1minus3 | | 12.747 | 5.3017 | 2.356 | 23.138 | 5.781 | 1 | **.016** |
| Macula_3mm_Thk_ILM_BRM_um_S_1minus3 | | 4.680 | 5.9143 | -6.912 | 16.272 | .626 | 1 | .429 |
| Macula_3mm_Thk_ILM_BRM_um_N_1minus3 | | 6.566 | 4.9171 | -3.071 | 16.204 | 1.783 | 1 | .182 |
| Macula_3mm_Thk_ILM_BRM_um_I_1minus3 | | -10.705 | 5.4016 | -21.292 | -.118 | 3.928 | 1 | **.047** |
| Macula_3mm_Thk_ILM_BRM_um_S_Hemi_1minus3 | | -19.272 | 6.0107 | -31.053 | -7.492 | 10.281 | 1 | **.001** |
| Macula_3mm_Thk_ILM_BRM_um_I_Hemi_1minus3 | | -14.870 | 6.4007 | -27.416 | -2.325 | 5.398 | 1 | **.020** |
| Macula_3mm_Thk_ILM_BRM_um_All_1minus3 | | 5.622 | 6.8581 | -7.820 | 19.063 | .672 | 1 | .412 |
| Macula_3mm_Thk_ILM_BRM_um_S_Hemi_0minus3 | | -4.927 | 7.0861 | -18.816 | 8.961 | .484 | 1 | .487 |
| Macula_3mm_Thk_ILM_BRM_um_I_Hemi_0minus3 | | 6.706 | 7.4900 | -7.974 | 21.386 | .802 | 1 | .371 |
| Macula_3mm_Thk_ILM_BRM_um_All_0minus3 | | 11.432 | 7.1484 | -2.578 | 25.443 | 2.558 | 1 | .110 |
| Macula_3mm_Thk_ILM_BRM_um_S_Hemi_field | | 22.344 | 5.5881 | 11.391 | 33.296 | 15.988 | 1 | **<.001** |
| Macula_3mm_Thk_ILM_BRM_um_I_Hemi_field | | 11.116 | 5.2499 | .827 | 21.406 | 4.484 | 1 | **.034** |
| Macula_3mm_Thk_ILM_BRM_um_All_field | | 42.138 | 6.4884 | 29.421 | 54.855 | 42.177 | 1 | **<.001** |
| Macula_3mm_Thk_RPE_BRM_um_Center_1 | | -15.051 | 4.7722 | -24.405 | -5.698 | 9.947 | 1 | **.002** |
| Macula_3mm_Thk_RPE_BRM_um_T_1minus3 | | -14.306 | 5.7157 | -25.509 | -3.104 | 6.265 | 1 | **.012** |
| Macula_3mm_Thk_RPE_BRM_um_S_1minus3 | | -12.155 | 6.3787 | -24.657 | .347 | 3.631 | 1 | .057 |
| Macula_3mm_Thk_RPE_BRM_um_N_1minus3 | | -4.619 | 5.5631 | -15.522 | 6.285 | .689 | 1 | .406 |
| Macula_3mm_Thk_RPE_BRM_um_I_1minus3 | | 12.458 | 5.8008 | 1.089 | 23.827 | 4.612 | 1 | **.032** |
| Macula_3mm_Thk_RPE_BRM_um_S_Hemi_1minus3 | | 27.299 | 6.4349 | 14.687 | 39.911 | 17.998 | 1 | **<.001** |
| Macula_3mm_Thk_RPE_BRM_um_I_Hemi_1minus3 | | 1.970 | 7.9859 | -13.683 | 17.622 | .061 | 1 | .805 |
| Macula_3mm_Thk_RPE_BRM_um_All_1minus3 | | -35.526 | 8.3301 | -51.853 | -19.200 | 18.189 | 1 | **<.001** |
| Macula_3mm_Thk_RPE_BRM_um_S_Hemi_0minus3 | | 21.354 | 7.3404 | 6.967 | 35.741 | 8.463 | 1 | **.004** |
| Macula_3mm_Thk_RPE_BRM_um_I_Hemi_0minus3 | | 4.465 | 8.0221 | -11.258 | 20.188 | .310 | 1 | .578 |
| Macula_3mm_Thk_RPE_BRM_um_All_0minus3 | | 2.430 | 8.0322 | -13.313 | 18.172 | .091 | 1 | .762 |
| Macula_3mm_Thk_RPE_BRM_um_S_Hemi_field | | -24.630 | 5.5405 | -35.489 | -13.771 | 19.762 | 1 | **<.001** |
| Macula_3mm_Thk_RPE_BRM_um_I_Hemi_field | | -6.265 | 5.8879 | -17.805 | 5.275 | 1.132 | 1 | .287 |
| Macula_3mm_Thk_RPE_BRM_um_All_field | | -11.380 | 7.1007 | -25.298 | 2.537 | 2.569 | 1 | .109 |
| Macula_3mm_Vol_ILM_IPL_mm3_Center_1 | | 374.885 | 833.1398 | -1258.039 | 2007.809 | .202 | 1 | .653 |
| Macula_3mm_Vol_ILM_IPL_mm3_T_1minus3 | | -535.568 | 727.0269 | -1960.514 | 889.379 | .543 | 1 | .461 |
| Macula_3mm_Vol_ILM_IPL_mm3_S_1minus3 | | 1050.723 | 684.3540 | -290.586 | 2392.032 | 2.357 | 1 | .125 |
| Macula_3mm_Vol_ILM_IPL_mm3_N_1minus3 | | 1832.151 | 582.5197 | 690.433 | 2973.868 | 9.892 | 1 | **.002** |
| Macula_3mm_Vol_ILM_IPL_mm3_I_1minus3 | | 1190.958 | 769.5107 | -317.255 | 2699.171 | 2.395 | 1 | .122 |
| Macula_3mm_Vol_ILM_IPL_mm3_S_Hemi_1minus3 | | 471.551 | 718.7995 | -937.270 | 1880.372 | .430 | 1 | .512 |
| Macula_3mm_Vol_ILM_IPL_mm3_I_Hemi_1minus3 | | 2997.726 | 803.8993 | 1422.112 | 4573.339 | 13.905 | 1 | **<.001** |
| Macula_3mm_Vol_ILM_IPL_mm3_All_1minus3 | | -466.531 | 678.0252 | -1795.436 | 862.374 | .473 | 1 | .491 |
| Macula_3mm_Vol_ILM_IPL_mm3_S_Hemi_0minus3 | | 400.433 | 702.7300 | -976.893 | 1777.758 | .325 | 1 | .569 |
| Macula_3mm_Vol_ILM_IPL_mm3_I_Hemi_0minus3 | | 853.686 | 761.3627 | -638.557 | 2345.930 | 1.257 | 1 | .262 |
| Macula_3mm_Vol_ILM_IPL_mm3_All_0minus3 | | -2980.467 | 713.6042 | -4379.106 | -1581.829 | 17.444 | 1 | **<.001** |
| Macula_3mm_Vol_ILM_IPL_mm3_S_Hemi_field | | 2661.075 | 584.9278 | 1514.638 | 3807.513 | 20.697 | 1 | **<.001** |
| Macula_3mm_Vol_ILM_IPL_mm3_I_Hemi_field | | 1816.804 | 609.0661 | 623.056 | 3010.552 | 8.898 | 1 | **.003** |
| Macula_3mm_Vol_ILM_IPL_mm3_All_field | | 1201.404 | 734.8855 | -238.945 | 2641.753 | 2.673 | 1 | .102 |
| Macula_3mm_Vol_ILM_RPE_mm3_Center_1 | | -2888.155 | 810.3975 | -4476.505 | -1299.805 | 12.701 | 1 | **<.001** |
| Macula_3mm_Vol_ILM_RPE_mm3_T_1minus3 | | -460.738 | 584.8509 | -1607.025 | 685.549 | .621 | 1 | .431 |
| Macula_3mm_Vol_ILM_RPE_mm3_S_1minus3 | | -1103.326 | 675.3738 | -2427.034 | 220.382 | 2.669 | 1 | .102 |
| Macula_3mm_Vol_ILM_RPE_mm3_N_1minus3 | | 690.608 | 659.1306 | -601.264 | 1982.481 | 1.098 | 1 | .295 |
| Macula_3mm_Vol_ILM_RPE_mm3_I_1minus3 | | -2258.931 | 697.5140 | -3626.034 | -891.829 | 10.488 | 1 | **.001** |
| Macula_3mm_Vol_ILM_RPE_mm3_S_Hemi_1minus3 | | -1530.202 | 847.3385 | -3190.955 | 130.551 | 3.261 | 1 | .071 |
| Macula_3mm_Vol_ILM_RPE_mm3_I_Hemi_1minus3 | | -2994.077 | 728.1252 | -4421.176 | -1566.978 | 16.909 | 1 | **<.001** |
| Macula_3mm_Vol_ILM_RPE_mm3_All_1minus3 | | 1171.567 | 791.6012 | -379.943 | 2723.076 | 2.190 | 1 | .139 |
| Macula_3mm_Vol_ILM_RPE_mm3_S_Hemi_0minus3 | | 3420.392 | 711.3796 | 2026.114 | 4814.671 | 23.118 | 1 | **<.001** |
| Macula_3mm_Vol_ILM_RPE_mm3_I_Hemi_0minus3 | | -2964.029 | 755.8234 | -4445.416 | -1482.643 | 15.379 | 1 | **<.001** |
| Macula_3mm_Vol_ILM_RPE_mm3_All_0minus3 | | 676.958 | 831.6239 | -952.995 | 2306.911 | .663 | 1 | .416 |
| Macula_3mm_Vol_ILM_RPE_mm3_S_Hemi_field | | 1122.768 | 580.4025 | -14.800 | 2260.336 | 3.742 | 1 | .053 |
| Macula_3mm_Vol_ILM_RPE_mm3_I_Hemi_field | | -691.005 | 642.9731 | -1951.209 | 569.199 | 1.155 | 1 | .283 |
| Macula_3mm_Vol_ILM_RPE_mm3_All_field | | 370.302 | 758.7102 | -1116.743 | 1857.347 | .238 | 1 | .626 |
| Macula_3mm_Vol_ILM_BRM_mm3_Center_1 | | -5006.243 | 833.9246 | -6640.705 | -3371.781 | 36.039 | 1 | **<.001** |
| Macula_3mm_Vol_ILM_BRM_mm3_T_1minus3 | | 692.046 | 683.7491 | -648.077 | 2032.170 | 1.024 | 1 | .311 |
| Macula_3mm_Vol_ILM_BRM_mm3_S_1minus3 | | 858.680 | 693.4988 | -500.553 | 2217.913 | 1.533 | 1 | .216 |
| Macula_3mm_Vol_ILM_BRM_mm3_N_1minus3 | | -1345.862 | 720.1949 | -2757.418 | 65.694 | 3.492 | 1 | .062 |
| Macula_3mm_Vol_ILM_BRM_mm3_I_1minus3 | | 1638.871 | 724.8718 | 218.149 | 3059.594 | 5.112 | 1 | **.024** |
| Macula_3mm_Vol_ILM_BRM_mm3_S_Hemi_1minus3 | | -2459.460 | 755.5489 | -3940.308 | -978.611 | 10.596 | 1 | **.001** |
| Macula_3mm_Vol_ILM_BRM_mm3_I_Hemi_1minus3 | | 652.765 | 771.1179 | -858.598 | 2164.128 | .717 | 1 | .397 |
| Macula_3mm_Vol_ILM_BRM_mm3_All_1minus3 | | 389.048 | 758.9632 | -1098.492 | 1876.589 | .263 | 1 | .608 |
| Macula_3mm_Vol_ILM_BRM_mm3_S_Hemi_0minus3 | | -1514.483 | 853.8179 | -3187.935 | 158.969 | 3.146 | 1 | .076 |
| Macula_3mm_Vol_ILM_BRM_mm3_I_Hemi_0minus3 | | 1972.479 | 691.2779 | 617.599 | 3327.359 | 8.142 | 1 | **.004** |
| Macula_3mm_Vol_ILM_BRM_mm3_All_0minus3 | | 730.795 | 811.6364 | -859.983 | 2321.573 | .811 | 1 | .368 |
| Macula_3mm_Vol_ILM_BRM_mm3_S_Hemi_field | | -1294.316 | 601.0528 | -2472.358 | -116.274 | 4.637 | 1 | **.031** |
| Macula_3mm_Vol_ILM_BRM_mm3_I_Hemi_field | | 945.155 | 670.0818 | -368.181 | 2258.491 | 1.990 | 1 | .158 |
| Macula_3mm_Vol_ILM_BRM_mm3_All_field | | -2457.876 | 797.6724 | -4021.285 | -894.466 | 9.494 | 1 | **.002** |
| Macula_3mm_Vol_RPE_BRM_mm3_Center_1 | | 2542.004 | 814.5909 | 945.435 | 4138.573 | 9.738 | 1 | **.002** |
| Macula_3mm_Vol_RPE_BRM_mm3_T_1minus3 | | -1279.066 | 767.1227 | -2782.599 | 224.467 | 2.780 | 1 | .095 |
| Macula_3mm_Vol_RPE_BRM_mm3_S_1minus3 | | 1381.711 | 717.0300 | -23.642 | 2787.064 | 3.713 | 1 | .054 |
| Macula_3mm_Vol_RPE_BRM_mm3_N_1minus3 | | -802.590 | 789.6165 | -2350.209 | 745.030 | 1.033 | 1 | .309 |
| Macula_3mm_Vol_RPE_BRM_mm3_I_1minus3 | | -2330.381 | 858.0348 | -4012.099 | -648.664 | 7.376 | 1 | **.007** |
| Macula_3mm_Vol_RPE_BRM_mm3_S_Hemi_1minus3 | | 1355.245 | 754.6894 | -123.919 | 2834.409 | 3.225 | 1 | .073 |
| Macula_3mm_Vol_RPE_BRM_mm3_I_Hemi_1minus3 | | 1619.328 | 856.8778 | -60.122 | 3298.777 | 3.571 | 1 | .059 |
| Macula_3mm_Vol_RPE_BRM_mm3_All_1minus3 | | -1718.775 | 729.3398 | -3148.255 | -289.295 | 5.554 | 1 | **.018** |
| Macula_3mm_Vol_RPE_BRM_mm3_S_Hemi_0minus3 | | -1793.456 | 765.0178 | -3292.863 | -294.049 | 5.496 | 1 | **.019** |
| Macula_3mm_Vol_RPE_BRM_mm3_I_Hemi_0minus3 | | -895.870 | 797.7898 | -2459.509 | 667.770 | 1.261 | 1 | .261 |
| Macula_3mm_Vol_RPE_BRM_mm3_All_0minus3 | | 1800.198 | 752.5603 | 325.207 | 3275.189 | 5.722 | 1 | **.017** |
| Macula_3mm_Vol_RPE_BRM_mm3_S_Hemi_field | | 3257.994 | 651.2647 | 1981.539 | 4534.449 | 25.026 | 1 | **<.001** |
| Macula_3mm_Vol_RPE_BRM_mm3_I_Hemi_field | | -1792.968 | 660.7674 | -3088.048 | -497.887 | 7.363 | 1 | **.007** |
| Macula_3mm_Vol_RPE_BRM_mm3_All_field | | -1649.447 | 724.1197 | -3068.696 | -230.198 | 5.189 | 1 | **.023** |
| (Scale) | | 1^a^ |  |  |  |  |  |  |
| Dependent Variable: Class_Num  Model: (Threshold), Macula_3mm_Thk_ILM_IPL_um_Center_1, Macula_3mm_Thk_ILM_IPL_um_T_1minus3, Macula_3mm_Thk_ILM_IPL_um_S_1minus3, Macula_3mm_Thk_ILM_IPL_um_N_1minus3, Macula_3mm_Thk_ILM_IPL_um_I_1minus3, Macula_3mm_Thk_ILM_IPL_um_S_Hemi_1minus3, Macula_3mm_Thk_ILM_IPL_um_I_Hemi_1minus3, Macula_3mm_Thk_ILM_IPL_um_All_1minus3, Macula_3mm_Thk_ILM_IPL_um_S_Hemi_0minus3, Macula_3mm_Thk_ILM_IPL_um_I_Hemi_0minus3, Macula_3mm_Thk_ILM_IPL_um_All_0minus3, Macula_3mm_Thk_ILM_IPL_um_S_Hemi_field, Macula_3mm_Thk_ILM_IPL_um_I_Hemi_field, Macula_3mm_Thk_ILM_IPL_um_All_field, Macula_3mm_Thk_ILM_RPE_um_Center_1, Macula_3mm_Thk_ILM_RPE_um_T_1minus3, Macula_3mm_Thk_ILM_RPE_um_S_1minus3, Macula_3mm_Thk_ILM_RPE_um_N_1minus3, Macula_3mm_Thk_ILM_RPE_um_I_1minus3, Macula_3mm_Thk_ILM_RPE_um_S_Hemi_1minus3, Macula_3mm_Thk_ILM_RPE_um_I_Hemi_1minus3, Macula_3mm_Thk_ILM_RPE_um_All_1minus3, Macula_3mm_Thk_ILM_RPE_um_S_Hemi_0minus3, Macula_3mm_Thk_ILM_RPE_um_I_Hemi_0minus3, Macula_3mm_Thk_ILM_RPE_um_All_0minus3, Macula_3mm_Thk_ILM_RPE_um_S_Hemi_field, Macula_3mm_Thk_ILM_RPE_um_I_Hemi_field, Macula_3mm_Thk_ILM_RPE_um_All_field, Macula_3mm_Thk_ILM_BRM_um_Center_1, Macula_3mm_Thk_ILM_BRM_um_T_1minus3, Macula_3mm_Thk_ILM_BRM_um_S_1minus3, Macula_3mm_Thk_ILM_BRM_um_N_1minus3, Macula_3mm_Thk_ILM_BRM_um_I_1minus3, Macula_3mm_Thk_ILM_BRM_um_S_Hemi_1minus3, Macula_3mm_Thk_ILM_BRM_um_I_Hemi_1minus3, Macula_3mm_Thk_ILM_BRM_um_All_1minus3, Macula_3mm_Thk_ILM_BRM_um_S_Hemi_0minus3, Macula_3mm_Thk_ILM_BRM_um_I_Hemi_0minus3, Macula_3mm_Thk_ILM_BRM_um_All_0minus3, Macula_3mm_Thk_ILM_BRM_um_S_Hemi_field, Macula_3mm_Thk_ILM_BRM_um_I_Hemi_field, Macula_3mm_Thk_ILM_BRM_um_All_field, Macula_3mm_Thk_RPE_BRM_um_Center_1, Macula_3mm_Thk_RPE_BRM_um_T_1minus3, Macula_3mm_Thk_RPE_BRM_um_S_1minus3, Macula_3mm_Thk_RPE_BRM_um_N_1minus3, Macula_3mm_Thk_RPE_BRM_um_I_1minus3, Macula_3mm_Thk_RPE_BRM_um_S_Hemi_1minus3, Macula_3mm_Thk_RPE_BRM_um_I_Hemi_1minus3, Macula_3mm_Thk_RPE_BRM_um_All_1minus3, Macula_3mm_Thk_RPE_BRM_um_S_Hemi_0minus3, Macula_3mm_Thk_RPE_BRM_um_I_Hemi_0minus3, Macula_3mm_Thk_RPE_BRM_um_All_0minus3, Macula_3mm_Thk_RPE_BRM_um_S_Hemi_field, Macula_3mm_Thk_RPE_BRM_um_I_Hemi_field, Macula_3mm_Thk_RPE_BRM_um_All_field, Macula_3mm_Vol_ILM_IPL_mm3_Center_1, Macula_3mm_Vol_ILM_IPL_mm3_T_1minus3, Macula_3mm_Vol_ILM_IPL_mm3_S_1minus3, Macula_3mm_Vol_ILM_IPL_mm3_N_1minus3, Macula_3mm_Vol_ILM_IPL_mm3_I_1minus3, Macula_3mm_Vol_ILM_IPL_mm3_S_Hemi_1minus3, Macula_3mm_Vol_ILM_IPL_mm3_I_Hemi_1minus3, Macula_3mm_Vol_ILM_IPL_mm3_All_1minus3, Macula_3mm_Vol_ILM_IPL_mm3_S_Hemi_0minus3, Macula_3mm_Vol_ILM_IPL_mm3_I_Hemi_0minus3, Macula_3mm_Vol_ILM_IPL_mm3_All_0minus3, Macula_3mm_Vol_ILM_IPL_mm3_S_Hemi_field, Macula_3mm_Vol_ILM_IPL_mm3_I_Hemi_field, Macula_3mm_Vol_ILM_IPL_mm3_All_field, Macula_3mm_Vol_ILM_RPE_mm3_Center_1, Macula_3mm_Vol_ILM_RPE_mm3_T_1minus3, Macula_3mm_Vol_ILM_RPE_mm3_S_1minus3, Macula_3mm_Vol_ILM_RPE_mm3_N_1minus3, Macula_3mm_Vol_ILM_RPE_mm3_I_1minus3, Macula_3mm_Vol_ILM_RPE_mm3_S_Hemi_1minus3, Macula_3mm_Vol_ILM_RPE_mm3_I_Hemi_1minus3, Macula_3mm_Vol_ILM_RPE_mm3_All_1minus3, Macula_3mm_Vol_ILM_RPE_mm3_S_Hemi_0minus3, Macula_3mm_Vol_ILM_RPE_mm3_I_Hemi_0minus3, Macula_3mm_Vol_ILM_RPE_mm3_All_0minus3, Macula_3mm_Vol_ILM_RPE_mm3_S_Hemi_field, Macula_3mm_Vol_ILM_RPE_mm3_I_Hemi_field, Macula_3mm_Vol_ILM_RPE_mm3_All_field, Macula_3mm_Vol_ILM_BRM_mm3_Center_1, Macula_3mm_Vol_ILM_BRM_mm3_T_1minus3, Macula_3mm_Vol_ILM_BRM_mm3_S_1minus3, Macula_3mm_Vol_ILM_BRM_mm3_N_1minus3, Macula_3mm_Vol_ILM_BRM_mm3_I_1minus3, Macula_3mm_Vol_ILM_BRM_mm3_S_Hemi_1minus3, Macula_3mm_Vol_ILM_BRM_mm3_I_Hemi_1minus3, Macula_3mm_Vol_ILM_BRM_mm3_All_1minus3, Macula_3mm_Vol_ILM_BRM_mm3_S_Hemi_0minus3, Macula_3mm_Vol_ILM_BRM_mm3_I_Hemi_0minus3, Macula_3mm_Vol_ILM_BRM_mm3_All_0minus3, Macula_3mm_Vol_ILM_BRM_mm3_S_Hemi_field, Macula_3mm_Vol_ILM_BRM_mm3_I_Hemi_field, Macula_3mm_Vol_ILM_BRM_mm3_All_field, Macula_3mm_Vol_RPE_BRM_mm3_Center_1, Macula_3mm_Vol_RPE_BRM_mm3_T_1minus3, Macula_3mm_Vol_RPE_BRM_mm3_S_1minus3, Macula_3mm_Vol_RPE_BRM_mm3_N_1minus3, Macula_3mm_Vol_RPE_BRM_mm3_I_1minus3, Macula_3mm_Vol_RPE_BRM_mm3_S_Hemi_1minus3, Macula_3mm_Vol_RPE_BRM_mm3_I_Hemi_1minus3, Macula_3mm_Vol_RPE_BRM_mm3_All_1minus3, Macula_3mm_Vol_RPE_BRM_mm3_S_Hemi_0minus3, Macula_3mm_Vol_RPE_BRM_mm3_I_Hemi_0minus3, Macula_3mm_Vol_RPE_BRM_mm3_All_0minus3, Macula_3mm_Vol_RPE_BRM_mm3_S_Hemi_field, Macula_3mm_Vol_RPE_BRM_mm3_I_Hemi_field, Macula_3mm_Vol_RPE_BRM_mm3_All_field, offset = AgePerToday | | | | | | | | |
| a. Fixed at the displayed value. | | | | | | | | |

### Using Retina3DflowDensity parameters

| **Parameter Estimates** | | | | | | | | |
| --- | --- | --- | --- | --- | --- | --- | --- | --- |
| Parameter | | B | Std. Error | 95% Wald Confidence Interval | | Hypothesis Test | | |
|  |  |  |  | Lower | Upper | Wald Chi-Square | df | Sig. |
| Threshold | [Class_Num=0] | -132.154 | 7.5911 | -147.032 | -117.276 | 303.077 | 1 | .000 |
|  | [Class_Num=1] | -128.389 | 7.5702 | -143.226 | -113.551 | 287.633 | 1 | .000 |
| SVC_L1_DensityOfWhole_Image | | -104.283 | 31.2934 | -165.617 | -42.949 | 11.105 | 1 | **<.001** |
| SVC_L1_Whole_Image_S_Hemi | | 9.143 | 3.8800 | 1.538 | 16.748 | 5.553 | 1 | **.018** |
| SVC_L1_Whole_Image_I_Hemi | | 9.056 | 4.0711 | 1.077 | 17.036 | 4.949 | 1 | **.026** |
| SVC_L1_Whole_ETDRS | | 45.568 | 21.2272 | 3.964 | 87.173 | 4.608 | 1 | **.032** |
| SVC_L1_Fovea | | -5.733 | 2.4389 | -10.513 | -.953 | 5.526 | 1 | **.019** |
| SVC_L1_ParaFovea | | 5.708 | 36.3051 | -65.448 | 76.865 | .025 | 1 | .875 |
| SVC_L1_Para_S_Hemi | | -11.370 | 16.1214 | -42.967 | 20.228 | .497 | 1 | .481 |
| SVC_L1_Para_I_Hemi | | -11.606 | 16.1609 | -43.281 | 20.068 | .516 | 1 | .473 |
| SVC_L1_Para_T | | -5.745 | 2.1290 | -9.918 | -1.572 | 7.282 | 1 | **.007** |
| SVC_L1_Para_S | | -4.989 | 2.1023 | -9.109 | -.869 | 5.632 | 1 | **.018** |
| SVC_L1_Para_N | | -5.649 | 2.1001 | -9.765 | -1.533 | 7.236 | 1 | **.007** |
| SVC_L1_Para_I | | -5.566 | 2.1140 | -9.709 | -1.422 | 6.932 | 1 | **.008** |
| SVC_L1_G11 | | 9.314 | 3.4475 | 2.557 | 16.071 | 7.299 | 1 | **.007** |
| SVC_L1_G12 | | 9.046 | 3.4225 | 2.338 | 15.754 | 6.986 | 1 | **.008** |
| SVC_L1_G13 | | 9.516 | 3.4117 | 2.829 | 16.203 | 7.780 | 1 | **.005** |
| SVC_L1_G21 | | 9.507 | 3.4213 | 2.801 | 16.212 | 7.721 | 1 | **.005** |
| SVC_L1_G22 | | 10.120 | 3.3794 | 3.496 | 16.743 | 8.967 | 1 | **.003** |
| SVC_L1_G23 | | 9.807 | 3.3837 | 3.175 | 16.438 | 8.399 | 1 | **.004** |
| SVC_L1_G31 | | 9.421 | 3.4213 | 2.715 | 16.127 | 7.582 | 1 | **.006** |
| SVC_L1_G32 | | 9.182 | 3.4024 | 2.514 | 15.851 | 7.283 | 1 | **.007** |
| SVC_L1_G33 | | 9.140 | 3.3787 | 2.518 | 15.762 | 7.318 | 1 | **.007** |
| DVC_L2_DensityOfWhole_Image | | -304.639 | 62.1119 | -426.376 | -182.902 | 24.056 | 1 | **<.001** |
| DVC_L2_Whole_Image_S_Hemi | | -18.637 | 3.9182 | -26.317 | -10.958 | 22.625 | 1 | **<.001** |
| DVC_L2_Whole_Image_I_Hemi | | -16.253 | 4.1367 | -24.361 | -8.145 | 15.437 | 1 | **<.001** |
| DVC_L2_Whole_ETDRS | | -23.293 | 31.5921 | -85.212 | 38.627 | .544 | 1 | .461 |
| DVC_L2_Fovea | | 2.433 | 3.5887 | -4.601 | 9.467 | .460 | 1 | .498 |
| DVC_L2_ParaFovea | | -110.829 | 40.8698 | -190.933 | -30.726 | 7.354 | 1 | **.007** |
| DVC_L2_Para_S_Hemi | | 57.271 | 17.0068 | 23.938 | 90.603 | 11.340 | 1 | **<.001** |
| DVC_L2_Para_I_Hemi | | 55.442 | 17.1215 | 21.884 | 88.999 | 10.486 | 1 | **.001** |
| DVC_L2_Para_T | | 4.955 | 2.0800 | .879 | 9.032 | 5.675 | 1 | **.017** |
| DVC_L2_Para_S | | 4.386 | 2.0517 | .365 | 8.408 | 4.570 | 1 | **.033** |
| DVC_L2_Para_N | | 4.863 | 2.0878 | .771 | 8.955 | 5.425 | 1 | **.020** |
| DVC_L2_Para_I | | 4.482 | 2.0880 | .390 | 8.574 | 4.608 | 1 | **.032** |
| DVC_L2_G11 | | 38.040 | 7.1894 | 23.949 | 52.131 | 27.997 | 1 | **<.001** |
| DVC_L2_G12 | | 38.185 | 7.1034 | 24.262 | 52.107 | 28.897 | 1 | **<.001** |
| DVC_L2_G13 | | 38.344 | 7.1270 | 24.376 | 52.313 | 28.946 | 1 | **<.001** |
| DVC_L2_G21 | | 37.219 | 7.1100 | 23.283 | 51.154 | 27.402 | 1 | **<.001** |
| DVC_L2_G22 | | 37.301 | 7.0045 | 23.573 | 51.030 | 28.359 | 1 | **<.001** |
| DVC_L2_G23 | | 37.075 | 7.0600 | 23.238 | 50.913 | 27.578 | 1 | **<.001** |
| DVC_L2_G31 | | 38.040 | 7.0941 | 24.136 | 51.944 | 28.753 | 1 | **<.001** |
| DVC_L2_G32 | | 37.450 | 7.0202 | 23.691 | 51.210 | 28.459 | 1 | **<.001** |
| DVC_L2_G33 | | 37.381 | 7.0354 | 23.592 | 51.170 | 28.231 | 1 | **<.001** |
| FAZ_Area | | 22.529 | 14.8190 | -6.516 | 51.573 | 2.311 | 1 | .128 |
| Perimeter | | -12.215 | 3.4552 | -18.987 | -5.443 | 12.498 | 1 | **<.001** |
| AcircularityIndex | | 18.878 | 7.3046 | 4.561 | 33.194 | 6.679 | 1 | **.010** |
| FD_300_Area_Density | | .334 | .1320 | .075 | .592 | 6.387 | 1 | **.011** |
| FD_300_Length_Density | | -1.207 | .2132 | -1.625 | -.790 | 32.070 | 1 | **<.001** |
| (Scale) | | 1^a^ |  |  |  |  |  |  |
| Dependent Variable: Class_Num  Model: (Threshold), SVC_L1_DensityOfWhole_Image, SVC_L1_Whole_Image_S_Hemi, SVC_L1_Whole_Image_I_Hemi, SVC_L1_Whole_ETDRS, SVC_L1_Fovea, SVC_L1_ParaFovea, SVC_L1_Para_S_Hemi, SVC_L1_Para_I_Hemi, SVC_L1_Para_T, SVC_L1_Para_S, SVC_L1_Para_N, SVC_L1_Para_I, SVC_L1_G11, SVC_L1_G12, SVC_L1_G13, SVC_L1_G21, SVC_L1_G22, SVC_L1_G23, SVC_L1_G31, SVC_L1_G32, SVC_L1_G33, DVC_L2_DensityOfWhole_Image, DVC_L2_Whole_Image_S_Hemi, DVC_L2_Whole_Image_I_Hemi, DVC_L2_Whole_ETDRS, DVC_L2_Fovea, DVC_L2_ParaFovea, DVC_L2_Para_S_Hemi, DVC_L2_Para_I_Hemi, DVC_L2_Para_T, DVC_L2_Para_S, DVC_L2_Para_N, DVC_L2_Para_I, DVC_L2_G11, DVC_L2_G12, DVC_L2_G13, DVC_L2_G21, DVC_L2_G22, DVC_L2_G23, DVC_L2_G31, DVC_L2_G32, DVC_L2_G33, FAZ_Area, Perimeter, AcircularityIndex, FD_300_Area_Density, FD_300_Length_Density, offset = AgePerToday | | | | | | | | |
| a. Fixed at the displayed value. | | | | | | | | |

### Using Retina Map - Inner Retina Thickness parameters

| **Parameter Estimates** | | | | | | | | |
| --- | --- | --- | --- | --- | --- | --- | --- | --- |
| Parameter | | B | Std. Error | 95% Wald Confidence Interval | | Hypothesis Test | | |
|  |  |  |  | Lower | Upper | Wald Chi-Square | df | Sig. |
| Threshold | [Class_Num=0] | -102.411 | 2.3155 | -106.949 | -97.873 | 1956.090 | 1 | .000 |
|  | [Class_Num=1] | -99.236 | 2.3091 | -103.762 | -94.710 | 1846.870 | 1 | .000 |
| FoveaInnRet_Thickness | | .043 | .0159 | .012 | .074 | 7.445 | 1 | **.006** |
| InnRet_Thk_ParaFovea | | 1.916 | .4900 | .955 | 2.876 | 15.289 | 1 | **<.001** |
| InnRet_Thk_Para_S_Hemisphere | | -.807 | .2603 | -1.318 | -.297 | 9.620 | 1 | **.002** |
| InnRet_Thk_Para_I_Hemisphere | | -.025 | .2913 | -.596 | .546 | .007 | 1 | .931 |
| InnRet_Thk_Para_Tempo | | -.477 | .1280 | -.728 | -.226 | 13.875 | 1 | **<.001** |
| InnRet_Thk_Para_Superior | | -.035 | .1491 | -.327 | .257 | .055 | 1 | .815 |
| InnRet_Thk_Para_Nasal | | -.264 | .1269 | -.513 | -.016 | 4.346 | 1 | **.037** |
| InnRet_Thk_Para_Inferior | | -.468 | .1583 | -.778 | -.158 | 8.735 | 1 | **.003** |
| InnRet_Thk_PeriFovea | | .571 | .3214 | -.059 | 1.201 | 3.157 | 1 | .076 |
| InnRet_Thk_Peri_S_Hemisphere | | -.184 | .2188 | -.613 | .245 | .706 | 1 | .401 |
| InnRet_Thk_Peri_I_Hemisphere | | -.402 | .2157 | -.825 | .020 | 3.482 | 1 | .062 |
| InnRet_Thk_Peri_Tempo | | .058 | .0872 | -.113 | .229 | .443 | 1 | .506 |
| InnRet_Thk_Peri_Superior | | .021 | .0984 | -.172 | .214 | .046 | 1 | .830 |
| InnRet_Thk_Peri_Nasal | | -.103 | .0844 | -.268 | .063 | 1.477 | 1 | .224 |
| InnRet_Thk_Peri_Inferior | | -.022 | .1007 | -.219 | .176 | .046 | 1 | .830 |
| (Scale) | | 1^a^ |  |  |  |  |  |  |
| Dependent Variable: Class_Num  Model: (Threshold), FoveaInnRet_Thickness, InnRet_Thk_ParaFovea, InnRet_Thk_Para_S_Hemisphere, InnRet_Thk_Para_I_Hemisphere, InnRet_Thk_Para_Tempo, InnRet_Thk_Para_Superior, InnRet_Thk_Para_Nasal, InnRet_Thk_Para_Inferior, InnRet_Thk_PeriFovea, InnRet_Thk_Peri_S_Hemisphere, InnRet_Thk_Peri_I_Hemisphere, InnRet_Thk_Peri_Tempo, InnRet_Thk_Peri_Superior, InnRet_Thk_Peri_Nasal, InnRet_Thk_Peri_Inferior, offset = AgePerToday | | | | | | | | |
| a. Fixed at the displayed value. | | | | | | | | |

### Using Retina Map - Inner Retina Volume parameters

| **Parameter Estimates** | | | | | | | | |
| --- | --- | --- | --- | --- | --- | --- | --- | --- |
| Parameter | | B | Std. Error | 95% Wald Confidence Interval | | Hypothesis Test | | |
|  |  |  |  | Lower | Upper | Wald Chi-Square | df | Sig. |
| Threshold | [Class_Num=0] | -105.472 | 2.2038 | -109.792 | -101.153 | 2290.463 | 1 | .000 |
|  | [Class_Num=1] | -102.205 | 2.1949 | -106.507 | -97.903 | 2168.310 | 1 | .000 |
| FoveaInnRet_Volumn | | 45.114 | 20.4219 | 5.088 | 85.140 | 4.880 | 1 | **.027** |
| InnRet_Vol_ParaFovea | | -192.600 | 60.8462 | -311.857 | -73.344 | 10.020 | 1 | **.002** |
| InnRet_Vol_Para_S_Hemisphere | | 336.437 | 101.3692 | 137.757 | 535.116 | 11.015 | 1 | **<.001** |
| InnRet_Vol_Para_I_Hemisphere | | 333.029 | 90.2580 | 156.127 | 509.932 | 13.614 | 1 | **<.001** |
| InnRet_Vol_Para_Tempo | | -269.859 | 77.8413 | -422.425 | -117.293 | 12.019 | 1 | **<.001** |
| InnRet_Vol_Para_Superior | | -156.706 | 98.1465 | -349.070 | 35.657 | 2.549 | 1 | .110 |
| InnRet_Vol_Para_Nasal | | -159.739 | 72.2724 | -301.390 | -18.087 | 4.885 | 1 | **.027** |
| InnRet_Vol_Para_Inferior | | -93.711 | 58.5263 | -208.421 | 20.998 | 2.564 | 1 | .109 |
| InnRet_Vol_PeriFovea | | -122.013 | 59.2692 | -238.178 | -5.847 | 4.238 | 1 | **.040** |
| InnRet_Vol_Peri_S_Hemisphere | | -320.559 | 143.2121 | -601.250 | -39.869 | 5.010 | 1 | **.025** |
| InnRet_Vol_Peri_I_Hemisphere | | -384.353 | 139.5279 | -657.823 | -110.883 | 7.588 | 1 | **.006** |
| InnRet_Vol_Peri_Tempo | | 483.711 | 127.4335 | 233.946 | 733.476 | 14.408 | 1 | **<.001** |
| InnRet_Vol_Peri_Superior | | 460.120 | 135.3658 | 194.808 | 725.433 | 11.554 | 1 | **<.001** |
| InnRet_Vol_Peri_Nasal | | 449.320 | 126.8904 | 200.619 | 698.020 | 12.539 | 1 | **<.001** |
| InnRet_Vol_Peri_Inferior | | 482.941 | 131.7124 | 224.790 | 741.093 | 13.444 | 1 | **<.001** |
| (Scale) | | 1^a^ |  |  |  |  |  |  |
| Dependent Variable: Class_Num  Model: (Threshold), FoveaInnRet_Volumn, InnRet_Vol_ParaFovea, InnRet_Vol_Para_S_Hemisphere, InnRet_Vol_Para_I_Hemisphere, InnRet_Vol_Para_Tempo, InnRet_Vol_Para_Superior, InnRet_Vol_Para_Nasal, InnRet_Vol_Para_Inferior, InnRet_Vol_PeriFovea, InnRet_Vol_Peri_S_Hemisphere, InnRet_Vol_Peri_I_Hemisphere, InnRet_Vol_Peri_Tempo, InnRet_Vol_Peri_Superior, InnRet_Vol_Peri_Nasal, InnRet_Vol_Peri_Inferior, offset = AgePerToday | | | | | | | | |
| a. Fixed at the displayed value. | | | | | | | | |

### Using Retina Map - Full Retina Thickness/Volume and RPE parameters

| **Parameter Estimates** | | | | | | | | |
| --- | --- | --- | --- | --- | --- | --- | --- | --- |
| Parameter | | B | Std. Error | 95% Wald Confidence Interval | | Hypothesis Test | | |
|  |  |  |  | Lower | Upper | Wald Chi-Square | df | Sig. |
| Threshold | [Class_Num=0] | -112.731 | 3.2363 | -119.074 | -106.388 | 1213.399 | 1 | .000 |
|  | [Class_Num=1] | -109.093 | 3.1950 | -115.355 | -102.831 | 1165.853 | 1 | .000 |
| FoveaFullRet_Thickness | | .362 | .1604 | .048 | .677 | 5.106 | 1 | **.024** |
| FullRet_Thk_ParaFovea | | -2.510 | .5778 | -3.642 | -1.377 | 18.870 | 1 | **<.001** |
| FullRet_Thk_Para_S_Hemisphere | | .259 | .5465 | -.812 | 1.330 | .224 | 1 | .636 |
| FullRet_Thk_Para_I_Hemisphere | | 1.815 | .3825 | 1.065 | 2.564 | 22.503 | 1 | **<.001** |
| FullRet_Thk_Para_Tempo | | .309 | .1765 | -.037 | .655 | 3.067 | 1 | .080 |
| FullRet_Thk_Para_Superior | | .477 | .2926 | -.097 | 1.050 | 2.657 | 1 | .103 |
| FullRet_Thk_Para_Nasal | | -.340 | .3326 | -.992 | .312 | 1.046 | 1 | .306 |
| FullRet_Thk_Para_Inferior | | -.760 | .2930 | -1.335 | -.186 | 6.733 | 1 | **.009** |
| FullRet_Thk_PeriFovea | | -1.129 | .4651 | -2.041 | -.217 | 5.890 | 1 | **.015** |
| FullRet_Thk_Peri_S_Hemisphere | | 1.742 | .3288 | 1.098 | 2.386 | 28.081 | 1 | **<.001** |
| FullRet_Thk_Peri_I_Hemisphere | | .942 | .4482 | .064 | 1.820 | 4.417 | 1 | **.036** |
| FullRet_Thk_Peri_Tempo | | -.276 | .0832 | -.439 | -.112 | 10.963 | 1 | **<.001** |
| FullRet_Thk_Peri_Superior | | -.528 | .1389 | -.800 | -.255 | 14.420 | 1 | **<.001** |
| FullRet_Thk_Peri_Nasal | | -.221 | .1274 | -.471 | .029 | 3.009 | 1 | .083 |
| FullRet_Thk_Peri_Inferior | | -.484 | .2668 | -1.007 | .039 | 3.294 | 1 | .070 |
| FoveaFullRet_Volumn | | -432.803 | 202.6171 | -829.925 | -35.681 | 4.563 | 1 | **.033** |
| FullRet_Vol_ParaFovea | | -701.849 | 425.5136 | -1535.840 | 132.143 | 2.721 | 1 | .099 |
| FullRet_Vol_Para_S_Hemisphere | | 1931.155 | 401.8544 | 1143.535 | 2718.775 | 23.094 | 1 | **<.001** |
| FullRet_Vol_Para_I_Hemisphere | | 1769.329 | 393.5067 | 998.070 | 2540.588 | 20.217 | 1 | **<.001** |
| FullRet_Vol_Para_Tempo | | -1254.639 | 294.2359 | -1831.331 | -677.947 | 18.182 | 1 | **<.001** |
| FullRet_Vol_Para_Superior | | -1227.336 | 319.2702 | -1853.094 | -601.578 | 14.778 | 1 | **<.001** |
| FullRet_Vol_Para_Nasal | | -832.834 | 293.4688 | -1408.023 | -257.646 | 8.054 | 1 | **.005** |
| FullRet_Vol_Para_Inferior | | -836.276 | 353.9144 | -1529.935 | -142.616 | 5.583 | 1 | **.018** |
| FullRet_Vol_PeriFovea | | 205.438 | 80.8635 | 46.949 | 363.928 | 6.454 | 1 | **.011** |
| FullRet_Vol_Peri_S_Hemisphere | | 303.305 | 285.5352 | -256.333 | 862.944 | 1.128 | 1 | .288 |
| FullRet_Vol_Peri_I_Hemisphere | | 316.323 | 279.5529 | -231.590 | 864.237 | 1.280 | 1 | .258 |
| FullRet_Vol_Peri_Tempo | | -544.381 | 281.7183 | -1096.538 | 7.777 | 3.734 | 1 | .053 |
| FullRet_Vol_Peri_Superior | | -512.601 | 284.0371 | -1069.303 | 44.102 | 3.257 | 1 | .071 |
| FullRet_Vol_Peri_Nasal | | -582.045 | 277.7257 | -1126.378 | -37.713 | 4.392 | 1 | **.036** |
| FullRet_Vol_Peri_Inferior | | -461.779 | 278.5702 | -1007.766 | 84.209 | 2.748 | 1 | .097 |
| FoveaRPE_Elevation_Height | | -.007 | .0224 | -.051 | .037 | .099 | 1 | .753 |
| RPE_Elev_ParaFovea_Tempo | | .104 | .0265 | .052 | .156 | 15.495 | 1 | **<.001** |
| RPE_Elev_Para_Superior | | .050 | .0309 | -.010 | .111 | 2.653 | 1 | .103 |
| RPE_Elev_Para_Nasal | | .003 | .0201 | -.037 | .042 | .017 | 1 | .895 |
| RPE_Elev_Para_Inferior | | -.139 | .0250 | -.188 | -.090 | 30.837 | 1 | **<.001** |
| RPE_Elev_PeriFovea_Tempo | | .046 | .0146 | .017 | .075 | 9.959 | 1 | **.002** |
| RPE_Elev_Peri_Superior | | -.113 | .0234 | -.159 | -.067 | 23.167 | 1 | **<.001** |
| RPE_Elev_Peri_Nasal | | .021 | .0186 | -.016 | .057 | 1.243 | 1 | .265 |
| RPE_Elev_Peri_Inferior | | .074 | .0225 | .030 | .118 | 10.871 | 1 | **<.001** |
| (Scale) | | 1^a^ |  |  |  |  |  |  |
| Dependent Variable: Class_Num  Model: (Threshold), FoveaFullRet_Thickness, FullRet_Thk_ParaFovea, FullRet_Thk_Para_S_Hemisphere, FullRet_Thk_Para_I_Hemisphere, FullRet_Thk_Para_Tempo, FullRet_Thk_Para_Superior, FullRet_Thk_Para_Nasal, FullRet_Thk_Para_Inferior, FullRet_Thk_PeriFovea, FullRet_Thk_Peri_S_Hemisphere, FullRet_Thk_Peri_I_Hemisphere, FullRet_Thk_Peri_Tempo, FullRet_Thk_Peri_Superior, FullRet_Thk_Peri_Nasal, FullRet_Thk_Peri_Inferior, FoveaFullRet_Volumn, FullRet_Vol_ParaFovea, FullRet_Vol_Para_S_Hemisphere, FullRet_Vol_Para_I_Hemisphere, FullRet_Vol_Para_Tempo, FullRet_Vol_Para_Superior, FullRet_Vol_Para_Nasal, FullRet_Vol_Para_Inferior, FullRet_Vol_PeriFovea, FullRet_Vol_Peri_S_Hemisphere, FullRet_Vol_Peri_I_Hemisphere, FullRet_Vol_Peri_Tempo, FullRet_Vol_Peri_Superior, FullRet_Vol_Peri_Nasal, FullRet_Vol_Peri_Inferior, FoveaRPE_Elevation_Height, RPE_Elev_ParaFovea_Tempo, RPE_Elev_Para_Superior, RPE_Elev_Para_Nasal, RPE_Elev_Para_Inferior, RPE_Elev_PeriFovea_Tempo, RPE_Elev_Peri_Superior, RPE_Elev_Peri_Nasal, RPE_Elev_Peri_Inferior, offset = AgePerToday | | | | | | | | |
| a. Fixed at the displayed value. | | | | | | | | |

# Details of Parameters

GCC related parameters investigates Average, Superior (s), Inferior (I), I subtracted from S (S_I) thicknesses in the following layers definitions: 1) $Inner\_Retina$ (InnRet: ILM to 10 μm below OPL (1)), 2) $Full\_Retina$ (FullRet: ILM to RPE/BRM complex (1)), and 3) $Outer\_Retina$ (OutRet:10 μm below OPL to RPE/BRM complex (2) (3)). Additionally, global loss volume (GLV), focal loss volume (FLV), and root mean square (RMS) (4).

ONH parameters were categorized into 1) optic disc analysis and 2) peripapillary RNFL (pRNFL) thickness. Parameters based on optic disc analysis: optic disc area/volume (ONH_DiscArea, ONH_Disc_Volume), cup area/volume (ONH_CupArea, ONH_CupVolume), rim area/volume (ONH_RimArea, ONH_RimVolume), and cup-to-disc (area: ONH_Area_C_D_ratio, horizontal: ONH_H_C_D_ratio, vertical: ONH_V_C_D_ratio) ratios. Parameters based on pRNFL thickness: hemisphere S (S-Hemi) and hemisphere I (I-Hemi), and average pRNFL thickness (5). Additionally, other pRNFL regions included quadrants-based Superior (S), Inferior (I), Temporal (T), Nasal (N), as well as supertemporal (ST), superonasal (SN), inferotemporal (IT), inferonasal (IN), nasal upper (NU), nasal lower (NL), temporal upper (TU), and temporal lower (TL).

Macula_3mm parameters target retinal thickness/volume changes in various quadrants and layers (ILM to IPL, ILM to RPE, ILM to BRM, and RPE to BRM). The thickness/volume analyses for distinct layers definitions include $Center\_1$ (1mm foveal ring), quadrants (S, I, T, N) in the 3mm ring excluding 1mm (1minus3), S and I hemispheres in 1minus3, and combined hemispheres (All_1minus3). Additionally, the thickness/volume analyses for distinct layers definitions include S and I hemispheres field (including $Center\_1$), combined hemispheres field (All_field).

The retina map measures retinal OCT thickness (μm) and volume ($mm^{3}$) around the fovea using 1 mm, 3 mm, and 5 mm circles. The foveal thickness/volume was denoted by $Fovea$, whilst quadrants of ($S_{3}, I_{3}, T_{3}, N_{3}$) and ($S_{5}, I_{5}, T_{5}, N_{5}$) corresponded to parafovea (Para) and perifovea (Peri), respectively. Also, hemispheres $S\_Hemi_{3}$ (Para_S_Hemisphere) and $I\_Hemi_{3}$ (Para_I_Hemisphere) represented 3 mm rings, while $S\_Hemi_{5}$ (Peri_S_Hemisphere) and $I\_Hemi_{5}$ (Peri_I_Hemisphere) indicated 5 mm rings. Additionally, $parafovea$ was the combined $S\_Hemi_{3}$ and $I\_Hemi_{3}$, whilst $perifovea$was the combined $S\_Hemi_{5}$ and $I\_Hemi_{5}$. The retina map ($\mu m/mm^{3}$) was calculated in $Inner\_Retina$ and $Full\_Retina$ layers, similarly to GCC parameters, as well as in RPE thickness (Elevation).

Retina3DFlowDensity investigates retinal VD changes using quadrants (S, I, T, N), hemispheres (S and I), not following Early Treatment of Diabetic Retinopathy Study (ETDRS) ($Whole\_Image$), both hemispheres combined (Whole_ETDRS), $Whole Image$ in $S\_Hemi_{3}$ and$I\_Hemi_{3}$, the 3x3 grid G, and $FD\_300$ ($Area\_Density$ and $Length\_Density$). Other Retina3DFlowDensity parameters include $Fovea$, $FAZ\_Area$, $FAZ\_Perim$ (Perimeter) (6), and $AcirIndx$(AcircularityIndex) (7). Importantly, VD was computed for various retinal layers including A) superficial vascular complex (SVC) from ILM to 10 μm above the IPL, B) deep vascular complex (DVC) from 10 μm above IPL to 10 μm below OPL, C) inner vascular complex (IVC) from ILM to 10 μm below OPL.

# References

1. Hanumunthadu D, Keane PA, Balaskas K, Dubis AM, Kalitzeos A, Michaelides M, et al. Agreement between spectral-domain and swept-source optical coherence tomography retinal thickness measurements in macular and retinal disease. Ophthalmology and therapy. 2021;10:913-22.

2. Venkatesh R, Sinha S, Gangadharaiah D, Gadde SG, Mohan A, Shetty R, et al. Retinal structural-vascular-functional relationship using optical coherence tomography and optical coherence tomography–angiography in myopia. Eye and Vision. 2019;6(1):1-12.

3. Ye J, Wang M, Shen M, Huang S, Xue A, Lin J, et al. Deep retinal capillary plexus decreasing correlated with the outer retinal layer alteration and visual acuity impairment in pathological myopia. Investigative ophthalmology & visual science. 2020;61(4):45-.

4. Rao HL, Zangwill LM, Weinreb RN, Sample PA, Alencar LM, Medeiros FA. Comparison of different spectral domain optical coherence tomography scanning areas for glaucoma diagnosis. Ophthalmology. 2010;117(9):1692-9. e1.

5. González-García AO, Vizzeri G, Bowd C, Medeiros FA, Zangwill LM, Weinreb RN. Reproducibility of RTVue retinal nerve fiber layer thickness and optic disc measurements and agreement with Stratus optical coherence tomography measurements. American Journal of Ophthalmology. 2009;147(6):1067-74. e1.

6. Mo S, Krawitz B, Efstathiadis E, Geyman L, Weitz R, Chui TY, et al. Imaging foveal microvasculature: optical coherence tomography angiography versus adaptive optics scanning light ophthalmoscope fluorescein angiography. Investigative ophthalmology & visual science. 2016;57(9):OCT130-OCT40.

7. Tam J, Dhamdhere KP, Tiruveedhula P, Manzanera S, Barez S, Bearse MA, et al. Disruption of the retinal parafoveal capillary network in type 2 diabetes before the onset of diabetic retinopathy. Investigative Ophthalmology & Visual Science. 2011;52(12):9257-66.
